# Prioritizing non-communicable diseases in the post-pandemic era based on a comprehensive analysis of the GBD 2019 from 1990 to 2019

**DOI:** 10.1038/s41598-023-40595-7

**Published:** 2023-08-16

**Authors:** Jianhao Shu, Weifeng Jin

**Affiliations:** 1 The Second Clinical Medical College, Zhejiang Chinese Medical University, Hangzhou, 310053 China; 2 College of Pharmaceutical Science, Zhejiang Chinese Medical University, Hangzhou, 310053 China

**Keywords:** Diseases, Health care

## Abstract

This study aimed to assess the burden of communicable diseases (CDs) and non-communicable diseases (NCDs) globally, regionally, and nationally from 1990 to 2019, and propose global strategies to transform the public health policy. Using data from the Global Burden of Disease Study (GBD) 2019, we analyzed CDs and NCDs across various factors such as sex, age, year, and location, and evaluate the temporal trends of these diseases with joinpoint analysis. We also examined the differences between regions based on their socio-demographic index (SDI). In 2019, there were 7,862,907 (95% uncertainty interval [UI], 7,183,475 to 8,654,104) deaths from CDs and 42,034,124 (40,081,323 to 43,942,475) deaths from NCDs recorded worldwide. The low SDI region had markedly high age-standardized death and DALY rates of CDs. Although the age-standardized incidence rate of CDs has decreased in about half of the regions since 1990, NCDs have been on the rise in most regions. Over the past 30 years, the global burden of CDs has decreased significantly, while the burden of NCDs has aggrandized to an extent. In the post-pandemic era, effective interventions and cooperation among countries should be promoted to allocate medical resources more reasonably and improve healthcare for NCD patients.

## Introduction

As the COVID-19 pandemic continues to rage around the world, the death toll is rising due to the spread of novel coronavirus and its variants. It has been more than three years since the emergence of COVID-19, and infection prevention and control for COVID-19 have consumed substantial medical resources in various countries, diverting attention from non-communicable diseases (NCDs) and resulting in alarming consequences. According to the World Health Organization (WHO), as of January 2023, the number of deaths reported due to COVID-19 has surpassed 660 million. While communicable diseases (CDs) have historically brought a significant burden to human beings of all ages during outbreaks^1^, the death rate from CDs has dramatically reduced in high-income countries since the beginning of the twentieth century^2^, thanks to medical advances, access to health care, and improved hygiene, especially for lower respiratory infections and diarrheal diseases over the past two decades. Despite advancements in healthcare, low- and middle-income countries (LMICs) continue to face significant burdens from communicable diseases (CDs), particularly related to neglected tropical diseases, HIV infection, tuberculosis, and malaria. This has resulted in persistently high rates of mortality and morbidity in these regions^3^. Furthermore, the 21st-century landscape of social, political, and ecological changes has substantially increased the susceptibility of a vast population to life-threatening acute and chronic infections^4^. This emerging risk scenario presents new challenges for healthcare systems worldwide.

However, the impact of NCDs on human health cannot be ignored. The industrial revolution and associated changes in nutrition, epidemiology, and demographics have had a profound impact on human ecology and biology, leading to significant changes in life-history characteristics. Additionally, genetic variants that were once associated with higher fitness now potentially predispose populations to NCDs, such as Alzheimer’s disease, cancer, and coronary artery disease, through antagonistic pleiotropic effects^5,6^. Global epidemiological studies have demonstrated that chronic NCDs, such as atherosclerosis and metabolic disorders, are the leading causes of premature mortality and morbidity^7^. On 21 September 2022, WHO introduced a report urging international leaders to take immediate steps to tackle NCDs, including cancers, cardiovascular diseases, diabetes, and chronic respiratory diseases, which collectively account for the deaths of 17 million people under the age of 70 worldwide each year, with 86% of these deaths occurring in LMICs^8,9^. The WHO predicts that by 2030, NCDs will account for 77% of the total global burden of diseases^7^.

In this study, our aim was to comprehensively analyze the global burden and trends of CDs and NCDs from 1990 to 2019, explore the double burden of CDs and NCDs during the epidemiological transition, and investigate variations in disease burden among different countries, age groups, and genders, with the goal of informing health systems in prioritizing prevention, monitoring, and disease management efforts. Furthermore, we examined the current post-pandemic era and proposed corresponding strategies, offering valuable insights for policymakers, public health practitioners, and researchers.

## Materials and methods

### Overview

The GBD 2019 report provides estimates for 369 diseases and injuries and 87 risk factors across 204 countries and regions. It includes a wide range of models for disease and injury outcomes, such as the Cause of Death Ensemble Model, Spatio-temporal Gaussian Process Regression, and Bayesian meta-regression tool.

In this study, "non-communicable diseases" refer to the aggregate of all non-communicable diseases included in the GBD 2019 database. The term encompasses various health conditions, including Cardiovascular diseases, Neoplasms, Chronic respiratory diseases, Diabetes and kidney diseases, Digestive diseases, and other non-communicable disease categories. The term "communicable diseases" refers to the aggregate of all communicable diseases included in the GBD database. This encompasses a wide range of diseases, including but not limited to Lower respiratory infections, Diarrheal diseases, Tuberculosis, HIV/AIDS, Malaria, and nearly 30 other types of infectious diseases. To collect data on CDs and NCDs from 1990 to 2019, we used the GBD Results Tool (https://vizhub.healthdata.org/gbd-results/) and considered factors such as location, age, sex, and gender. The burden of disease in this paper was measured using four indicators from the GBD: incidence, prevalence, death, and DALY, with all age-standardized and age-specific rates, including 95% uncertainty interval data, available. The study design used in this research was ecological in nature.

### Join point regression analysis

The Join point regression model is a linear statistical tool that helps assess trends in the burden of CDs and NCDs over time. Unlike traditional trend analysis, which is based on linear trends and lacks objectivity, the model estimates the pattern of change in incidence, prevalence, death, and disability-adjusted life year (DALY) rates. The model calculates the turning point of the moving trend by minimizing the sum of squares of the residual error between the estimated and actual values.

In this study, we employed Join point regression analysis (version 4.9.1.0, April 2022) to determine the estimated annual percentage change (APC) and quantify the trend of disease from 1990 to 2019. We used the term "increasing" to describe trends when both the estimated APC and the lower limit of its 95% uncertainty interval were greater than 0. Conversely, we used the term "reduced" to describe trends when both the estimated APC and the upper limit of its 95% uncertainty interval were less than 0. If neither of these criteria was met, we used the term "stable." Statistical significance was defined as p < 0.05.

### Software

This article utilized RStudio, an integrated development environment (IDE) for the R programming language, version 4.2.0, for creating visualizations, and relies on specific R packages, such as dplyr, ggplot2, reshape2, ggmap, rgdal, maps, pheatmap, and more.

## Results

### Global burden of communicable diseases

In 2019, there were a total of 26,129,005,559 (24,320,309,554 to 28,173,220,203) new cases of CDs, out of which 7,862,907 (7,183,475 to 8,654,104) resulted in death, 3,142,340,323 (3,073,416,265 to 3,213,648,653) were prevalent cases and 420,392,536 (459,373,669 to 384,040,485) were cases of disability-adjusted life years (DALYs) (Table 1). The incidence, death, and DALY rates of CDs were higher in men than women, while the prevalence was slightly higher in women (Table 1). Furthermore, the < 20 years age group had the highest number of incident cases across all age groups, which remained relatively stable (Supplementary Fig. 1).

**Table 1:** Global burden of communicable diseases in 2019.

Location	Sex	Incidence		Deaths		Prevalence		DALYs	
		Count (*10^5^)	ASIR (per 100,000)	Count (*10^5^)	ASDR (per 100,000)	Count (*10^5^)	ASPR (per 100,000)	Count (*10^5^)	Age-standardized DALY rate (per 100,000)
Global	Both	261,290.06 (243,203.1 to 281,732.2)	342,348.2 (317,830.7 to 369,569.47)	78.63 (71.83 to 86.54)	105.62 (96.63 to 116.65)	31,423.4 (30,734.16 to 32,136.49)	39,966.92 (39,126.62 to 40,811.95)	4203.93 (3840.4 to 4593.74)	5814.3 (5296.23 to 6407.37)
High SDI	Both	32,629.03 (30,154.84 to 35,597.85)	350,083.47 (318,838.07 to 386,646.89)	4.59 (3.93 to 4.93)	21.18 (18.82 to 22.38)	2982.89 (2833.03 to 3137.74)	26,004.64 (24,800.21 to 27,207.29)	89.84 (82.94 to 97.57)	649.65 (596.06 to 718.2)
High-middle SDI	Both	43,031.18 (40,045.49 to 46,395.71)	316,718.88 (291,947.18 to 344,846.16)	5.1 (4.74 to 5.36)	29.63 (27.76 to 31.23)	5602.09 (5422.58 to 5786.74)	35,751.87 (34,597.47 to 36,807.55)	194.28 (182.6 to 204.91)	1358.16 (1276.13 to 1422.2)
Middle SDI	Both	76,890.33 (71,501.35 to 83,057.62)	329,226.01 (305,069.06 to 356,358.29)	15.15 (14.07 to 16.26)	70.57 (65.28 to 76.31)	10,624.67 (10,334.53 to 10,891.53)	42,852.45 (41,711.28 to 43,869.74)	722.17 (676.61 to 759.91)	3251.96 (3043.88 to 3435.68)
Low-middle SDI	Both	64,371.45 (59,723.11 to 69,404.61)	367,695.42 (342,958.48 to 394,827.7)	24.85 (22.32 to 27.99)	179.16 (158.38 to 205.6)	7102.23 (6975.96 to 7223.79)	40,126.74 (39,450.84 to 40,770.68)	1257.31 (1163.95 to 1356.07)	7644.84 (7078.04 to 8259.7)
Low SDI	Both	44,204.22 (40,756.28 to 47,744.2)	387,443.51 (363,221.69 to 413,261.72)	28.9 (25.55 to 33.17)	341.58 (306.18 to 383.17)	5091.37 (5066.22 to 5108.8)	46,722.78 (46,471.34 to 46,789.4)	1937.75 (1708.52 to 2223.91)	15,592.11 (13,909.73 to 17,527.34)

**Figure 1:**
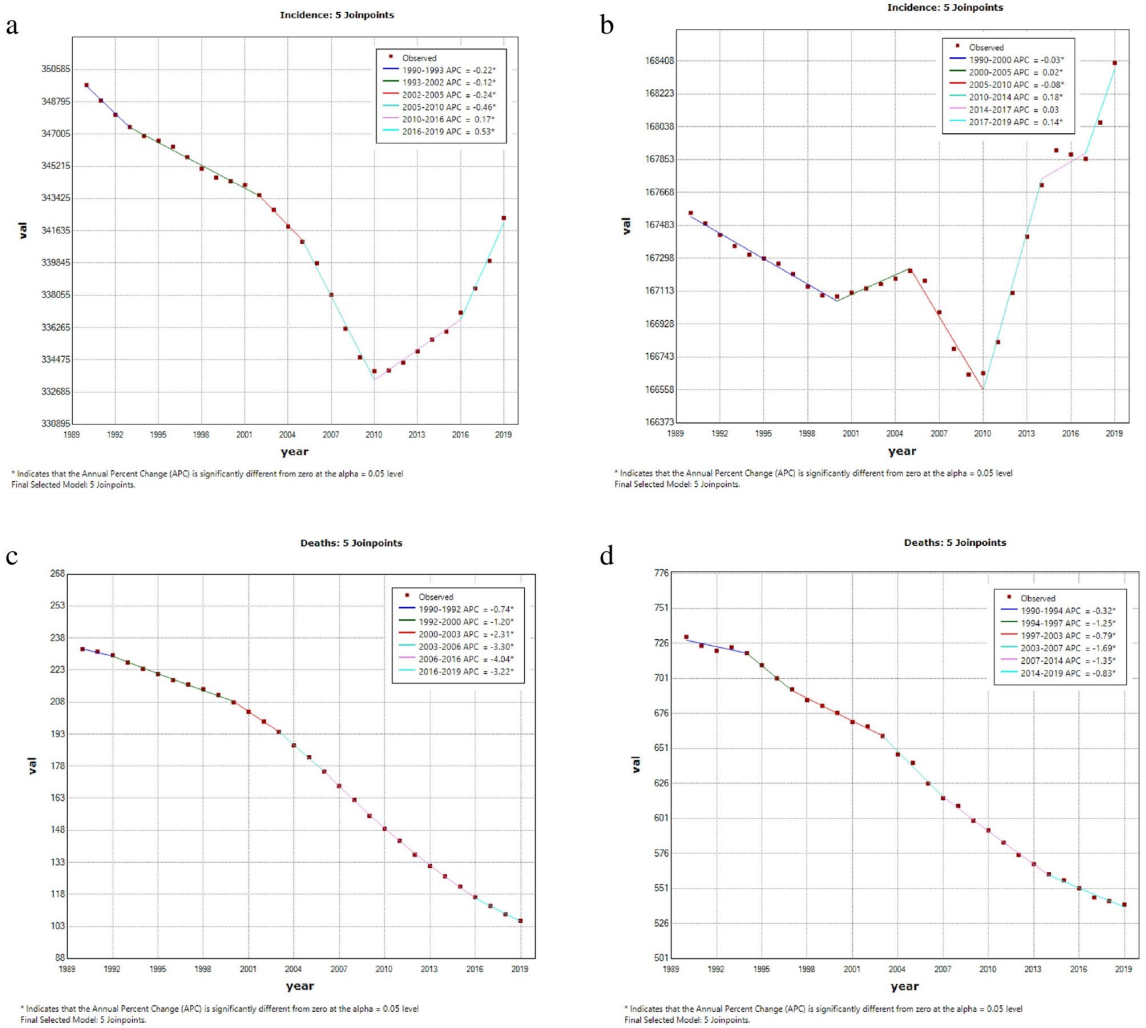

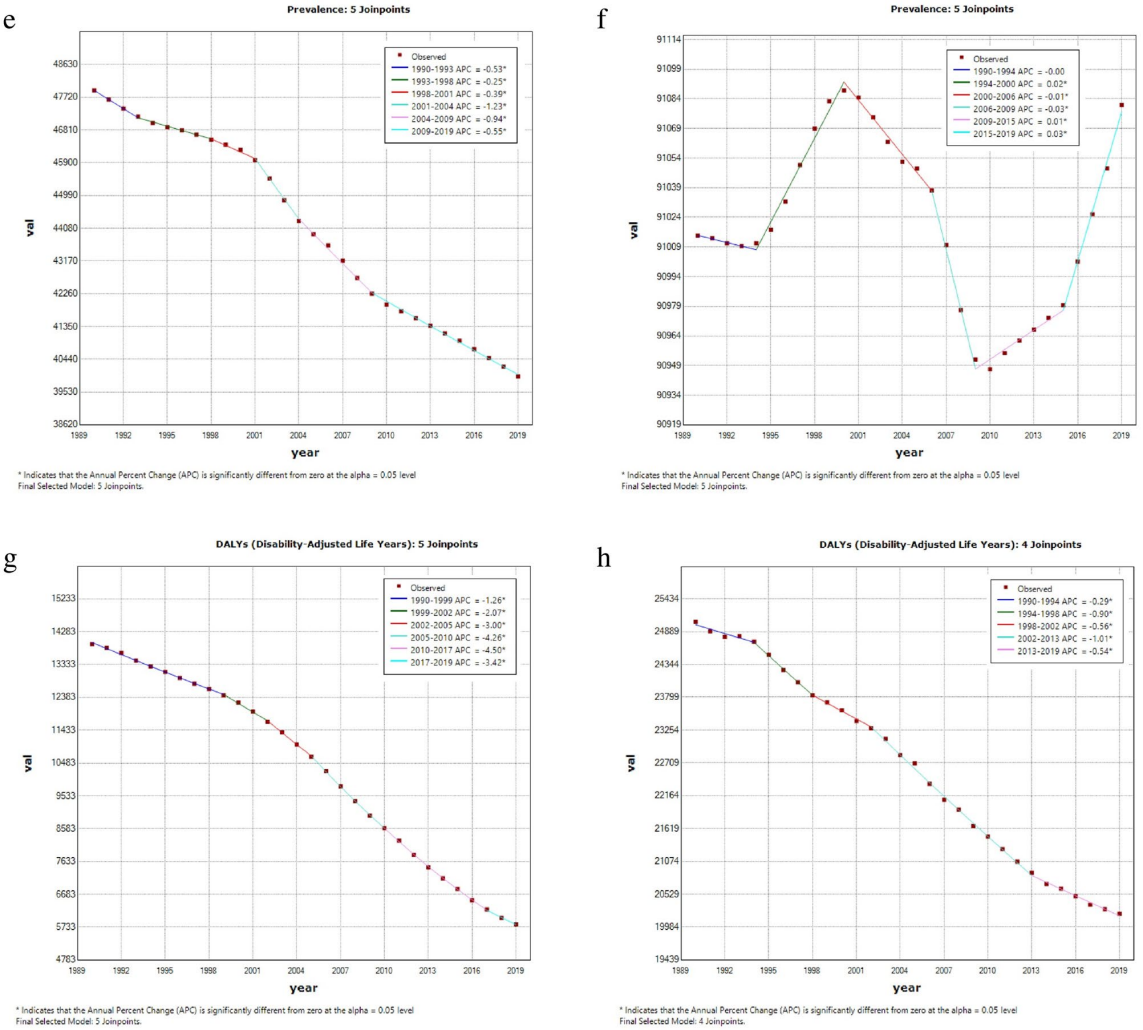
Global trends for age-standardized rates (per 100,000 population) of communicable diseases and NCDs from 1990 to 2019. (**a**) age-standardized incidence rate of communicable disease; (**b**) age-standardized incidence rate of non-communicable disease; (**c**) age-standardized death rate of communicable disease; (**d**) age-standardized death rate of non-communicable disease; (**e**) age-standardized prevalence rate of communicable disease; (**f**) age-standardized prevalence rate of non-communicable disease; (**g**) age-standardized DALYs rate of communicable disease; (**h**) age-standardized DALYs rate of noncommunicable disease. *DALY* disability-adjusted life year.

In the past 30 years, there has been a global decrease in age-standardized death, prevalence, and DALY rates of CDs. As showed in Fig. 1, the global age-standardized death rate (ASDR) decreased most significantly between the year 2006 and 2016 (APC =  − 4.04%; [95% CI − 4.10% to − 3.97%]; p < 0.001). However, the global age-standardized incidence rate (ASIR) initially decreased and then increased (Fig. 1).

### Global burden of non-communicable diseases

In 2019, there were 13,065,679,568 (12,535,051,312 to 13,611,832,793) new cases of NCDs, 42,034,124 (40,081,323 to 43,942,475) deaths, 7,104,354,703 (7,040,626,561 to 7,163,713,567) prevalent cases and 1,620,165,811 (1,429,471,142 to 1,816,695,861) cases of DALYs (Table 2). Women had higher incidence and prevalence rates of NCDs than men, while men had slightly higher death and DALY rates (Table 2). In terms of age groups, the highest number of incident cases was observed in the < 20 years age group, whereas the lowest was observed in the > 80 years age group. Overall, there was an upward trend (Supplementary Fig. 1).

**Table 2:** Global burden of non-communicable diseases in 2019.

Location	Sex	Incidence		Deaths		Prevalence		DALYs	
		Count (*10^5^)	ASIR (per 100,000)	Count (*10^5^)	ASDR (per 100,000)	Count (*10^5^)	ASPR (per 100,000)	Count (*10^5^)	Age-standardized DALY rate (per 100,000)
Global	Both	130,656.8 (125,350.51 to 136,118.33)	168,397.03 (161,059.69 to 176,059.97)	420.34 (400.81 to 439.42)	539.62 (515.14 to 563.36)	71,043.55 (70,406.27 to 71,637.14)	91,080.83 (90,169.62 to 91,943.29)	16,201.66 (14,294.71 to 18,166.96)	20,204.91 (17,826.02 to 22,636.76)
High SDI	Both	17,666.85 (17,112.36 to 18,271.2)	164,187.74 (157,384.3 to 171,300.71)	78.59 (77.38 to 80.08)	384.27 (377.91 to 391.2)	9460.34 (9401.78 to 9515.73)	89,547.13 (88,610.54 to 90,458.36)	2563.75 (2243.12 to 2919.95)	17,507.2 (14,927.12 to 20,439.22)
High-middle SDI	Both	23,639.81 (22,820.97 to 24,542.92)	161,016.21 (153,952.54 to 168,272.74)	102.54 (96.89 to 107.92)	524.96 (496.79 to 551.71)	13,296.52 (13,202.59 to 13,381.09)	89,882.75 (88,861.62 to 90,802.85)	3410.28 (3031.29 to 3814.72)	18,667.65 (16,416.46 to 21,033.27)
Middle SDI	Both	39,193.76 (37,659.92 to 40,875.68)	163,896.57 (156,789.82 to 171,646.73)	127.37 (119.21 to 135.81)	589.79 (554.01 to 626.59)	22,050.36 (21,858.31 to 22,222.28)	90,722.94 (89,749.07 to 91,589.75)	4991.81 (4400.38 to 5615.25)	20,376.1 (18,013.35 to 22,855.4)
Low-middle SDI	Both	30,285.26 (28,861.42 to 31,700.28)	174,860.77 (167,267.34 to 182,599.1)	79.67 (73.65 to 85.67)	649.36 (600.94 to 695.24)	16,110.09 (15,943.51 to 16,268.49)	91,731.15 (90,820.7 to 92,590.57)	3460.84 (3035.8 to 3907.22)	23,021.95 (20,400.94 to 25,774.33)
Low SDI	Both	19,279.98 (18,122.24 to 20,477.87)	178,207.15 (169,841.02 to 186,829.01)	31.93 (29.13 to 34.99)	650.71 (600.47 to 701.27)	10,085.5 (9947.32 to 10,225.05)	92,251.39 (91,389.14 to 93,101.48)	1765.48 (1529.52 to 2014.06)	23,340.05 (20,727 to 26,140.43)

In the period from 1990 to 2019, there was a substantial decline in the age-standardized death and DALY rates of NCDs worldwide. Notably, as depicted in Fig. 1, the global ASDR decreased most significantly between 2003 and 2007 (APC =  − 1.69%; [95% CI − 2.05% to − 1.32%]; p < 0.001). However, trends in global ASIR and age-standardized prevalence rate (ASPR) were relatively unstable with slight rate fluctuations (Fig. 1).

### Regional and national burden of communicable diseases

**Figure 2:**
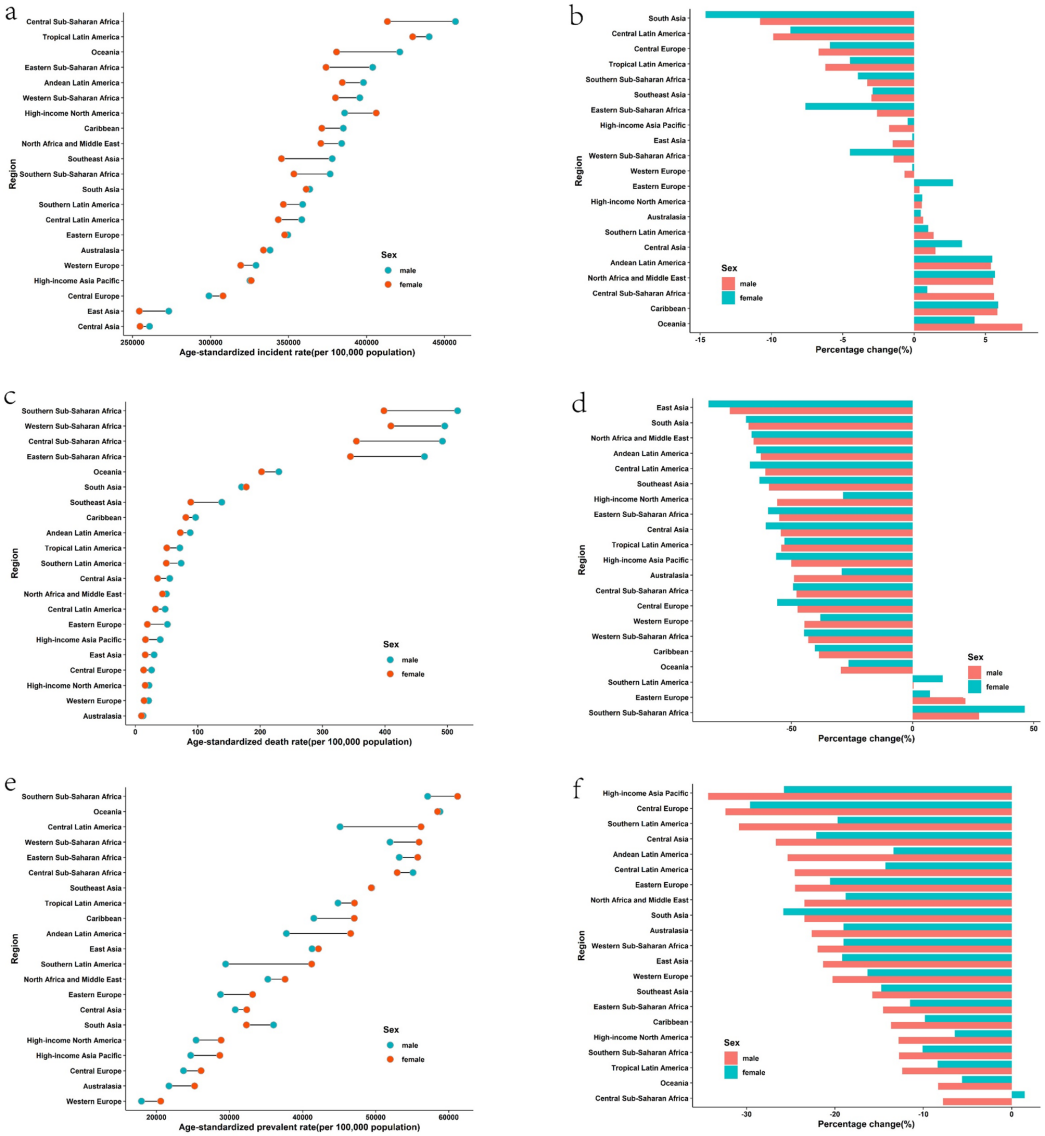

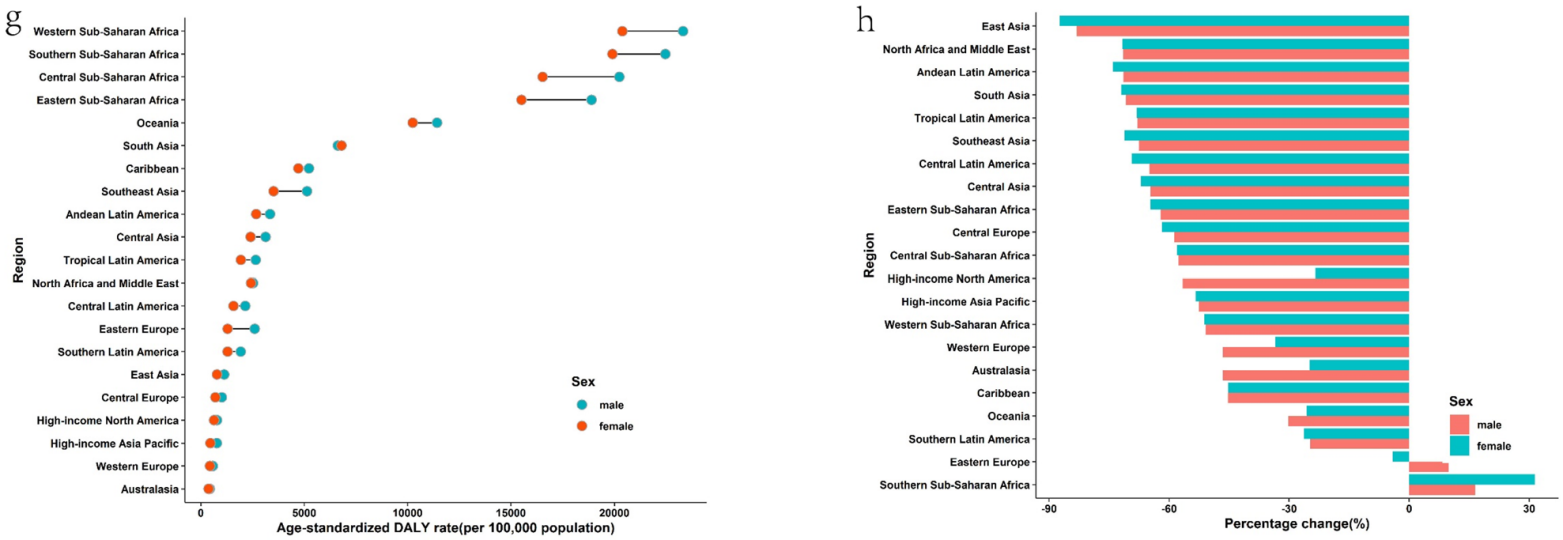
Regional age-standardized incidence rates (per 100,000 population) of communicable disease in 2019 and their sex-specific percentage changes from 1990 to 2019. (**a**) age-standardized incidence rate in 2019; (**b**) percentage change in age-standardized incidence rate, 1990–2019; (**c**) age-standardized death rate in 2019; (**d**) percentage change in age-standardized death rate, 1990–2019; (**e**) age-standardized prevalence rate in 2019; (**f**) percentage change in age-standardized prevalence rate in 2019; (**g**) age-standardized DALY rate in 2019; (**h**) percentage change in age-standardized DALY rate, 1990–2019. *DALY* disability-adjusted life year.

In 2019, the region with the highest ASIR of CDs was Central Sub-Saharan Africa, followed by tropical Latin America. The highest ASPR and ASDR were both observed in southern Sub-Saharan Africa (Fig. 2 and Supplementary Table 3). Since 1990, the ASIR of CDs had decreased in about half of the regions, with South Asia exhibiting the largest decline. However, in another half of the regions, the ASIR had increased, with the largest increase observed in Oceania.

To provide a clearer picture of the situation at the national level, it is worth noting that in 2019, Central Africa, Kenya, and Angola were the top three countries with the highest ASIR in that order. Lesotho, Central Africa, and Mozambique were the top three countries with the highest ASDR, while Liberia, Uganda, and Burundi were the top three countries with the highest ASPR. The top three countries with the highest age-standardized DALY rates were Lesotho, Central Africa, and Mozambique (Fig. 4 and Supplementary Table 7). It is notable that all the countries mentioned above are located in Africa.

### Regional and national burden of non-communicable diseases

**Figure 3:**
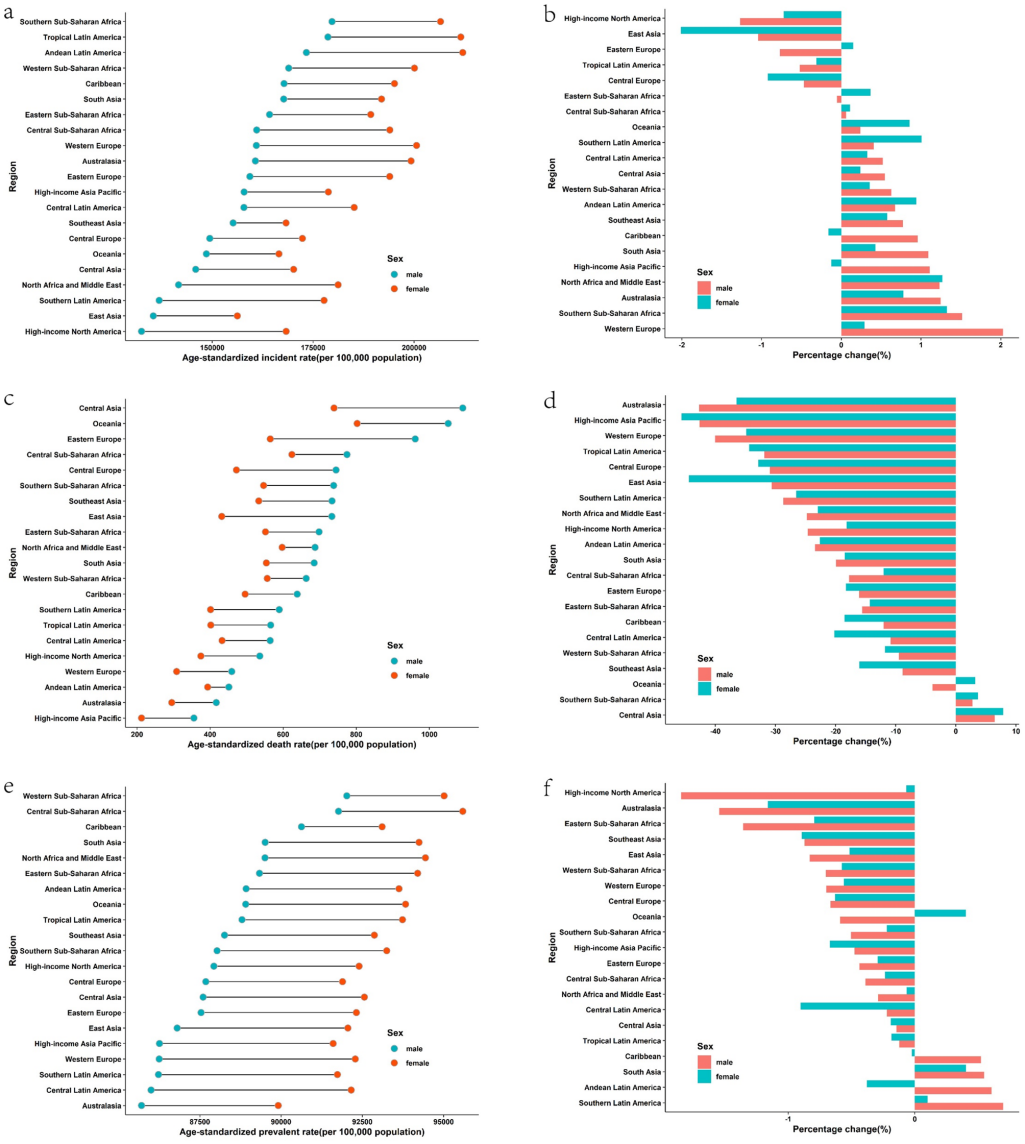

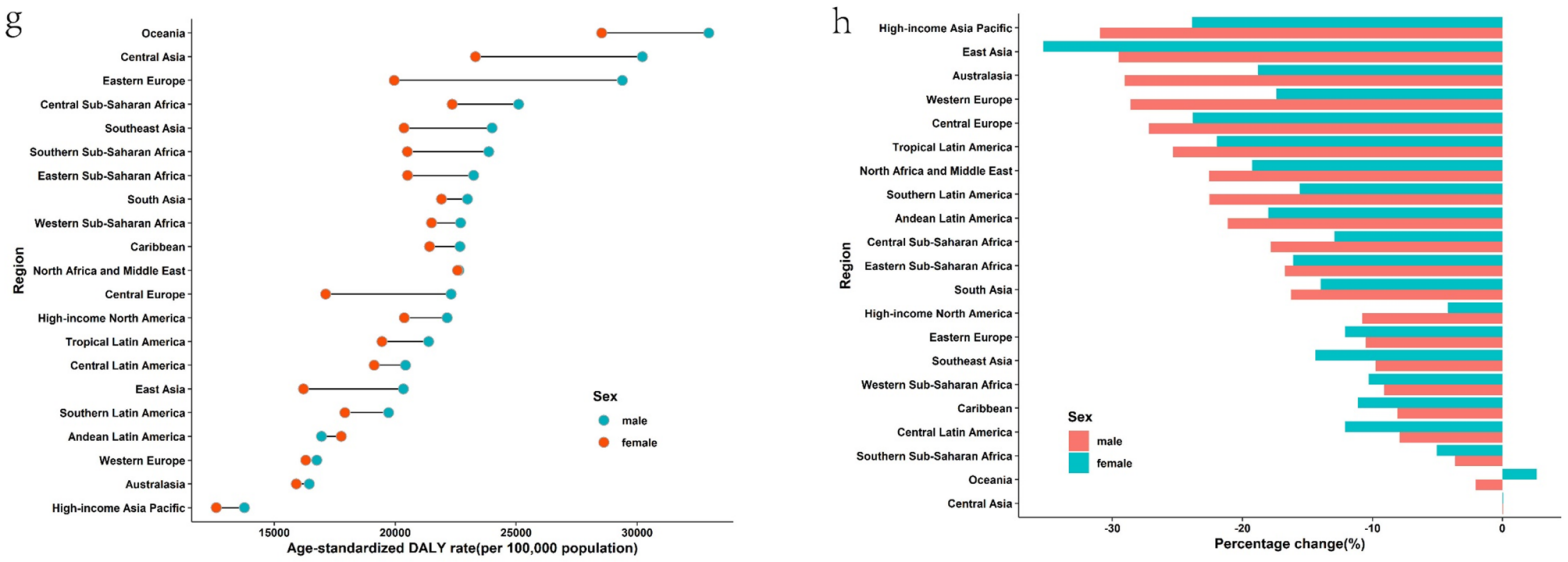
Regional age-standardized incidence rates (per 100,000 population) of non-communicable diseases in 2019 and their sex-specific percentage changes from 1990 to 2019. (**a**) The age-standardized incidence rate in 2019; (**b**) percentage change in age-standardized incidence rate, 1990–2019; (**c**) age-standardized death rate in 2019; (**d**) percentage change in age-standardized death rate, 1990–2019; (**e**) age-standardized prevalence rate in 2019; (**f**) percentage change in age-standardized prevalence rate in 2019; (**g**) age-standardized DALY rate in 2019; (**h**) percentage change in age-standardized DALY rate, 1990–2019. *DALY* disability-adjusted life year.

In 2019, the highest ASIR of NCDs was recorded in southern Sub-Saharan Africa and tropical Latin America, while the highest ASDR was observed in Central Asia, followed by Oceania. The highest prevalence was found in western and central Sub-Saharan Africa, and the highest DALY rate was reported in Oceania and Central Asia, respectively (Fig. 3 and Supplementary Table 4). Since 1990, ASIR for NCDs has increased in more than half of the regions, with East Asia exhibiting the greatest decline and southern Sub-Saharan Africa showing the greatest incline.

**Figure 4:**
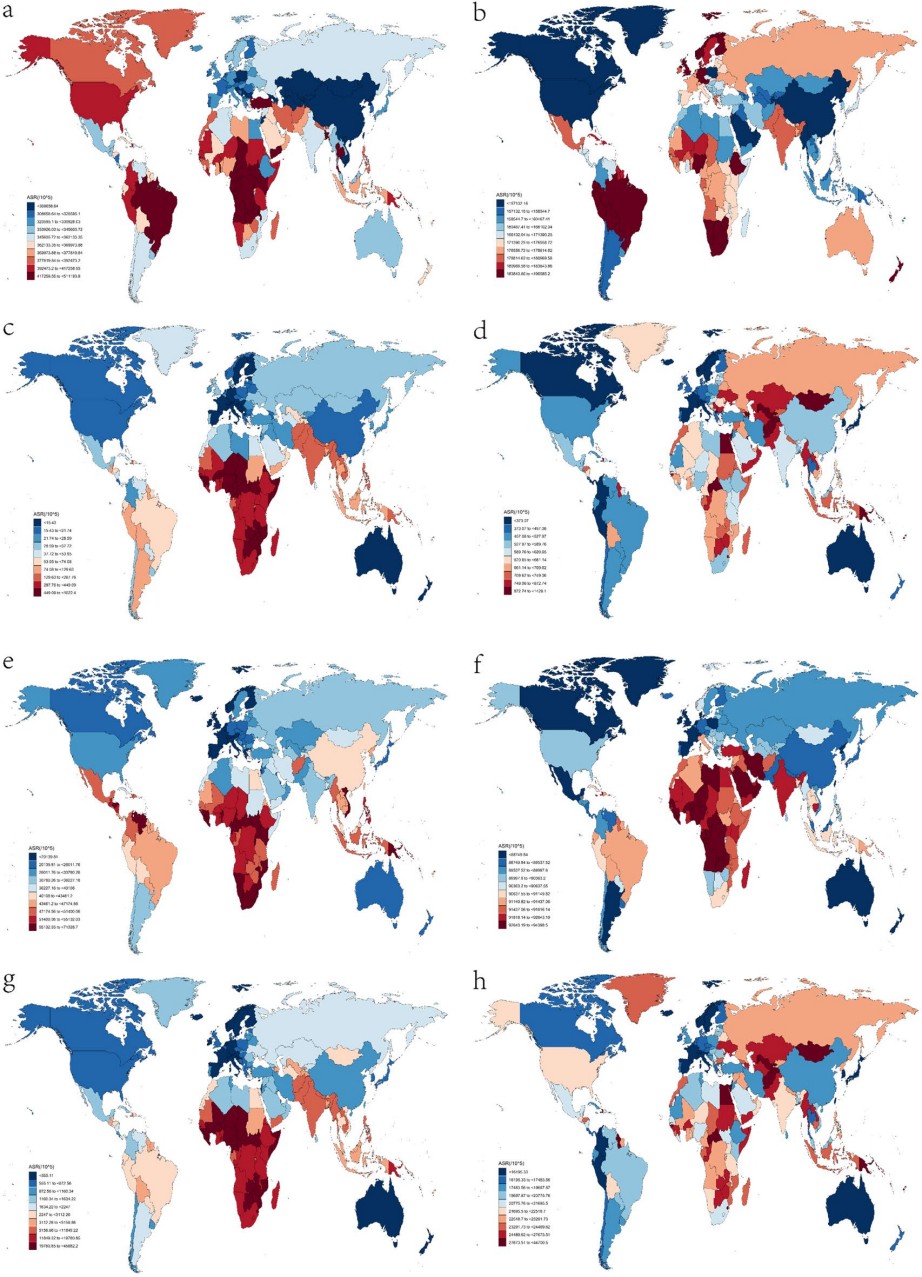
National age-standardized rates (per 100,000 population) of communicable diseases and NCDs in 2019. (**a**) The age-standardized incidence rate of communicable diseases; (**b**) age-standardized incidence rate of non-communicable diseases; (**c**) age-standardized death rate of communicable diseases; (**d**) age-standardized death rate of non-communicable diseases; (**e**) age-standardized prevalence rate of communicable diseases; (**f**) age-standardized prevalence rate of non-communicable diseases; (**g**) age-standardized DALYs rate of communicable diseases; (**h**) age-standardized DALYs rate of non-communicable diseases. *DALY* disability-adjusted life year. The original data was obtained from the GBD studies. There may be some problems with the regional partition, albeit it was not the focus of the present study.

At the national level, in 2019, Ecuador, Brazil, and South Africa had the highest ASIR for NCDs, while the Solomon Islands, Kiribati, and Nauru had the highest ASDR. Additionally, Nigeria, Burkina Faso, and the Central African Republic had the highest ASPR, and the Solomon Islands, Kiribati, and Nauru had the highest age-standardized DALY rates. (See Fig. 4 and Supplementary Table 8) It is noteworthy that all the countries mentioned are mainly concentrated in Africa.

### Communicable diseases and SDI

**Figure 5:**
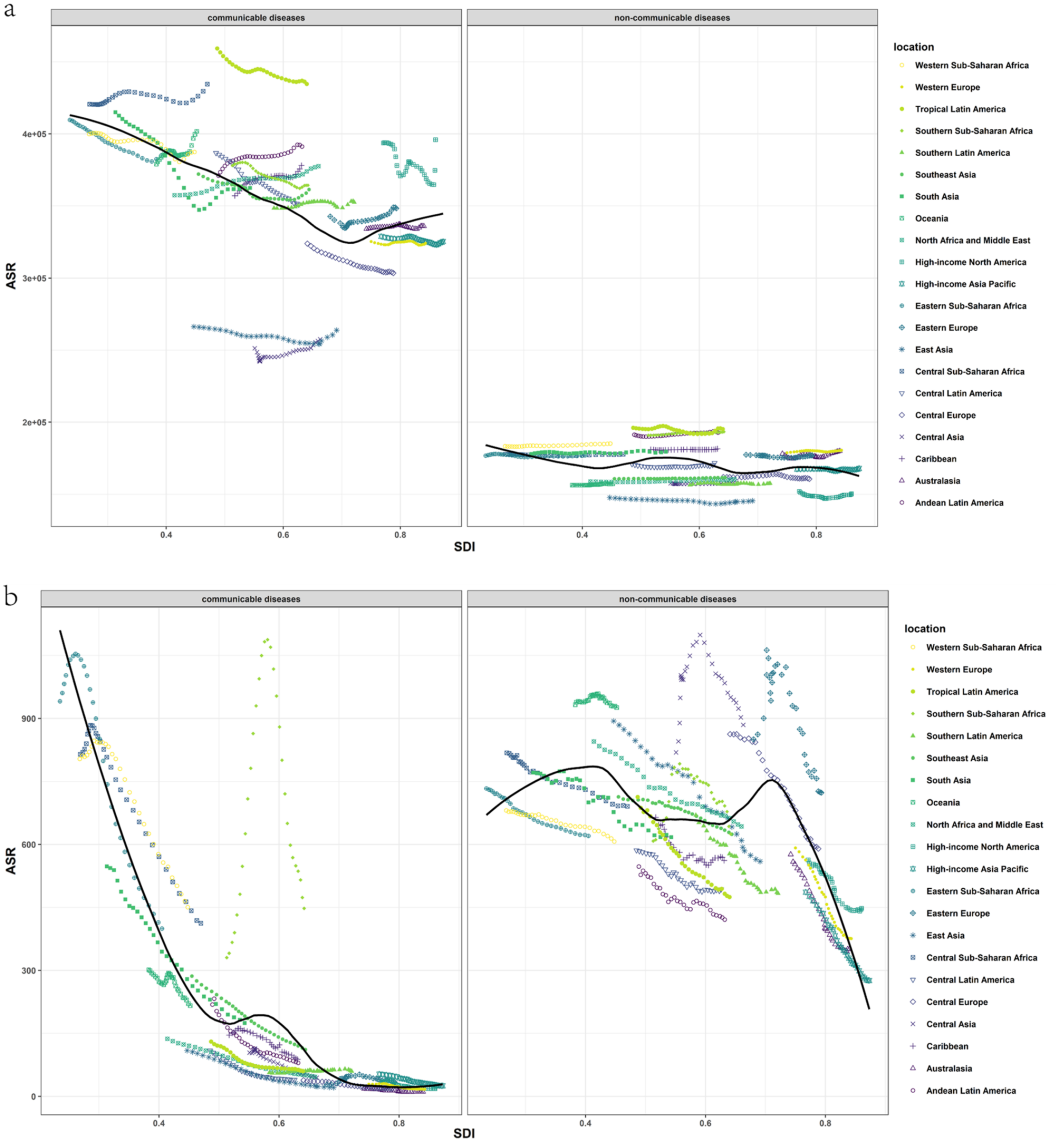

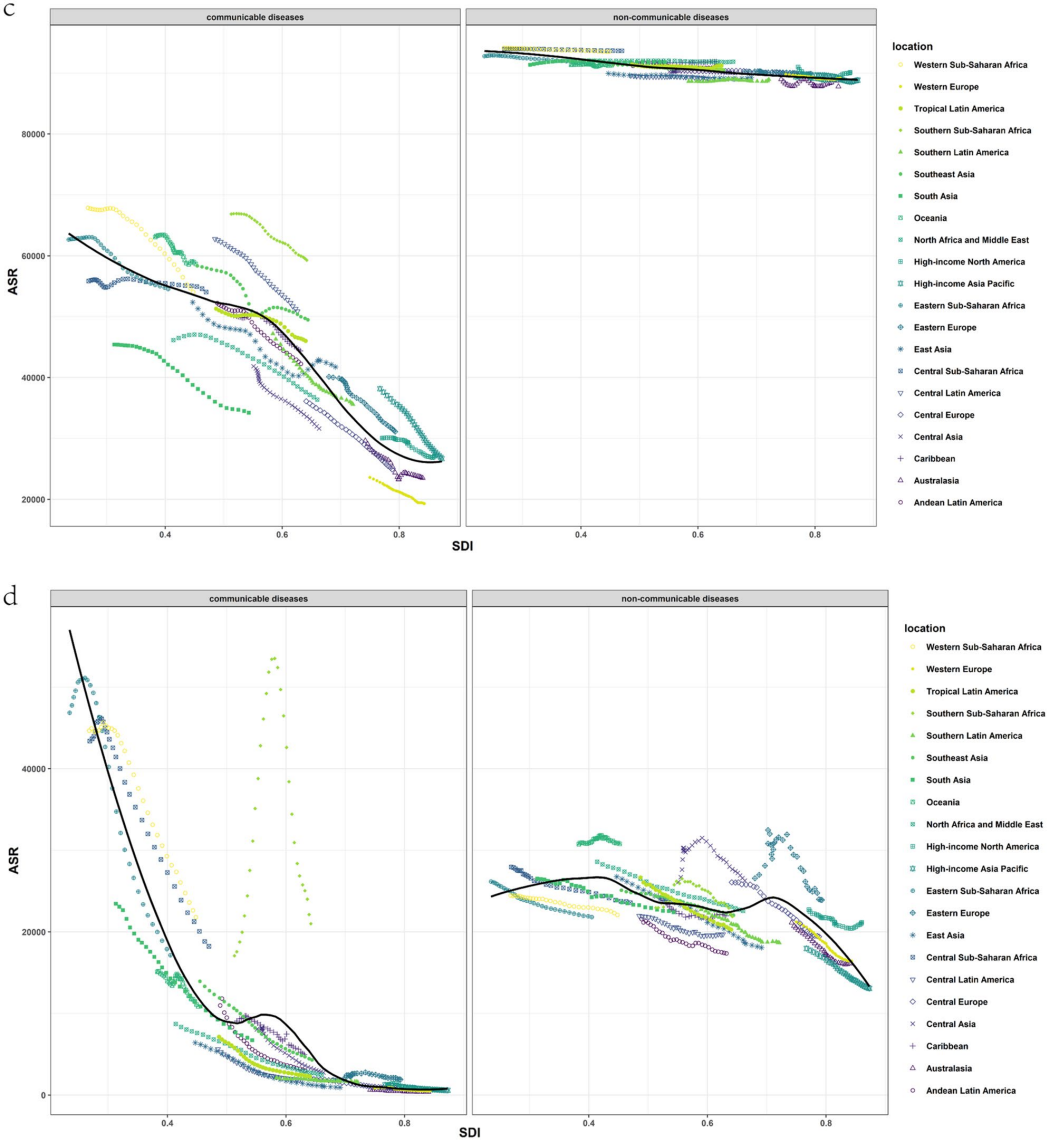
Trends for age-standardized rates (per 100,000 population) of communicable diseases and NCDs among 21 regions by SDI, 1990–2019. (**a**) Age-standardized incidence rate; (**b**) age-standardized death rate; (**c**) age-standardized prevalence date; (**d**) age-standardized DALY rate. *SDI* Socio-demographic Index, *DALY* disability-adjusted life year.

The burden of CDs varies considerably based on the socio-demographic index (SDI). As demonstrated in Fig. 5, the first half of ASIR decreased with increasing SDI, but it increased when SDI approached 1, and there were significant differences between distinct regions. According to the SDI, some regions displayed decreasing ASIR, while others presented increasing ASIR or were not monotonically associated with SDI. In contrast, the associations between SDI, age-standardized prevalence, death, and DALY rate illustrated similar trends. As the SDI increased, ASDR, ASPR, and age-standardized DALY rate dramatically decreased, and areas with higher SDI generally exhibited lower ASDR, ASPR, and age-standardized DALY rate (Fig. 5). Since 1990, ASDR, ASPR, and age-standardized DALY rate have declined across all SDI quintiles (Supplementary Fig. 2). Countries with low SDI experienced the largest decline in age-standardized death and DALY rates, whereas countries with high SDI and high-middle SDI remained stable at low levels. ASIR has been increasing in all SDI countries in recent years, although it continues to decrease compared to the figures from 1990 (Supplementary Fig. 2).

### Non-communicable diseases and SDI

The association between NCDs and SDI was less clear compared to CDs, but overall trends for each indicator showed a decrease with an increase in SDI. However, there were significant differences between regions at the same level of SDI. Figure 5 shows that for different regions, ASIR, ASDR, ASPR, and age-standardized DALY rates were considerably different.

Since 1990, there were no significant changes in age-standardized incidence and prevalence rates across all SDI quintiles. However, the decline in ASDR and DALY rates was substantial, with countries with high SDI exhibiting the greatest decline (Supplementary Fig. 2). In contrast, the upward trend in ASIR and ASPR was more pronounced in high and high-middle SDI countries in recent years (Supplementary Fig. 2).

### The burden of different communicable diseases

**Figure 6:**
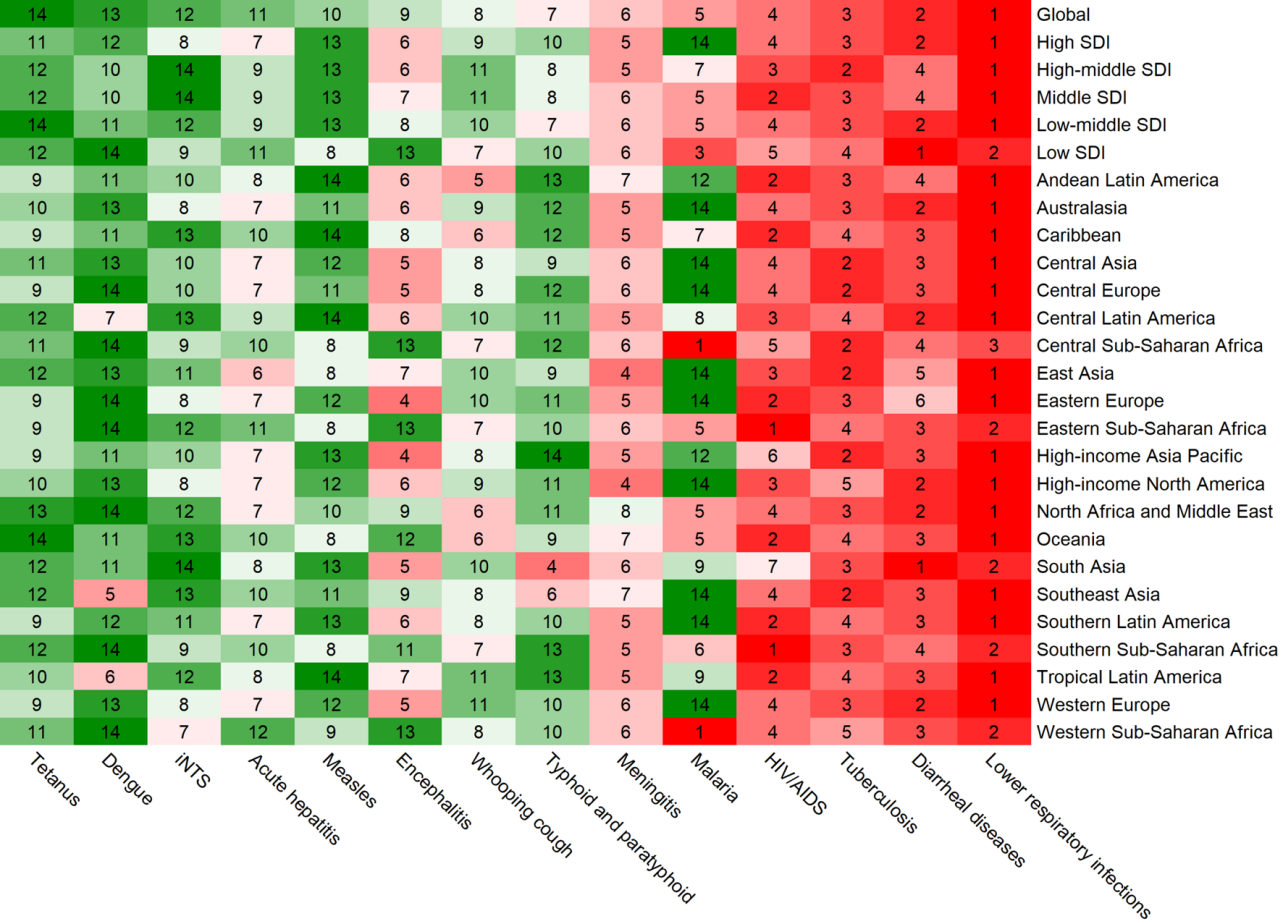
Global and regional communicable diseases ranked by the total number of deaths in 2019.

**Figure 7:**
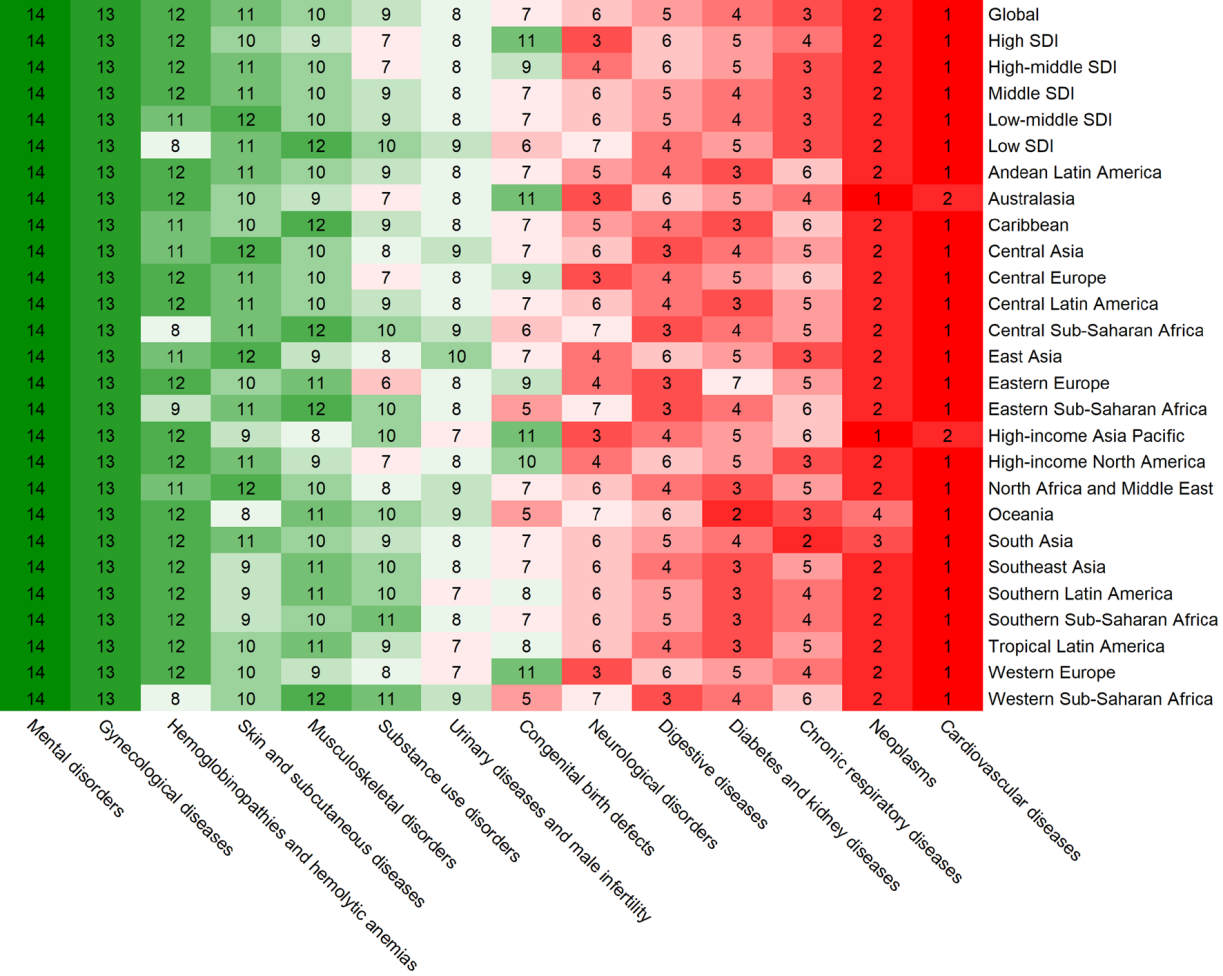
Global and regional non-communicable diseases ranked by the total number of deaths in 2019.

In line with the previous study^10^, lower respiratory infections were found to account for the highest number of deaths and DALYs among CDs in 2019. However, diarrhea and tuberculosis were the leading causes of incident and prevalent cases, respectively (Supplementary Table 5, Fig. 6, and Supplementary Figs. 3–5). The other three common causes of death from CDs were diarrhea, tuberculosis, and HIV/AIDS (Supplementary Table 5). Figure 7 illustrates the global and regional rankings of different CDs based on the number of deaths, while incident cases, prevalent cases, and DALYs were demonstrated in Supplementary Figs. 3–5. Tetanus had the greatest decline in age-standardized incidence, prevalence, death, and DALY rate, whereas AIDS had the most significant increase over the past 30 years (Supplementary Fig. 11).

Furthermore, the burden of different CDs varied by age. In 2019, the burden of most CDs such as the number of incident cases, deaths, prevalent cases, and DALYs was highest in children aged < 20 years, with tuberculosis and AIDS being exceptions. Specifically, AIDS mainly affected the 20 to 39 age group, as shown in Supplementary Fig. 9.

### The burden of different non-communicable diseases

Among the different NCDs, cardiovascular diseases were responsible for the highest number of deaths and DALYs globally in 2019. However, skin and subcutaneous diseases accounted for the largest number of incident cases, and neurological diseases accounted for the largest number of prevalent cases (Supplementary Table 6, Fig. 7, and Supplementary Figs. 6–8). The other three common causes of death were cancers, chronic respiratory diseases, and diabetes and renal diseases (Supplementary Table 6). Supplementary Fig. 11 depicts the global and regional rankings of different NCDs based on the number of deaths; the rankings according to the number of incident cases, the number of prevalent cases, and DALYs were presented in Supplementary Figs. 6–8. Over the past 30 years, the greatest decline in ASIR has been for cardiovascular diseases, while the greatest decline in death and DALY has been for congenital birth defects. The greatest decline in prevalence has been for chronic respiratory diseases. Nevertheless, the increases in ASIR, ASPR, and age-standardized DALY rate were the most pronounced for diabetes and renal diseases (Supplementary Fig. 12).

The relationship between age and the types of NCDs differed from that of CDs. In 2019, with the exception of congenital birth defects, hemoglobinopathies, and hemolytic anemia, the burden of most NCDs in terms of the number of incident cases, deaths, prevalent cases, and DALYs was markedly lower in the < 20 years age group than that of CDs (Supplementary Fig. 10). Most deaths caused by NCDs occurred in the 60–79 years age group. Deaths caused by nervous system diseases were mainly in the > 80 years age group, while those caused by mental disorders were mainly in the 20–39 years age group.

## Discussion

Despite improvements in global health and healthcare access, there remains a significant risk of communicable disease outbreaks. Examples include the severe acute respiratory syndrome coronavirus outbreak in 2003, the Middle East respiratory syndrome coronavirus outbreak in 2012, and the ongoing pandemic of COVID-19. However, the burden of NCDs should not be underestimated. According to the 2019 Global Health Estimates, ischemic heart disease, stroke, and chronic obstructive pulmonary disease are the top three causes of death worldwide^11^. Our study found that in 2019, there were 26.1 billion new cases and 7.86 million deaths globally due to CDs, and 13 billion new cases and 42.03 million deaths due to NCDs. Certain regions, such as Southern Sub-Saharan Africa, are faced with the challenge a double burden of CDs and NCDs^12,13^.

### The burden of communicable and non-communicable diseases at different levels

The global burden of disease shows that NCDs are responsible for more deaths, prevalence, and DALYs than CDs, while the incidence of CDs is higher. Over time, the age-standardized death, prevalence, and DALY rate of CDs have decreased significantly, and incidence has also shown a declining trend, although it has demonstrated signs of increasing in recent years. Age-standardized death and DALY rate for NCDs have also decreased, consistent with previous study^14^, but the incidence and prevalence have fluctuated and kept increasing in recent years. When focusing on absolute values, due to population growth, the number of incident and prevalent cases of both CDs and NCDs has increased each year, while the number of deaths and DALYs due to CDs has decreased each year, and the number of deaths and DALYs due to NCDs increased each year, which is consistent with the research findings of Christopher et al. in 2013^15^. The burden of CDs is mainly concentrated in the younger age group (< 20 years old), while the burden of incidence and prevalence of NCDs is mainly concentrated in younger age group (< 20 years old), and the burden of death and DALY is mainly concentrated in older age groups (60–79 years old and > 80 years old).

At regional and national levels, the burden of both CDs and NCDs was found to be higher in the African region and countries, especially in Sub-Saharan Africa, while the burden was lower in North America, East Asia, and Oceania, although North America had a slightly higher ASIR for CDs. Over the past 30 years, the age-standardized incidence rate of about half of the regions with CDs have shown a downward trend, while most of the NCDs have shown an upward trend. Other indicators such as ASDR, ASPR and age-standardized DALY rate have shown a downward trend in most areas. Previous studies have highlighted the increasing burden of disease in Africa, such as Bigna et al. who pointed out that the burden of NCDs in Sub-Saharan Africa has dramatically increased in the past 20 years^16^, Gouda et al. who also stated that NCDs in Sub-Saharan Africa pose an increasing challenge to health systems^17^ and Modjadji et al. who had described the challenge of addressing multiple disease burdens in Africa, characterized by high rates of CDs such as HIV, AIDS, and tuberculosis, as well as NCDs such as cardiovascular disease and diabetes^18^.

Our study found that diarrhea was the CD with the highest burden, while cardiovascular diseases were the NCD with the highest burden, as estimated by four different comprehensive evaluation methods. Diarrhea is a leading cause of incidence and death among infants and young children worldwide, particularly in developing countries^19^, and rotavirus is the the most common cause of pediatric diarrhea^20^. Our study showed that globally, there were approximately 1.53 million deaths due to diarrhea in 2019 across all age groups. However, the burden of diarrhea incidence is even greater. Inadequate hygiene, sanitation, and access to clean water are major risk factors for the high burden of diarrheal diseases in developing countries^20,21^. Furthermore, some studies suggest that climate change may exacerbate the risk of diarrheal diseases, particularly in areas with heavy rainfall and high temperatures^22,23^.

Several bacterial, viral, parasitic, fungal, and non-communicable diarrheal agents have been identified, but 30% to 40% of diarrheal cases remain undiagnosed^19^. Shigella infection is a major cause of diarrheal deaths in children in low—and middle-income countries^24,25^. Cholera, caused by Vibrio cholerae, is another severe diarrheal disease that can quickly become fatal if left untreated. It is usually transmitted through contaminated water and human-to-human contact, and is most prevalent in the Ganges Delta of the Bay of Bengal, Bangladesh and India^26,27^. As previously reported by Roth et al., cardiovascular diseases, particularly ischemic heart disease and stroke, remain the leading cause of death and disability worldwide^28^. Our study also found that in 2019, the number of deaths due to cardiovascular disease globally across all age groups was about 18.56 million. Feigin et al.’s study on stroke similarly revealed that although the incidence, prevalence, death, and DALYs of stroke tended to decrease from 1990 to 2013, the overall burden of stroke in terms of the absolute number of people affected or disabled by stroke among men and women of all ages worldwide continued to increase^29^.

### The burden of communicable and non-communicable diseases and SDI

The SDI is calculated as the geometric mean of three indices: the total fertility rate under the age of 25 (TFU25), the average education level of the population aged 15 years and older (EDU15 +) and the lagged distributive income per capita (LDI) index. Our study found that there was a significant association between the burden of CDs and NCDs and SDI. Specifically, the age-standardized incidence, death, prevalence, and DALY rates of CDs decreased with an increase in SDI. However, the relationship between these indicators of NCDs and SDI was not as clear-cut. Although the overall trend was decreasing with an increase in SDI, there were significant regional differences.

In terms of the time span, it is noteworthy noting that since 2010, the ASIR for both CD and NCDs has increased in all SDI countries, while the ASPR for NCDs has also increased, especially for high and high-middle SDI regions. Previous studies have found that the decline in age-standardized DALY rate for NCDs has accelerated over the past decade in countries with lower SDI, while improvements have started to plateau or even reverse in countries with higher SDI^30^. This is consistent with the findings of our study (Supplementary Fig. 2). In addition, Roth et al. likewise pointed out that there is a concern that the age-standardized rate burden of cardiovascular disease is starting to rise in places where it had previously declined in high-income countries^28^.

### COVID-19 and SDI

**Figure 8:**
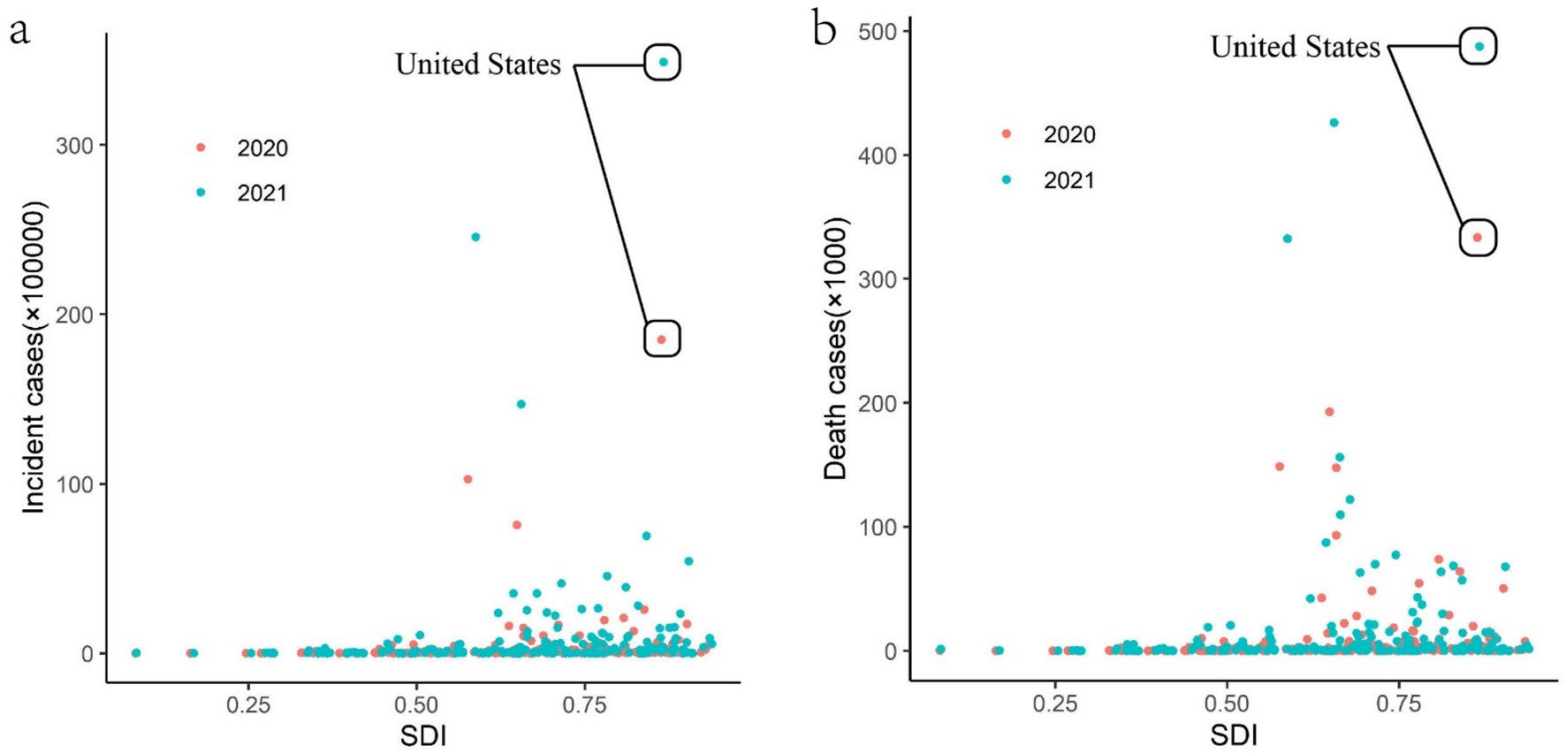
Scatter plot of the relationship between COVID-19 and the predicted SDI in 2021 and 2022. (**a**) The relationship between COVID-19 incident cases and the predicted SDI; (**b**) the relationship between COVID-19 deaths and the predicted SDI.

Due to the ongoing COVID-19 pandemic, we conducted further research to investigate whether COVID-19 aligns with the patterns of CDs observed in our previous study. We gathered data on the number of COVID-19 cases and deaths in various countries for the years 2020 and 2021 from “Our World in Data” (https://ourworldindata.org/coronavirus). As the most recent SDI data was unavailable, we used the grey prediction method to forecast the SDI data for each country in 2020 and 2021, based on the SDI data from 1990 to 2019. Figure 8 illustrates the relationship between COVID-19 burden and SDI across all countries worldwide.

The results shown in Fig. 5 indicate that unlike many other CDs, COVID-19 has a higher incidence rate and mortality rate in some countries with high SDI. In the case of the United States, it is speculated that the reason for this may be related to the continued community transmission due to insufficient COVID-19 vaccination^31^. Additionally, some experts have suggested that the ideology of medical freedom related to political extremism could have contributed to the high number of deaths caused by severe acute respiratory syndrome coronavirus-2^32^.

### Epidemiological transition

In this study, we have revealed that the global burden of NCDs is greater than that of CDs, which is consistent with the idea of epidemiological transformation proposed by some previous studies^33–35^. According to the GBD 2019 study by Stroke Collaborators, a growing portion of the global population is reaching the end of the epidemiological transition, where the risk burden has shifted toward metabolic risk factors and an increasing proportion of the disease burden is driven by stroke and other NCDs^33^. Sawyer et al. reported that the epidemiological transition to NCDs is characterized by an increasing age of disease burden across the life course^35^. Mercer, in his study of the epidemic transition model, pointed out that the major feature of the epidemiological transition is the shift of recorded causes of death from CDs to other morbid diseases^36^. Over the past 30 years, China has completed the epidemiological transition from CDs to NCDs^37^. Wong et al. also reported in their study of cirrhosis in the Asia–Pacific region that mortality due to Hepatitis B virus infection is decreasing, whereas the prevalence and incidence of fatal liver disease due to metabolic syndrome and non-alcoholic fatty liver disease continue to increase^38^. The shift in disease status from CDs to NCDs in LMICs has led to the inclusion of NCDs in the 2030 Agenda for sustainable development. However, the healthcare systems of these countries are not adequately prepared to deal with the high epidemic and economic costs of chronic diseases^39^. These aforementioned points emphasize the importance of controlling and preventing NCDs.

### Current situation and strategies

In the early days of the pandemic, a WHO assessment found that 94% of countries reported that some or all of their Ministry of Health staff working on NCDs had been reassigned to work on COVID-19. This, coupled with intermittent lockdowns and high infection rates, exacerbated the strain on the healthcare system, leading to a substantial decline in screening and treatment for NCDs. In fact, cancer registration and screening declined rapidly in many countries, even in those with low COVID-19 incidences, such as New Zealand, where cancer registration had decreased by almost 40%^40^. Sheldon et al. argued in their study that the exclusive focus on COVID-19 had obscured another, less obvious epidemic^41^. Every year, NCDs such as obesity, diabetes, heart disease, stroke, cancer, chronic respiratory diseases, and mental health disorders cause more premature deaths and suffering than COVID-19. A report from Italy showed that the majority (96.2%) of patients who died from COVID-19 had comorbidities, mainly other NCDs^42^. Chan et al. also reported that in developed countries, the pressure of managing the acute effect of COVID-19 on health systems had increased the existing burden of chronic NCDs or long-term conditions^43^. During the same time, Natalie Dean, a biostatistician at Emory University in Atlanta, Georgia, warned that the Omicron variant was spreading far beyond the detection capacity of many countries, the novel coronavirus virus could continue to spread for a long time in the future, and the threat of new variants is imminent^44^. These findings are consistent with the results of Yadav et al., who suggested that COVID-19 and NCDs bidirectional relationship, whereby NCDs increase vulnerability to COVID-19, and COVID-19 increases the risk factors associated with NCDs^45^. Therefore, developing a reasonable and sound healthcare system, as well as adjusting policies, is crucial.

In this study, diarrhea, a communicable disease, was found to have the highest burden. A meta-analysis conducted by Wolf et al. suggested that interventions such as high-quality drinking water and filtered water at point-of-use could reduce the risk of diarrhea by approximately 50%^21^. In addition, Nandi et al. suggested that clean tap water and improved sanitation could effectively reduce the burden of mortality and morbidity due to diarrhea in children in India. Therefore, expanding access to tap water and improving sanitation could significantly and equitably reduce the burden of diarrhea in children in India^46^. Troeger et al. also highlighted the importance of prioritizing the introduction of vaccines and interventions to decrease diarrhea-related morbidity and mortality^47^.

It is crucial not to neglect the issue of NCDs, even amidst the pandemic. Building resilient healthcare systems that can treat NCDs during and after the pandemic, especially by expanding diagnostic capacity, is essential^40^. To decrease NCDs-related mortality, primary prevention should address underlying causes, and secondary prevention should ensure early detection and effective management. Detection and tracing of COVID-19 cases is similar to that of other NCDs, where concerted action is required to detect and manage cases early^41^. Monitoring social changes and inequalities exacerbated by COVID-19 is crucial for predicting downstream consequences of NCDs^48^. Nikoloski et al. reported that patients with chronic diseases are advised to continue their existing treatment for their conditions, as they are more likely to be affected by COVID-19^49^. Abolfazl Avan et al. proposed that, to reduce inequalities in stroke care, social and economic policies should be a health priority, especially in less affluent countries^50^. Telemedicine and virtual visits should receive more attention. Takahashi et al. also reported that telehealth can provide health services remotely through telecommunication technology and has had a significant effect on the evolving medical landscape^51^. Recent research has revealed the importance of an active lifestyle and regular exercise, as physical inactivity is a focal modifiable risk factor for many NCDs and mental health conditions^52^.

## Conclusions

The COVID-19 pandemic has created unprecedented challenges to the global healthcare system, especially in managing NCDs. It is essential to develop resilient healthcare systems capable of treating NCDs, expanding diagnostic capacity, and prioritizing the introduction of vaccines and interventions to reduce disease-related morbidity and mortality. Additionally, addressing underlying causes and promoting primary prevention of NCDs, as well as monitoring social changes and inequalities exacerbated by COVID-19, are also critical steps towards improving global health outcomes.

### Supplementary Information

Supplementary Information.

## Data Availability

The datasets generated and analysed during the current study are available in the Global Burden of Disease Study 2019 repository, https://ghdx.healthdata.org/gbd-2019.
