# Supplementary material for: Prioritizing non-communicable diseases in the post-pandemic era based on a comprehensive analysis of the GBD 2019 from 1990 to 2019
Source: Sci Rep. 2023 Aug 16;13:13325. doi: 10.1038/s41598-023-40595-7 (PMC10432467; doi:10.1038/s41598-023-40595-7)
Supplement: Supplementary file 1 — Supplementary Information. [file 41598_2023_40595_MOESM1_ESM.docx]

**Contents**

[Figures 2](#_Toc130391553)

[Figure 1 Total burden of communicable and non-communicable diseases for men and women in different age groups from 1990 to 2019. 3](#_Toc130391554)

[Figure 2 Trends in the burden of communicable and non-communicable diseases at five SDI quintiles, 1990-2019. 4](#_Toc130391555)

[Figure 3 Global and regional communicable diseases ranked by total number of new cases in 2019. 5](#_Toc130391556)

[Figure 4 Global and regional communicable diseases ranked by total number of people affected in 2019. 6](#_Toc130391557)

[Figure 5 Global and regional communicable diseases ranked by DALYs in 2019. 7](#_Toc130391558)

[Figure 6 Global and regional non-communicable diseases ranked by total number of new cases in 2019. 8](#_Toc130391559)

[Figure 7 Global and regional non-communicable diseases ranked by total number of people affected in 2019. 9](#_Toc130391560)

[Figure 8 Global and regional non-communicable diseases ranked by DALYs in 2019. 10](#_Toc130391561)

[Figure 9 Proportions of communicable disease burden by age in 2019. 11](#_Toc130391562)

[Figure 10 Proportions of non-communicable disease burden by age in 2019. 11](#_Toc130391563)

[Figure 11 Percentage change in age-standardized rate (per 100,000 population) of different communicable diseases, 1990-2019. 12](#_Toc130391564)

[Figure 12 Percentage change in age-standardized rate (per 100,000 population) of different non-communicable diseases, 1990-2019. 12](#_Toc130391565)

[Tables 13](#_Toc130391566)

[Table 1 Global burden of communicable diseases in 2019. 13](#_Toc130391567)

[Table 2 Global burden of non-communicable diseases in 2019. 14](#_Toc130391568)

[Table 3 Regional burden of communicable diseases in 2019. 15](#_Toc130391569)

[Table 4 Regional burden of non-communicable diseases in 2019. 20](#_Toc130391570)

[Table 5 The global burden of different communicable diseases in 2019. 25](#_Toc130391571)

[Table 6 The global burden of different non-communicable diseases in 2019. 28](#_Toc130391572)

[Table 7 Burden of communicable diseases in 204 countries and regions in 2019. 31](#_Toc130391573)

[Table 8 Burden of non-communicable diseases in 204 countries and regions in 2019. 46](#_Toc130391574)

## Figures

| a.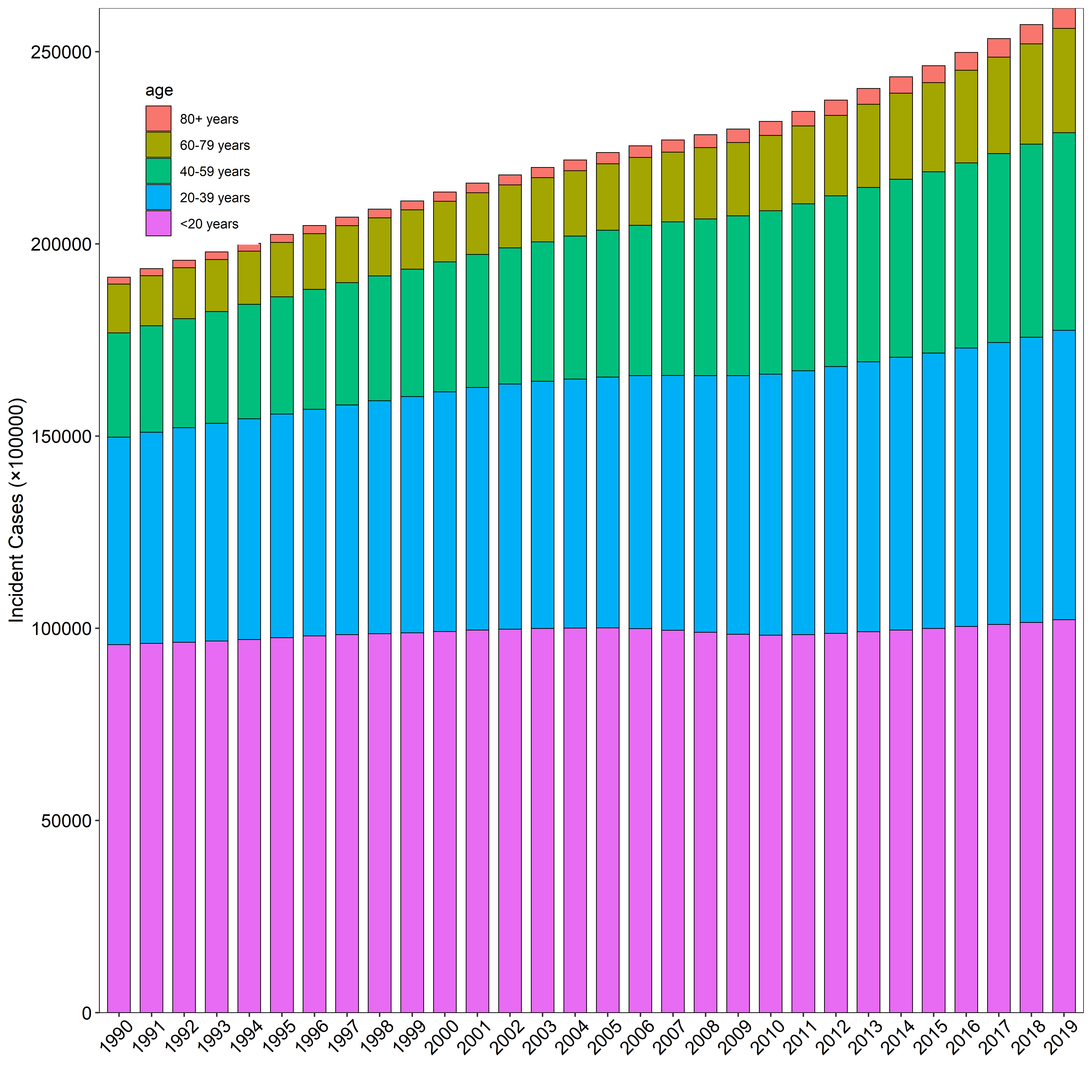 | b.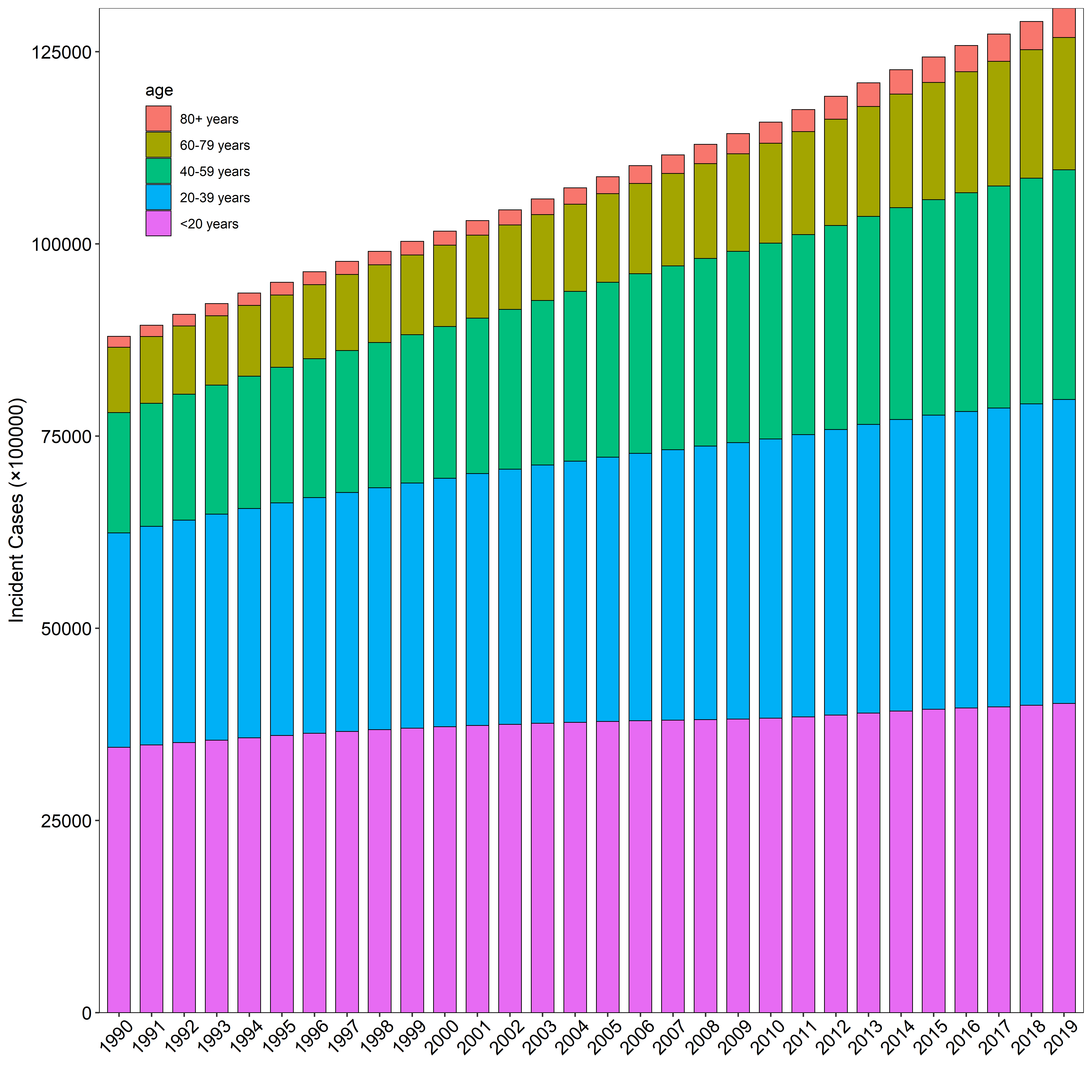 |
| --- | --- |
| c.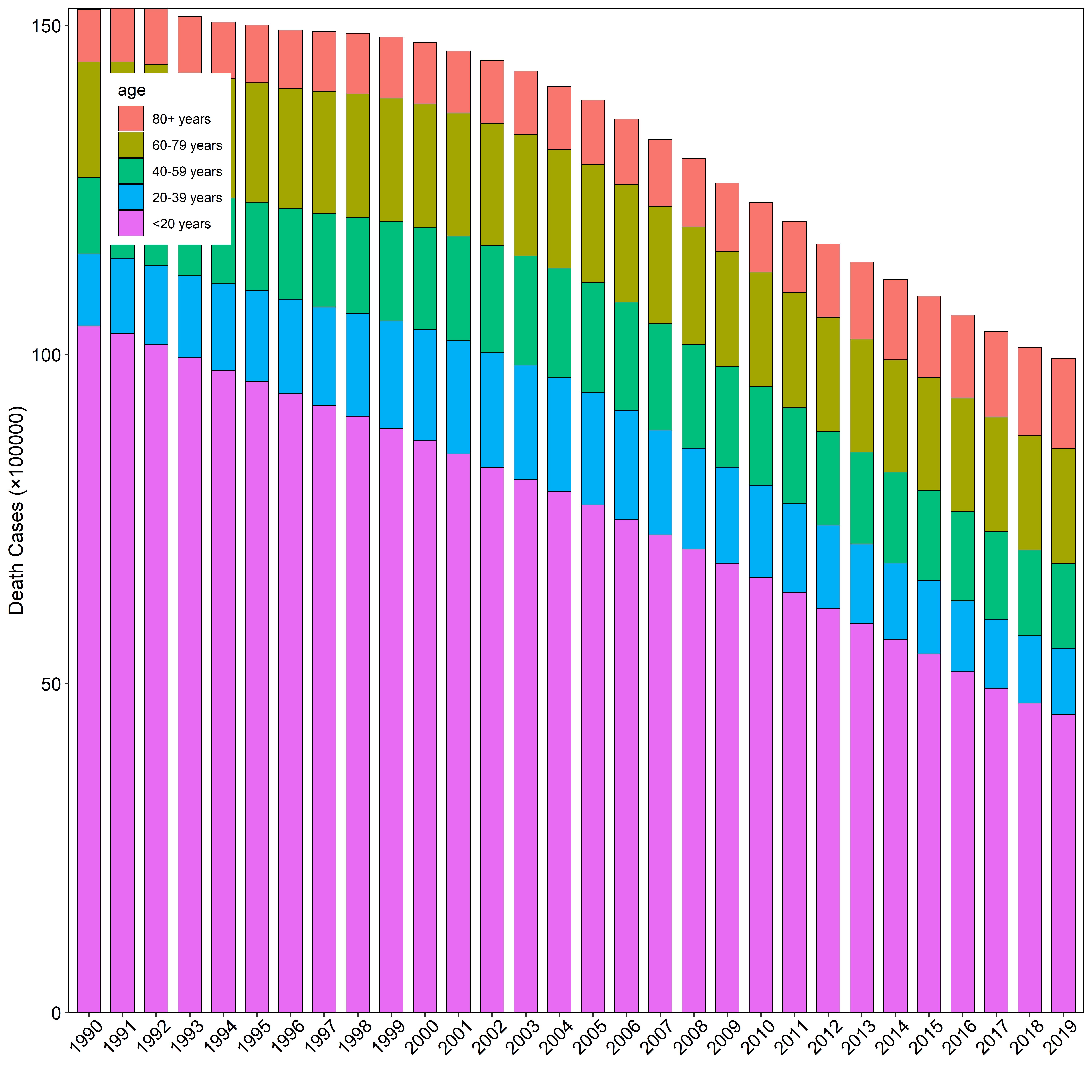 | d.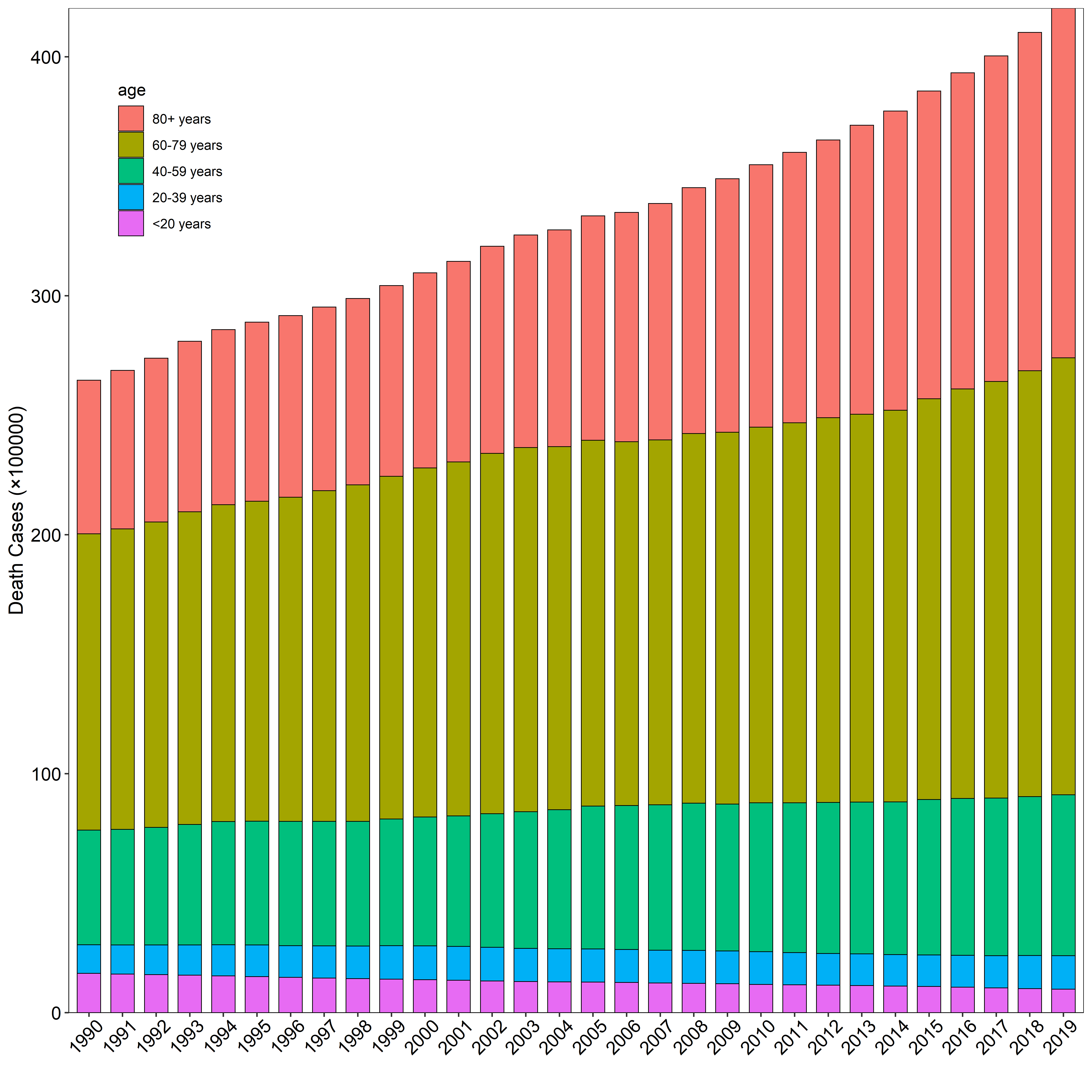 |
| e.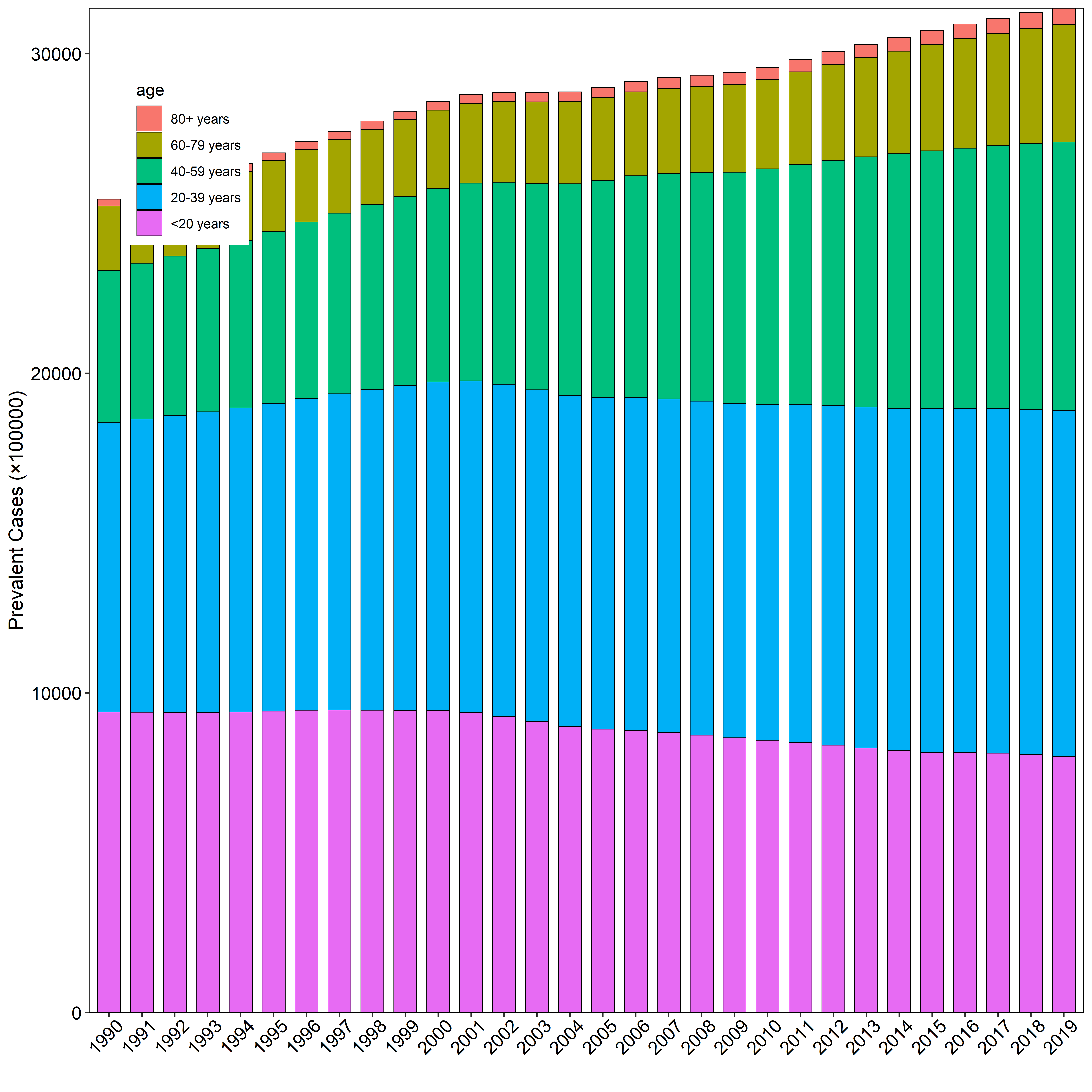 | f.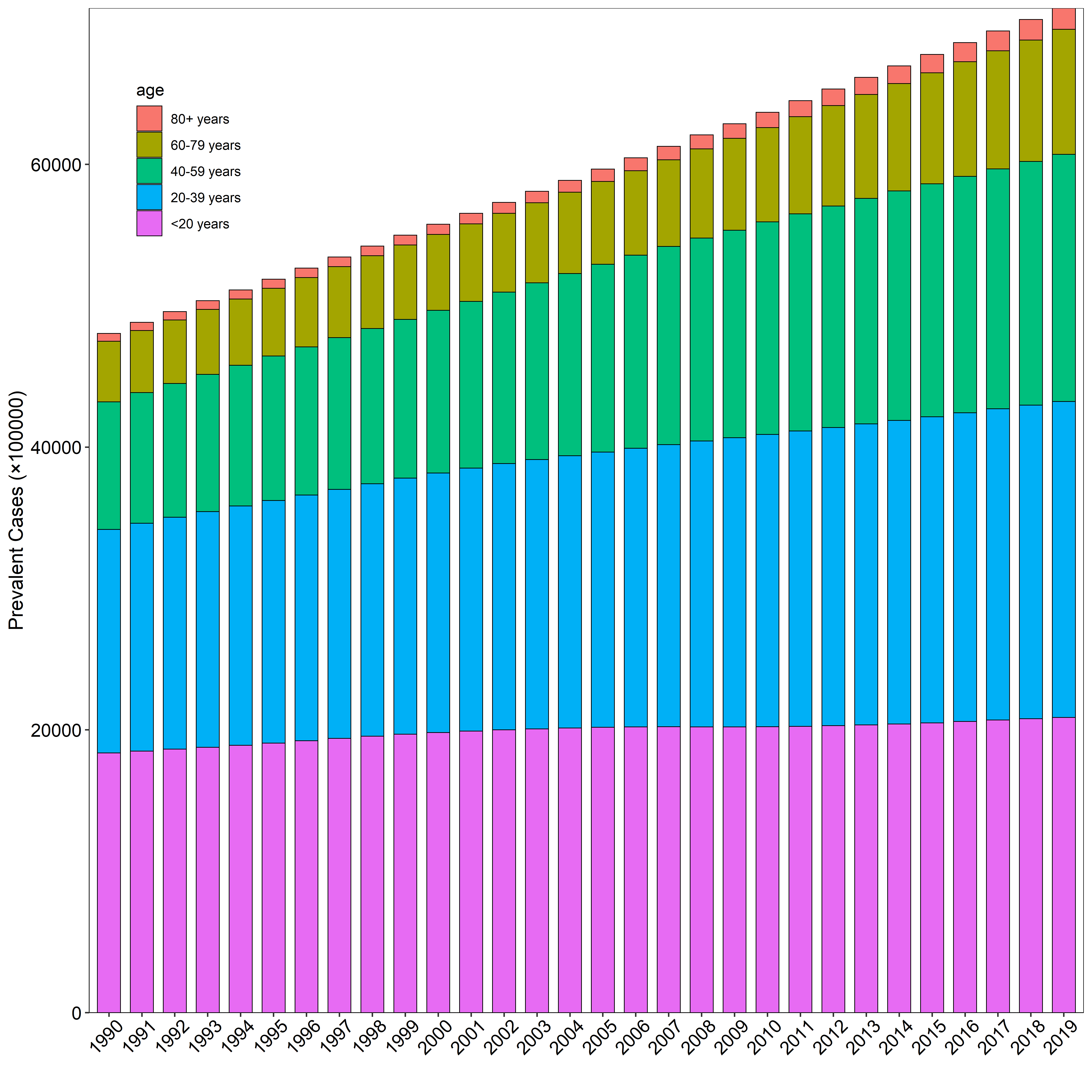 |
| g.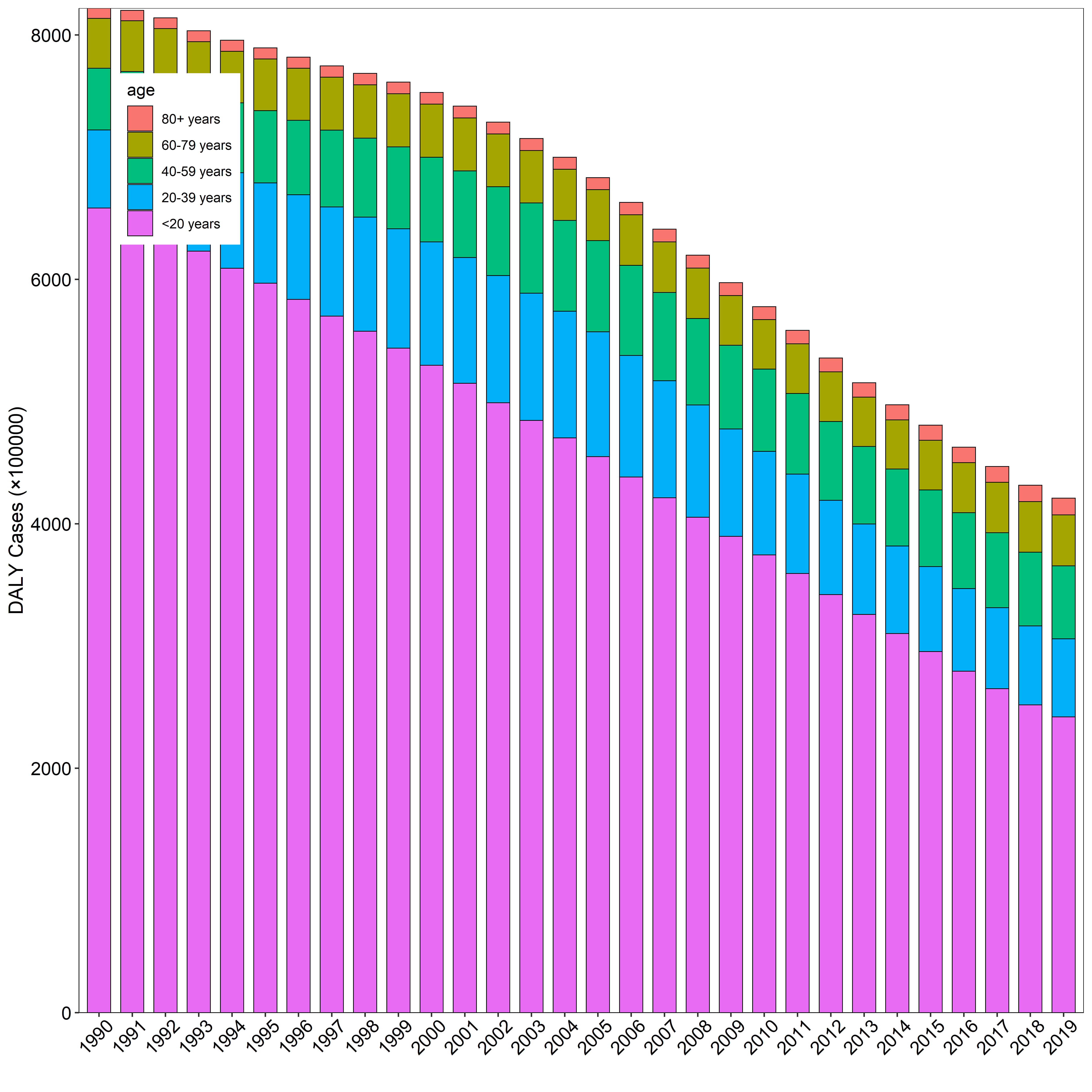 | h.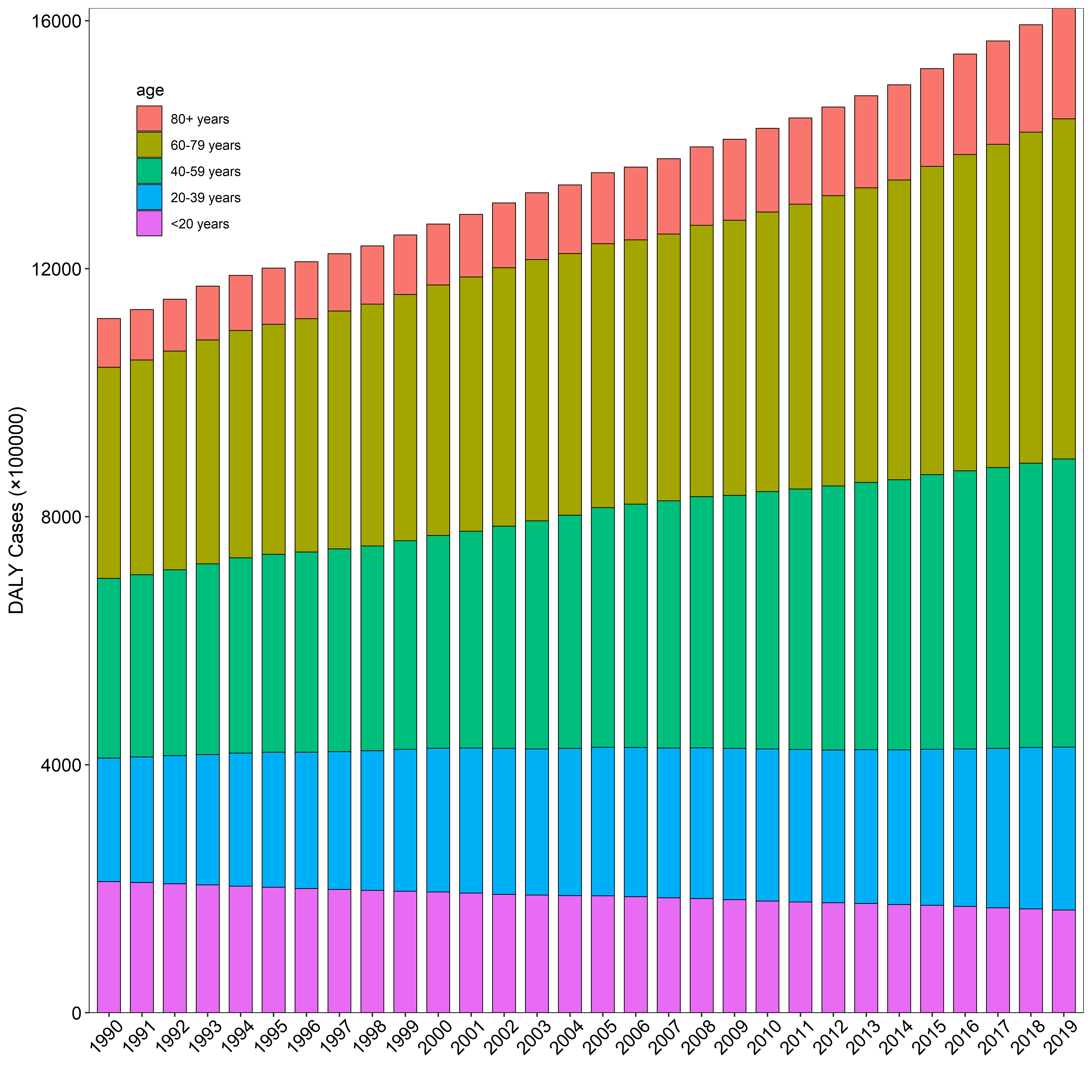 |

Figure 1 Total burden of communicable and non-communicable diseases for men and women in different age groups from 1990 to 2019.

(a) Incidence of communicable diseases; (b) Incidence of non-communicable diseases; (c) Deaths from communicable diseases; (d) Number of deaths from non-communicable diseases; (e) The number of communicable diseases; (f) Number of cases of non-communicable diseases; (g) DALYs of communicable diseases; (h) DALYs of non-communicable diseases. Abbreviation: DALYs disability-adjusted life years.

| a.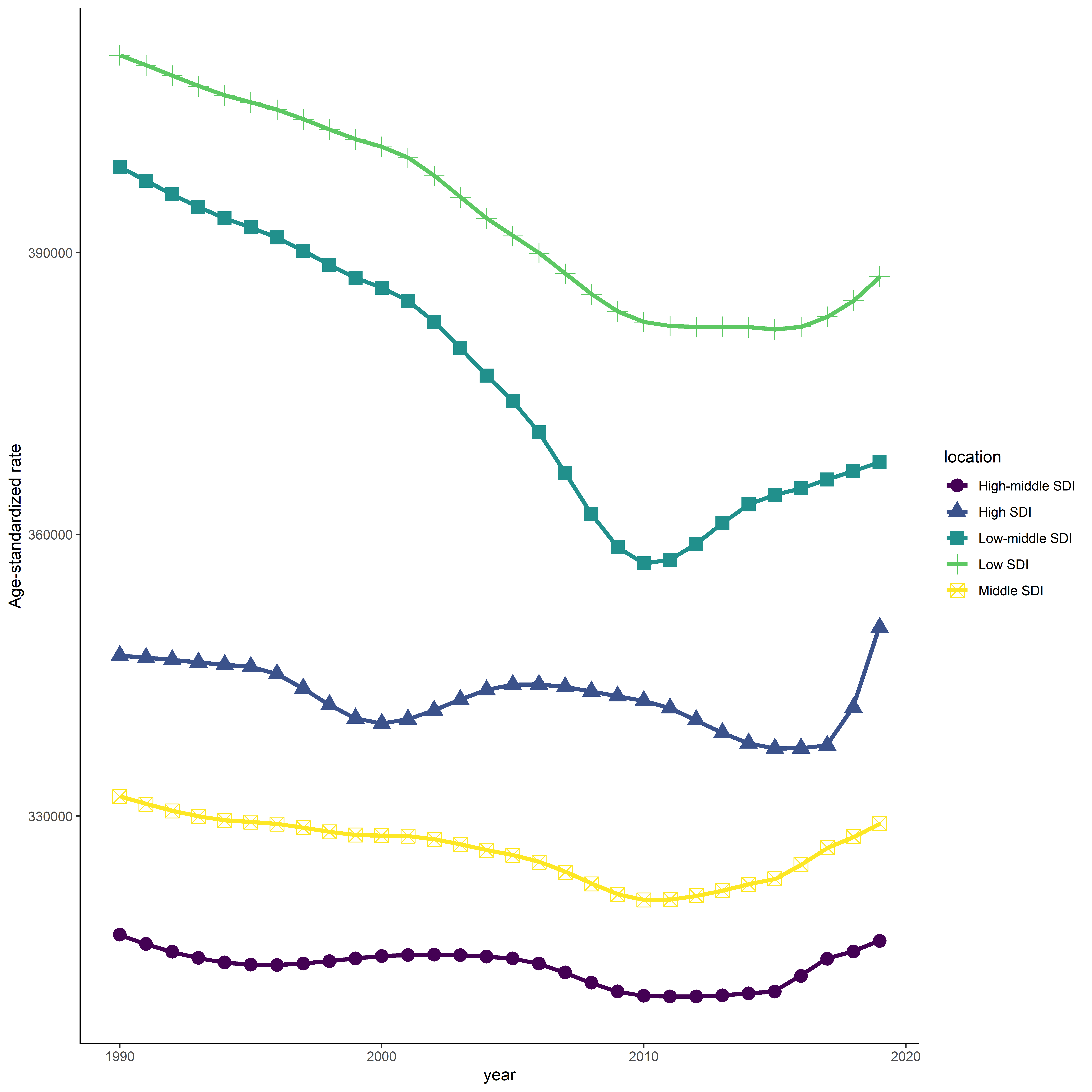 | b.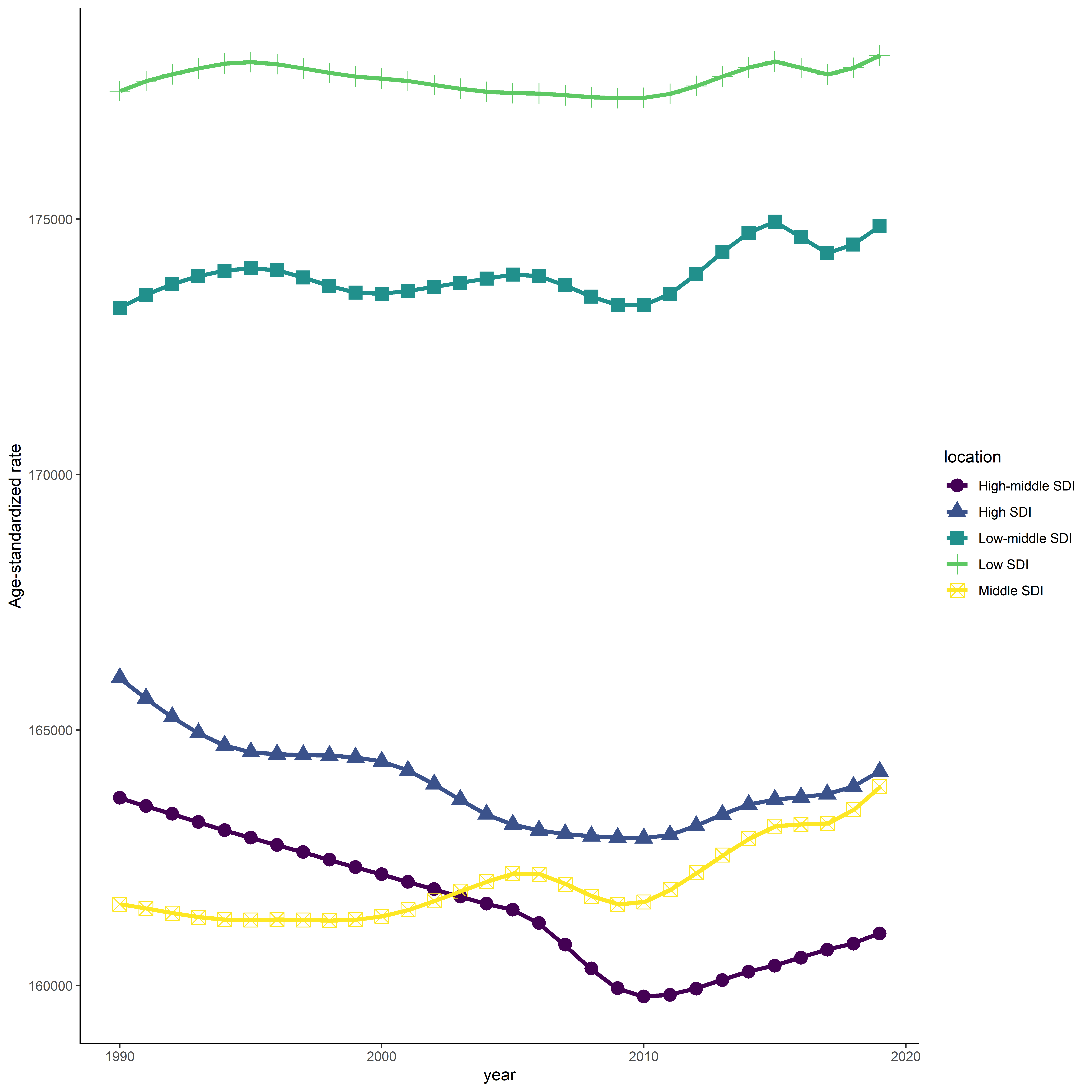 |
| --- | --- |
| c.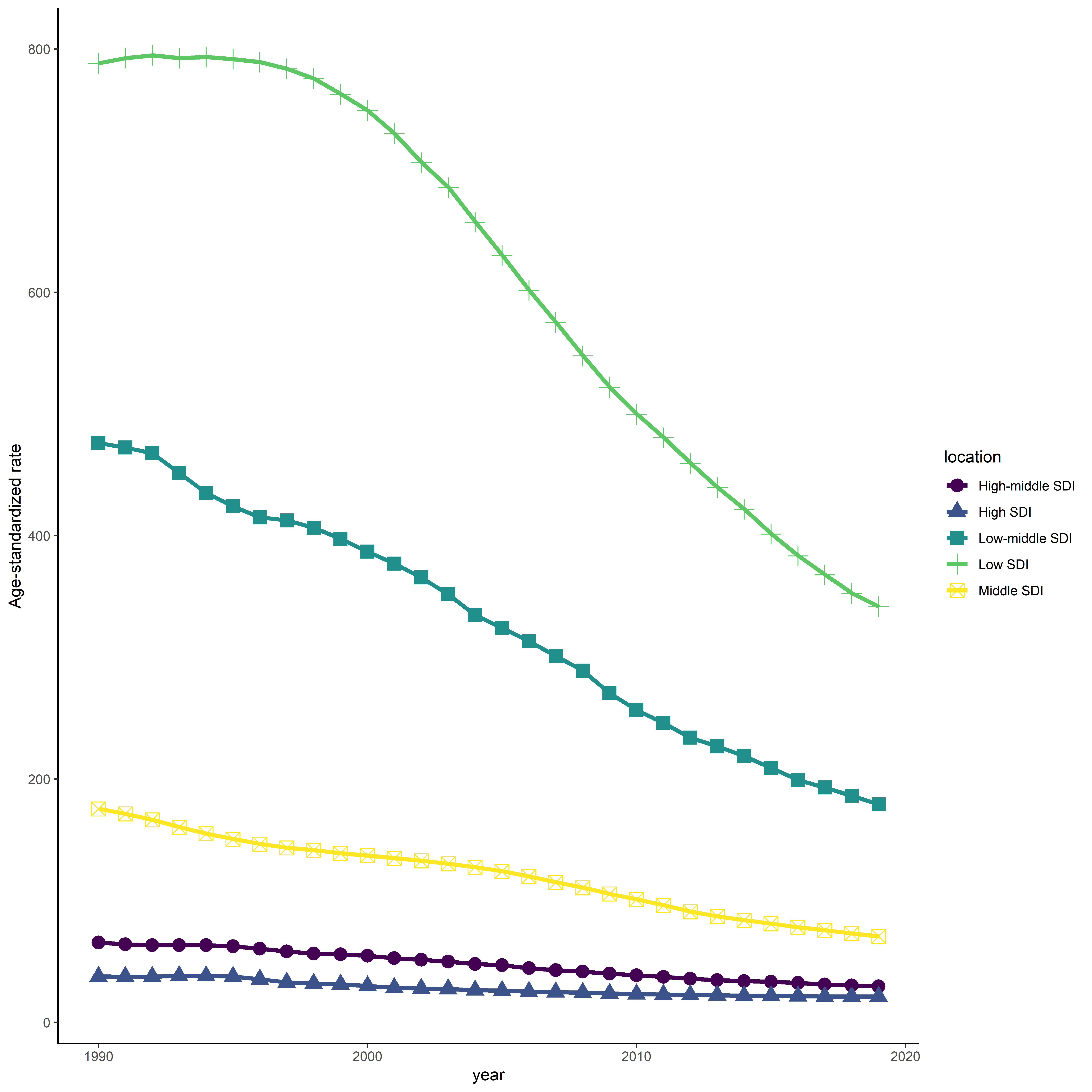 | d.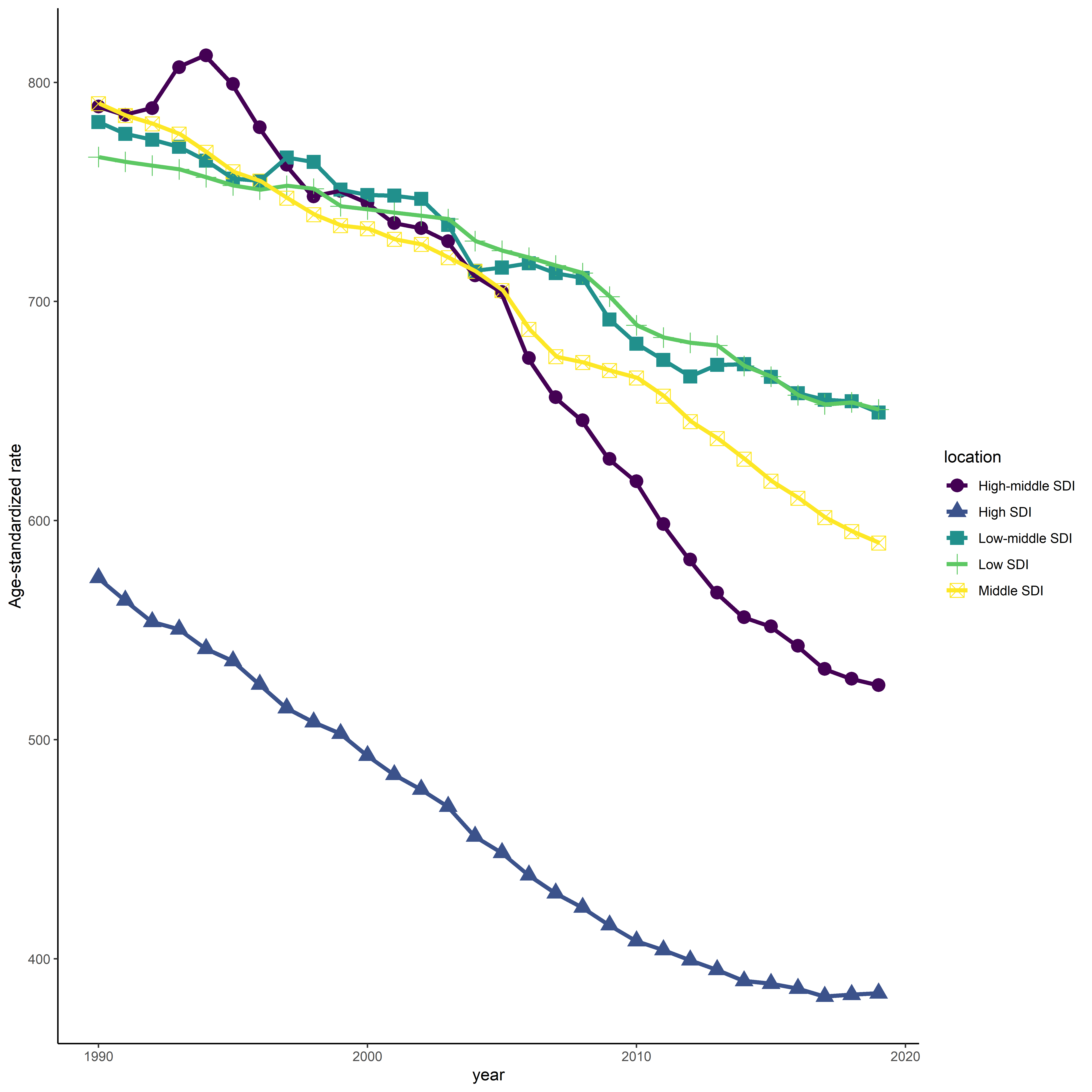 |
| e.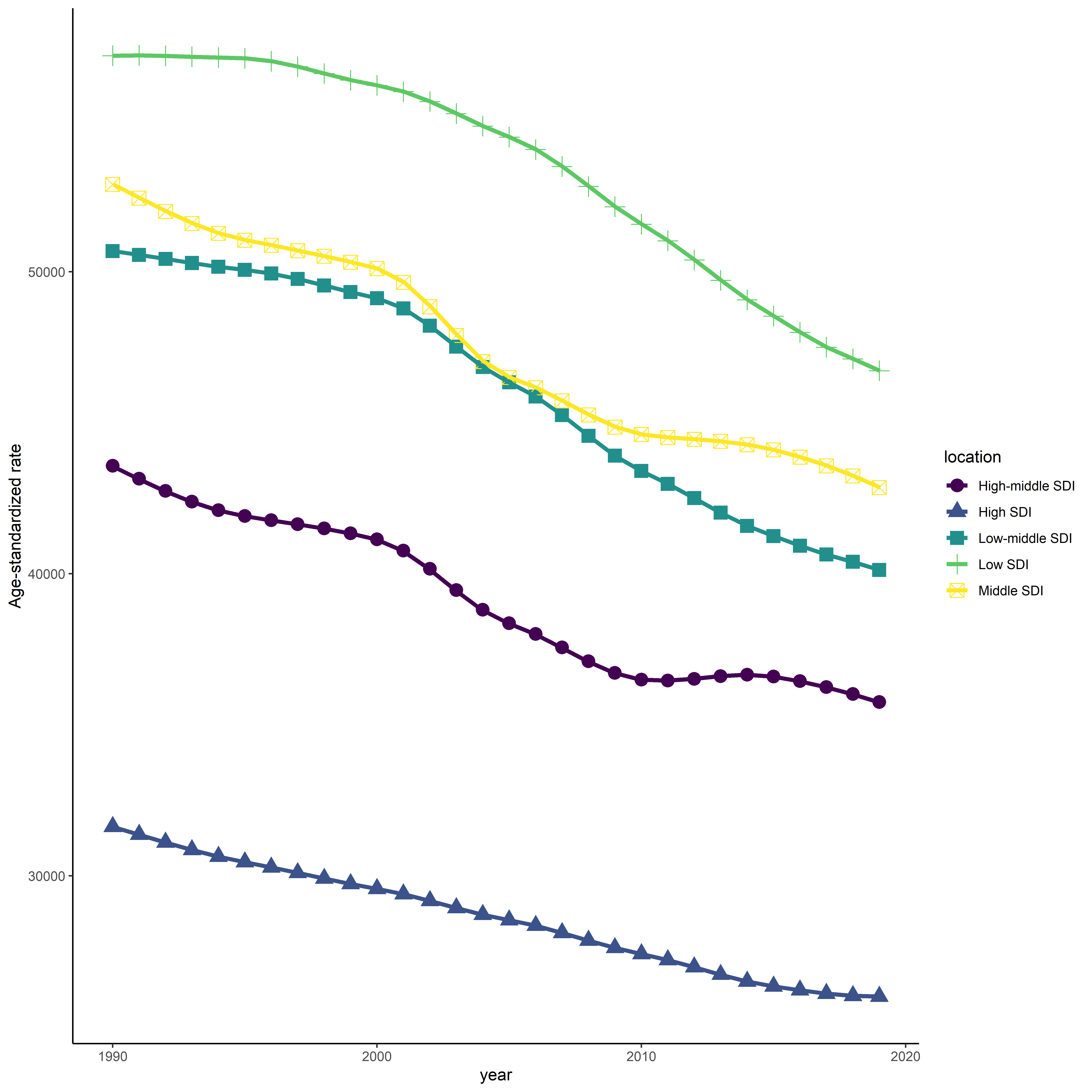 | f.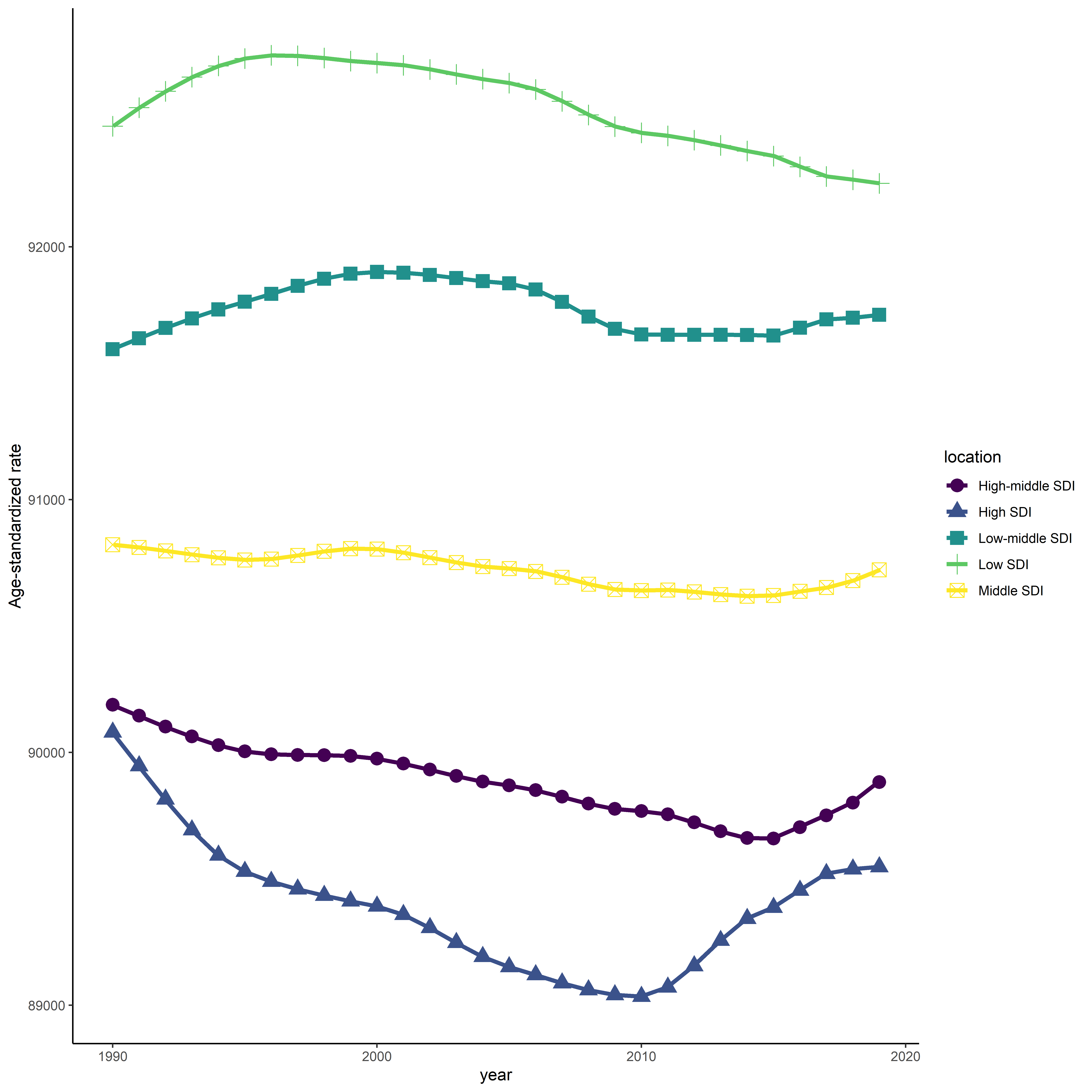 |
| g.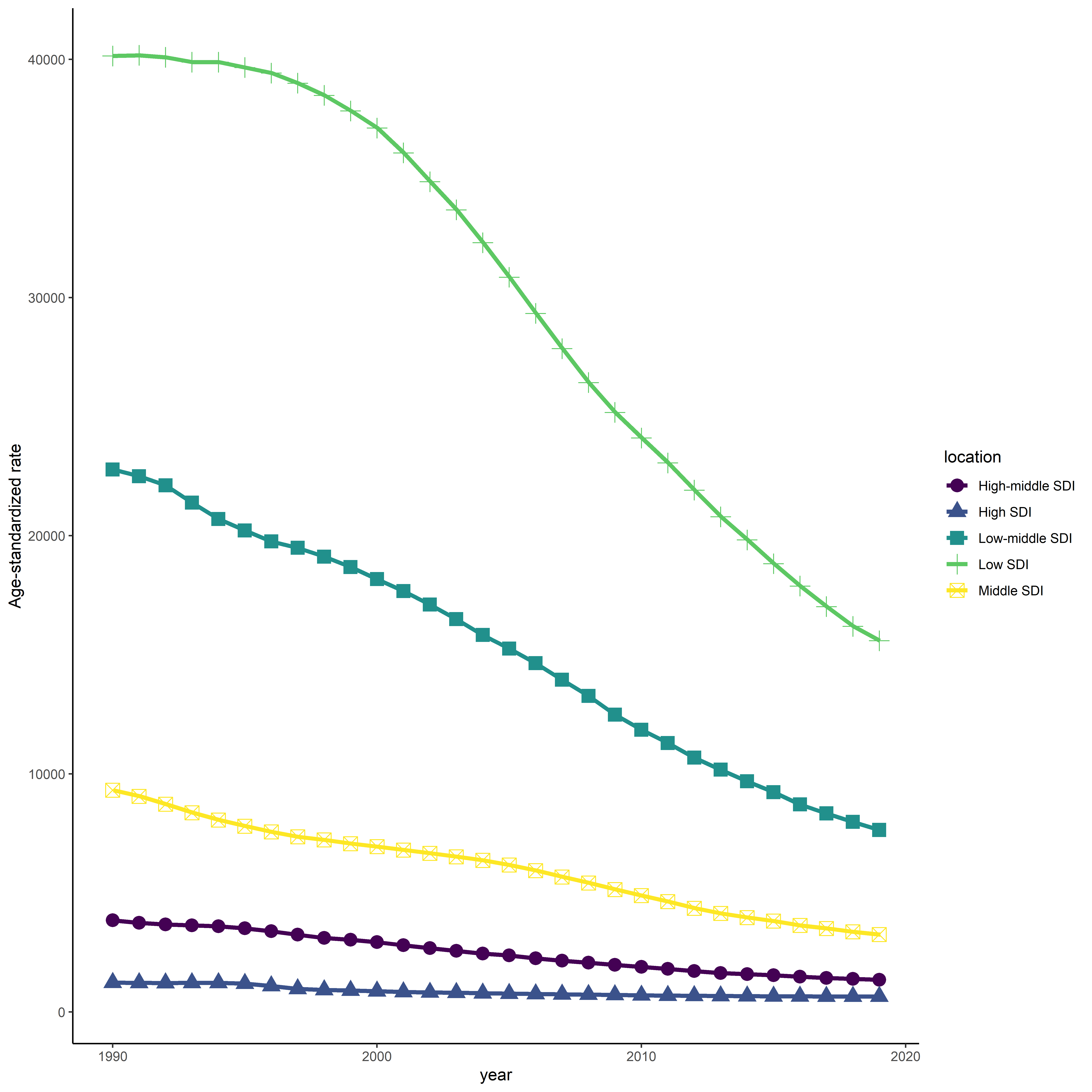 | h.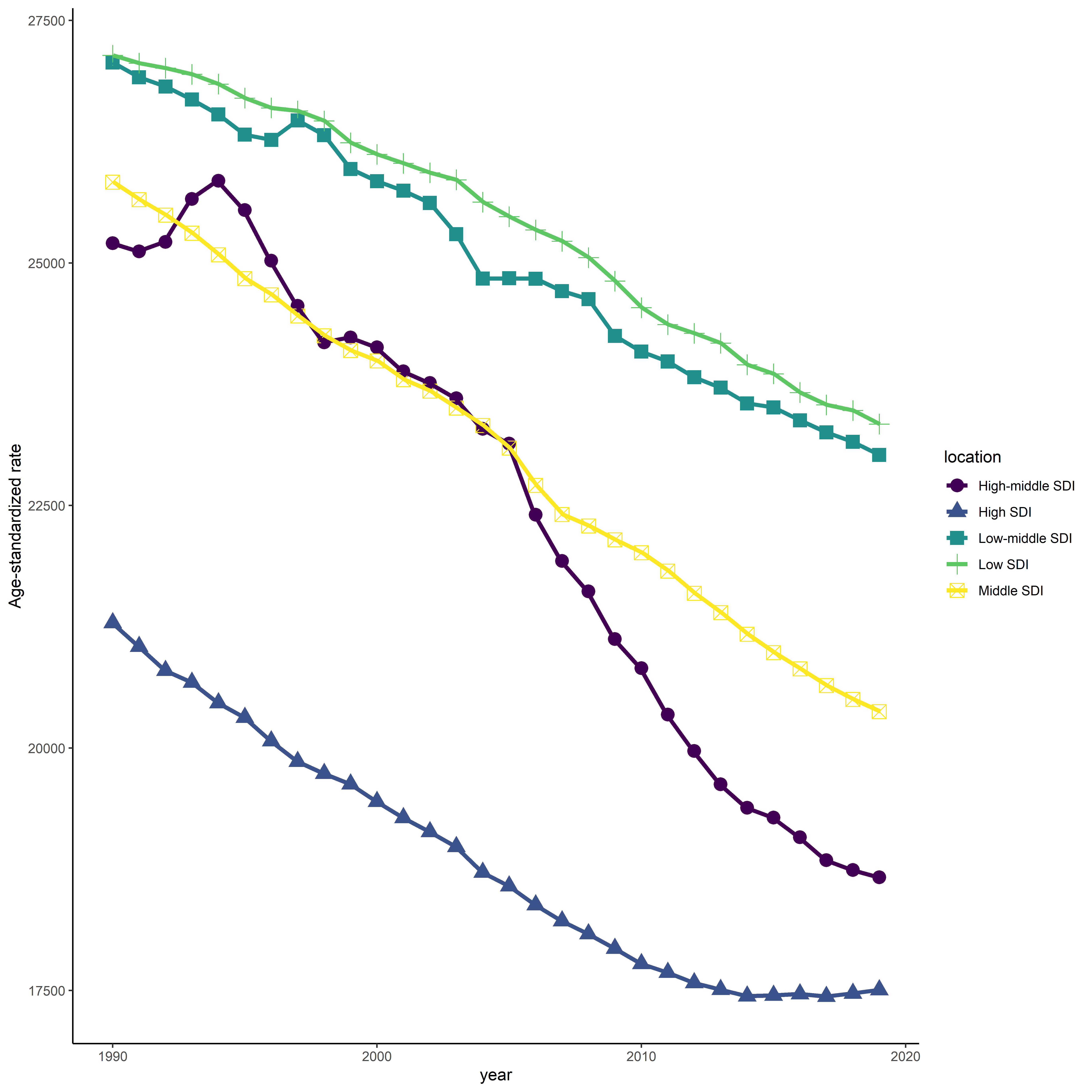 |

Figure 2 Trends in the burden of communicable and non-communicable diseases at five SDI quintiles, 1990-2019.

(a) Incidence of communicable diseases; (b) Incidence of non-communicable diseases; (c) Deaths of communicable diseases; (d) Deaths of non-communicable diseases; (e) Prevalence of communicable diseases; (f) Prevalence of non-communicable diseases; (g) DALY of communicable diseases; (h) DALY of non-communicable diseases. Abbreviation: DAL Ys disability Adjusted life years.


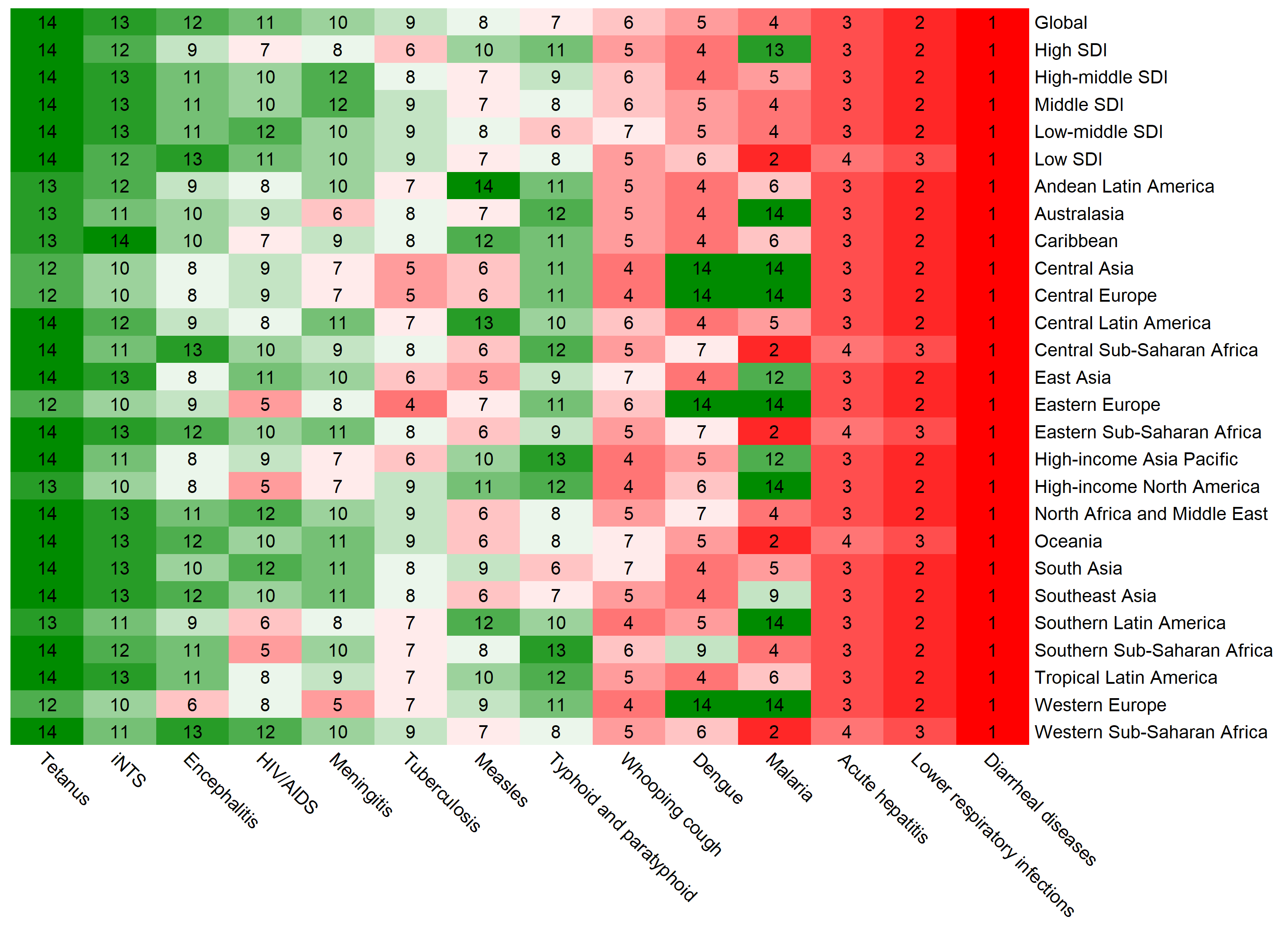


Figure 3 Global and regional communicable diseases ranked by total number of new cases in 2019.


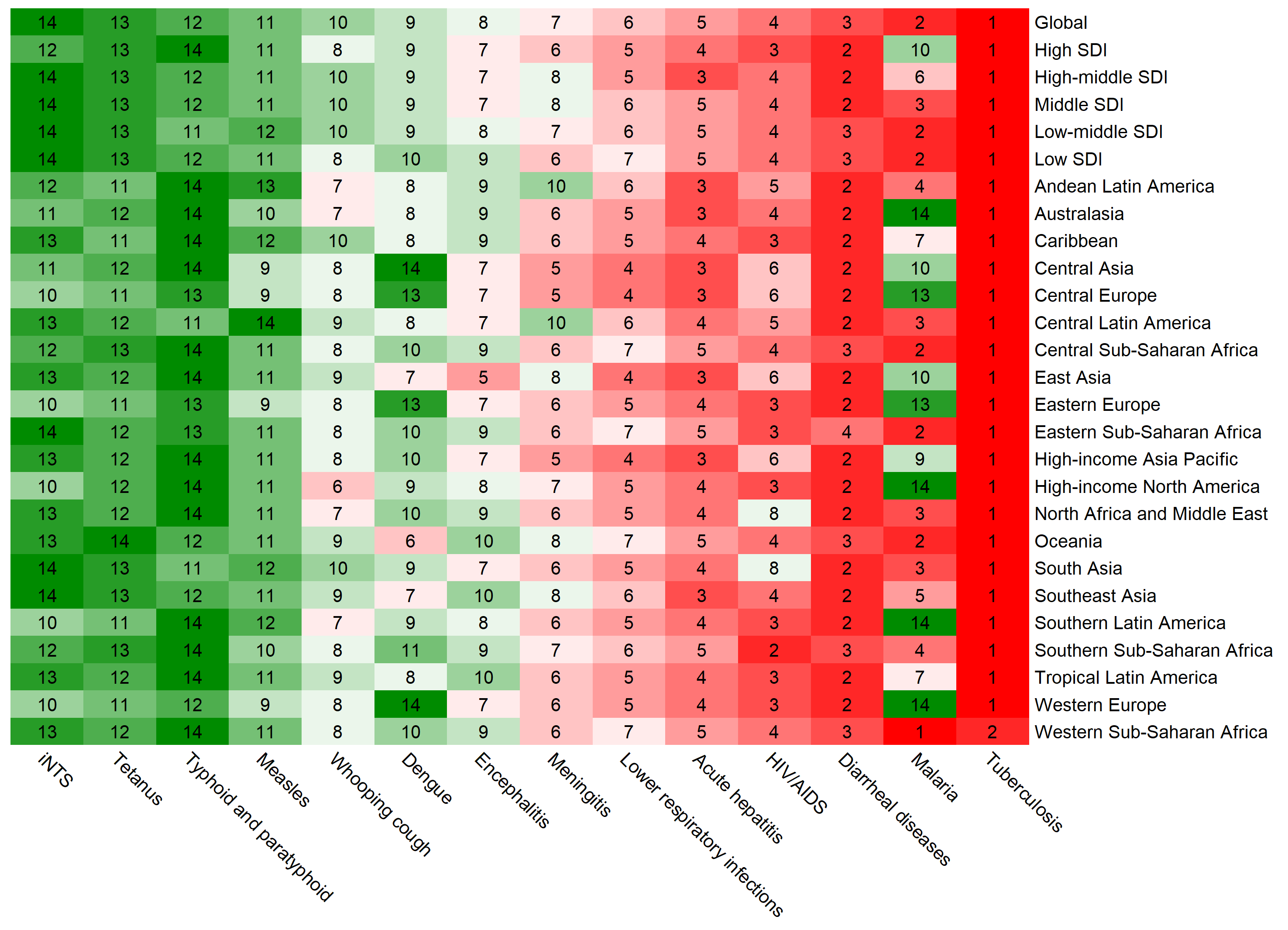


Figure 4 Global and regional communicable diseases ranked by total number of people affected in 2019.


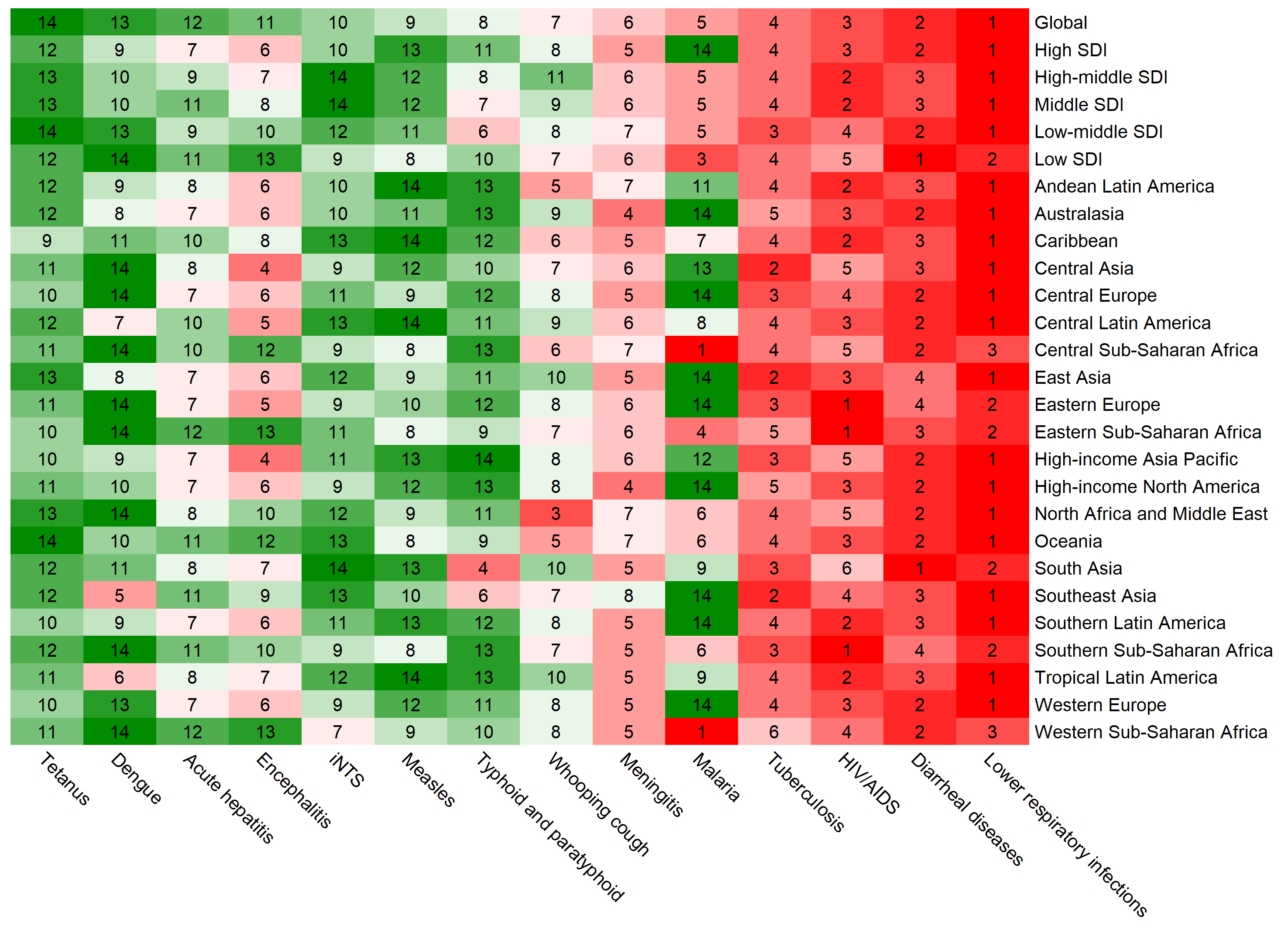


Figure 5 Global and regional communicable diseases ranked by DALYs in 2019.


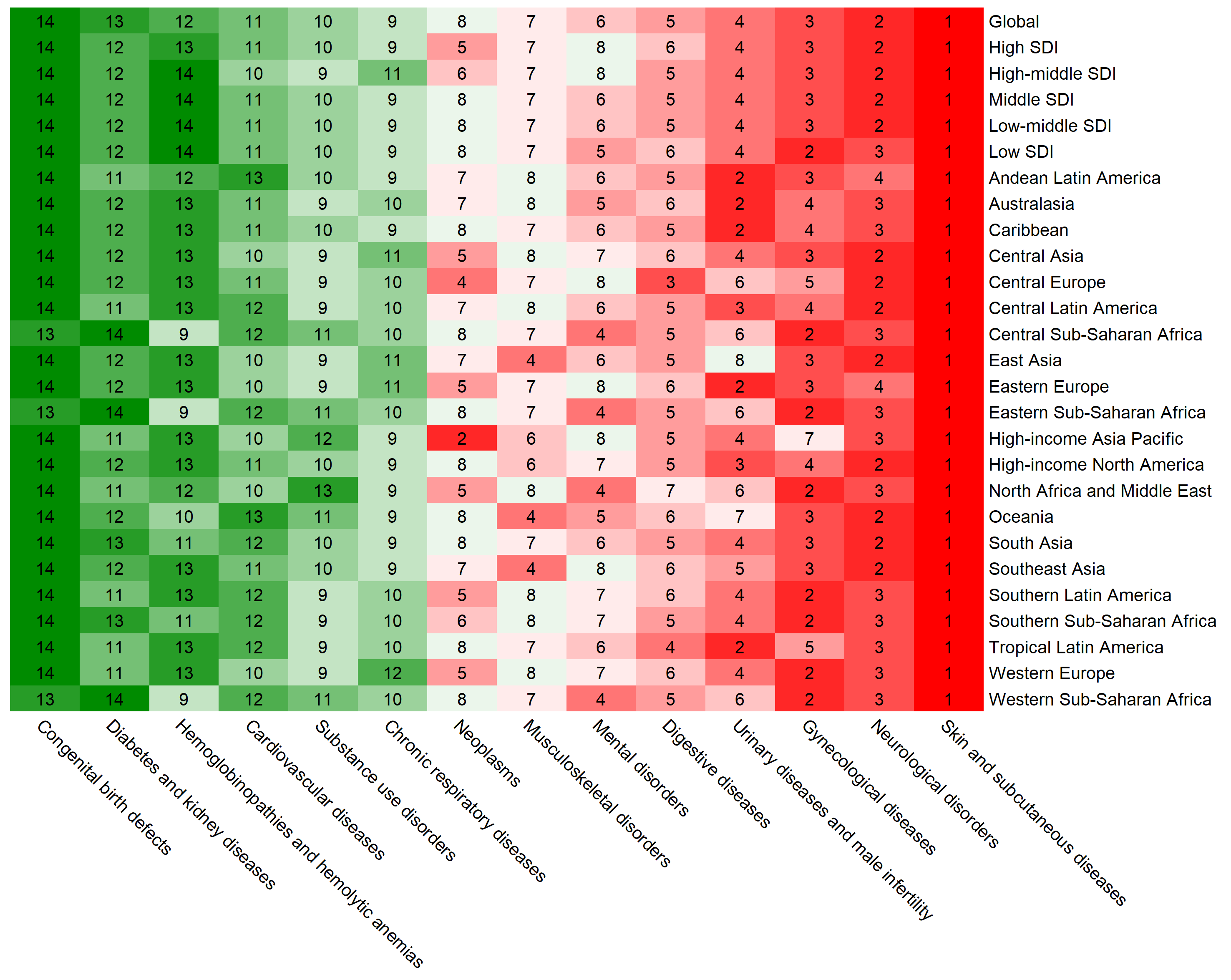


Figure 6 Global and regional non-communicable diseases ranked by total number of new cases in 2019.


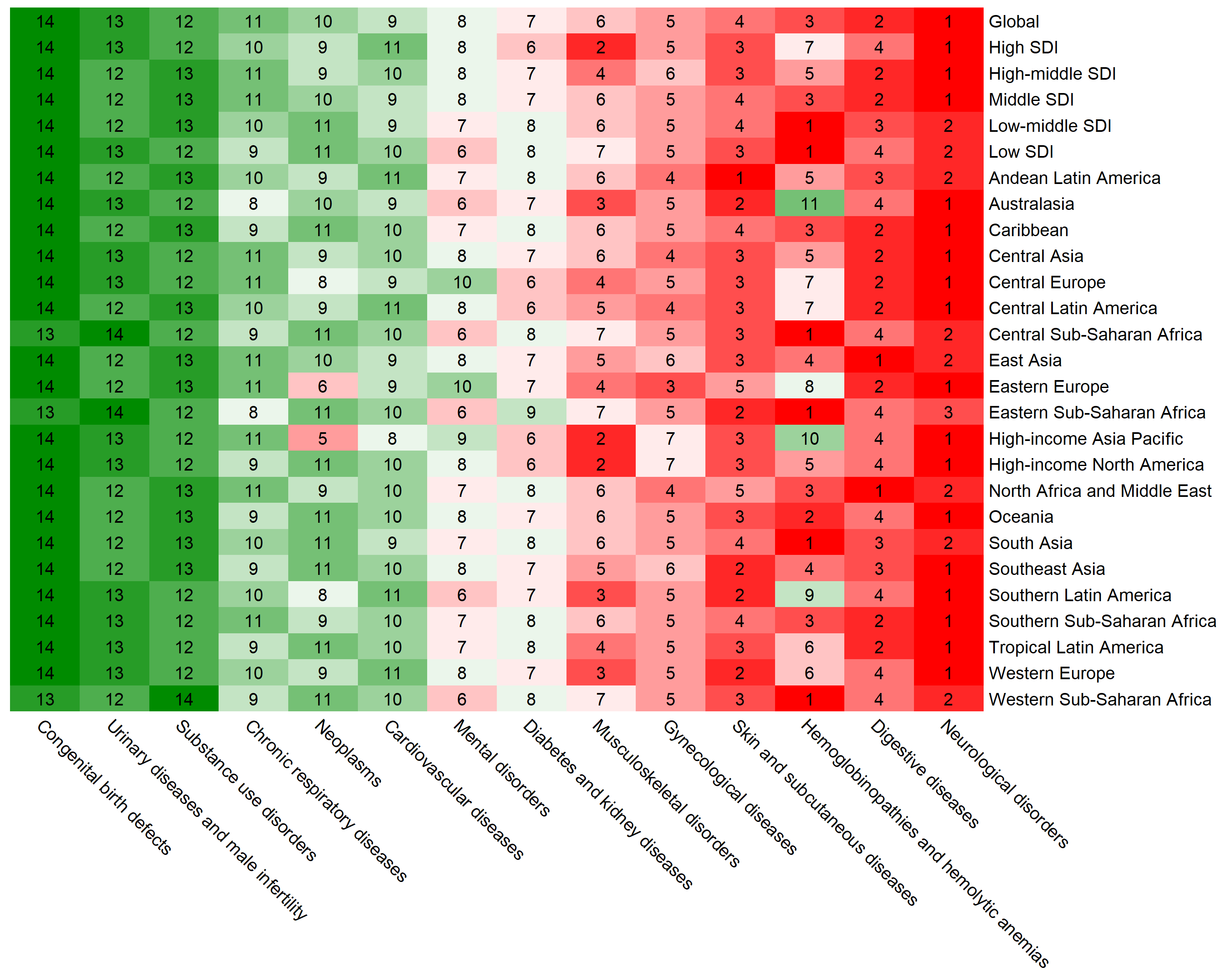


Figure 7 Global and regional non-communicable diseases ranked by total number of people affected in 2019.


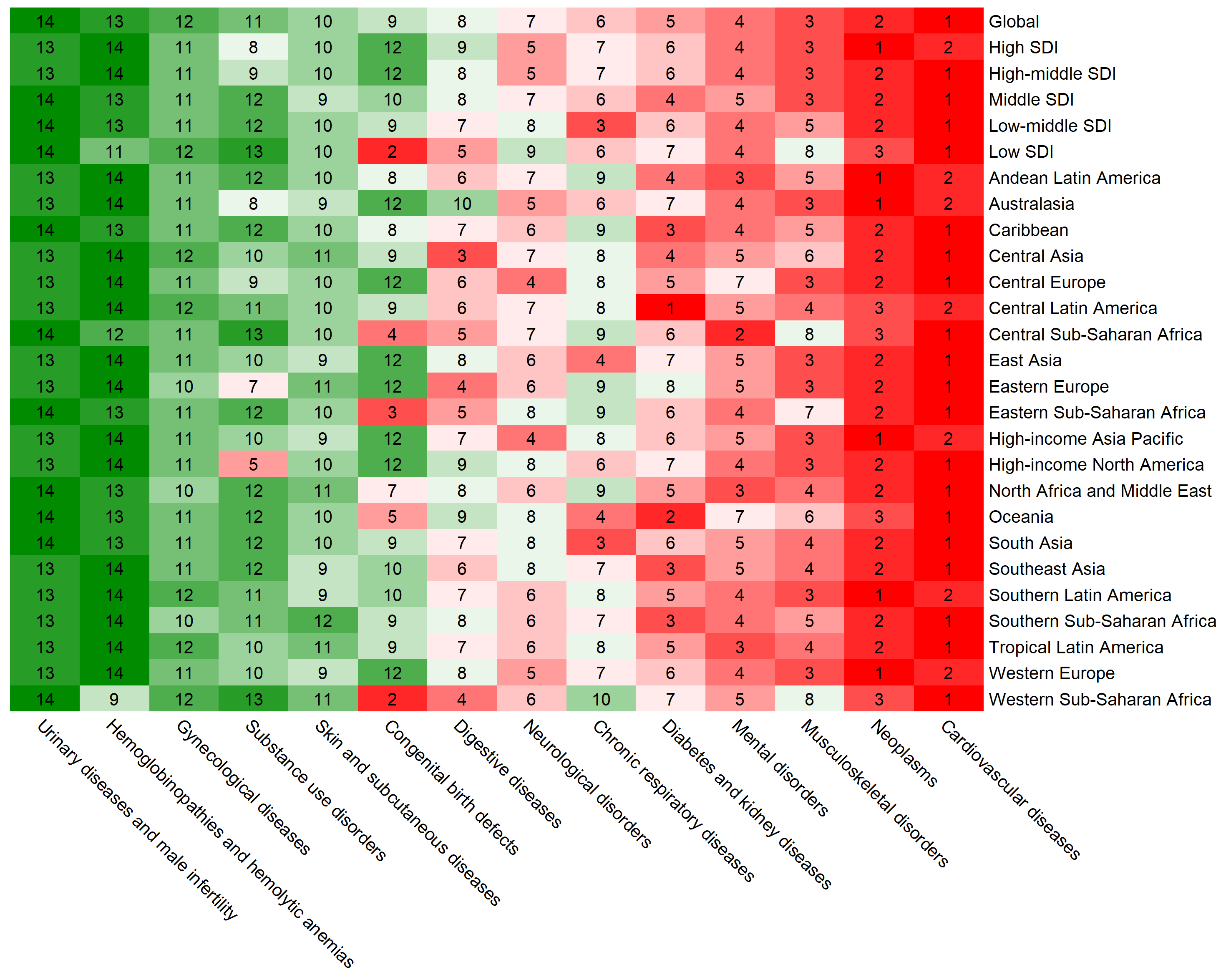


Figure 8 Global and regional non-communicable diseases ranked by DALYs in 2019.

| a.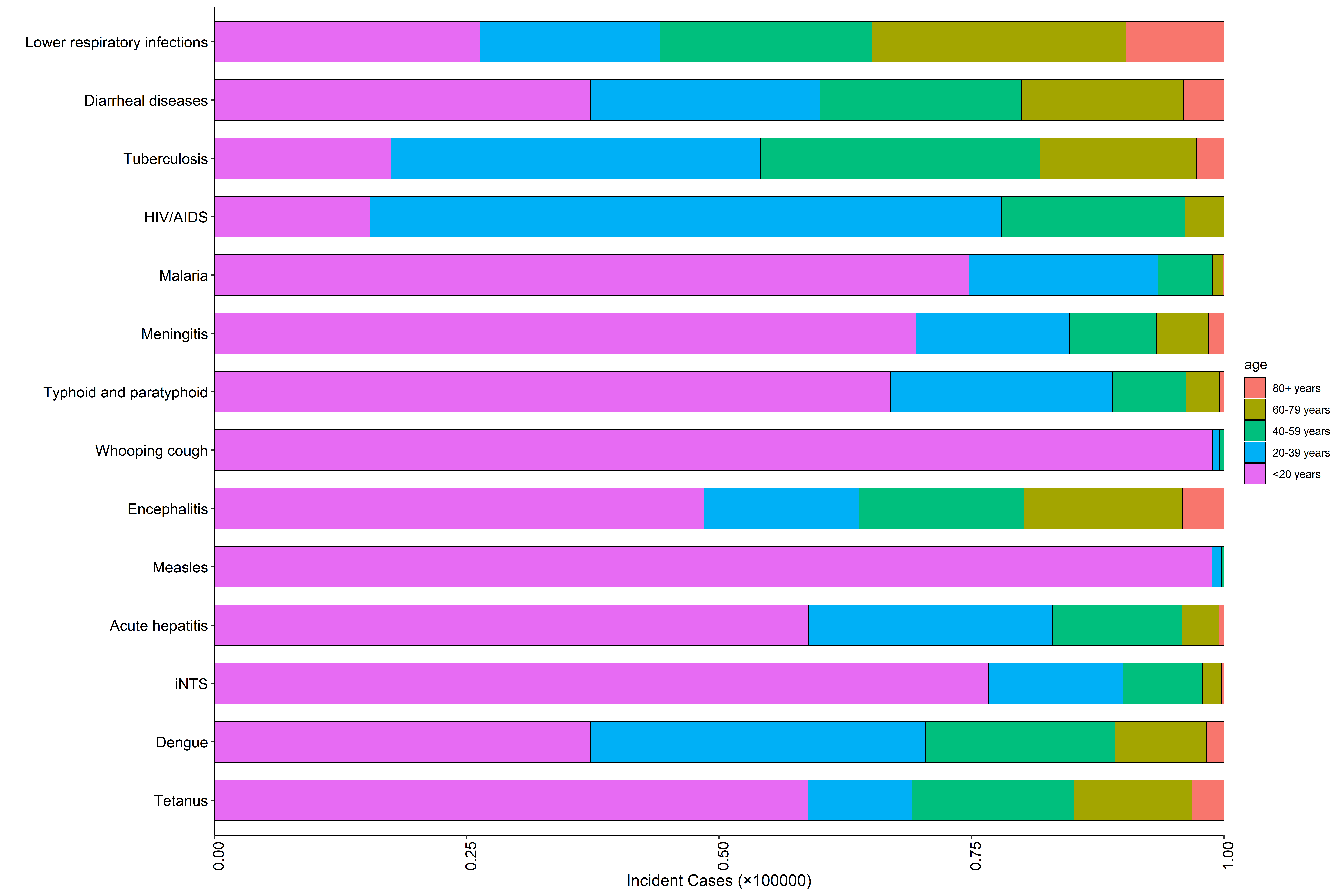 | b.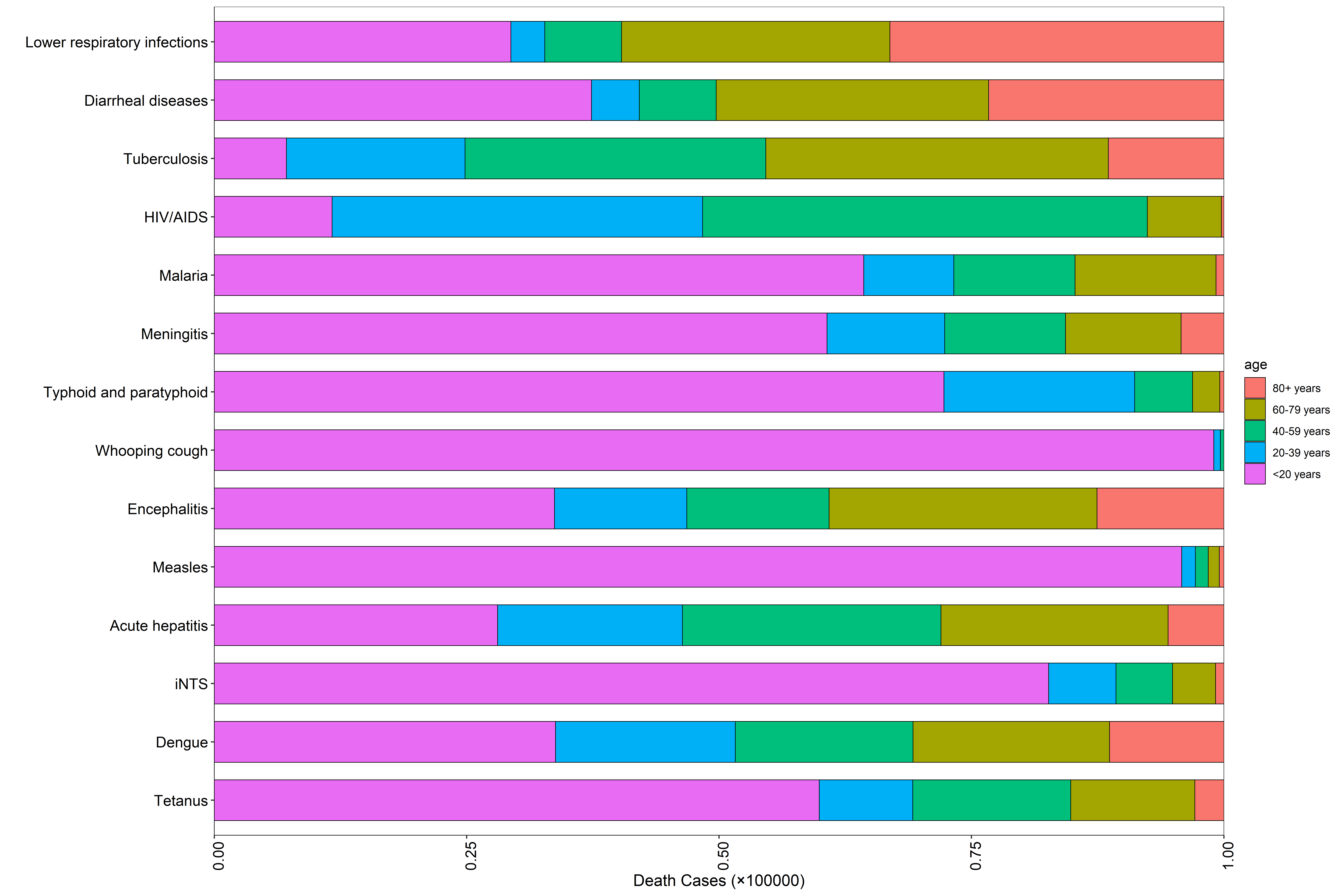 |
| --- | --- |
| c.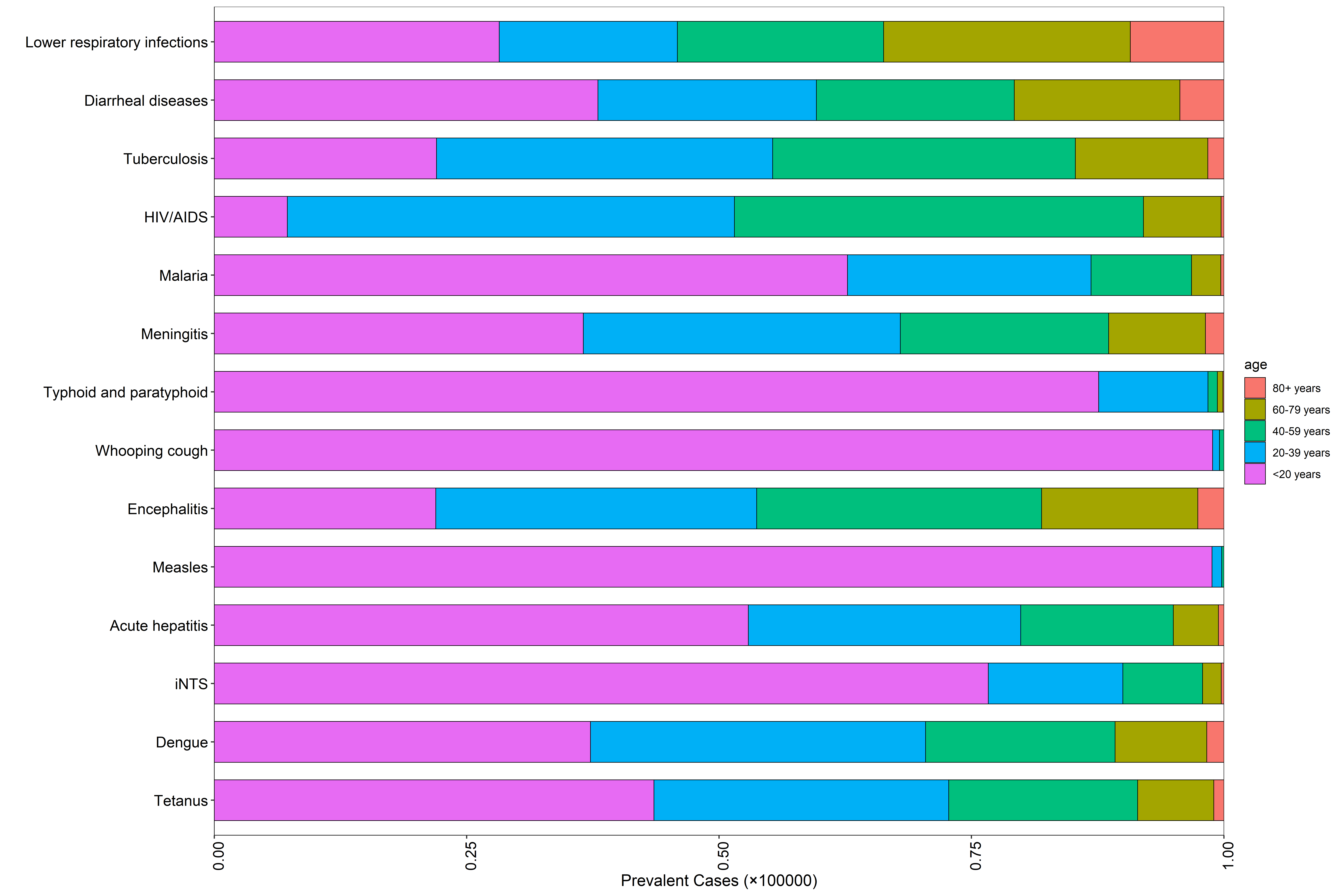 | d.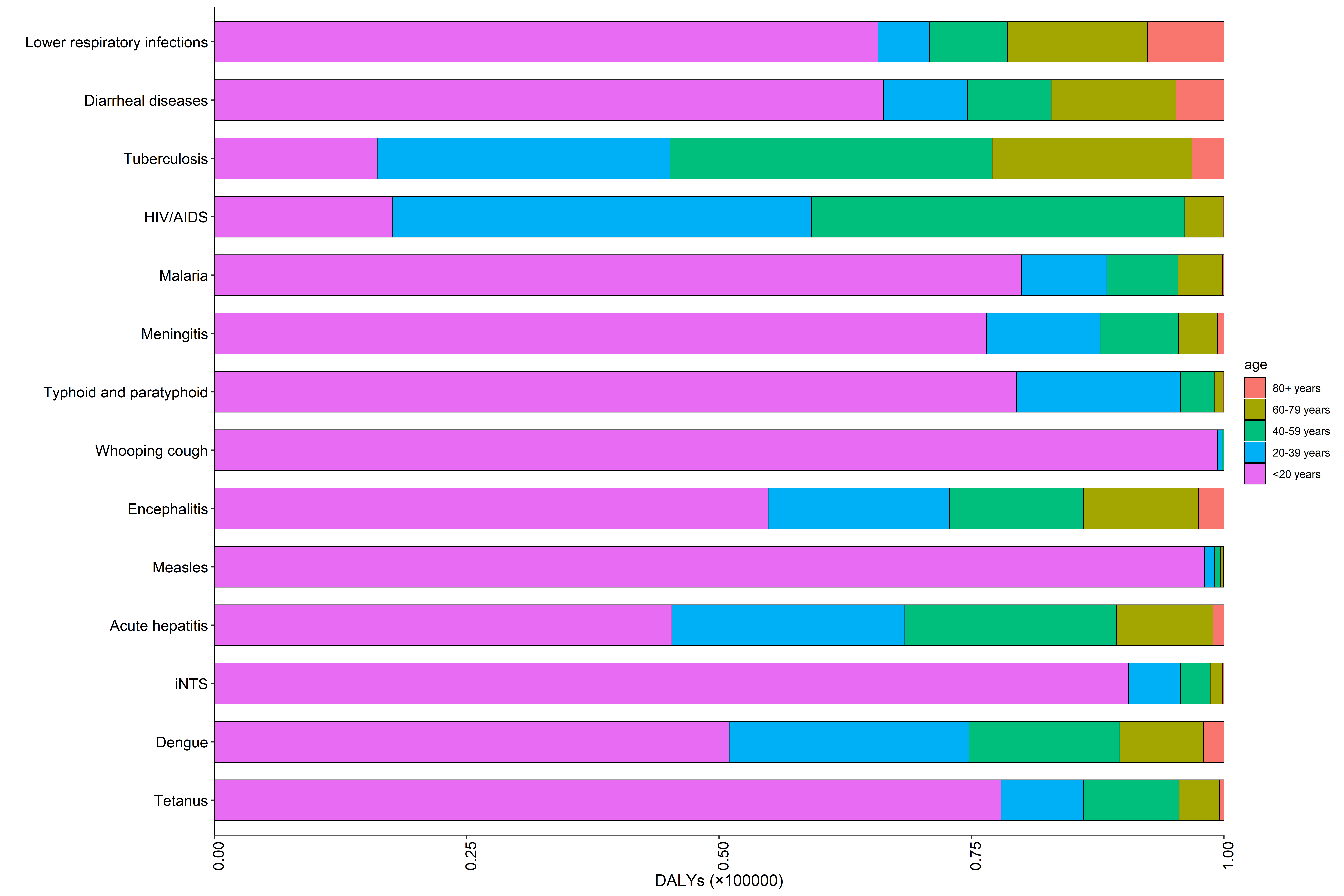 |

Figure 9 Proportions of communicable disease burden by age in 2019.

(a) Incidence; (b) Deaths; (c) Prevalence; (d) DALYs. Abbreviation: DAL Ys disability Adjusted life years.

| a.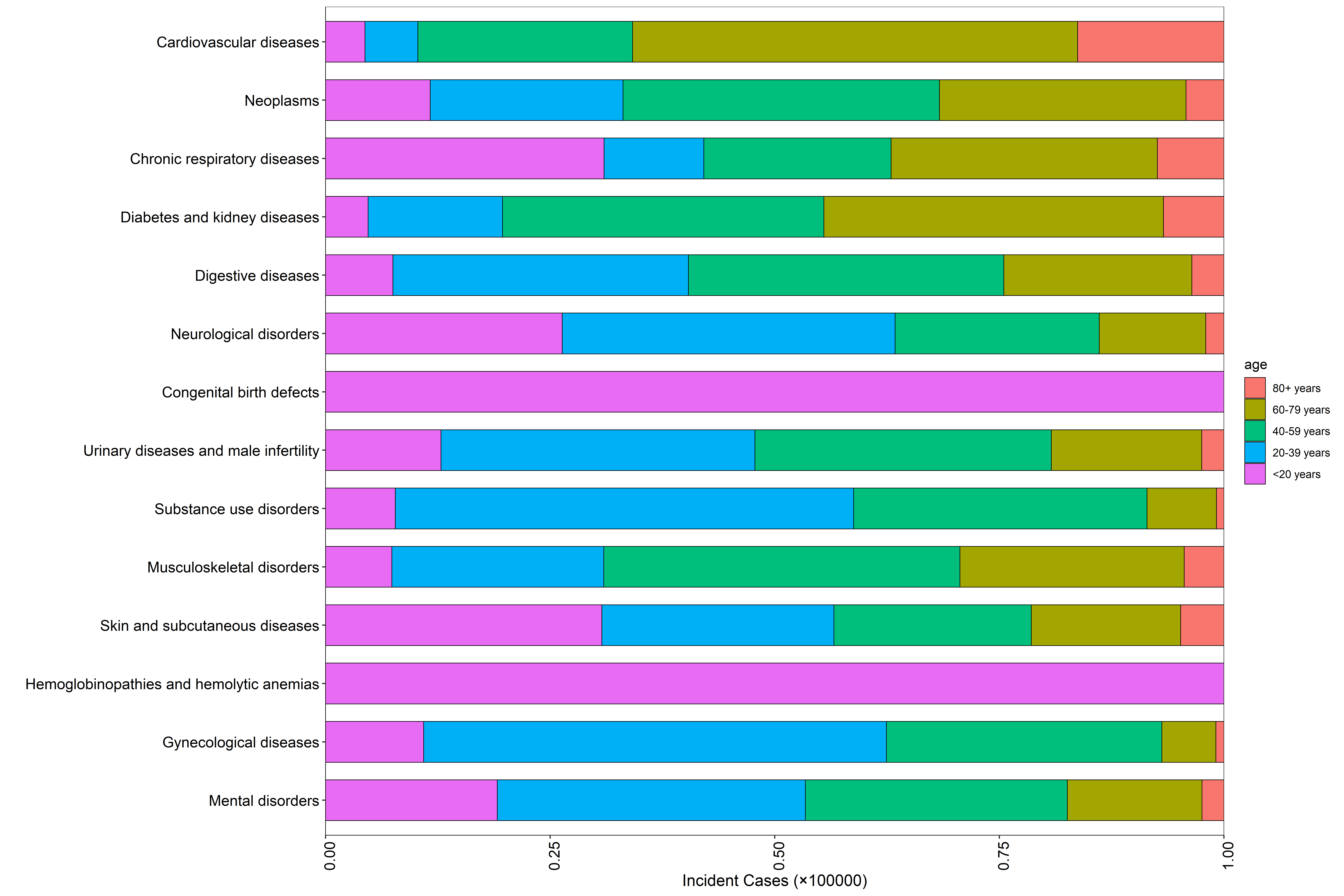 | b.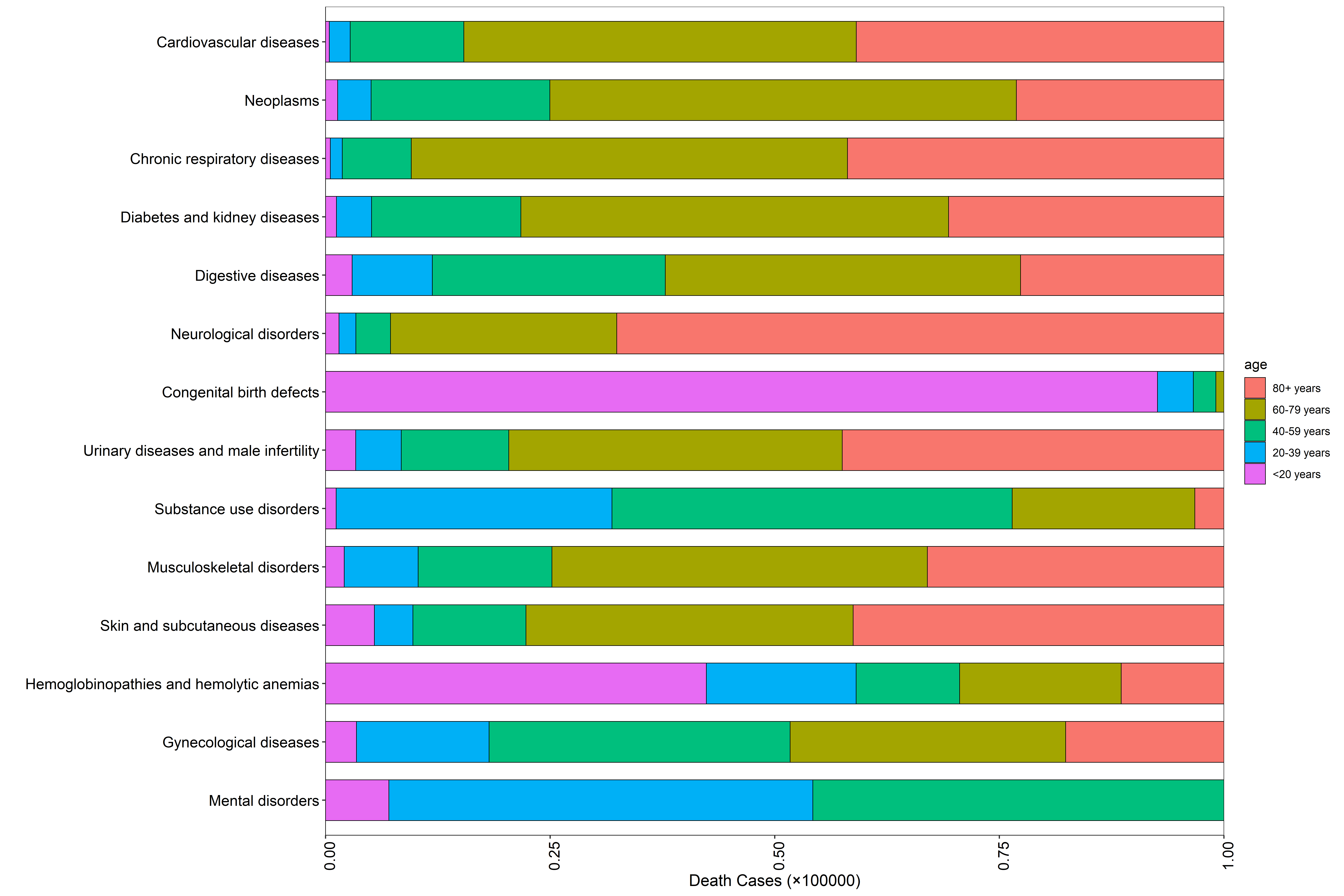 |
| --- | --- |
| c.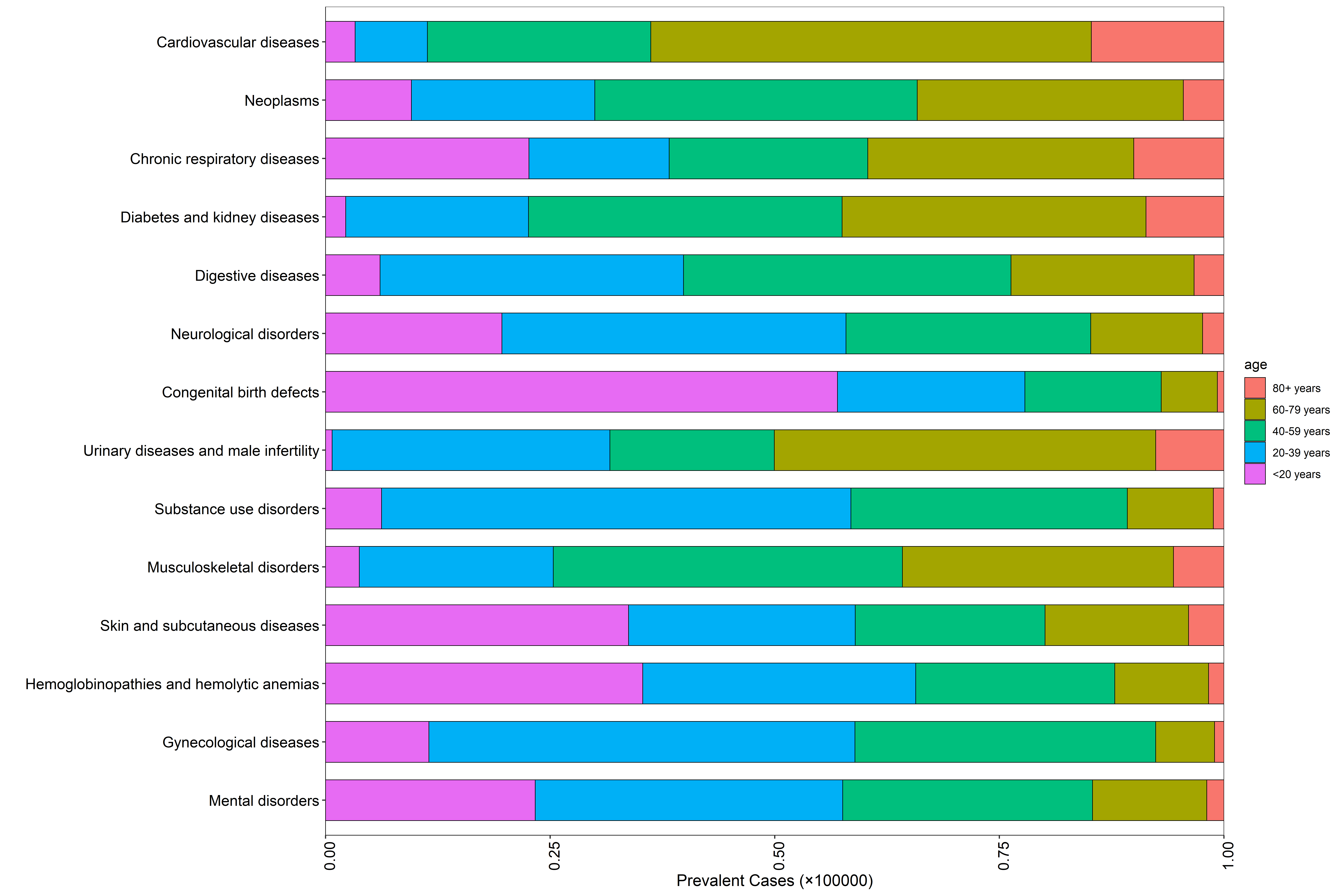 | d.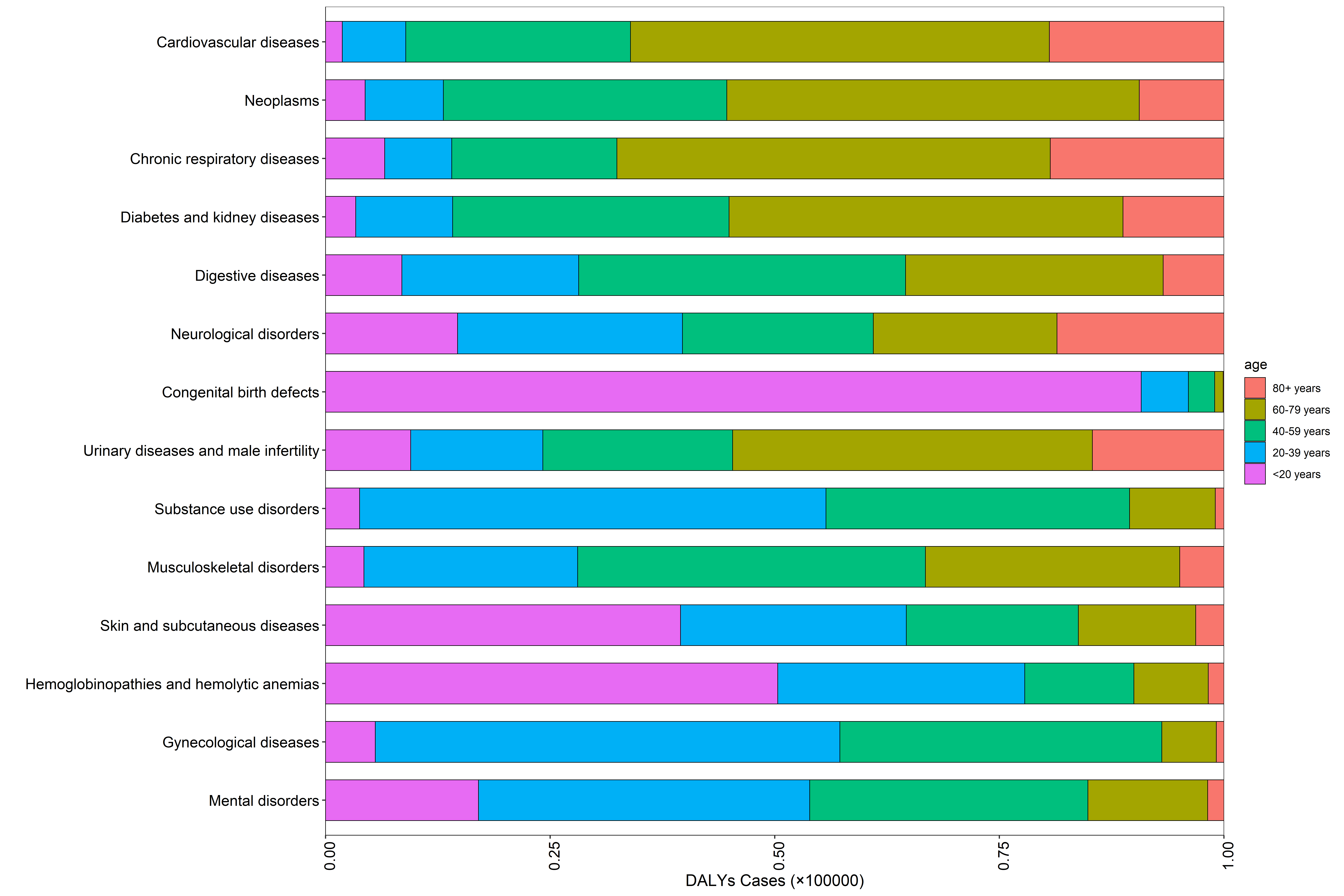 |

Figure 10 Proportions of non-communicable disease burden by age in 2019.

(a) Incidence; (b) Deaths; (c) Prevalence; (d) DALYs. Abbreviation: DAL Ys disability Adjusted life years.


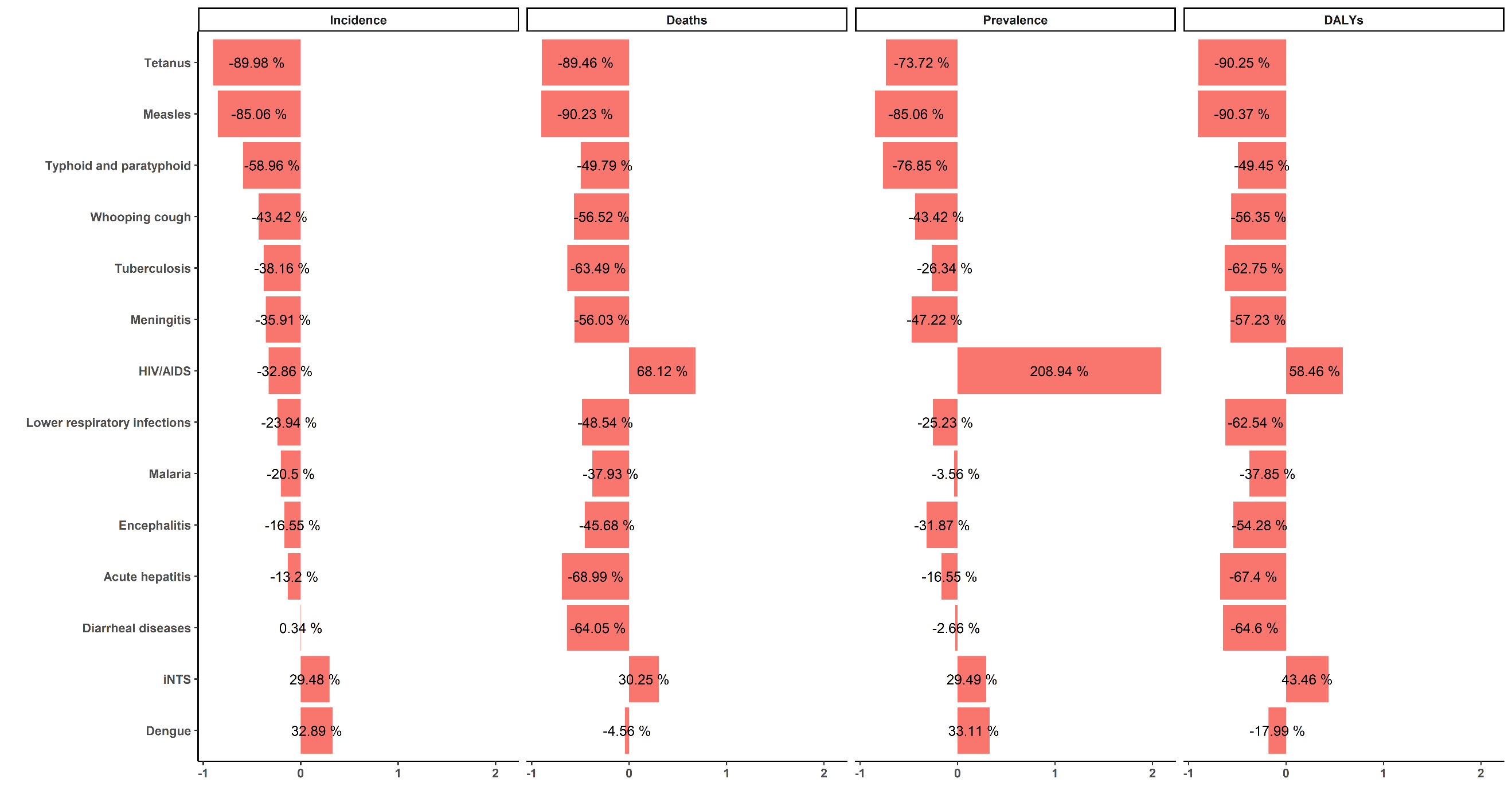


Figure 11 Percentage change in age-standardized rate (per 100,000 population) of different communicable diseases, 1990-2019.


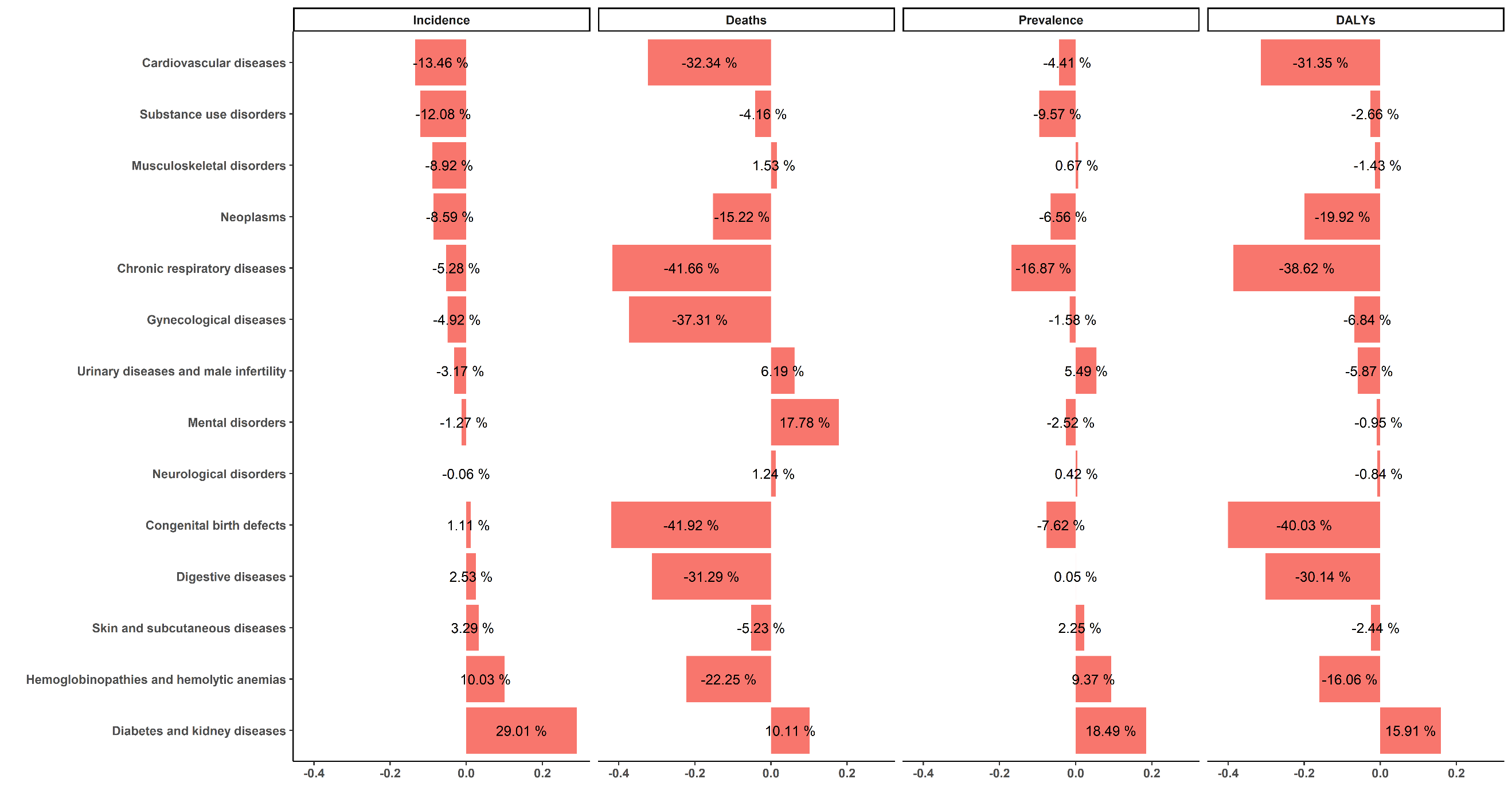


Figure 12 Percentage change in age-standardized rate (per 100,000 population) of different non-communicable diseases, 1990-2019.

## Tables

Table 1 Global burden of communicable diseases in 2019.

| **location** | **sex** | **Incidence** | | **Deaths** | | **Prevalence** | | **DALYs** | |
| --- | --- | --- | --- | --- | --- | --- | --- | --- | --- |
|  |  | **Count**  **(*10^5^)** | **ASIR**  **(per100,000)** | **Count**  **(*10^5^)** | **ASDR**  **(per100,000)** | **Count**  **(*10^5^)** | **ASPR**  **(per100,000)** | **Count**  **(*10^5^)** | **Age-standardized DALY rate**  **(per100,000)** |
| Global | Both | 261290.06 (243203.1 to 281732.2) | 342348.2 (317830.7 to 369569.47) | 78.63 (71.83 to 86.54) | 105.62 (96.63 to 116.65) | 31423.4 (30734.16 to 32136.49) | 39966.92 (39126.62 to 40811.95) | 4203.93 (3840.4 to 4593.74) | 5814.3 (5296.23 to 6407.37) |
|  | Female | 127762.38 (118987.12 to 137553.25) | 336863.92 (312630.3 to 363387.03) | 37.11 (33.11 to 41.92) | 96.2 (85.51 to 108.17) | 15957.24 (15631.16 to 16270.14) | 40613.84 (39810.01 to 41372.03) | 1973.95 (1793.7 to 2138.45) | 5547.99 (5005 to 6064.25) |
|  | Male | 133527.68 (124177.45 to 144084.59) | 348094.22 (323714.59 to 375809.65) | 41.52 (38.16 to 46.06) | 117.16 (107.53 to 129.61) | 15466.16 (15098 to 15851.58) | 39277.3 (38358.46 to 40207.54) | 2229.97 (2031.83 to 2472.75) | 6101.63 (5535.99 to 6799.48) |
| High SDI | Both | 32629.03 (30154.84 to 35597.85) | 350083.47 (318838.07 to 386646.89) | 4.59 (3.93 to 4.93) | 21.18 (18.82 to 22.38) | 2982.89 (2833.03 to 3137.74) | 26004.64 (24800.21 to 27207.29) | 89.84 (82.94 to 97.57) | 649.65 (596.06 to 718.2) |
|  | Female | 16285.2 (15037.15 to 17745.92) | 351677.77 (319575.02 to 389866.63) | 2.23 (1.82 to 2.46) | 16.4 (14.13 to 17.55) | 1602.33 (1524.89 to 1681.76) | 27645.58 (26415.12 to 28947.4) | 40.44 (36.87 to 44.21) | 561.98 (507.62 to 631.82) |
|  | Male | 16343.83 (15137.19 to 17834.72) | 349040.94 (318120.77 to 385146.1) | 2.36 (2.12 to 2.5) | 28.01 (25.45 to 29.3) | 1380.57 (1316.67 to 1453.36) | 24374.77 (23346.05 to 25455.99) | 49.4 (46.25 to 53.22) | 755.87 (703.89 to 818.56) |
| High-middle SDI | Both | 43031.18 (40045.49 to 46395.71) | 316718.88 (291947.18 to 344846.16) | 5.1 (4.74 to 5.36) | 29.63 (27.76 to 31.23) | 5602.09 (5422.58 to 5786.74) | 35751.87 (34597.47 to 36807.55) | 194.28 (182.6 to 204.91) | 1358.16 (1276.13 to 1422.2) |
|  | Female | 21018.47 (19564.09 to 22663.83) | 311190.18 (286959.56 to 338924.7) | 2.2 (1.99 to 2.38) | 22.66 (20.98 to 24.11) | 2874.67 (2782.68 to 2966.1) | 36365.94 (35267.74 to 37410.81) | 79.34 (74.38 to 84.73) | 1131.05 (1056.66 to 1187.15) |
|  | Male | 22012.71 (20434.95 to 23700.5) | 322537.15 (297279.53 to 350745.23) | 2.9 (2.72 to 3.04) | 38.76 (36.42 to 40.6) | 2727.42 (2640.99 to 2812.4) | 35094.47 (34002.12 to 36134.62) | 114.94 (107.72 to 120.94) | 1603.74 (1498.71 to 1674.8) |
| Middle SDI | Both | 76890.33 (71501.35 to 83057.62) | 329226.01 (305069.06 to 356358.29) | 15.15 (14.07 to 16.26) | 70.57 (65.28 to 76.31) | 10624.67 (10334.53 to 10891.53) | 42852.45 (41711.28 to 43869.74) | 722.17 (676.61 to 759.91) | 3251.96 (3043.88 to 3435.68) |
|  | Female | 37080.29 (34493.65 to 40017.1) | 320663.41 (297195.86 to 347053.63) | 6.92 (6.2 to 7.76) | 62.48 (56.1 to 69.73) | 5392.98 (5259.07 to 5511.02) | 43530.1 (42411.45 to 44435.06) | 326.73 (303.27 to 345.25) | 2991.49 (2789.63 to 3164.9) |
|  | Male | 39810.04 (36970.33 to 43037.74) | 337900.84 (312962.02 to 366007.28) | 8.23 (7.68 to 8.91) | 79.9 (74.53 to 86.83) | 5231.69 (5093.51 to 5371.49) | 42120.06 (41027.8 to 43154.55) | 395.43 (369.15 to 421.43) | 3524.14 (3270.4 to 3769.76) |
| Low-middle SDI | Both | 64371.45 (59723.11 to 69404.61) | 367695.42 (342958.48 to 394827.7) | 24.85 (22.32 to 27.99) | 179.16 (158.38 to 205.6) | 7102.23 (6975.96 to 7223.79) | 40126.74 (39450.84 to 40770.68) | 1257.31 (1163.95 to 1356.07) | 7644.84 (7078.04 to 8259.7) |
|  | Female | 31628.56 (29364.23 to 34066.76) | 363398.43 (339226.71 to 389802.68) | 12.24 (10.5 to 14.96) | 172.45 (144.63 to 217.18) | 3517.01 (3472.23 to 3555.05) | 39927.72 (39416.76 to 40345.48) | 609.63 (558.37 to 656.4) | 7449.64 (6817.16 to 8113.21) |
|  | Male | 32742.89 (30380.93 to 35399.39) | 372088.18 (347277.96 to 399346.64) | 12.61 (11.48 to 14.17) | 186.05 (167.83 to 215.05) | 3585.22 (3507.95 to 3658.58) | 40290.15 (39511.1 to 41063.88) | 647.68 (592.64 to 703.18) | 7852.45 (7211.88 to 8481.13) |
| Low SDI | Both | 44204.22 (40756.28 to 47744.2) | 387443.51 (363221.69 to 413261.72) | 28.9 (25.55 to 33.17) | 341.58 (306.18 to 383.17) | 5091.37 (5066.22 to 5108.8) | 46722.78 (46471.34 to 46789.4) | 1937.75 (1708.52 to 2223.91) | 15592.11 (13909.73 to 17527.34) |
|  | Female | 21670.52 (20003.07 to 23396.22) | 379430.77 (355415.4 to 404592.17) | 13.5 (11.87 to 15.27) | 313.82 (270.44 to 368.9) | 2559.86 (2560.5 to 2550.81) | 46919.7 (46953.32 to 46613.02) | 916.63 (810.15 to 1039.06) | 14652.43 (13138.38 to 16310.43) |
|  | Male | 22533.7 (20770.54 to 24420.71) | 395590.81 (370586.82 to 422439.88) | 15.4 (13.49 to 17.92) | 371.03 (332.2 to 420.44) | 2531.51 (2515.72 to 2546.45) | 46498.93 (46151.02 to 46685.83) | 1021.13 (899.88 to 1189.49) | 16544.12 (14892.58 to 18819.85) |

Table 2 Global burden of non-communicable diseases in 2019.

| **location** | **sex** | **Incidence** | | **Deaths** | | **Prevalence** | | **DALYs** | |
| --- | --- | --- | --- | --- | --- | --- | --- | --- | --- |
|  |  | **Count**  **(*10^5^)** | **ASIR**  **(per100,000)** | **Count**  **(*10^5^)** | **ASDR**  **(per100,000)** | **Count**  **(*10^5^)** | **ASPR**  **(per100,000)** | **Count**  **(*10^5^)** | **Age-standardized DALY rate**  **(per100,000)** |
| Global | Both | 130656.8 (125350.51 to 136118.33) | 168397.03 (161059.69 to 176059.97) | 420.34 (400.81 to 439.42) | 539.62 (515.14 to 563.36) | 71043.55 (70406.27 to 71637.14) | 91080.83 (90169.62 to 91943.29) | 16201.66 (14294.71 to 18166.96) | 20204.91 (17826.02 to 22636.76) |
|  | Female | 70721.5 (67923.63 to 73515.27) | 181868.81 (174075.43 to 189744.71) | 196.35 (185.67 to 207.53) | 452.3 (427.71 to 477.77) | 36391.69 (36143.64 to 36639.76) | 93574.66 (92838.86 to 94318.45) | 7905.41 (6876.8 to 9054.29) | 19035.8 (16524.85 to 21830.58) |
|  | Male | 59935.3 (57272.12 to 62735.36) | 155132.08 (147885.47 to 162710.56) | 223.99 (209.97 to 237.53) | 644.32 (606.33 to 680.52) | 34651.85 (34258.6 to 35032.25) | 88689.45 (87608.81 to 89722.07) | 8296.25 (7392.95 to 9221.26) | 21579.51 (19268.95 to 23917.09) |
| High SDI | Both | 17666.85 (17112.36 to 18271.2) | 164187.74 (157384.3 to 171300.71) | 78.59 (77.38 to 80.08) | 384.27 (377.91 to 391.2) | 9460.34 (9401.78 to 9515.73) | 89547.13 (88610.54 to 90458.36) | 2563.75 (2243.12 to 2919.95) | 17507.2 (14927.12 to 20439.22) |
|  | Female | 9796.38 (9492.29 to 10125.03) | 181692.61 (174544.31 to 189238.7) | 38.85 (38.22 to 39.64) | 314.13 (308.84 to 320.31) | 4848.72 (4826.84 to 4870.37) | 92190.75 (91429.83 to 92951.44) | 1280.06 (1099.63 to 1487.86) | 16999.53 (14116.06 to 20332.57) |
|  | Male | 7870.47 (7611.78 to 8166.15) | 147562.27 (140901.71 to 154444.27) | 39.74 (39.1 to 40.44) | 469.44 (461.59 to 478.14) | 4611.62 (4573.53 to 4648.03) | 87058.12 (85931.12 to 88172.36) | 1283.68 (1140.94 to 1436.22) | 18222.08 (15935.35 to 20684.1) |
| High-middle SDI | Both | 23639.81 (22820.97 to 24542.92) | 161016.21 (153952.54 to 168272.74) | 102.54 (96.89 to 107.92) | 524.96 (496.79 to 551.71) | 13296.52 (13202.59 to 13381.09) | 89882.75 (88861.62 to 90802.85) | 3410.28 (3031.29 to 3814.72) | 18667.65 (16416.46 to 21033.27) |
|  | Female | 13023.51 (12567.47 to 13516.79) | 175373.96 (167930.57 to 182892.92) | 49.06 (46.01 to 52.83) | 424.58 (397.78 to 457.52) | 6829.85 (6793.26 to 6865.3) | 92602.77 (91748.89 to 93404.02) | 1650.18 (1431.71 to 1900.99) | 17139.7 (14672.7 to 19991.12) |
|  | Male | 10616.3 (10235.46 to 11063.51) | 147162.16 (140287.26 to 154340.54) | 53.48 (49.16 to 57.79) | 654.19 (605.14 to 702.94) | 6466.67 (6406.04 to 6522.18) | 87353.05 (86172.74 to 88406.66) | 1760.1 (1565.14 to 1952.76) | 20559.13 (18249.85 to 22853.42) |
| Middle SDI | Both | 39193.76 (37659.92 to 40875.68) | 163896.57 (156789.82 to 171646.73) | 127.37 (119.21 to 135.81) | 589.79 (554.01 to 626.59) | 22050.36 (21858.31 to 22222.28) | 90722.94 (89749.07 to 91589.75) | 4991.81 (4400.38 to 5615.25) | 20376.1 (18013.35 to 22855.4) |
|  | Female | 21069.17 (20235.87 to 21905.27) | 176232.9 (168716.66 to 184173.86) | 56.73 (51.92 to 61.62) | 492.53 (452.16 to 533.44) | 11259.07 (11183.11 to 11330.26) | 93263.98 (92472.18 to 94012.9) | 2389.96 (2077.07 to 2750.85) | 19003.48 (16498.45 to 21865.77) |
|  | Male | 18124.59 (17379.4 to 18985.19) | 151764.73 (144713.24 to 159416.58) | 70.65 (64.32 to 77.27) | 702.76 (644.59 to 762.69) | 10791.29 (10673.45 to 10899.78) | 88307.75 (87185.13 to 89314.61) | 2601.85 (2303.31 to 2899.51) | 21930.57 (19506.88 to 24347.09) |
| Low-middle SDI | Both | 30285.26 (28861.42 to 31700.28) | 174860.77 (167267.34 to 182599.1) | 79.67 (73.65 to 85.67) | 649.36 (600.94 to 695.24) | 16110.09 (15943.51 to 16268.49) | 91731.15 (90820.7 to 92590.57) | 3460.84 (3035.8 to 3907.22) | 23021.95 (20400.94 to 25774.33) |
|  | Female | 16219.93 (15479.55 to 16937.61) | 187377.8 (179184.4 to 195478.54) | 36.76 (32.65 to 40.33) | 571.07 (508.22 to 624.7) | 8255.38 (8190.79 to 8321.3) | 94168.25 (93452.45 to 94897.91) | 1709.26 (1472.57 to 1962.79) | 22086.38 (19160 to 25244.32) |
|  | Male | 14065.33 (13380.11 to 14802.23) | 162428.94 (155047.45 to 170254.96) | 42.92 (38.91 to 47.11) | 737.83 (672.96 to 805.15) | 7854.71 (7750.48 to 7952.88) | 89372.14 (88275.71 to 90428.71) | 1751.58 (1543.28 to 1978.75) | 24080.41 (21422.98 to 26952.57) |
| Low SDI | Both | 19279.98 (18122.24 to 20477.87) | 178207.15 (169841.02 to 186829.01) | 31.93 (29.13 to 34.99) | 650.71 (600.47 to 701.27) | 10085.5 (9947.32 to 10225.05) | 92251.39 (91389.14 to 93101.48) | 1765.48 (1529.52 to 2014.06) | 23340.05 (20727 to 26140.43) |
|  | Female | 10252.76 (9636.96 to 10834.89) | 191314.18 (182386.8 to 200014.57) | 14.84 (13.28 to 16.56) | 595.53 (536.82 to 654.05) | 5177.88 (5123.16 to 5233.66) | 94342.61 (93641.6 to 95039.5) | 871.28 (740.87 to 1002.06) | 22742.8 (19803.89 to 25820.78) |
|  | Male | 9027.22 (8440.88 to 9641.42) | 164894.35 (157064.95 to 173222.09) | 17.08 (15.33 to 18.94) | 710.38 (643.17 to 775.65) | 4907.62 (4823.57 to 4990.03) | 90177.12 (89141.78 to 91213.42) | 894.2 (779.79 to 1018.26) | 23964.41 (21281.52 to 26775.63) |

Table 3 Regional burden of communicable diseases in 2019.

| **location** | **sex** | **Incidence** | | **Deaths** | | **Prevalence** | | **DALYs** | |
| --- | --- | --- | --- | --- | --- | --- | --- | --- | --- |
|  |  | **Count**  **(*10^5^)** | **ASIR**  **(per100,000)** | **Count**  **(*10^5^)** | **ASDR**  **(per100,000)** | **Count**  **(*10^5^)** | **ASPR**  **(per100,000)** | **Count**  **(*10^5^)** | **Age-standardized DALY rate**  **(per100,000)** |
| Andean Latin America | Both | 2478.67 ( 2287.68 to 2684.33 ) | 391253.29 ( 361626.82 to 423629.04 ) | 0.45 ( 0.37 to 0.53 ) | 79.77 ( 66.32 to 94.52 ) | 267.43 ( 258.93 to 275.94 ) | 42263.92 ( 40943.87 to 43611.01 ) | 18.43 ( 15.88 to 21.13 ) | 3006.67 ( 2596.31 to 3435.96 ) |
|  | Female | 1214.61 ( 1124.9 to 1318.4 ) | 384454.84 ( 356637.82 to 417095.95 ) | 0.21 ( 0.17 to 0.25 ) | 72.03 ( 59.73 to 86.28 ) | 148.73 ( 143.31 to 153.98 ) | 46583.24 ( 44890.63 to 48198.83 ) | 8.24 ( 7.12 to 9.36 ) | 2677.43 ( 2312.17 to 3046.16 ) |
|  | Male | 1264.06 ( 1160.04 to 1371.14 ) | 397993.3 ( 367031.37 to 431118.29 ) | 0.24 ( 0.19 to 0.28 ) | 87.85 ( 72.1 to 105.14 ) | 118.7 ( 116.52 to 121.1 ) | 37791.57 ( 37068.6 to 38644.19 ) | 10.19 ( 8.72 to 11.77 ) | 3345.04 ( 2860.4 to 3875.16 ) |
| Australasia | Both | 913.84 ( 834.54 to 1000.13 ) | 336006.87 ( 302326.04 to 372890.45 ) | 0.06 ( 0.05 to 0.07 ) | 10.96 ( 9.76 to 11.62 ) | 75.15 ( 71.76 to 78.69 ) | 23505.77 ( 22559.89 to 24322.74 ) | 1.39 ( 1.26 to 1.55 ) | 401.29 ( 366.4 to 448.25 ) |
|  | Female | 457 ( 415.3 to 502.56 ) | 333947.19 ( 298869.2 to 371210.22 ) | 0.03 ( 0.03 to 0.04 ) | 9.79 ( 8.5 to 10.54 ) | 41.43 ( 39.76 to 43.01 ) | 25221.06 ( 24377.44 to 25934.18 ) | 0.68 ( 0.62 to 0.76 ) | 373.52 ( 339.51 to 424.2 ) |
|  | Male | 456.84 ( 417.24 to 502.03 ) | 338167.3 ( 304304.29 to 375550.29 ) | 0.03 ( 0.02 to 0.03 ) | 12.38 ( 11.33 to 13.07 ) | 33.72 ( 32.4 to 34.88 ) | 21710.48 ( 21032.22 to 22226.26 ) | 0.7 ( 0.65 to 0.76 ) | 431.83 ( 401.36 to 460 ) |
| Caribbean | Both | 1759.7 ( 1635.64 to 1892.78 ) | 378122.47 ( 350365.47 to 407715.65 ) | 0.42 ( 0.38 to 0.46 ) | 88.27 ( 79.63 to 98.08 ) | 214.38 ( 209.11 to 219.62 ) | 44380.81 ( 43333.42 to 45396.95 ) | 21.91 ( 19.79 to 24.06 ) | 4967 ( 4472.08 to 5515.06 ) |
|  | Female | 874.78 ( 815.48 to 942.99 ) | 371350.26 ( 343784.5 to 400877.17 ) | 0.2 ( 0.18 to 0.22 ) | 80.98 ( 72.02 to 89.56 ) | 115.96 ( 113.3 to 118.43 ) | 47078.94 ( 46048.79 to 47994.14 ) | 10.46 ( 9.57 to 11.39 ) | 4717.61 ( 4295.34 to 5122.25 ) |
|  | Male | 884.91 ( 818.67 to 952.57 ) | 385125.97 ( 356299.71 to 415627.6 ) | 0.22 ( 0.19 to 0.24 ) | 96.53 ( 86.32 to 106.49 ) | 98.42 ( 96.76 to 100.15 ) | 41537.05 ( 40861.55 to 42227.81 ) | 11.44 ( 10.28 to 12.71 ) | 5226.32 ( 4658.19 to 5810.08 ) |
| Central Asia | Both | 2429.04 ( 2252.67 to 2615.54 ) | 257567.58 ( 239276.59 to 277109.62 ) | 0.37 ( 0.32 to 0.42 ) | 44.33 ( 39.69 to 50.42 ) | 299.13 ( 289.48 to 308.68 ) | 31628.31 ( 30693.03 to 32523.89 ) | 25.41 ( 22.93 to 28.23 ) | 2755.87 ( 2488.18 to 3059.9 ) |
|  | Female | 1206.83 ( 1119.35 to 1300.06 ) | 254825.91 ( 236763.15 to 274052.82 ) | 0.15 ( 0.13 to 0.17 ) | 35.61 ( 31.67 to 40.99 ) | 155.62 ( 152.24 to 158.79 ) | 32355.44 ( 31721.52 to 32993.38 ) | 10.99 ( 10.07 to 11.86 ) | 2408.19 ( 2204.18 to 2616.27 ) |
|  | Male | 1222.22 ( 1132.46 to 1318.84 ) | 260908.38 ( 242454.18 to 280682.79 ) | 0.22 ( 0.19 to 0.25 ) | 55.04 ( 49.79 to 62.25 ) | 143.51 ( 138.46 to 148.33 ) | 30789.97 ( 29758.15 to 31819.56 ) | 14.43 ( 12.97 to 16.26 ) | 3133.08 ( 2827.02 to 3510.64 ) |
| Central Europe | Both | 3238.05 ( 3019.64 to 3488.84 ) | 303455.3 ( 280355.62 to 327907.26 ) | 0.36 ( 0.32 to 0.39 ) | 18.71 ( 16.79 to 20.61 ) | 320.27 ( 306.83 to 335.95 ) | 24929.84 ( 24038.68 to 25856.09 ) | 11.01 ( 9.96 to 11.93 ) | 846.51 ( 772.59 to 912.43 ) |
|  | Female | 1675.79 ( 1562.39 to 1803.13 ) | 308024.29 ( 284681.57 to 332657.18 ) | 0.16 ( 0.14 to 0.17 ) | 13.31 ( 11.91 to 14.81 ) | 172.82 ( 166.22 to 180.56 ) | 26116.72 ( 25297.73 to 26898.56 ) | 4.63 ( 4.18 to 5.13 ) | 697.92 ( 629.1 to 766.86 ) |
|  | Male | 1562.26 ( 1451.31 to 1689.04 ) | 298998.64 ( 275980.64 to 324059.41 ) | 0.2 ( 0.18 to 0.22 ) | 25.99 ( 23.02 to 28.7 ) | 147.45 ( 141.62 to 153.76 ) | 23708.43 ( 22993.93 to 24559.24 ) | 6.38 ( 5.8 to 6.86 ) | 1016.63 ( 938.59 to 1084 ) |
| Central Latin America | Both | 8656.1 ( 7968.09 to 9462.59 ) | 350963.34 ( 322426.47 to 382999.76 ) | 0.91 ( 0.81 to 1.04 ) | 39.49 ( 34.97 to 45.03 ) | 1288.44 ( 1244.15 to 1336.36 ) | 50892.81 ( 49174.5 to 52731.44 ) | 44.03 ( 39.36 to 49.32 ) | 1851.6 ( 1647.17 to 2085.62 ) |
|  | Female | 4308.1 ( 3970.09 to 4692.43 ) | 343464.22 ( 316253.67 to 374333.88 ) | 0.39 ( 0.34 to 0.45 ) | 32.24 ( 28.17 to 37.2 ) | 734.32 ( 711.04 to 757.42 ) | 56206.99 ( 54434.59 to 57939.57 ) | 19.04 ( 16.91 to 21.23 ) | 1575.2 ( 1398.9 to 1768.18 ) |
|  | Male | 4348.01 ( 3985.56 to 4757.35 ) | 358492.01 ( 328914.53 to 390962.65 ) | 0.52 ( 0.46 to 0.59 ) | 47.69 ( 42.22 to 54.24 ) | 554.12 ( 533.63 to 577.04 ) | 45122.88 ( 43469.24 to 46928.11 ) | 24.98 ( 22.21 to 27.9 ) | 2150.08 ( 1906.07 to 2401.69 ) |
| Central Sub-Saharan Africa | Both | 5927.02 ( 5429.11 to 6443.86 ) | 434538.75 ( 405312.61 to 467125.31 ) | 3.65 ( 3.16 to 4.24 ) | 412.25 ( 351.8 to 477.36 ) | 678.37 ( 689.56 to 663.96 ) | 54050.45 ( 55075.74 to 52618.67 ) | 243.33 ( 214.5 to 276.75 ) | 18236.42 ( 16113.39 to 20698.42 ) |
|  | Female | 2878.22 ( 2628.26 to 3132.82 ) | 413366 ( 383319.57 to 445091.87 ) | 1.68 ( 1.46 to 1.92 ) | 354.22 ( 301.88 to 416.46 ) | 335.36 ( 346.17 to 321.11 ) | 52949.66 ( 54728.68 to 50679.96 ) | 113.82 ( 101.68 to 126.93 ) | 16528.46 ( 14682.66 to 18527.3 ) |
|  | Male | 3048.81 ( 2792.69 to 3321.14 ) | 456983.85 ( 424902.05 to 490849.84 ) | 1.96 ( 1.69 to 2.31 ) | 492.17 ( 425.02 to 580.49 ) | 343.01 ( 346.14 to 338.64 ) | 55111.62 ( 55652.57 to 54513.23 ) | 129.5 ( 113.44 to 149.56 ) | 20244.02 ( 17611.47 to 23311.38 ) |
| East Asia | Both | 37108.97 ( 34409.63 to 40367.21 ) | 263838.48 ( 241684.8 to 287821.28 ) | 3.14 ( 2.85 to 3.48 ) | 21.2 ( 19.56 to 23.2 ) | 6757.67 ( 6543.01 to 6965.64 ) | 41744.47 ( 40301.51 to 43424.4 ) | 129.56 ( 117.97 to 138.78 ) | 943.5 ( 877.11 to 1008.4 ) |
|  | Female | 17405.39 ( 16118.71 to 18936.44 ) | 254385.65 ( 232994.4 to 277694.09 ) | 1.27 ( 1.09 to 1.48 ) | 15.79 ( 14.16 to 17.83 ) | 3380.89 ( 3282.28 to 3469.47 ) | 42167.83 ( 40867.04 to 43580.69 ) | 51.3 ( 46.11 to 56.5 ) | 781.94 ( 713.6 to 850.23 ) |
|  | Male | 19703.58 ( 18239.74 to 21382.24 ) | 273265.45 ( 250995.58 to 298042.03 ) | 1.87 ( 1.67 to 2.12 ) | 30.16 ( 27.68 to 33.1 ) | 3376.78 ( 3273.34 to 3490.34 ) | 41289.88 ( 39849.63 to 42945.99 ) | 78.26 ( 71.29 to 84.18 ) | 1126.88 ( 1046.05 to 1206.38 ) |
| Eastern Europe | Both | 7004.16 ( 6516.31 to 7531.02 ) | 348351.14 ( 321357.68 to 376007.17 ) | 0.89 ( 0.83 to 0.95 ) | 33.68 ( 31.84 to 35.23 ) | 732.09 ( 704.52 to 760.85 ) | 31116.19 ( 30015.63 to 32262.46 ) | 43.83 ( 41.1 to 46.78 ) | 1912.63 ( 1805.3 to 2023.89 ) |
|  | Female | 3686.38 ( 3436.08 to 3964.6 ) | 347544.02 ( 320479.86 to 374819.5 ) | 0.28 ( 0.26 to 0.31 ) | 19.3 ( 18.08 to 20.52 ) | 421.88 ( 408.59 to 435.1 ) | 33160.09 ( 32137.01 to 34088.86 ) | 14.65 ( 13.44 to 15.91 ) | 1295.38 ( 1206.56 to 1395.47 ) |
|  | Male | 3317.78 ( 3078.92 to 3577.53 ) | 349573.5 ( 322012.88 to 378375.42 ) | 0.61 ( 0.56 to 0.67 ) | 51.31 ( 47.41 to 55.1 ) | 310.21 ( 302.57 to 320.09 ) | 28765.38 ( 28084.71 to 29598.4 ) | 29.17 ( 27.17 to 31.18 ) | 2604.29 ( 2455.37 to 2750.11 ) |
| Eastern Sub-Saharan Africa | Both | 16121.23 ( 14800.4 to 17453.84 ) | 388622.14 ( 364263.98 to 415013.7 ) | 10.93 ( 9.81 to 12.43 ) | 399.75 ( 367.82 to 439.04 ) | 2115.78 ( 2099.9 to 2127.05 ) | 54529.06 ( 54169.57 to 54714.36 ) | 697.62 ( 618.84 to 801.45 ) | 17147.73 ( 15585.27 to 19062.59 ) |
|  | Female | 7895.45 ( 7265.76 to 8551.78 ) | 374041.12 ( 350487.27 to 399734.9 ) | 4.99 ( 4.45 to 5.67 ) | 344.72 ( 309.77 to 391.58 ) | 1098.28 ( 1093.36 to 1098.92 ) | 55748.86 ( 55493.92 to 55733.01 ) | 326.56 ( 291.53 to 370.69 ) | 15508.65 ( 14085.53 to 17078.92 ) |
|  | Male | 8225.78 ( 7540.72 to 8923.19 ) | 403911.25 ( 377613.31 to 431636.17 ) | 5.94 ( 5.32 to 6.73 ) | 463.37 ( 423.45 to 509.97 ) | 1017.5 ( 1010.47 to 1023.69 ) | 53232.81 ( 52871.52 to 53416.81 ) | 371.05 ( 328.43 to 426.83 ) | 18896.69 ( 17153.82 to 21086.03 ) |
| High-income Asia Pacific | Both | 5428.98 ( 4983.29 to 5953.86 ) | 325394.35 ( 293411.42 to 364101.23 ) | 1.54 ( 1.24 to 1.71 ) | 25.43 ( 21.34 to 27.72 ) | 575.85 ( 552.85 to 604.01 ) | 26681.02 ( 25659.83 to 27591.61 ) | 21.26 ( 19.34 to 22.8 ) | 594.71 ( 542.94 to 656.15 ) |
|  | Female | 2706.75 ( 2479.76 to 2973.38 ) | 326055.74 ( 293477.85 to 364856.94 ) | 0.67 ( 0.49 to 0.77 ) | 16.25 ( 12.66 to 18.37 ) | 317.65 ( 308.72 to 328.42 ) | 28676.46 ( 28021.54 to 29239.91 ) | 8.48 ( 7.45 to 9.33 ) | 461.92 ( 414.26 to 536.52 ) |
|  | Male | 2722.23 ( 2492.6 to 2992.06 ) | 325269.12 ( 293374.15 to 364066.1 ) | 0.87 ( 0.75 to 0.94 ) | 39.8 ( 34.48 to 42.8 ) | 258.2 ( 247.78 to 270.82 ) | 24688.12 ( 23728.95 to 25694.36 ) | 12.78 ( 11.86 to 13.5 ) | 774.09 ( 723.53 to 827.55 ) |
| High-income North America | Both | 13419.42 ( 12354.95 to 14663.28 ) | 395957.39 ( 360728.94 to 437508.86 ) | 1.21 ( 1.08 to 1.27 ) | 18.48 ( 17.25 to 19.03 ) | 1112.5 ( 1055.74 to 1167.14 ) | 27170.1 ( 25888.3 to 28313.35 ) | 31.56 ( 29.06 to 34.68 ) | 701.63 ( 640.35 to 779.35 ) |
|  | Female | 6941.47 ( 6392.79 to 7594.67 ) | 406203.11 ( 368982.55 to 449864.91 ) | 0.62 ( 0.53 to 0.66 ) | 15.77 ( 14.33 to 16.4 ) | 606.45 ( 579.3 to 630.49 ) | 28851.49 ( 27616.03 to 29927.54 ) | 14.98 ( 13.69 to 16.46 ) | 635.38 ( 577.02 to 716.73 ) |
|  | Male | 6477.95 ( 5966.29 to 7091.34 ) | 385938.46 ( 351164.68 to 425220.59 ) | 0.59 ( 0.54 to 0.61 ) | 21.96 ( 20.9 to 22.57 ) | 506.04 ( 482.51 to 531.28 ) | 25424.13 ( 24276.17 to 26465.97 ) | 16.59 ( 15.39 to 18.16 ) | 776.23 ( 719.09 to 852.12 ) |
| North Africa and Middle East | Both | 22776.67 ( 21065.3 to 24581.5 ) | 377630.51 ( 350746.57 to 406066.49 ) | 2.15 ( 1.9 to 2.44 ) | 46.65 ( 41.98 to 52.08 ) | 2211.9 ( 2141.5 to 2284.83 ) | 36375.54 ( 35291.14 to 37558.09 ) | 140.91 ( 127.71 to 154.56 ) | 2476.6 ( 2254.43 to 2705.86 ) |
|  | Female | 10761.93 ( 9944.92 to 11629.17 ) | 370667.65 ( 344049.32 to 398618.34 ) | 0.99 ( 0.87 to 1.12 ) | 43.43 ( 38.74 to 48.76 ) | 1095.07 ( 1065.73 to 1120.81 ) | 37594.85 ( 36580.61 to 38482.44 ) | 67.38 ( 61.36 to 73.19 ) | 2427.9 ( 2219.08 to 2633.17 ) |
|  | Male | 12014.74 ( 11120.42 to 12978.56 ) | 384067.47 ( 356760.21 to 413271.54 ) | 1.16 ( 1.03 to 1.31 ) | 49.82 ( 44.76 to 55.4 ) | 1116.83 ( 1082.75 to 1152.07 ) | 35249.04 ( 34229.97 to 36268.43 ) | 73.53 ( 65.67 to 81.99 ) | 2524.33 ( 2265.76 to 2792.47 ) |
| Oceania | Both | 527.21 ( 486.78 to 570.43 ) | 401808.27 ( 375057.78 to 430844.16 ) | 0.23 ( 0.18 to 0.3 ) | 216.05 ( 169.92 to 285.24 ) | 75.74 ( 73.89 to 78.48 ) | 58663.86 ( 57270.47 to 61113.38 ) | 15.86 ( 13.03 to 19.73 ) | 10855.65 ( 8794.23 to 13875.7 ) |
|  | Female | 244.1 ( 224.58 to 264.51 ) | 380799.65 ( 354240.6 to 410420.82 ) | 0.11 ( 0.08 to 0.15 ) | 202.32 ( 152.6 to 283.9 ) | 36.93 ( 36.41 to 37.77 ) | 58466.89 ( 57699.55 to 60072.68 ) | 7.36 ( 5.94 to 9.29 ) | 10249.75 ( 8155.72 to 13372.4 ) |
|  | Male | 283.11 ( 261.32 to 306.26 ) | 421294.19 ( 392913.54 to 450828.15 ) | 0.12 ( 0.1 to 0.16 ) | 229.93 ( 183.34 to 305.78 ) | 38.81 ( 37.98 to 40.16 ) | 58806.62 ( 57538.51 to 61122.15 ) | 8.5 ( 7.06 to 10.53 ) | 11423.87 ( 9420.36 to 14364.9 ) |
| South Asia | Both | 64616.15 ( 60163.75 to 69576.22 ) | 362512.91 ( 339030.88 to 388561.52 ) | 22.97 ( 20.07 to 26.87 ) | 174.49 ( 148.9 to 210.18 ) | 6265.25 ( 6147.43 to 6384.72 ) | 34212.88 ( 33612.73 to 34840.36 ) | 1081.69 ( 1011.84 to 1159.12 ) | 6713.89 ( 6224.03 to 7234.66 ) |
|  | Female | 31515.01 ( 29357.44 to 33884.15 ) | 361346.48 ( 338230.27 to 387098.02 ) | 11.67 ( 9.47 to 15.18 ) | 177.74 ( 139.44 to 241.31 ) | 2890.75 ( 2859.23 to 2916.63 ) | 32323.54 ( 32013.43 to 32600.89 ) | 534.8 ( 493.74 to 580.38 ) | 6805.01 ( 6225.58 to 7465.44 ) |
|  | Male | 33101.14 ( 30779.62 to 35726.45 ) | 363611.55 ( 339753.55 to 389695.31 ) | 11.31 ( 9.9 to 13.42 ) | 170.22 ( 147.31 to 210.31 ) | 3374.5 ( 3294.74 to 3463.31 ) | 36032.17 ( 35255.67 to 36903.97 ) | 546.9 ( 508.45 to 589.78 ) | 6629.77 ( 6139.36 to 7189.97 ) |
| Southeast Asia | Both | 23713.07 ( 21908.05 to 25904.16 ) | 361365.52 ( 333716.27 to 394522.54 ) | 6 ( 5.44 to 6.62 ) | 111.27 ( 100.73 to 122.44 ) | 3408.31 ( 3318.04 to 3502.28 ) | 49468.47 ( 48196.09 to 50797.79 ) | 260.12 ( 241.52 to 277.38 ) | 4299 ( 3984.93 to 4591.54 ) |
|  | Female | 11326.84 ( 10458.72 to 12369.9 ) | 345464 ( 318057.27 to 377521.63 ) | 2.53 ( 2.21 to 2.93 ) | 88.86 ( 77.39 to 102.25 ) | 1705.73 ( 1670.66 to 1742.32 ) | 49394.27 ( 48322.28 to 50449.08 ) | 106.21 ( 97.81 to 114.2 ) | 3516.49 ( 3249.08 to 3785.51 ) |
|  | Male | 12386.23 ( 11444.38 to 13539.46 ) | 378007.3 ( 348879.84 to 411767.27 ) | 3.47 ( 3.12 to 3.8 ) | 138.45 ( 124.03 to 151.76 ) | 1702.59 ( 1657.86 to 1745.53 ) | 49452.44 ( 48214.7 to 50685.24 ) | 153.91 ( 141.79 to 164.18 ) | 5139.26 ( 4723.43 to 5475.52 ) |
| Southern Latin America | Both | 2269.9 ( 2076.89 to 2496.09 ) | 352584.65 ( 320075.68 to 388138.13 ) | 0.5 ( 0.46 to 0.53 ) | 59.98 ( 55.47 to 63.32 ) | 250.86 ( 242.48 to 258.85 ) | 35601.76 ( 34524.81 to 36624.99 ) | 11.7 ( 11.17 to 11.99 ) | 1585.13 ( 1524.99 to 1610.21 ) |
|  | Female | 1136.33 ( 1037.05 to 1252.44 ) | 346728.86 ( 313285.01 to 384801.11 ) | 0.25 ( 0.22 to 0.27 ) | 49.67 ( 44.95 to 52.91 ) | 151.33 ( 146.01 to 157.03 ) | 41243.44 ( 39906.49 to 42615.92 ) | 5.21 ( 4.94 to 5.35 ) | 1289.83 ( 1228.14 to 1309.44 ) |
|  | Male | 1133.57 ( 1032.28 to 1240.76 ) | 359065.38 ( 325244.15 to 394405.75 ) | 0.25 ( 0.23 to 0.26 ) | 73.35 ( 68.66 to 77.07 ) | 99.53 ( 97.73 to 101.66 ) | 29474.16 ( 29072.99 to 29996.34 ) | 6.49 ( 6.24 to 6.66 ) | 1927.08 ( 1859.94 to 1960.98 ) |
| Southern Sub-Saharan Africa | Both | 2835.28 ( 2623.39 to 3059.54 ) | 364386.32 ( 339367.04 to 390868.61 ) | 3.09 ( 2.85 to 3.41 ) | 447.79 ( 417.43 to 489.32 ) | 463.12 ( 454.95 to 470.43 ) | 59273.5 ( 58269.55 to 60126.29 ) | 161.83 ( 148.21 to 179.96 ) | 21004.73 ( 19390.1 to 23065.9 ) |
|  | Female | 1412.7 ( 1311.72 to 1524.83 ) | 353467.32 ( 328840.97 to 380171.14 ) | 1.49 ( 1.35 to 1.68 ) | 398.34 ( 364.66 to 444.87 ) | 246.63 ( 243.65 to 249.62 ) | 61209.51 ( 60480.47 to 61916.68 ) | 80.12 ( 72.59 to 90.12 ) | 19912.67 ( 18183.43 to 22155.32 ) |
|  | Male | 1422.58 ( 1314.52 to 1536 ) | 376708.97 ( 350883.13 to 403417.73 ) | 1.6 ( 1.45 to 1.8 ) | 516.36 ( 474.92 to 568.36 ) | 216.49 ( 213.22 to 219.21 ) | 57109.54 ( 56301.61 to 57796.99 ) | 81.71 ( 73.46 to 91.66 ) | 22465.73 ( 20373.52 to 24821.26 ) |
| Tropical Latin America | Both | 9395.33 ( 8682.63 to 10216.81 ) | 434673.38 ( 399226.21 to 475015.08 ) | 1.35 ( 1.27 to 1.39 ) | 60.09 ( 56.8 to 62.5 ) | 1084.74 ( 1060.6 to 1104.45 ) | 46026.25 ( 45060.67 to 46884.03 ) | 49.47 ( 47.58 to 50.63 ) | 2278.88 ( 2198.14 to 2319.07 ) |
|  | Female | 4728.46 ( 4375.73 to 5131.61 ) | 429574.23 ( 394531.03 to 468904.55 ) | 0.63 ( 0.58 to 0.67 ) | 50.32 ( 46.68 to 52.95 ) | 572.67 ( 566.21 to 576.15 ) | 47098.09 ( 46710.92 to 47392.36 ) | 21.71 ( 20.7 to 22.46 ) | 1932.77 ( 1858.1 to 1961.34 ) |
|  | Male | 4666.87 ( 4289.01 to 5091.57 ) | 440099.01 ( 403456.81 to 481336.31 ) | 0.71 ( 0.68 to 0.75 ) | 71.39 ( 68.13 to 74.3 ) | 512.07 ( 500.8 to 522.26 ) | 44837.09 ( 43857.9 to 45602.03 ) | 27.76 ( 26.81 to 28.89 ) | 2655.53 ( 2562.59 to 2764.61 ) |
| Western Europe | Both | 12876.53 ( 11878.71 to 14052.82 ) | 324122.48 ( 293423.89 to 360168.79 ) | 1.88 ( 1.63 to 2 ) | 16.97 ( 15.34 to 17.75 ) | 961.57 ( 908.9 to 1014.63 ) | 19285.22 ( 18343.97 to 20278.65 ) | 32.69 ( 30.37 to 35.34 ) | 504.79 ( 465.12 to 566.09 ) |
|  | Female | 6404.27 ( 5901.03 to 6978.85 ) | 319332.71 ( 289056.2 to 354986.75 ) | 1 ( 0.84 to 1.09 ) | 14.01 ( 12.36 to 14.81 ) | 528.05 ( 499.09 to 559.16 ) | 20589.16 ( 19558.05 to 21686.64 ) | 15.62 ( 14.31 to 17 ) | 439.26 ( 396.74 to 497.46 ) |
|  | Male | 6472.26 ( 5967.33 to 7077.3 ) | 329176.09 ( 297926.51 to 365332.72 ) | 0.87 ( 0.79 to 0.92 ) | 21.26 ( 19.66 to 22.07 ) | 433.52 ( 412.66 to 454.82 ) | 17954.07 ( 17183.18 to 18787.49 ) | 17.06 ( 16.04 to 18.37 ) | 582.22 ( 545.27 to 636.69 ) |
| Western Sub-Saharan Africa | Both | 17794.73 ( 16499.83 to 19170.22 ) | 387500.07 ( 363133.12 to 412865.45 ) | 16.55 ( 14.2 to 19.38 ) | 450.59 ( 395 to 515.97 ) | 2264.84 ( 2251.47 to 2275.41 ) | 54046.65 ( 53556.46 to 54441.77 ) | 1160.33 ( 998.73 to 1361.64 ) | 21813.18 ( 19117.57 to 25192.19 ) |
|  | Female | 8981.96 ( 8339.1 to 9661.25 ) | 380092.34 ( 356926.45 to 404678.97 ) | 7.79 ( 6.73 to 9.08 ) | 409.47 ( 354.32 to 475.87 ) | 1200.69 ( 1197.26 to 1205.44 ) | 55944.11 ( 55608.06 to 56225.66 ) | 551.7 ( 481.72 to 636.31 ) | 20384.98 ( 17899.53 to 23400.19 ) |
|  | Male | 8812.76 ( 8148.82 to 9504.6 ) | 395692.11 ( 370621.65 to 421608.77 ) | 8.77 ( 7.44 to 10.42 ) | 495.82 ( 429.65 to 576.35 ) | 1064.15 ( 1062.87 to 1059.86 ) | 51963.83 ( 51713.82 to 52075.3 ) | 608.63 ( 517 to 731.31 ) | 23324.08 ( 20219.09 to 27217.24 ) |

Table 4 Regional burden of non-communicable diseases in 2019.

| **location** | **sex** | **Incidence** | | **Deaths** | | **Prevalence** | | **DALYs** | |
| --- | --- | --- | --- | --- | --- | --- | --- | --- | --- |
|  |  | **Count**  **(*10^5^)** | **ASIR**  **(per100,000)** | **Count**  **(*10^5^)** | **ASDR**  **(per100,000)** | **Count**  **(*10^5^)** | **ASPR**  **(per100,000)** | **Count**  **(*10^5^)** | **Age-standardized DALY rate**  **(per100,000)** |
| Andean Latin America | Both | 1215.65 ( 1161.7 to 1273.19 ) | 192843.44 ( 184382.96 to 202111.12 ) | 2.3 ( 1.93 to 2.72 ) | 421.02 ( 355.35 to 498.01 ) | 579.29 ( 571.58 to 586.27 ) | 91244.99 ( 90044.54 to 92333.43 ) | 103.42 ( 87.08 to 122.43 ) | 17359.04 ( 14625.58 to 20545.83 ) |
|  | Female | 672.93 ( 643.07 to 704.18 ) | 212066.99 ( 202905.53 to 221793.39 ) | 1.13 ( 0.96 to 1.33 ) | 393.55 ( 335.19 to 462.06 ) | 297.9 ( 294.73 to 300.57 ) | 93624.94 ( 92617.15 to 94477.55 ) | 54.4 ( 45.73 to 64.73 ) | 17773.34 ( 14972.51 to 21077.96 ) |
|  | Male | 542.72 ( 516.63 to 570.98 ) | 173332.37 ( 165194.01 to 182385.11 ) | 1.16 ( 0.96 to 1.39 ) | 451.25 ( 376.32 to 536.21 ) | 281.39 ( 276.55 to 285.76 ) | 88922.75 ( 87462.71 to 90254.85 ) | 49.02 ( 41.16 to 58.07 ) | 16954.23 ( 14289.26 to 20042.23 ) |
| Australasia | Both | 552.51 ( 531.49 to 574.74 ) | 180113.66 ( 172062.86 to 188443.57 ) | 1.87 ( 1.84 to 1.9 ) | 351.44 ( 345.32 to 358.09 ) | 265.23 ( 262.9 to 267.63 ) | 87794.32 ( 86614.65 to 88995.36 ) | 63.39 ( 54.74 to 73.04 ) | 16134.33 ( 13641.6 to 18988.05 ) |
|  | Female | 309.44 ( 297.03 to 321.45 ) | 199269.4 ( 190385.31 to 207983.93 ) | 0.91 ( 0.89 to 0.92 ) | 295.23 ( 289.97 to 301.11 ) | 137.62 ( 136.55 to 138.7 ) | 89909.76 ( 88757.61 to 91018.44 ) | 32.09 ( 27.25 to 37.55 ) | 15915.44 ( 13159.26 to 19089.47 ) |
|  | Male | 243.07 ( 232.66 to 254.42 ) | 160707.01 ( 152489.7 to 169233.18 ) | 0.96 ( 0.95 to 0.98 ) | 417.01 ( 409.96 to 424.94 ) | 127.61 ( 126.15 to 129.07 ) | 85703.86 ( 84332.66 to 87155.3 ) | 31.29 ( 27.55 to 35.42 ) | 16447.61 ( 14227.9 to 18958.59 ) |
| Caribbean | Both | 863.79 ( 827.51 to 902.09 ) | 181627.69 ( 173586.79 to 190076.77 ) | 2.88 ( 2.54 to 3.25 ) | 561.83 ( 495.3 to 632.84 ) | 436.99 ( 432.49 to 440.91 ) | 91869.41 ( 90835.71 to 92814.58 ) | 109.24 ( 95.02 to 125.81 ) | 21982.26 ( 19108.98 to 25310.57 ) |
|  | Female | 472.39 ( 452.47 to 492.46 ) | 195164.67 ( 186437.98 to 204143.33 ) | 1.38 ( 1.23 to 1.55 ) | 495.81 ( 439.45 to 557.06 ) | 224.71 ( 222.87 to 226.51 ) | 93106.4 ( 92212.94 to 93989.09 ) | 55.21 ( 47.62 to 63.89 ) | 21418.28 ( 18399.59 to 24812.58 ) |
|  | Male | 391.4 ( 374.27 to 410.32 ) | 167793.52 ( 160018.62 to 176077.1 ) | 1.5 ( 1.32 to 1.7 ) | 638.4 ( 560.74 to 721.82 ) | 212.28 ( 209.61 to 214.75 ) | 90624.5 ( 89416.27 to 91740.5 ) | 54.03 ( 46.96 to 61.96 ) | 22677.61 ( 19734.41 to 25993.77 ) |
| Central Asia | Both | 1456.63 ( 1382.79 to 1535.08 ) | 158072.93 ( 150502.05 to 166113.89 ) | 5.4 ( 4.98 to 5.87 ) | 884.68 ( 825.06 to 954.35 ) | 840.59 ( 829.5 to 850.62 ) | 90035.41 ( 88869.38 to 91078.98 ) | 207.59 ( 183.9 to 232.34 ) | 26284.95 ( 23504.34 to 29116.93 ) |
|  | Female | 800.16 ( 762.44 to 841.83 ) | 170170.71 ( 162156.83 to 178666.52 ) | 2.57 ( 2.37 to 2.77 ) | 739.18 ( 687.17 to 793 ) | 437.44 ( 433 to 441.58 ) | 92560.17 ( 91580.51 to 93472.09 ) | 99.6 ( 87.26 to 113.17 ) | 23318.9 ( 20592.64 to 26294.59 ) |
|  | Male | 656.48 ( 620.22 to 695 ) | 145925.67 ( 138484.55 to 153812.88 ) | 2.83 ( 2.6 to 3.09 ) | 1091.47 ( 1014.48 to 1176.6 ) | 403.15 ( 396.27 to 409.45 ) | 87599.26 ( 86173.7 to 88852.81 ) | 107.99 ( 96.48 to 120.46 ) | 30214.07 ( 27298.16 to 33308.35 ) |
| Central Europe | Both | 1978.51 ( 1907.66 to 2057.51 ) | 160708.84 ( 153468.63 to 168449.84 ) | 12.74 ( 11.41 to 14.09 ) | 589.9 ( 527.22 to 653.45 ) | 1070.04 ( 1061.89 to 1077.61 ) | 89732.18 ( 88528.54 to 90853.12 ) | 347.69 ( 305.29 to 392.98 ) | 19435.41 ( 16932.55 to 22293.64 ) |
|  | Female | 1100.65 ( 1059.9 to 1144.37 ) | 172354.19 ( 164833.64 to 180579.25 ) | 6.39 ( 5.75 to 7.08 ) | 471.96 ( 422.64 to 525.76 ) | 559.93 ( 556.65 to 562.95 ) | 91888.45 ( 90847.6 to 92834.1 ) | 167.5 ( 145.44 to 192.28 ) | 17125.22 ( 14637.43 to 20013.67 ) |
|  | Male | 877.86 ( 844.18 to 915.69 ) | 149397.36 ( 142240.37 to 156863.74 ) | 6.35 ( 5.61 to 7.11 ) | 744.77 ( 660.94 to 831.05 ) | 510.11 ( 504.92 to 514.92 ) | 87680.05 ( 86292.33 to 88986.03 ) | 180.19 ( 158.8 to 203.36 ) | 22310.36 ( 19596.26 to 25261.36 ) |
| Central Latin America | Both | 4277.27 ( 4101.53 to 4462.42 ) | 171787.83 ( 164492.88 to 179653.72 ) | 11.27 ( 9.95 to 12.72 ) | 493.37 ( 436.3 to 555.81 ) | 2241.5 ( 2216.08 to 2265.8 ) | 89069.91 ( 87989.33 to 90118.94 ) | 479.77 ( 414.61 to 559.96 ) | 19711.37 ( 17065.54 to 22967.98 ) |
|  | Female | 2366.9 ( 2271.59 to 2465.61 ) | 185169.78 ( 177486.86 to 193291.42 ) | 5.39 ( 4.71 to 6.13 ) | 432.68 ( 379.04 to 491.53 ) | 1188.9 ( 1179.02 to 1199.06 ) | 92154.89 ( 91273.48 to 93048.27 ) | 246.38 ( 207.97 to 291.73 ) | 19131.82 ( 16184.74 to 22636.94 ) |
|  | Male | 1910.36 ( 1825.53 to 2003.59 ) | 157847.82 ( 150752.57 to 165691.63 ) | 5.88 ( 5.12 to 6.78 ) | 564.52 ( 493.35 to 648.71 ) | 1052.6 ( 1036.39 to 1067.99 ) | 85997.58 ( 84645.08 to 87297.44 ) | 233.39 ( 202.4 to 270.65 ) | 20428.79 ( 17732.02 to 23623.66 ) |
| Central Sub-Saharan Africa | Both | 2268.3 ( 2116.75 to 2427.78 ) | 177796.54 ( 168810.33 to 187329.34 ) | 3.3 ( 2.76 to 3.93 ) | 690.71 ( 591.41 to 810.83 ) | 1198.39 ( 1181.1 to 1215.57 ) | 93676.6 ( 92767.28 to 94567.76 ) | 189.87 ( 157.41 to 223.82 ) | 23596.79 ( 19936.65 to 27539.67 ) |
|  | Female | 1234.58 ( 1153.4 to 1314.58 ) | 193991.49 ( 184174.91 to 203829.67 ) | 1.6 ( 1.34 to 1.94 ) | 623.84 ( 528.23 to 745.42 ) | 619.97 ( 613.28 to 626.96 ) | 95587.03 ( 94897.72 to 96283.06 ) | 93.84 ( 77.24 to 111.05 ) | 22353.39 ( 18790.13 to 26262.7 ) |
|  | Male | 1033.71 ( 959.06 to 1114.65 ) | 161026.89 ( 152544.61 to 170374.54 ) | 1.7 ( 1.41 to 2.03 ) | 774.7 ( 667.52 to 896.62 ) | 578.42 ( 567.37 to 588.9 ) | 91764.47 ( 90614.37 to 92876.23 ) | 96.03 ( 80.31 to 113.84 ) | 25098.68 ( 21325.43 to 29213.16 ) |
| East Asia | Both | 21557.89 ( 20785.28 to 22488.68 ) | 145531.03 ( 138908.49 to 152585.99 ) | 99.58 ( 87.57 to 112.35 ) | 559.61 ( 495.86 to 626.09 ) | 13655.63 ( 13563.25 to 13743.47 ) | 89297.18 ( 88303.31 to 90220.86 ) | 3370.36 ( 2926.69 to 3833.57 ) | 18083.15 ( 15732.93 to 20641.3 ) |
|  | Female | 11485.59 ( 11060.51 to 11960.61 ) | 156218.66 ( 149312.67 to 163645.1 ) | 42.28 ( 35.79 to 49.71 ) | 432.04 ( 368.1 to 504.42 ) | 6882.51 ( 6845.01 to 6917.81 ) | 92053.67 ( 91172.08 to 92880.08 ) | 1539.32 ( 1287.97 to 1811.7 ) | 16209.11 ( 13470.95 to 19109.47 ) |
|  | Male | 10072.3 ( 9676.17 to 10535.81 ) | 135417.45 ( 128640.57 to 142601.51 ) | 57.29 ( 47.4 to 68.64 ) | 733.14 ( 621.94 to 858.74 ) | 6773.11 ( 6711.56 to 6829.85 ) | 86798.65 ( 85616.9 to 87826.11 ) | 1831.04 ( 1534.21 to 2131.49 ) | 20340.45 ( 17277.64 to 23446.18 ) |
| Eastern Europe | Both | 3980.61 ( 3826.78 to 4144.94 ) | 176989.69 ( 169031.66 to 185017.39 ) | 24.37 ( 22.53 to 26.47 ) | 723.33 ( 669.35 to 786.04 ) | 1959.3 ( 1943.86 to 1973.63 ) | 89892.66 ( 88751.75 to 90924.03 ) | 711.77 ( 638.82 to 797.69 ) | 23916.09 ( 21314.48 to 27052.84 ) |
|  | Female | 2364.53 ( 2271.5 to 2456.69 ) | 193986.82 ( 185539.8 to 202474.23 ) | 12.96 ( 11.67 to 14.52 ) | 564.73 ( 506.74 to 635.09 ) | 1074.43 ( 1068.2 to 1080.24 ) | 92312.86 ( 91346.53 to 93228.05 ) | 351.74 ( 308.33 to 400.81 ) | 19957.29 ( 17279.43 to 23196.6 ) |
|  | Male | 1616.07 ( 1548.08 to 1687.72 ) | 159331.91 ( 151860.38 to 167263.97 ) | 11.41 ( 10.08 to 12.84 ) | 961.61 ( 856.13 to 1075.4 ) | 884.87 ( 875.23 to 893.82 ) | 87536.89 ( 86186.16 to 88758.87 ) | 360.03 ( 316.89 to 406.36 ) | 29382.42 ( 26004.87 to 33140.13 ) |
| Eastern Sub-Saharan Africa | Both | 7062.49 ( 6611.53 to 7519.37 ) | 177057.05 ( 168201.04 to 185839.97 ) | 9.59 ( 8.71 to 10.57 ) | 620.31 ( 569.67 to 674.05 ) | 3645.51 ( 3591.56 to 3696.91 ) | 91778.78 ( 90867.39 to 92655.71 ) | 561.38 ( 482.03 to 646.68 ) | 21817.68 ( 19238.99 to 24552.53 ) |
|  | Female | 3768.61 ( 3527.41 to 3991.86 ) | 189280.63 ( 179699.05 to 198423.57 ) | 4.4 ( 3.92 to 4.9 ) | 551.48 ( 496.95 to 606.51 ) | 1902.79 ( 1881.9 to 1923.76 ) | 94198.52 ( 93490.38 to 94887.66 ) | 272.06 ( 230.28 to 317.95 ) | 20507.54 ( 17895.63 to 23480.93 ) |
|  | Male | 3293.88 ( 3068.65 to 3535.03 ) | 164209.8 ( 155823.52 to 173005.18 ) | 5.18 ( 4.69 to 5.77 ) | 698.3 ( 642.39 to 757.82 ) | 1742.72 ( 1709.73 to 1774.6 ) | 89332.27 ( 88187.65 to 90424.44 ) | 289.33 ( 251.12 to 331.63 ) | 23238.85 ( 20708.86 to 25860.82 ) |
| High-income Asia Pacific | Both | 3313.29 ( 3199.42 to 3443.88 ) | 168063.41 ( 160951.5 to 175772.52 ) | 14.83 ( 14.56 to 15.28 ) | 276.03 ( 271.6 to 283.38 ) | 1759.83 ( 1749.16 to 1769.4 ) | 88846.62 ( 87764.66 to 89877.64 ) | 423.01 ( 368.72 to 483.06 ) | 13085.68 ( 10988.47 to 15495.28 ) |
|  | Female | 1784.54 ( 1723.05 to 1854.17 ) | 178781.43 ( 171460.41 to 186479.52 ) | 7.43 ( 7.25 to 7.71 ) | 212.37 ( 208.09 to 218.66 ) | 911.35 ( 907.31 to 915.27 ) | 91600.19 ( 90690.49 to 92528.01 ) | 211.42 ( 180.36 to 245.94 ) | 12606.1 ( 10308.79 to 15364.31 ) |
|  | Male | 1528.75 ( 1473.52 to 1591.52 ) | 157897.22 ( 150938.23 to 165340.01 ) | 7.4 ( 7.27 to 7.61 ) | 355.69 ( 349.4 to 365.17 ) | 848.48 ( 841.62 to 854.74 ) | 86255.2 ( 84974.38 to 87465.58 ) | 211.6 ( 188.13 to 237.23 ) | 13770.42 ( 11887.8 to 15825.41 ) |
| High-income North America | Both | 5766.72 ( 5572.34 to 5976.67 ) | 150390.51 ( 143406.77 to 157471.31 ) | 28.98 ( 28.73 to 29.25 ) | 447.97 ( 444.07 to 452.04 ) | 3397.77 ( 3373.81 to 3421.49 ) | 90131.91 ( 89136.17 to 91116.24 ) | 1046.27 ( 915.58 to 1189.88 ) | 21163.99 ( 18159.13 to 24479.19 ) |
|  | Female | 3279.82 ( 3176.06 to 3395.24 ) | 168322.2 ( 161221.77 to 175857.68 ) | 14.23 ( 14.06 to 14.42 ) | 374.95 ( 370.6 to 379.54 ) | 1764.2 ( 1754.33 to 1773.9 ) | 92398.03 ( 91504.08 to 93276.12 ) | 523.79 ( 450.92 to 605.43 ) | 20373.02 ( 17082.65 to 23990.17 ) |
|  | Male | 2486.9 ( 2394.09 to 2585.98 ) | 132498.58 ( 125603.12 to 139331.2 ) | 14.75 ( 14.59 to 14.93 ) | 536.04 ( 530.12 to 542.77 ) | 1633.57 ( 1618.67 to 1648.5 ) | 87928.47 ( 86759.12 to 89104.45 ) | 522.48 ( 466.22 to 584.18 ) | 22147.62 ( 19485.42 to 25022.19 ) |
| North Africa and Middle East | Both | 9691.47 ( 9191.28 to 10187.05 ) | 160498.06 ( 152835.38 to 168295.3 ) | 24.14 ( 21.94 to 26.48 ) | 643.01 ( 591.78 to 697.89 ) | 5581.08 ( 5518.36 to 5639.57 ) | 91882.71 ( 90880.47 to 92821.41 ) | 1130.84 ( 973.53 to 1300.19 ) | 22580.71 ( 19731.52 to 25645.16 ) |
|  | Female | 5295.21 ( 5028.21 to 5552.76 ) | 181192.91 ( 172599.09 to 189513.5 ) | 10.88 ( 9.93 to 11.91 ) | 597.14 ( 551.41 to 646.65 ) | 2758.81 ( 2734.2 to 2781.64 ) | 94438.15 ( 93617.46 to 95202.02 ) | 555.65 ( 472.48 to 648.51 ) | 22574.94 ( 19540.26 to 25909.24 ) |
|  | Male | 4396.27 ( 4163.13 to 4643.75 ) | 141674.55 ( 134409.19 to 149197.56 ) | 13.25 ( 12.02 to 14.6 ) | 687.2 ( 630.37 to 747.77 ) | 2822.28 ( 2781.46 to 2858.72 ) | 89503.03 ( 88237.06 to 90659.29 ) | 575.18 ( 501.22 to 656.15 ) | 22634.75 ( 19966.83 to 25590.6 ) |
| Oceania | Both | 205.01 ( 192.41 to 217.76 ) | 157362.25 ( 149185.81 to 166008.68 ) | 0.59 ( 0.49 to 0.72 ) | 925.88 ( 793.17 to 1091.64 ) | 118.46 ( 116.63 to 120.14 ) | 91292.72 ( 90193.39 to 92308.77 ) | 28.89 ( 24.07 to 34.36 ) | 30797.77 ( 26069.06 to 36285.63 ) |
|  | Female | 105.26 ( 98.94 to 111.59 ) | 166546.52 ( 158036.82 to 175868.08 ) | 0.25 ( 0.21 to 0.31 ) | 802.44 ( 679.31 to 957.4 ) | 59.34 ( 58.57 to 60.01 ) | 93828.77 ( 92892.57 to 94664.77 ) | 13.18 ( 10.96 to 15.69 ) | 28533.23 ( 24213.1 to 33711.65 ) |
|  | Male | 99.75 ( 93.42 to 106.42 ) | 148568.34 ( 140531.36 to 157182.3 ) | 0.34 ( 0.28 to 0.41 ) | 1051.51 ( 902.01 to 1227.63 ) | 59.12 ( 58 to 60.14 ) | 88910.35 ( 87592.28 to 90158.93 ) | 15.71 ( 13.01 to 18.82 ) | 32956.75 ( 28062.12 to 38873.26 ) |
| South Asia | Both | 32020.72 ( 30609.09 to 33426.13 ) | 179573.48 ( 172020.14 to 187260.73 ) | 77.03 ( 68.93 to 85.45 ) | 617.43 ( 551.52 to 682.83 ) | 16537.16 ( 16365.64 to 16698.37 ) | 91803.22 ( 90866.76 to 92684.04 ) | 3463.69 ( 3032.37 to 3979.49 ) | 22421.57 ( 19673.17 to 25508.32 ) |
|  | Female | 16862.76 ( 16132.03 to 17564.31 ) | 191942.66 ( 183815.52 to 200084.85 ) | 35.18 ( 29.38 to 40.41 ) | 553.92 ( 465.3 to 632.77 ) | 8336.2 ( 8273.43 to 8397.95 ) | 94242.91 ( 93516.23 to 94959.79 ) | 1707.25 ( 1442.69 to 1989.48 ) | 21912.56 ( 18637.52 to 25330.66 ) |
|  | Male | 15157.96 ( 14438.73 to 15886.7 ) | 167719.7 ( 160068.22 to 175479.97 ) | 41.84 ( 35.88 to 48.2 ) | 684.82 ( 594.15 to 784.72 ) | 8200.96 ( 8093.34 to 8305.44 ) | 89507.97 ( 88374.24 to 90593.79 ) | 1756.44 ( 1513.44 to 2032.01 ) | 22987.76 ( 19906.48 to 26426.99 ) |
| Southeast Asia | Both | 10839.07 ( 10337.53 to 11361.74 ) | 161641.35 ( 154040.9 to 169687.92 ) | 33.41 ( 30.53 to 36.1 ) | 624.27 ( 574.65 to 669.47 ) | 6151.6 ( 6090.85 to 6208.83 ) | 90523.31 ( 89514.41 to 91443.4 ) | 1402.17 ( 1231.11 to 1582.93 ) | 22037.69 ( 19507.52 to 24700.72 ) |
|  | Female | 5680.84 ( 5429.32 to 5946.7 ) | 168296.37 ( 160328.47 to 176334.2 ) | 15.53 ( 13.93 to 17.21 ) | 533.26 ( 481.41 to 584.7 ) | 3162.51 ( 3137.65 to 3186.35 ) | 92866.36 ( 92005.92 to 93683.03 ) | 677.25 ( 582.3 to 775.87 ) | 20361.67 ( 17611.77 to 23194.12 ) |
|  | Male | 5158.23 ( 4913.85 to 5424 ) | 155192.87 ( 147607.48 to 163301.68 ) | 17.88 ( 15.99 to 19.66 ) | 733.77 ( 662.77 to 798.47 ) | 2989.08 ( 2952.59 to 3022.95 ) | 88257.1 ( 87098.84 to 89313.82 ) | 724.91 ( 641.44 to 812.55 ) | 24003.84 ( 21323.82 to 26732.86 ) |
| Southern Latin America | Both | 1078.46 ( 1028.42 to 1126.27 ) | 157580.51 ( 149631.36 to 165575.65 ) | 4.06 ( 3.97 to 4.16 ) | 483.27 ( 472.12 to 495.95 ) | 606.19 ( 599.13 to 612.45 ) | 88963.92 ( 87667.71 to 90149.48 ) | 142.67 ( 125.9 to 161.7 ) | 18659.12 ( 16344.34 to 21305.61 ) |
|  | Female | 627.84 ( 600.13 to 655.74 ) | 177716.41 ( 169198.91 to 186228.34 ) | 2.01 ( 1.96 to 2.06 ) | 401.33 ( 391.83 to 412.1 ) | 319.98 ( 317.14 to 322.71 ) | 91730.21 ( 90671.87 to 92757.28 ) | 73.36 ( 63.32 to 84.76 ) | 17922.96 ( 15330.17 to 21004.49 ) |
|  | Male | 450.62 ( 426.87 to 473.76 ) | 136861.57 ( 129305.83 to 144367.28 ) | 2.05 ( 2 to 2.1 ) | 589.31 ( 574.89 to 604.51 ) | 286.22 ( 281.82 to 290.26 ) | 86225.51 ( 84663.04 to 87669.78 ) | 69.31 ( 62.39 to 77.38 ) | 19723.63 ( 17723.23 to 22088.84 ) |
| Southern Sub-Saharan Africa | Both | 1494.74 ( 1427.79 to 1564.11 ) | 193426.95 ( 184959.22 to 202038.3 ) | 3.1 ( 2.9 to 3.3 ) | 624.66 ( 588.52 to 661.26 ) | 707.76 ( 699.84 to 716.09 ) | 90630.23 ( 89677.17 to 91640.68 ) | 140.56 ( 123.13 to 158.93 ) | 21890.02 ( 19461.77 to 24461.04 ) |
|  | Female | 824.55 ( 786.69 to 861.81 ) | 206550.29 ( 197309 to 215820.09 ) | 1.6 ( 1.47 to 1.75 ) | 546.47 ( 503.96 to 591.17 ) | 374.54 ( 371.22 to 377.98 ) | 93248.3 ( 92441.34 to 94086.92 ) | 72.64 ( 62.46 to 83.85 ) | 20499.72 ( 17801.83 to 23402.29 ) |
|  | Male | 670.2 ( 638.57 to 703.51 ) | 179681.56 ( 171756.67 to 187855.48 ) | 1.49 ( 1.4 to 1.58 ) | 738.21 ( 698.04 to 775.78 ) | 333.23 ( 328.18 to 338.29 ) | 88027.82 ( 86813.66 to 89245.88 ) | 67.92 ( 60.26 to 76.02 ) | 23867.08 ( 21558.62 to 26318.96 ) |
| Tropical Latin America | Both | 4427.98 ( 4269.19 to 4591.92 ) | 195322.1 ( 187575.34 to 203875.63 ) | 10.98 ( 10.68 to 11.29 ) | 474.8 ( 461.61 to 488.53 ) | 2072.89 ( 2054.82 to 2088.98 ) | 91251.04 ( 90281.68 to 92138.3 ) | 478.5 ( 418.83 to 546.49 ) | 20284.99 ( 17763.94 to 23168.92 ) |
|  | Female | 2472.96 ( 2382.62 to 2563.14 ) | 211577.26 ( 203004.44 to 220279.98 ) | 5.25 ( 5.06 to 5.46 ) | 401.76 ( 387.63 to 417.63 ) | 1087.92 ( 1081.04 to 1094.38 ) | 93733.94 ( 92932.22 to 94460.63 ) | 243.03 ( 207.96 to 282.98 ) | 19450.2 ( 16605.91 to 22687.17 ) |
|  | Male | 1955.02 ( 1878.5 to 2038.83 ) | 178667.78 ( 171419.83 to 187030.61 ) | 5.73 ( 5.51 to 5.96 ) | 565.71 ( 544.94 to 587.55 ) | 984.97 ( 973.36 to 995.73 ) | 88795.55 ( 87562.17 to 89907.21 ) | 235.48 ( 209.71 to 264.71 ) | 21380.82 ( 19086.24 to 23986.53 ) |
| Western Europe | Both | 8484.27 ( 8200.54 to 8787.47 ) | 180578.83 ( 173135.32 to 188032.16 ) | 38.73 ( 38.28 to 39.27 ) | 375.71 ( 370.99 to 380.81 ) | 4078.42 ( 4054.72 to 4101.51 ) | 89198.66 ( 88272.79 to 90099.09 ) | 1117.4 ( 975.64 to 1280.17 ) | 16430.09 ( 13904.91 to 19347.94 ) |
|  | Female | 4773.56 ( 4610.32 to 4954.76 ) | 200612.77 ( 192093.2 to 209080.02 ) | 19.73 ( 19.48 to 20.06 ) | 308.47 ( 304.58 to 313.11 ) | 2125.2 ( 2116.22 to 2133.33 ) | 92281.55 ( 91541.58 to 92975.28 ) | 573.91 ( 492.07 to 667.42 ) | 16306.34 ( 13389.39 to 19629.46 ) |
|  | Male | 3710.72 ( 3584.91 to 3855.79 ) | 160940.79 ( 154230.7 to 168221.39 ) | 19.01 ( 18.79 to 19.24 ) | 459.31 ( 453.75 to 465.32 ) | 1953.22 ( 1936.39 to 1968.98 ) | 86248.36 ( 85078.24 to 87366.93 ) | 543.49 ( 484.39 to 608.43 ) | 16757.92 ( 14595.51 to 19212.82 ) |
| Western Sub-Saharan Africa | Both | 8121.4 ( 7603.18 to 8655.17 ) | 185240.66 ( 176425.01 to 194705.53 ) | 11.22 ( 9.59 to 13.03 ) | 607.07 ( 540.53 to 676.06 ) | 4139.93 ( 4089.21 to 4193.31 ) | 93546.6 ( 92792.94 to 94331.85 ) | 683.18 ( 564.86 to 806.4 ) | 22087.62 ( 19006.73 to 25248.97 ) |
|  | Female | 4438.36 ( 4163.56 to 4708.67 ) | 200094.66 ( 190591.96 to 209667.47 ) | 5.25 ( 4.33 to 6.25 ) | 556.38 ( 479.38 to 636.04 ) | 2165.44 ( 2143.7 to 2187.78 ) | 95012.5 ( 94360.4 to 95683.91 ) | 341.78 ( 279.53 to 407.99 ) | 21500.48 ( 18071.99 to 25176.49 ) |
|  | Male | 3683.04 ( 3436.27 to 3958.54 ) | 168964.99 ( 160881.33 to 177874.01 ) | 5.97 ( 5.01 to 7.02 ) | 663 ( 575.11 to 762.62 ) | 1974.49 ( 1944.6 to 2004.88 ) | 92019.2 ( 91121.28 to 92954.64 ) | 341.39 ( 282.34 to 404.8 ) | 22701.29 ( 19492.67 to 26045.57 ) |

Table 5 The global burden of different communicable diseases in 2019.

| **cause** | **sex** | **Incidence** | | **Deaths** | | **Prevalence** | | **DALYs** | |
| --- | --- | --- | --- | --- | --- | --- | --- | --- | --- |
|  |  | **Count**  **(*10^5^)** | **ASIR**  **(per100,000)** | **Count**  **(*10^5^)** | **ASDR**  **(per100,000)** | **Count**  **(*10^5^)** | **ASPR**  **(per100,000)** | **Count**  **(*10^5^)** | **Age-standardized DALY rate**  **(per100,000)** |
| Acute hepatitis | Both | 2639.52 ( 2440.34 to 2852.23 ) | 3615.87 ( 3360.49 to 3888.33 ) | 0.79 ( 0.66 to 0.95 ) | 1.02 ( 0.84 to 1.23 ) | 235.9 ( 214.62 to 257.69 ) | 319.49 ( 293.23 to 347.46 ) | 43.46 ( 35.65 to 52.16 ) | 57.95 ( 47.3 to 69.99 ) |
|  | Female | 1207.75 ( 1117.99 to 1310.38 ) | 3400.19 ( 3152.21 to 3666.91 ) | 0.33 ( 0.27 to 0.43 ) | 0.85 ( 0.7 to 1.11 ) | 105.98 ( 96.62 to 116.82 ) | 294.65 ( 270.11 to 322.78 ) | 18.48 ( 15.1 to 24.1 ) | 50.38 ( 40.74 to 66.19 ) |
|  | Male | 1431.77 ( 1307.13 to 1566.66 ) | 3832.22 ( 3509.18 to 4167.82 ) | 0.46 ( 0.36 to 0.56 ) | 1.2 ( 0.94 to 1.47 ) | 129.93 ( 116.75 to 144.98 ) | 344.39 ( 311.05 to 382.02 ) | 24.98 ( 19.41 to 29.87 ) | 65.58 ( 50.67 to 79.11 ) |
| Dengue | Both | 568.79 ( 370.83 to 1013.51 ) | 740.38 ( 478.25 to 1323.07 ) | 0.36 ( 0.09 to 0.44 ) | 0.48 ( 0.12 to 0.59 ) | 33.94 ( 21.74 to 60.45 ) | 44.18 ( 28.07 to 79.17 ) | 23.83 ( 8.28 to 32.73 ) | 32.16 ( 11.11 to 44.15 ) |
|  | Female | 294.02 ( 181.35 to 529.7 ) | 769.95 ( 469.04 to 1392.94 ) | 0.17 ( 0.04 to 0.21 ) | 0.44 ( 0.09 to 0.56 ) | 17.55 ( 10.69 to 31.54 ) | 45.95 ( 27.6 to 83.45 ) | 11.25 ( 3.44 to 15.48 ) | 30.81 ( 9.22 to 42.49 ) |
|  | Male | 274.77 ( 181.2 to 482.66 ) | 712.21 ( 466.46 to 1256.92 ) | 0.19 ( 0.04 to 0.24 ) | 0.52 ( 0.1 to 0.65 ) | 16.39 ( 10.65 to 29 ) | 42.5 ( 27.4 to 75.43 ) | 12.58 ( 3.62 to 17.34 ) | 33.54 ( 9.53 to 46.3 ) |
| Diarrheal diseases | Both | 65816.83 ( 60548.98 to 71426.07 ) | 86102.99 ( 79257.07 to 93570.54 ) | 15.34 ( 10.89 to 22.19 ) | 20.95 ( 15.08 to 29.84 ) | 990.5 ( 921.32 to 1064.8 ) | 1298.44 ( 1204.9 to 1399.22 ) | 809.18 ( 653.76 to 1030.18 ) | 1142.3 ( 928.69 to 1435.52 ) |
|  | Female | 32666.25 ( 30112.07 to 35390.74 ) | 85249.42 ( 78405.89 to 92593.77 ) | 8.27 ( 4.66 to 14.38 ) | 21.18 ( 12.64 to 35.4 ) | 492.67 ( 459.06 to 529.79 ) | 1286.65 ( 1192.44 to 1388.95 ) | 397.98 ( 294.3 to 561.78 ) | 1125.56 ( 849.4 to 1552.44 ) |
|  | Male | 33150.58 ( 30444.45 to 35992.51 ) | 87105.01 ( 80131.09 to 94668.16 ) | 7.07 ( 5.21 to 10.88 ) | 20.67 ( 15.32 to 31.57 ) | 497.83 ( 462.05 to 535.34 ) | 1312.41 ( 1218.89 to 1412.5 ) | 411.2 ( 331.1 to 516.94 ) | 1156.51 ( 930.53 to 1444.79 ) |
| Encephalitis | Both | 14.45 ( 12.8 to 16.15 ) | 19.33 ( 17.06 to 21.7 ) | 0.9 ( 0.77 to 1.23 ) | 1.19 ( 1.01 to 1.62 ) | 44.99 ( 33.72 to 55.73 ) | 56.78 ( 42.65 to 70.29 ) | 47.97 ( 40.59 to 64.18 ) | 65.32 ( 55.05 to 87.29 ) |
|  | Female | 6.94 ( 6.17 to 7.75 ) | 18.62 ( 16.42 to 20.93 ) | 0.43 ( 0.35 to 0.61 ) | 1.09 ( 0.88 to 1.56 ) | 22.45 ( 16.95 to 27.7 ) | 56.44 ( 42.78 to 69.62 ) | 22.09 ( 18.2 to 31.21 ) | 60.83 ( 49.96 to 86.29 ) |
|  | Male | 7.5 ( 6.66 to 8.4 ) | 20.15 ( 17.8 to 22.59 ) | 0.47 ( 0.4 to 0.7 ) | 1.29 ( 1.08 to 1.9 ) | 22.55 ( 16.79 to 28.03 ) | 57.22 ( 42.6 to 71.13 ) | 25.88 ( 21.4 to 36.61 ) | 69.8 ( 57.66 to 98.46 ) |
| HIV/AIDS | Both | 19.89 ( 17.61 to 22.59 ) | 25.24 ( 22.39 to 28.57 ) | 8.64 ( 7.86 to 9.96 ) | 10.72 ( 9.7 to 12.39 ) | 368.48 ( 351.49 to 388.57 ) | 454.32 ( 433.76 to 478.59 ) | 476.32 ( 426.31 to 556.5 ) | 601.49 ( 536.16 to 703.92 ) |
|  | Female | 9.99 ( 8.62 to 11.65 ) | 25.74 ( 22.29 to 30 ) | 4.28 ( 3.83 to 5.02 ) | 10.72 ( 9.51 to 12.61 ) | 200.5 ( 192.67 to 208.98 ) | 497.88 ( 478.37 to 518.72 ) | 242.86 ( 213.58 to 286.17 ) | 620.75 ( 543.97 to 733.98 ) |
|  | Male | 9.91 ( 8.78 to 11.1 ) | 24.82 ( 22.02 to 27.84 ) | 4.35 ( 3.99 to 5 ) | 10.74 ( 9.81 to 12.4 ) | 167.98 ( 155.93 to 183.79 ) | 412.33 ( 383 to 450.7 ) | 233.46 ( 211.09 to 271.4 ) | 583.34 ( 525.67 to 679.55 ) |
| Invasive Non-typhoidal Salmonella (iNTS) | Both | 5.94 ( 4.86 to 7.18 ) | 8.44 ( 6.87 to 10.29 ) | 0.79 ( 0.43 to 1.24 ) | 1.14 ( 0.62 to 1.79 ) | 0.14 ( 0.1 to 0.18 ) | 0.2 ( 0.15 to 0.26 ) | 61.14 ( 33.23 to 97.06 ) | 89.42 ( 48.57 to 141.47 ) |
|  | Female | 2.71 ( 2.22 to 3.3 ) | 7.91 ( 6.45 to 9.68 ) | 0.35 ( 0.19 to 0.55 ) | 1.03 ( 0.57 to 1.62 ) | 0.06 ( 0.05 to 0.08 ) | 0.18 ( 0.14 to 0.25 ) | 27.05 ( 14.84 to 42.79 ) | 81.72 ( 44.93 to 129.68 ) |
|  | Male | 3.23 ( 2.64 to 3.9 ) | 8.96 ( 7.29 to 10.9 ) | 0.44 ( 0.24 to 0.69 ) | 1.24 ( 0.67 to 1.95 ) | 0.08 ( 0.06 to 0.1 ) | 0.21 ( 0.16 to 0.28 ) | 34.09 ( 18.44 to 53.33 ) | 96.72 ( 52.23 to 150.83 ) |
| Lower respiratory infections | Both | 4889.03 ( 4575.73 to 5226.36 ) | 6294.97 ( 5887.37 to 6737.34 ) | 24.93 ( 22.68 to 27.36 ) | 34.31 ( 31.08 to 37.88 ) | 109.7 ( 103.14 to 116.14 ) | 141.85 ( 133.42 to 150.34 ) | 971.9 ( 848.71 to 1130.83 ) | 1386.1 ( 1203.47 to 1622.79 ) |
|  | Female | 2319.26 ( 2173.94 to 2476.39 ) | 5837.29 ( 5450.42 to 6245.71 ) | 11.97 ( 10.67 to 13.25 ) | 30.46 ( 27.19 to 33.95 ) | 51.96 ( 48.88 to 55.01 ) | 131.55 ( 123.69 to 139.75 ) | 457.84 ( 398.63 to 530.83 ) | 1319.41 ( 1135.4 to 1537.88 ) |
|  | Male | 2569.77 ( 2403.7 to 2747.01 ) | 6832.02 ( 6393.85 to 7313.07 ) | 12.96 ( 11.85 to 14.23 ) | 39.71 ( 35.99 to 43.56 ) | 57.73 ( 54.24 to 61.17 ) | 153.82 ( 144.73 to 163.08 ) | 514.06 ( 448.17 to 599.03 ) | 1468.25 ( 1277.31 to 1716.05 ) |
| Malaria | Both | 2313.57 ( 1860.34 to 2902.17 ) | 3247.02 ( 2602.06 to 4109.56 ) | 6.43 ( 3.02 to 11.54 ) | 8.95 ( 4.23 to 16 ) | 1807.16 ( 1631.13 to 2030.8 ) | 2466.35 ( 2226.78 to 2773.01 ) | 464.38 ( 235.07 to 801.16 ) | 668.11 ( 337.28 to 1152.22 ) |
|  | Female | 1158.35 ( 931.8 to 1450.28 ) | 3334.43 ( 2666.85 to 4223.5 ) | 3.1 ( 1.47 to 5.5 ) | 8.82 ( 4.23 to 15.63 ) | 912.78 ( 823.23 to 1025.88 ) | 2541.55 ( 2294 to 2858.09 ) | 229.33 ( 117.98 to 387.47 ) | 680.28 ( 348.53 to 1148.64 ) |
|  | Male | 1155.22 ( 928.59 to 1451.86 ) | 3164.05 ( 2537.39 to 4001.64 ) | 3.34 ( 1.56 to 6 ) | 9.11 ( 4.3 to 16.3 ) | 894.38 ( 807.48 to 1004.52 ) | 2394.37 ( 2161.9 to 2689.41 ) | 235.05 ( 116.91 to 410.4 ) | 657.49 ( 327.12 to 1151.54 ) |
| Measles | Both | 128.06 ( 45.49 to 276.9 ) | 191.04 ( 67.85 to 413.04 ) | 0.83 ( 0.31 to 1.8 ) | 1.24 ( 0.46 to 2.69 ) | 3.51 ( 1.25 to 7.59 ) | 5.23 ( 1.86 to 11.32 ) | 70.26 ( 25.27 to 153.76 ) | 104.75 ( 37.53 to 229.56 ) |
|  | Female | 62.68 ( 22.24 to 135.39 ) | 193.18 ( 68.52 to 417.2 ) | 0.41 ( 0.15 to 0.89 ) | 1.25 ( 0.46 to 2.73 ) | 1.72 ( 0.61 to 3.71 ) | 5.29 ( 1.88 to 11.43 ) | 34.37 ( 12.25 to 75.65 ) | 105.86 ( 37.53 to 233.36 ) |
|  | Male | 65.38 ( 23.25 to 141.51 ) | 189.02 ( 67.21 to 409.1 ) | 0.43 ( 0.16 to 0.92 ) | 1.23 ( 0.45 to 2.66 ) | 1.79 ( 0.64 to 3.88 ) | 5.18 ( 1.84 to 11.21 ) | 35.88 ( 12.82 to 78.6 ) | 103.72 ( 36.92 to 227.46 ) |
| Meningitis | Both | 25.07 ( 21.13 to 29.89 ) | 35.42 ( 29.57 to 42.47 ) | 2.36 ( 2.04 to 2.77 ) | 3.29 ( 2.82 to 3.89 ) | 76.84 ( 65.9 to 91.32 ) | 99.88 ( 85.55 to 118.79 ) | 163.33 ( 137.75 to 196.1 ) | 234.01 ( 195.83 to 282.54 ) |
|  | Female | 11.83 ( 9.95 to 14.05 ) | 33.94 ( 28.38 to 40.56 ) | 1.07 ( 0.91 to 1.26 ) | 3.01 ( 2.53 to 3.57 ) | 37.81 ( 32.44 to 44.8 ) | 98.77 ( 84.82 to 117.17 ) | 73.5 ( 60.82 to 88.9 ) | 215.73 ( 176.96 to 262.54 ) |
|  | Male | 13.25 ( 11.13 to 15.81 ) | 36.84 ( 30.66 to 44.3 ) | 1.29 ( 1.11 to 1.53 ) | 3.56 ( 3.05 to 4.27 ) | 39.02 ( 33.36 to 46.43 ) | 101.02 ( 86.3 to 120.13 ) | 89.83 ( 75.3 to 109.85 ) | 251.51 ( 209.74 to 308.95 ) |
| Tetanus | Both | 0.74 ( 0.53 to 1.01 ) | 1.03 ( 0.74 to 1.42 ) | 0.35 ( 0.26 to 0.48 ) | 0.49 ( 0.37 to 0.68 ) | 0.62 ( 0.45 to 0.81 ) | 0.81 ( 0.59 to 1.07 ) | 23.16 ( 17.7 to 32.79 ) | 33.74 ( 25.57 to 47.85 ) |
|  | Female | 0.32 ( 0.23 to 0.45 ) | 0.9 ( 0.65 to 1.28 ) | 0.15 ( 0.11 to 0.21 ) | 0.44 ( 0.32 to 0.61 ) | 0.31 ( 0.22 to 0.4 ) | 0.82 ( 0.59 to 1.08 ) | 10.32 ( 7.63 to 14.74 ) | 31.1 ( 22.97 to 44.41 ) |
|  | Male | 0.42 ( 0.28 to 0.6 ) | 1.15 ( 0.78 to 1.66 ) | 0.19 ( 0.14 to 0.29 ) | 0.54 ( 0.38 to 0.8 ) | 0.31 ( 0.23 to 0.41 ) | 0.81 ( 0.59 to 1.06 ) | 12.84 ( 9.4 to 19.79 ) | 36.31 ( 26.69 to 55.68 ) |
| Tuberculosis | Both | 84.97 ( 74.46 to 97.28 ) | 106.71 ( 93.96 to 121.94 ) | 11.8 ( 10.79 to 12.93 ) | 14.64 ( 13.39 to 16.03 ) | 18297.29 ( 16653.73 to 20159.28 ) | 23085.13 ( 20993.95 to 25380.06 ) | 470.3 ( 428.14 to 513.78 ) | 590.42 ( 536.85 to 646.42 ) |
|  | Female | 37.43 ( 32.69 to 43.06 ) | 94.93 ( 83.02 to 109.08 ) | 4.19 ( 3.66 to 5.02 ) | 10.08 ( 8.81 to 12.07 ) | 8843.41 ( 8032.54 to 9762.54 ) | 22334.02 ( 20271.33 to 24598.98 ) | 172.32 ( 151.62 to 199.94 ) | 437.03 ( 385.88 to 506.96 ) |
|  | Male | 47.55 ( 41.57 to 54.44 ) | 119.61 ( 105.29 to 136.47 ) | 7.61 ( 6.88 to 8.36 ) | 19.72 ( 17.85 to 21.63 ) | 9453.89 ( 8612.17 to 10419.62 ) | 23844.79 ( 21695.94 to 26216.02 ) | 297.98 ( 268.58 to 327.82 ) | 750.49 ( 675.73 to 825.02 ) |
| Typhoid and paratyphoid | Both | 130.37 ( 83.73 to 196.29 ) | 177.26 ( 113.45 to 264.59 ) | 1.33 ( 0.66 to 2.29 ) | 1.84 ( 0.89 to 3.14 ) | 3.27 ( 1.93 to 5.29 ) | 4.56 ( 2.68 to 7.39 ) | 96.92 ( 46.94 to 166.82 ) | 135.37 ( 65.14 to 232.59 ) |
|  | Female | 58.46 ( 37.47 to 87.71 ) | 162.15 ( 103.68 to 242.95 ) | 0.59 ( 0.29 to 1.04 ) | 1.68 ( 0.81 to 2.92 ) | 1.37 ( 0.77 to 2.28 ) | 3.93 ( 2.2 to 6.53 ) | 43.05 ( 21.11 to 74.8 ) | 123.51 ( 60.2 to 213.8 ) |
|  | Male | 71.91 ( 46.36 to 108.16 ) | 191.77 ( 123.29 to 285.33 ) | 0.74 ( 0.36 to 1.26 ) | 1.99 ( 0.97 to 3.39 ) | 1.9 ( 1.12 to 3.04 ) | 5.17 ( 3.05 to 8.25 ) | 53.87 ( 25.79 to 91.96 ) | 146.62 ( 70.72 to 249.51 ) |
| Whooping cough | Both | 195.19 ( 149.33 to 248.12 ) | 293.58 ( 224.59 to 373.18 ) | 1.17 ( 0.54 to 2.12 ) | 1.75 ( 0.8 to 3.19 ) | 26.74 ( 20.46 to 33.99 ) | 40.22 ( 30.77 to 51.12 ) | 101.92 ( 47.37 to 184.82 ) | 153.41 ( 71.25 to 278.26 ) |
|  | Female | 106.54 ( 81.5 to 135.42 ) | 331.27 ( 253.4 to 421.08 ) | 0.63 ( 0.29 to 1.15 ) | 1.96 ( 0.91 to 3.58 ) | 14.59 ( 11.16 to 18.55 ) | 45.38 ( 34.71 to 57.68 ) | 55.22 ( 25.84 to 100.38 ) | 171.85 ( 80.44 to 312.46 ) |
|  | Male | 88.65 ( 67.83 to 112.7 ) | 258.29 ( 197.62 to 328.35 ) | 0.53 ( 0.24 to 0.96 ) | 1.55 ( 0.71 to 2.81 ) | 12.14 ( 9.29 to 15.44 ) | 35.38 ( 27.07 to 44.98 ) | 46.7 ( 21.63 to 83.82 ) | 136.16 ( 63.13 to 244.29 ) |

Table 6 The global burden of different non-communicable diseases in 2019.

| **cause** | **sex** | **Incidence** | | **Deaths** | | **Prevalence** | | **DALYs** | |
| --- | --- | --- | --- | --- | --- | --- | --- | --- | --- |
|  |  | **Count**  **(*10^5^)** | **ASIR**  **(per100,000)** | **Count**  **(*10^5^)** | **ASDR**  **(per100,000)** | **Count**  **(*10^5^)** | **ASPR**  **(per100,000)** | **Count**  **(*10^5^)** | **Age-standardized DALY rate**  **(per100,000)** |
| Cardiovascular diseases | Both | 554.52 ( 523.31 to 588.59 ) | 684.33 ( 646.42 to 725.55 ) | 185.63 ( 170.8 to 197.21 ) | 239.85 ( 219.43 to 254.9 ) | 5231.99 ( 4971.32 to 5501.57 ) | 6431.57 ( 6109.95 to 6759.8 ) | 3931.07 ( 3677.76 to 4172.58 ) | 4863.64 ( 4548.71 to 5164.24 ) |
|  | Female | 276.39 ( 260.84 to 293 ) | 642.72 ( 607.18 to 680.06 ) | 89.37 ( 79.24 to 97.05 ) | 204.03 ( 180.93 to 221.55 ) | 2752.29 ( 2614.26 to 2898.27 ) | 6402.62 ( 6078.89 to 6740.01 ) | 1708.41 ( 1562.79 to 1839.23 ) | 3948.54 ( 3616.57 to 4248.62 ) |
|  | Male | 278.13 ( 261.79 to 295.72 ) | 729.81 ( 687.74 to 774.48 ) | 96.26 ( 89.04 to 102.84 ) | 280.83 ( 259.16 to 299.8 ) | 2479.71 ( 2349.49 to 2613.68 ) | 6471.7 ( 6136.67 to 6814.43 ) | 2222.67 ( 2064.25 to 2371.83 ) | 5851.28 ( 5448.15 to 6247.67 ) |
| Chronic respiratory diseases | Both | 776.25 ( 688.85 to 879.3 ) | 1001.57 ( 882.99 to 1144.44 ) | 39.74 ( 35.82 to 43.04 ) | 51.28 ( 45.9 to 55.51 ) | 4545.57 ( 4173.54 to 4991.44 ) | 5789.16 ( 5290.68 to 6418.14 ) | 1035.33 ( 947.92 to 1122.66 ) | 1293.74 ( 1182.99 to 1403.57 ) |
|  | Female | 386.14 ( 343.63 to 433.92 ) | 973.44 ( 860.64 to 1108.64 ) | 17.41 ( 14.57 to 19.61 ) | 39.73 ( 33.24 to 44.75 ) | 2311.53 ( 2125.59 to 2526.6 ) | 5695.91 ( 5209.31 to 6282.15 ) | 465.26 ( 411.2 to 513.71 ) | 1093.03 ( 965.72 to 1208.96 ) |
|  | Male | 390.11 ( 344.22 to 446.06 ) | 1034.12 ( 910.04 to 1187.41 ) | 22.34 ( 20.3 to 24.53 ) | 66.72 ( 60.55 to 73.06 ) | 2234.04 ( 2036.8 to 2484.08 ) | 5907.65 ( 5382.04 to 6581.16 ) | 570.07 ( 518.95 to 626.42 ) | 1538.69 ( 1399.47 to 1690.3 ) |
| Congenital birth defects | Both | 85.18 ( 73.05 to 99.21 ) | 131.52 ( 112.79 to 153.17 ) | 5.49 ( 4.48 to 6.9 ) | 8.25 ( 6.71 to 10.42 ) | 508.62 ( 443.13 to 586.51 ) | 692.1 ( 604.58 to 797.71 ) | 527.85 ( 436.02 to 655.22 ) | 788.75 ( 650.07 to 984.61 ) |
|  | Female | 40.66 ( 34.78 to 47.47 ) | 130.03 ( 111.24 to 151.8 ) | 2.47 ( 1.99 to 3.16 ) | 7.66 ( 6.15 to 9.8 ) | 245.16 ( 214.28 to 282.09 ) | 678.7 ( 594.46 to 776.32 ) | 239.48 ( 198.67 to 301.09 ) | 737.05 ( 608.06 to 929.8 ) |
|  | Male | 44.52 ( 38.07 to 51.88 ) | 132.9 ( 113.65 to 154.88 ) | 3.02 ( 2.4 to 4 ) | 8.81 ( 6.98 to 11.71 ) | 263.47 ( 227.88 to 305.89 ) | 705.38 ( 612.34 to 817.28 ) | 288.37 ( 231.46 to 371.66 ) | 837.17 ( 670.27 to 1084.26 ) |
| Diabetes and kidney diseases | Both | 419.49 ( 397.94 to 443.24 ) | 510.65 ( 484.86 to 538.32 ) | 29.89 ( 27.73 to 31.83 ) | 37.89 ( 34.98 to 40.37 ) | 9935.55 ( 9465.13 to 10407.95 ) | 12121.36 ( 11555.67 to 12672.4 ) | 1127.26 ( 992.72 to 1283.22 ) | 1377.72 ( 1214.16 to 1565.71 ) |
|  | Female | 214.69 ( 203.72 to 226.41 ) | 505.9 ( 480.07 to 533.52 ) | 14.87 ( 13.47 to 16.04 ) | 34.13 ( 30.94 to 36.8 ) | 5173.93 ( 4930.99 to 5419.97 ) | 12200.86 ( 11621.48 to 12775.89 ) | 541.29 ( 472.8 to 619.55 ) | 1264.14 ( 1103.14 to 1445.93 ) |
|  | Male | 204.79 ( 194.02 to 216.21 ) | 514.7 ( 489.11 to 543.35 ) | 15.02 ( 14 to 16.08 ) | 42.72 ( 39.64 to 45.65 ) | 4761.63 ( 4531.61 to 4991.69 ) | 12034.84 ( 11478.13 to 12591.17 ) | 585.97 ( 513.27 to 664.54 ) | 1505.24 ( 1323.41 to 1703.24 ) |
| Digestive diseases | Both | 4435.25 ( 4055.78 to 4844.2 ) | 5454.63 ( 4988.69 to 5942.26 ) | 25.58 ( 23.9 to 27.16 ) | 32.07 ( 29.87 to 34.05 ) | 22762.71 ( 21512.27 to 23980.56 ) | 27911.7 ( 26407.85 to 29378.01 ) | 889.92 ( 814.14 to 975.84 ) | 1096.99 ( 1002.19 to 1202.93 ) |
|  | Female | 2362.61 ( 2163.92 to 2581.51 ) | 5733.67 ( 5237.4 to 6253.29 ) | 10.34 ( 9.41 to 11.14 ) | 24.01 ( 21.88 to 25.9 ) | 11054.43 ( 10454.37 to 11647.7 ) | 26735.44 ( 25282.05 to 28176.71 ) | 344.88 ( 309.52 to 387.94 ) | 831.39 ( 743.06 to 935.69 ) |
|  | Male | 2072.64 ( 1889.13 to 2271.47 ) | 5174.07 ( 4728.24 to 5653.95 ) | 15.24 ( 14.17 to 16.37 ) | 40.8 ( 38.02 to 43.82 ) | 11708.28 ( 11060.5 to 12343.77 ) | 29095.9 ( 27493.45 to 30652.71 ) | 545.03 ( 499.02 to 594.75 ) | 1371.07 ( 1254.29 to 1496.2 ) |
| Gynecological diseases | Both | 7738.55 ( 6728.01 to 8763.96 ) | 9649.93 ( 8415.25 to 10928.82 ) | 0.09 ( 0.05 to 0.13 ) | 0.11 ( 0.06 to 0.16 ) | 15270.84 ( 14145.04 to 16432.08 ) | 19025.16 ( 17582 to 20507.56 ) | 297.41 ( 203.77 to 413.4 ) | 367.57 ( 252.66 to 510.83 ) |
|  | Female | 7738.55 ( 6728.01 to 8763.96 ) | 19386.76 ( 16910.21 to 21978.6 ) | 0.09 ( 0.05 to 0.13 ) | 0.2 ( 0.11 to 0.3 ) | 15270.84 ( 14145.04 to 16432.08 ) | 38198.73 ( 35253.23 to 41181.87 ) | 297.41 ( 203.77 to 413.4 ) | 737.37 ( 506.62 to 1025.04 ) |
|  | Male | 0 ( 0 to 0 ) | 0 ( 0 to 0 ) | 0 ( 0 to 0 ) | 0 ( 0 to 0 ) | 0 ( 0 to 0 ) | 0 ( 0 to 0 ) | 0 ( 0 to 0 ) | 0 ( 0 to 0 ) |
| Hemoglobinopathies and hemolytic anemias | Both | 448.96 ( 424.12 to 473.57 ) | 693.2 ( 654.85 to 731.19 ) | 0.93 ( 0.77 to 1.15 ) | 1.25 ( 1.03 to 1.54 ) | 21146.71 ( 20261.42 to 22051.54 ) | 27443.95 ( 26296.66 to 28619.01 ) | 128.16 ( 99.71 to 166.8 ) | 172.59 ( 134.58 to 224.14 ) |
|  | Female | 292.88 ( 279.44 to 305.95 ) | 936.6 ( 893.64 to 978.42 ) | 0.47 ( 0.36 to 0.57 ) | 1.22 ( 0.94 to 1.52 ) | 13947.52 ( 13477.85 to 14409.06 ) | 36393.78 ( 35167.54 to 37602.64 ) | 76.65 ( 57.65 to 101.46 ) | 205.84 ( 154.32 to 270.63 ) |
|  | Male | 156.08 ( 144.65 to 167.81 ) | 465.97 ( 431.84 to 500.97 ) | 0.46 ( 0.36 to 0.62 ) | 1.28 ( 1 to 1.71 ) | 7199.18 ( 6780.6 to 7651.36 ) | 18603.73 ( 17519.04 to 19770.18 ) | 51.5 ( 39.54 to 67.38 ) | 139.89 ( 107.54 to 182.59 ) |
| Mental disorders | Both | 3714.86 ( 3339.7 to 4119.65 ) | 4660.91 ( 4189.44 to 5161.64 ) | 0 ( 0 to 0 ) | 0 ( 0 to 0 ) | 9700.7 ( 9009.16 to 10443.62 ) | 12261.98 ( 11382.9 to 13213.32 ) | 1253.11 ( 929.95 to 1631.78 ) | 1566.24 ( 1160.15 to 2042.81 ) |
|  | Female | 2188.16 ( 1957.3 to 2436.92 ) | 5445.28 ( 4869.79 to 6051.33 ) | 0 ( 0 to 0 ) | 0.01 ( 0.01 to 0.01 ) | 5079.02 ( 4712.25 to 5473.56 ) | 12760.02 ( 11831.71 to 13763.11 ) | 684.61 ( 507.04 to 898.36 ) | 1703.31 ( 1261.49 to 2237.79 ) |
|  | Male | 1526.71 ( 1374.54 to 1685.02 ) | 3862.83 ( 3483.58 to 4251.39 ) | 0 ( 0 to 0 ) | 0 ( 0 to 0 ) | 4621.69 ( 4275.01 to 4997.5 ) | 11727.33 ( 10835.7 to 12693.93 ) | 568.5 ( 420.99 to 744.35 ) | 1426.46 ( 1056.41 to 1869.45 ) |
| Musculoskeletal disorders | Both | 3227.49 ( 2926.71 to 3543.09 ) | 3944.53 ( 3580.13 to 4325.84 ) | 1.18 ( 0.95 to 1.36 ) | 1.51 ( 1.21 to 1.74 ) | 15149.64 ( 14329.31 to 16011.27 ) | 18407.49 ( 17412.57 to 19433.56 ) | 1500.75 ( 1087.82 to 1978.17 ) | 1825.56 ( 1322.52 to 2402 ) |
|  | Female | 1819.86 ( 1655.16 to 1998.73 ) | 4368.03 ( 3972.83 to 4797.58 ) | 0.81 ( 0.58 to 0.98 ) | 1.89 ( 1.35 to 2.29 ) | 8685.48 ( 8247.99 to 9143.07 ) | 20601.03 ( 19534.54 to 21705.72 ) | 887.13 ( 645.26 to 1167.21 ) | 2110.94 ( 1536.35 to 2777.34 ) |
|  | Male | 1407.63 ( 1276.86 to 1548.33 ) | 3505.66 ( 3181.04 to 3848.53 ) | 0.36 ( 0.32 to 0.41 ) | 1.06 ( 0.92 to 1.2 ) | 6464.16 ( 6097.67 to 6853.09 ) | 16121.19 ( 15216.58 to 17073.68 ) | 613.62 ( 442.19 to 810.97 ) | 1526.65 ( 1100.01 to 2016.24 ) |
| Neoplasms | Both | 3050.97 ( 2585.99 to 3646.16 ) | 3763.65 ( 3199.59 to 4473.75 ) | 100.8 ( 94.13 to 106.62 ) | 125.41 ( 117.07 to 132.74 ) | 4854.04 ( 4178.26 to 5618.92 ) | 5959.29 ( 5138.24 to 6883.2 ) | 2513.9 ( 2357.47 to 2657.05 ) | 3062.36 ( 2871.4 to 3237.78 ) |
|  | Female | 1687.72 ( 1416.77 to 2018.54 ) | 4116.48 ( 3461.87 to 4918.36 ) | 43.63 ( 39.95 to 46.87 ) | 100.47 ( 92.01 to 107.93 ) | 2767.08 ( 2372.06 to 3208 ) | 6695.98 ( 5754.87 to 7759.95 ) | 1101.24 ( 1018.21 to 1183.06 ) | 2583.87 ( 2387.31 to 2778.41 ) |
|  | Male | 1363.25 ( 1161.58 to 1623.78 ) | 3457.29 ( 2948.02 to 4109.41 ) | 57.17 ( 52.75 to 61.34 ) | 157.14 ( 144.79 to 168.27 ) | 2086.96 ( 1797.11 to 2415.16 ) | 5287.48 ( 4570.47 to 6108.08 ) | 1412.66 ( 1302.71 to 1519.64 ) | 3624.28 ( 3341.22 to 3891.98 ) |
| Neurological disorders | Both | 8051.78 ( 7258.38 to 8888.47 ) | 10259.5 ( 9223.2 to 11324.16 ) | 22.21 ( 10.28 to 47.6 ) | 30.68 ( 13.83 to 66.32 ) | 26589.32 ( 24528.48 to 28585.29 ) | 33451.93 ( 30870.74 to 36082.04 ) | 977.24 ( 559.43 to 1594.17 ) | 1253.56 ( 719.7 to 2039.81 ) |
|  | Female | 4226.34 ( 3826.79 to 4656.38 ) | 10784.59 ( 9731.93 to 11861.19 ) | 13.25 ( 5.38 to 29.34 ) | 30.28 ( 12.39 to 66.88 ) | 14506.78 ( 13467.6 to 15556.94 ) | 36401.08 ( 33706.3 to 39072.56 ) | 564.63 ( 302.58 to 952.58 ) | 1377.77 ( 725.51 to 2310.39 ) |
|  | Male | 3825.44 ( 3434.06 to 4229.96 ) | 9737.45 ( 8714.78 to 10772.62 ) | 8.96 ( 4.74 to 18.29 ) | 30.77 ( 15.28 to 64.61 ) | 12082.55 ( 11068.89 to 13068.14 ) | 30489.39 ( 28013.6 to 32984.15 ) | 412.61 ( 250.66 to 646.04 ) | 1125.09 ( 696.53 to 1754.77 ) |
| Skin and subcutaneous diseases | Both | 48592.68 ( 46806.93 to 50604.99 ) | 62694.19 ( 60319.5 to 65337.73 ) | 0.99 ( 0.75 to 1.24 ) | 1.3 ( 0.98 to 1.63 ) | 20249.39 ( 19712.39 to 20832.11 ) | 26152.42 ( 25428.78 to 26919.39 ) | 428.84 ( 286.27 to 634.38 ) | 559.42 ( 372.62 to 827.69 ) |
|  | Female | 24128.24 ( 23231.59 to 25090.75 ) | 61816.95 ( 59497.69 to 64330.16 ) | 0.55 ( 0.41 to 0.7 ) | 1.29 ( 0.96 to 1.62 ) | 10496.42 ( 10233.45 to 10782.4 ) | 26959.27 ( 26266.16 to 27732.4 ) | 228.81 ( 153.15 to 337.49 ) | 597.62 ( 398.9 to 879.98 ) |
|  | Male | 24464.43 ( 23495.76 to 25483.51 ) | 63624.18 ( 61120.27 to 66336.08 ) | 0.43 ( 0.27 to 0.6 ) | 1.31 ( 0.82 to 1.82 ) | 9752.97 ( 9473.21 to 10055.73 ) | 25365.88 ( 24612.52 to 26189.41 ) | 200.02 ( 133.64 to 296.9 ) | 522.24 ( 348.03 to 774.48 ) |
| Substance use disorders | Both | 697.29 ( 602.11 to 800.51 ) | 863.26 ( 746.73 to 992.46 ) | 2.96 ( 2.73 to 3.11 ) | 3.59 ( 3.31 to 3.77 ) | 1614.57 ( 1453.37 to 1810.19 ) | 1997.96 ( 1800.27 to 2237 ) | 351.35 ( 282.49 to 429.59 ) | 432.55 ( 347.18 to 529.78 ) |
|  | Female | 195.08 ( 169.38 to 224.89 ) | 486.15 ( 421.4 to 561.1 ) | 0.62 ( 0.59 to 0.65 ) | 1.48 ( 1.41 to 1.55 ) | 469.37 ( 423.4 to 527.35 ) | 1167.64 ( 1050.34 to 1315.05 ) | 104.29 ( 80.53 to 130.65 ) | 257.93 ( 198.75 to 324.12 ) |
|  | Male | 502.2 ( 430.22 to 579.11 ) | 1239.71 ( 1064.99 to 1432.06 ) | 2.34 ( 2.12 to 2.48 ) | 5.75 ( 5.22 to 6.1 ) | 1145.21 ( 1026.47 to 1276.65 ) | 2831.37 ( 2544.57 to 3158.86 ) | 247.06 ( 201.21 to 298.46 ) | 607.58 ( 494.2 to 733.26 ) |
| Urinary diseases and male infertility | Both | 5314.29 ( 4783.03 to 5825.64 ) | 6603.59 ( 5949 to 7235.99 ) | 2.97 ( 2.5 to 3.21 ) | 3.9 ( 3.29 to 4.23 ) | 1569.12 ( 1244.87 to 1975.74 ) | 1928.18 ( 1528.75 to 2432.55 ) | 93.38 ( 79.85 to 108.66 ) | 117.3 ( 100.42 to 136.28 ) |
|  | Female | 3568.69 ( 3191.12 to 3925.65 ) | 8893.05 ( 7942.74 to 9785.08 ) | 1.51 ( 1.25 to 1.66 ) | 3.49 ( 2.89 to 3.84 ) | 72.72 ( 65.17 to 79.91 ) | 180.05 ( 161.24 to 197.96 ) | 34.38 ( 29.16 to 37.65 ) | 83.06 ( 70.59 to 91.05 ) |
|  | Male | 1745.6 ( 1562.99 to 1943.84 ) | 4348.96 ( 3914.77 to 4825.85 ) | 1.46 ( 1.2 to 1.6 ) | 4.47 ( 3.64 to 4.88 ) | 1496.4 ( 1176.35 to 1901.52 ) | 3846.71 ( 3043.68 to 4861.6 ) | 59 ( 48.17 to 72.05 ) | 156.71 ( 128.8 to 190.67 ) |

Table 7 Burden of communicable diseases in 204 countries and regions in 2019.

| **location** | **Incidence** | | **Deaths** | | **Prevalence** | | **DALYs** | |
| --- | --- | --- | --- | --- | --- | --- | --- | --- |
|  | **Count**  **(*10^5^)** | **ASIR**  **(per100,000)** | **Count**  **(*10^5^)** | **ASDR**  **(per100,000)** | **Count**  **(*10^5^)** | **ASPR**  **(per100,000)** | **Count**  **(*10^5^)** | **Age-standardized DALY rate**  **(per100,000)** |
| Afghanistan | 1624.1 ( 1476.17 to 1775.05 ) | 388518.91 ( 358739.9 to 419479.63 ) | 0.41 ( 0.34 to 0.49 ) | 140.38 ( 121.25 to 161.46 ) | 167.13 ( 159.24 to 174.19 ) | 48085.25 ( 45872.99 to 50098.83 ) | 33.25 ( 27.83 to 38.92 ) | 7214.25 ( 6219.81 to 8148.24 ) |
| Albania | 83.15 ( 77.17 to 89.87 ) | 321079.32 ( 296821.69 to 347602.25 ) | 0.01 ( 0 to 0.01 ) | 17.79 ( 15.42 to 19.73 ) | 7.44 ( 7.23 to 7.69 ) | 25161.79 ( 24563.04 to 25785.43 ) | 0.25 ( 0.23 to 0.26 ) | 1077.78 ( 981.78 to 1094.26 ) |
| Algeria | 1494.72 ( 1375.63 to 1620.04 ) | 360255.5 ( 332697.75 to 388183.12 ) | 0.09 ( 0.08 to 0.1 ) | 31.51 ( 27.42 to 36.53 ) | 119.17 ( 117.03 to 122.23 ) | 28130.25 ( 27617.98 to 28751.99 ) | 4.69 ( 4.45 to 4.74 ) | 1219.86 ( 1147.75 to 1234.64 ) |
| American Samoa | 2.01 ( 1.85 to 2.18 ) | 367519.23 ( 340460.34 to 396589.21 ) | 0 ( 0 to 0 ) | 49.46 ( 41.41 to 58.07 ) | 0.24 ( 0.23 to 0.24 ) | 43335.21 ( 42356.87 to 44328.92 ) | 0.01 ( 0.01 to 0.01 ) | 2186.76 ( 1902.64 to 2509.08 ) |
| Andorra | 2.32 ( 2.11 to 2.56 ) | 319588.33 ( 287524.26 to 355466.22 ) | 0 ( 0 to 0 ) | 17.32 ( 12.64 to 26.3 ) | 0.18 ( 0.17 to 0.19 ) | 18771.88 ( 17967.87 to 19462.99 ) | 0.01 ( 0 to 0.01 ) | 563.02 ( 432.14 to 857.54 ) |
| Angola | 1415.35 ( 1282.46 to 1554.14 ) | 450343.34 ( 417278.6 to 486300.04 ) | 0.77 ( 0.65 to 0.92 ) | 392.88 ( 336.68 to 463.03 ) | 150.35 ( 150.85 to 150.59 ) | 54465.16 ( 54577.45 to 54206.11 ) | 50.32 ( 42.69 to 58.89 ) | 16661.41 ( 14131.93 to 19524.68 ) |
| Antigua and Barbuda | 3.12 ( 2.88 to 3.37 ) | 365936.97 ( 336820.32 to 394892.02 ) | 0 ( 0 to 0 ) | 49.04 ( 43.7 to 54.88 ) | 0.4 ( 0.39 to 0.42 ) | 42297.42 ( 40741.42 to 43945.09 ) | 0.01 ( 0.01 to 0.02 ) | 1708.95 ( 1563.97 to 1858.18 ) |
| Argentina | 1542.28 ( 1407.03 to 1693.47 ) | 351878.33 ( 319231.13 to 388257.94 ) | 0.41 ( 0.38 to 0.44 ) | 76.1 ( 70 to 80.84 ) | 170.01 ( 165.79 to 173.78 ) | 36133.85 ( 35239.33 to 36830.55 ) | 9.42 ( 9.02 to 9.62 ) | 1915.02 ( 1840.02 to 1938.32 ) |
| Armenia | 103.39 ( 95.62 to 111.51 ) | 353496.35 ( 326167.45 to 382535.22 ) | 0.01 ( 0.01 to 0.01 ) | 21.02 ( 18.9 to 23.52 ) | 10.1 ( 9.87 to 10.37 ) | 31069.66 ( 30376.65 to 31649.66 ) | 0.36 ( 0.33 to 0.38 ) | 1305.84 ( 1191.11 to 1407.05 ) |
| Australia | 759.71 ( 692.47 to 834.22 ) | 330935.94 ( 297189.17 to 367788.54 ) | 0.05 ( 0.04 to 0.06 ) | 10.76 ( 9.53 to 11.48 ) | 63.75 ( 60.86 to 66.6 ) | 23500.35 ( 22573.7 to 24268.41 ) | 1.15 ( 1.05 to 1.27 ) | 392.54 ( 357.15 to 433.83 ) |
| Austria | 256.29 ( 235.73 to 280.17 ) | 319868.84 ( 289592.23 to 355949.02 ) | 0.02 ( 0.01 to 0.02 ) | 7.66 ( 7.07 to 8.04 ) | 19.11 ( 18.19 to 19.86 ) | 18604.93 ( 17874.34 to 19134.51 ) | 0.42 ( 0.38 to 0.47 ) | 384.19 ( 341.61 to 430.19 ) |
| Azerbaijan | 281.47 ( 259.58 to 304.82 ) | 275378.69 ( 254406.72 to 297709.35 ) | 0.04 ( 0.03 to 0.04 ) | 48.27 ( 41.1 to 56.32 ) | 34.96 ( 34.17 to 35.82 ) | 31904.87 ( 31211.66 to 32706.82 ) | 2.59 ( 2.28 to 2.95 ) | 3167.91 ( 2748.63 to 3660.13 ) |
| Bahamas | 12.95 ( 11.93 to 14.07 ) | 357074.39 ( 329284.35 to 387604.13 ) | 0 ( 0 to 0 ) | 61.37 ( 56.31 to 67.27 ) | 1.57 ( 1.54 to 1.6 ) | 38825.39 ( 38127.12 to 39571.72 ) | 0.1 ( 0.1 to 0.11 ) | 2641.11 ( 2536.85 to 2753.64 ) |
| Bahrain | 48.27 ( 43.96 to 53.12 ) | 365665.08 ( 337705.75 to 396820.1 ) | 0 ( 0 to 0 ) | 28.21 ( 24.16 to 32.08 ) | 4.43 ( 4.33 to 4.55 ) | 27552.7 ( 27035.2 to 28178.19 ) | 0.08 ( 0.08 to 0.09 ) | 858.29 ( 789.82 to 898.75 ) |
| Bangladesh | 6642.9 ( 6137.56 to 7194.48 ) | 420403.58 ( 390184.39 to 453890.38 ) | 1.22 ( 0.98 to 1.6 ) | 102.42 ( 81.26 to 136.07 ) | 650.9 ( 626.38 to 675.06 ) | 39991.64 ( 38415.09 to 41450.25 ) | 58.53 ( 52.09 to 64.96 ) | 4147.54 ( 3683.43 to 4666.01 ) |
| Barbados | 10.92 ( 10.19 to 11.72 ) | 375898.5 ( 349135.14 to 408019.78 ) | 0 ( 0 to 0 ) | 48.47 ( 42.47 to 54.83 ) | 1.33 ( 1.3 to 1.36 ) | 40013.14 ( 39322.28 to 40742.6 ) | 0.06 ( 0.05 to 0.06 ) | 1681.77 ( 1544.85 to 1805.22 ) |
| Belarus | 292.18 ( 270.84 to 315.54 ) | 321700.39 ( 296128.12 to 348356.61 ) | 0.02 ( 0.02 to 0.02 ) | 15.53 ( 13.35 to 18.73 ) | 31.29 ( 30.3 to 32.25 ) | 29257.02 ( 28562.75 to 30018.45 ) | 0.98 ( 0.87 to 1.11 ) | 951.76 ( 852.45 to 1062.73 ) |
| Belgium | 352.95 ( 325.76 to 384.64 ) | 329878.88 ( 299629.59 to 366505.22 ) | 0.08 ( 0.06 to 0.08 ) | 26.53 ( 23.22 to 28.72 ) | 24.85 ( 23.63 to 26.09 ) | 19203.99 ( 18404.61 to 19921.34 ) | 1.18 ( 1.09 to 1.26 ) | 641.31 ( 603.25 to 696.52 ) |
| Belize | 14.67 ( 13.4 to 16.01 ) | 362992.24 ( 334826.71 to 392221.81 ) | 0 ( 0 to 0 ) | 74.3 ( 68.07 to 80.69 ) | 1.64 ( 1.63 to 1.66 ) | 40933.58 ( 40561.11 to 41465.66 ) | 0.11 ( 0.1 to 0.11 ) | 2950.37 ( 2788.53 to 3071.02 ) |
| Benin | 479.78 ( 443.68 to 516.98 ) | 373646.63 ( 349602.87 to 398532 ) | 0.4 ( 0.32 to 0.5 ) | 410.71 ( 328.45 to 515.18 ) | 67.63 ( 67.45 to 67.93 ) | 57512 ( 57142.52 to 57903.49 ) | 28.33 ( 22.8 to 35.01 ) | 18641.44 ( 14994.24 to 22903.7 ) |
| Bermuda | 2.3 ( 2.15 to 2.47 ) | 366777.38 ( 337996.15 to 397413.65 ) | 0 ( 0 to 0 ) | 21.93 ( 19.94 to 24.67 ) | 0.28 ( 0.27 to 0.29 ) | 37265.22 ( 36121.46 to 38309.4 ) | 0.01 ( 0.01 to 0.01 ) | 1026.71 ( 962.35 to 1091.12 ) |
| Bhutan | 28.77 ( 26.76 to 31.16 ) | 387400.6 ( 360457.65 to 417747.27 ) | 0.01 ( 0 to 0.01 ) | 100.86 ( 76.26 to 129.26 ) | 2.26 ( 2.15 to 2.36 ) | 29162.29 ( 27701.57 to 30373.21 ) | 0.28 ( 0.24 to 0.34 ) | 4186.38 ( 3656.18 to 4972.28 ) |
| Bolivia (Plurinational State of) | 447.02 ( 410.94 to 483.97 ) | 365215.97 ( 337627.69 to 394275.04 ) | 0.11 ( 0.09 to 0.14 ) | 124.52 ( 103.66 to 153.19 ) | 48.67 ( 47.95 to 49 ) | 42555.07 ( 41884.04 to 43018.66 ) | 5.27 ( 4.49 to 6.32 ) | 4445.01 ( 3808.31 to 5421.87 ) |
| Bosnia and Herzegovina | 97.4 ( 90.06 to 105.75 ) | 318038.61 ( 292312.58 to 345708.7 ) | 0.01 ( 0 to 0.01 ) | 12.24 ( 10.14 to 14.83 ) | 9.7 ( 9.5 to 9.98 ) | 26041.45 ( 25648.76 to 26388.23 ) | 0.24 ( 0.21 to 0.27 ) | 693.87 ( 620.8 to 758.91 ) |
| Botswana | 86.99 ( 80.13 to 94.18 ) | 377719.86 ( 350871.67 to 406170.92 ) | 0.09 ( 0.08 to 0.1 ) | 466.85 ( 412.45 to 540.28 ) | 12.29 ( 12.13 to 12.36 ) | 52976.48 ( 52355.71 to 53284.34 ) | 4.62 ( 4.07 to 5.52 ) | 20778.15 ( 18485.96 to 24207.04 ) |
| Brazil | 9104.65 ( 8413.96 to 9896.87 ) | 435175.38 ( 399609.18 to 475709.75 ) | 1.32 ( 1.24 to 1.36 ) | 60.24 ( 57.17 to 62.32 ) | 1053.54 ( 1030.51 to 1071.78 ) | 46048.64 ( 45098.56 to 46846.34 ) | 47.97 ( 46.26 to 49.12 ) | 2272.26 ( 2193.72 to 2308.58 ) |
| Brunei Darussalam | 13.64 ( 12.28 to 15.27 ) | 315602.12 ( 283138.88 to 351810.58 ) | 0 ( 0 to 0 ) | 69.97 ( 61.51 to 78.32 ) | 1.52 ( 1.44 to 1.73 ) | 32445.84 ( 30678.37 to 36941.55 ) | 0.05 ( 0.05 to 0.05 ) | 1590.22 ( 1493.26 to 1683.31 ) |
| Bulgaria | 194.56 ( 180.67 to 210.75 ) | 306509.58 ( 281538.17 to 333328.57 ) | 0.02 ( 0.02 to 0.03 ) | 20.36 ( 16.72 to 24.5 ) | 21.93 ( 21.3 to 22.64 ) | 27601.4 ( 27073.88 to 28004.18 ) | 0.75 ( 0.66 to 0.86 ) | 1030.72 ( 908.62 to 1159 ) |
| Burkina Faso | 921.8 ( 849.26 to 993.78 ) | 405120.47 ( 378674.93 to 431673.95 ) | 0.93 ( 0.77 to 1.13 ) | 493.27 ( 423.12 to 576.96 ) | 107.18 ( 102.53 to 108.51 ) | 52430.22 ( 50375.43 to 53078.31 ) | 66.58 ( 55.17 to 82.63 ) | 22924.63 ( 19286.65 to 27134.57 ) |
| Burundi | 418.63 ( 387.21 to 448.78 ) | 343526.1 ( 323028.82 to 366048.52 ) | 0.37 ( 0.3 to 0.45 ) | 474.17 ( 387.22 to 580.21 ) | 72.02 ( 70.59 to 72.76 ) | 63411.36 ( 62304.36 to 64091.38 ) | 24.5 ( 20.21 to 29.42 ) | 19448.48 ( 16094.6 to 23403.07 ) |
| Cabo Verde | 18.68 ( 17.29 to 20.16 ) | 337591.41 ( 313489.97 to 362960.6 ) | 0.01 ( 0 to 0.01 ) | 114.72 ( 102.63 to 131.71 ) | 2.09 ( 2.08 to 2.11 ) | 37228 ( 36966.2 to 37621.64 ) | 0.19 ( 0.18 to 0.22 ) | 3843.92 ( 3532.21 to 4199.63 ) |
| Cambodia | 494.25 ( 453.53 to 537.83 ) | 302079.42 ( 279041.75 to 326992.7 ) | 0.23 ( 0.2 to 0.26 ) | 205.25 ( 179.82 to 225.75 ) | 73.01 ( 71.05 to 75 ) | 44285.47 ( 43150.32 to 45436.68 ) | 10.32 ( 9.16 to 11.7 ) | 6917.62 ( 6221.78 to 7711.5 ) |
| Cameroon | 1275.44 ( 1171.02 to 1384.72 ) | 437504.45 ( 407258.09 to 469943 ) | 0.99 ( 0.82 to 1.21 ) | 476.01 ( 393.14 to 588.86 ) | 155.15 ( 152.05 to 157.75 ) | 56964.53 ( 55943.21 to 57857.51 ) | 64.31 ( 53.71 to 76.1 ) | 22090.34 ( 18634.32 to 26493.82 ) |
| Canada | 1303.38 ( 1193.35 to 1426.56 ) | 388278.56 ( 352720.39 to 428498.72 ) | 0.12 ( 0.1 to 0.13 ) | 15.65 ( 13.77 to 16.8 ) | 94.6 ( 90.53 to 98.55 ) | 23047.31 ( 22192.17 to 23883.45 ) | 2.6 ( 2.33 to 2.91 ) | 556.29 ( 501.12 to 629.84 ) |
| Central African Republic | 282.12 ( 257.45 to 309.61 ) | 511193.83 ( 473307.82 to 552906.27 ) | 0.36 ( 0.3 to 0.44 ) | 964.02 ( 794.05 to 1168.26 ) | 30.29 ( 30.51 to 30.57 ) | 60262.74 ( 60773.46 to 60571.46 ) | 23.49 ( 19.7 to 28.58 ) | 42509.12 ( 35604.37 to 51093.23 ) |
| Chad | 718.94 ( 662.82 to 774.15 ) | 431544.17 ( 404474.51 to 459089.9 ) | 0.85 ( 0.73 to 0.99 ) | 565.21 ( 495.74 to 658.21 ) | 78.77 ( 78.52 to 79.61 ) | 54089.73 ( 53754.85 to 54689.51 ) | 63.73 ( 54.44 to 75.05 ) | 27296.53 ( 23955.11 to 31645.65 ) |
| Chile | 614.73 ( 561.06 to 675.94 ) | 355855.86 ( 323369.77 to 393765.23 ) | 0.07 ( 0.06 to 0.07 ) | 28.73 ( 26.2 to 30.39 ) | 68.87 ( 65.92 to 72.03 ) | 34948.93 ( 33423.14 to 36543.36 ) | 1.81 ( 1.69 to 1.9 ) | 903.13 ( 843.72 to 951.57 ) |
| China | 35761.71 ( 33140.59 to 38925.74 ) | 263227.93 ( 241161.9 to 287276.37 ) | 2.87 ( 2.58 to 3.18 ) | 20.17 ( 18.52 to 22.2 ) | 6511.59 ( 6300.31 to 6710.77 ) | 41614.79 ( 40163.77 to 43287.16 ) | 121.9 ( 111.24 to 130.36 ) | 922.26 ( 851.91 to 985.29 ) |
| Colombia | 1901.54 ( 1738.31 to 2087.79 ) | 407989.99 ( 372401.97 to 448319.21 ) | 0.14 ( 0.12 to 0.16 ) | 27.25 ( 22.74 to 32.12 ) | 247.76 ( 238.44 to 257.2 ) | 50395.03 ( 48445.86 to 52384.2 ) | 6.75 ( 5.96 to 7.71 ) | 1450.55 ( 1271.04 to 1668.55 ) |
| Comoros | 28.63 ( 26.27 to 31.92 ) | 403543.97 ( 373328.66 to 445634.1 ) | 0.01 ( 0.01 to 0.02 ) | 266.69 ( 227.6 to 341.11 ) | 3.07 ( 3.03 to 3.52 ) | 44356.86 ( 43850.38 to 50622.3 ) | 0.71 ( 0.62 to 0.83 ) | 10588.35 ( 9245.4 to 12494.76 ) |
| Congo | 232.85 ( 212.39 to 253.43 ) | 435586.43 ( 402998.94 to 471187.3 ) | 0.13 ( 0.11 to 0.16 ) | 375.14 ( 317.86 to 445.84 ) | 24.48 ( 24.55 to 25.06 ) | 48395.3 ( 48767.61 to 49038.19 ) | 7.62 ( 6.66 to 8.87 ) | 15500.92 ( 13489.08 to 18075.03 ) |
| Cook Islands | 0.69 ( 0.64 to 0.74 ) | 378719.31 ( 349333.43 to 408912.74 ) | 0 ( 0 to 0 ) | 59.34 ( 46.48 to 89.55 ) | 0.08 ( 0.08 to 0.08 ) | 42094.5 ( 41462.64 to 42667.66 ) | 0 ( 0 to 0.01 ) | 2138.68 ( 1659.66 to 3417.9 ) |
| Costa Rica | 155.59 ( 143.35 to 169.44 ) | 340206.98 ( 312934.33 to 371143.47 ) | 0.01 ( 0.01 to 0.01 ) | 21.46 ( 17.78 to 25.78 ) | 23 ( 22.14 to 23.74 ) | 46826.77 ( 45018.22 to 48366.81 ) | 0.47 ( 0.41 to 0.52 ) | 997.48 ( 893.02 to 1114.83 ) |
| Croatia | 117.18 ( 108.98 to 126.93 ) | 297463.32 ( 273397.33 to 325177.35 ) | 0.01 ( 0.01 to 0.01 ) | 8.31 ( 6.74 to 9.93 ) | 11.57 ( 11.33 to 11.84 ) | 24040.07 ( 23571.5 to 24470.89 ) | 0.24 ( 0.21 to 0.27 ) | 472.84 ( 417.15 to 500.9 ) |
| Cuba | 412.3 ( 384 to 441.35 ) | 367654.09 ( 339175.56 to 395759.55 ) | 0.08 ( 0.07 to 0.09 ) | 41.16 ( 34.71 to 48.01 ) | 49.18 ( 48.09 to 50.27 ) | 38588.9 ( 37814.75 to 39438.96 ) | 1.8 ( 1.61 to 2.01 ) | 1255.02 ( 1147.06 to 1370.72 ) |
| Cyprus | 39.35 ( 35.95 to 43.34 ) | 326054.62 ( 295082.17 to 362198.92 ) | 0 ( 0 to 0 ) | 14.27 ( 12.75 to 15.87 ) | 2.87 ( 2.72 to 3 ) | 19219.83 ( 18411.95 to 19942.01 ) | 0.06 ( 0.05 to 0.06 ) | 426.74 ( 390.16 to 443.39 ) |
| Czechia | 312.86 ( 290.08 to 337.12 ) | 307750.3 ( 283844.27 to 333212.68 ) | 0.04 ( 0.03 to 0.05 ) | 18.57 ( 15.72 to 21.97 ) | 27.68 ( 26.89 to 28.51 ) | 23195.46 ( 22681.47 to 23647.6 ) | 0.93 ( 0.82 to 1.04 ) | 658.34 ( 583.58 to 726.99 ) |
| Democratic People's Republic of Korea | 726.04 ( 673.65 to 788.55 ) | 286985.82 ( 264963.77 to 312142.71 ) | 0.12 ( 0.1 to 0.16 ) | 46.31 ( 38.94 to 56.93 ) | 129.94 ( 125.79 to 133.07 ) | 46601.13 ( 44983.47 to 47816.85 ) | 4.74 ( 4.07 to 5.86 ) | 1887.05 ( 1673.58 to 2248.42 ) |
| Democratic Republic of the Congo | 3857.85 ( 3524 to 4187.73 ) | 424588.49 ( 394289.95 to 456489.11 ) | 2.3 ( 1.96 to 2.68 ) | 391.14 ( 322.62 to 465.43 ) | 456.17 ( 467.38 to 443.3 ) | 53843.31 ( 55492.17 to 52206.7 ) | 157.56 ( 137.98 to 180.23 ) | 17533.73 ( 15344.54 to 19914.92 ) |
| Denmark | 178.03 ( 164.23 to 194.04 ) | 329029.44 ( 298034.52 to 363301.38 ) | 0.03 ( 0.03 to 0.03 ) | 23.03 ( 20.32 to 24.96 ) | 13.26 ( 12.61 to 13.82 ) | 20155.73 ( 19410.17 to 20690.08 ) | 0.49 ( 0.46 to 0.52 ) | 558.08 ( 526.94 to 600.97 ) |
| Djibouti | 45.03 ( 41.35 to 49.09 ) | 375088.59 ( 347233.76 to 405661.04 ) | 0.03 ( 0.02 to 0.03 ) | 323.37 ( 276.91 to 386.38 ) | 3.8 ( 3.93 to 3.69 ) | 32826.82 ( 33827.84 to 31657.31 ) | 1.52 ( 1.29 to 1.83 ) | 13032.26 ( 11040.59 to 15585.97 ) |
| Dominica | 2.42 ( 2.24 to 2.61 ) | 360738.24 ( 331857.93 to 390494.86 ) | 0 ( 0 to 0 ) | 55.82 ( 47.53 to 65.28 ) | 0.29 ( 0.29 to 0.3 ) | 40333.21 ( 39732.38 to 40907.65 ) | 0.02 ( 0.01 to 0.02 ) | 2354.79 ( 2065.92 to 2663.22 ) |
| Dominican Republic | 385.65 ( 355.95 to 417.38 ) | 357293.93 ( 329752.15 to 385992.66 ) | 0.06 ( 0.05 to 0.06 ) | 57.31 ( 48.18 to 66.29 ) | 51.78 ( 50.51 to 53.18 ) | 47510.37 ( 46327.55 to 48851.06 ) | 3.13 ( 2.7 to 3.56 ) | 2928.2 ( 2525.4 to 3328.83 ) |
| Ecuador | 647.01 ( 593.33 to 703.3 ) | 369536.86 ( 339850.45 to 400488.87 ) | 0.09 ( 0.08 to 0.1 ) | 61.66 ( 55.16 to 70.57 ) | 79.99 ( 76 to 84.22 ) | 45824.58 ( 43542.45 to 48163.82 ) | 3.91 ( 3.52 to 4.32 ) | 2355.2 ( 2113.66 to 2601.44 ) |
| Egypt | 3218.85 ( 2968.44 to 3497.39 ) | 325449.03 ( 302394.56 to 351657.47 ) | 0.36 ( 0.28 to 0.46 ) | 49.85 ( 38.94 to 61.82 ) | 384.41 ( 375.5 to 392.39 ) | 40574.22 ( 39557.08 to 41375.21 ) | 24.12 ( 19.94 to 28.97 ) | 2557.44 ( 2120.88 to 3058.19 ) |
| El Salvador | 222.29 ( 203.31 to 241.77 ) | 358056.43 ( 329077.72 to 387660.52 ) | 0.04 ( 0.03 to 0.04 ) | 56.64 ( 45.4 to 69.4 ) | 32 ( 30.92 to 33.13 ) | 51143.93 ( 49465.74 to 52940.65 ) | 1.36 ( 1.16 to 1.55 ) | 2246.97 ( 1912.35 to 2579.89 ) |
| Equatorial Guinea | 61.52 ( 55.38 to 67.75 ) | 428251.24 ( 393528.13 to 463138.38 ) | 0.04 ( 0.03 to 0.05 ) | 434.63 ( 330.39 to 576.4 ) | 7.74 ( 7.54 to 7.76 ) | 57582.53 ( 56763.99 to 57327.42 ) | 2.44 ( 1.92 to 3.15 ) | 19980.62 ( 15592.55 to 26031.62 ) |
| Eritrea | 270.05 ( 246.99 to 291.82 ) | 405926.54 ( 377634.65 to 432902.08 ) | 0.17 ( 0.13 to 0.22 ) | 464.75 ( 362.88 to 591.35 ) | 29.4 ( 29.14 to 29.6 ) | 46687.55 ( 46218.14 to 47017.21 ) | 9.48 ( 7.55 to 12.53 ) | 16622.92 ( 13324.86 to 21472.77 ) |
| Estonia | 42.03 ( 39.03 to 45.25 ) | 337243.63 ( 310651.12 to 365478.57 ) | 0 ( 0 to 0 ) | 14.11 ( 12.24 to 16.34 ) | 4.23 ( 4.09 to 4.36 ) | 28780.49 ( 27867.62 to 29533.61 ) | 0.13 ( 0.11 to 0.14 ) | 870.36 ( 772.35 to 965.13 ) |
| Eswatini | 42.52 ( 39.14 to 45.91 ) | 375216.68 ( 348579.68 to 401999.12 ) | 0.05 ( 0.05 to 0.06 ) | 627.75 ( 542.88 to 725.02 ) | 6.41 ( 6.35 to 6.48 ) | 59452.73 ( 58823.75 to 59942.25 ) | 3.07 ( 2.71 to 3.49 ) | 29593.15 ( 26263.19 to 33492.05 ) |
| Ethiopia | 3458.73 ( 3166.1 to 3772.74 ) | 324362.14 ( 301810.41 to 348672.51 ) | 2.03 ( 1.82 to 2.28 ) | 302.19 ( 276.32 to 330.13 ) | 561.77 ( 558.38 to 560.88 ) | 54669.9 ( 54911.56 to 53921.46 ) | 130.56 ( 115.79 to 148.9 ) | 12212.38 ( 11145.41 to 13392.1 ) |
| Fiji | 33.31 ( 30.83 to 36 ) | 370451.37 ( 343455.77 to 399375.09 ) | 0 ( 0 to 0.01 ) | 73.96 ( 58.92 to 90.79 ) | 4.28 ( 4.16 to 4.5 ) | 46850.02 ( 45509.83 to 49281.06 ) | 0.28 ( 0.24 to 0.33 ) | 3372.29 ( 2861.99 to 3863.03 ) |
| Finland | 157.33 ( 144.77 to 172.46 ) | 317058.73 ( 287605.64 to 351781.14 ) | 0.01 ( 0.01 to 0.01 ) | 6.88 ( 6.31 to 7.41 ) | 11.81 ( 11.26 to 12.37 ) | 18786.14 ( 18061.04 to 19440.29 ) | 0.23 ( 0.21 to 0.26 ) | 322.12 ( 289.24 to 378.88 ) |
| France | 1922.14 ( 1767.36 to 2101.38 ) | 315826.66 ( 285787.14 to 351709.96 ) | 0.28 ( 0.23 to 0.31 ) | 14.92 ( 13.02 to 16.01 ) | 141.36 ( 134.46 to 148.74 ) | 19156.06 ( 18267.3 to 19771.78 ) | 4.46 ( 4.15 to 4.82 ) | 447.54 ( 417.44 to 489.21 ) |
| Gabon | 77.34 ( 70.25 to 84.32 ) | 441101.73 ( 404502.25 to 476716.06 ) | 0.04 ( 0.03 to 0.04 ) | 281.76 ( 235.38 to 339 ) | 9.34 ( 9.34 to 9.49 ) | 54510.55 ( 54584.13 to 55135.55 ) | 1.9 ( 1.64 to 2.25 ) | 11846.25 ( 10245.74 to 13806.99 ) |
| Gambia | 67.61 ( 62.63 to 72.99 ) | 313333.02 ( 292533.37 to 334696.28 ) | 0.05 ( 0.04 to 0.06 ) | 378.82 ( 324.75 to 460.36 ) | 7.99 ( 7.94 to 8.05 ) | 39740.93 ( 39546.8 to 40112.01 ) | 2.63 ( 2.29 to 3.04 ) | 13844.99 ( 12132.25 to 16251.01 ) |
| Georgia | 97.9 ( 90.75 to 105.56 ) | 280865.26 ( 259362.47 to 303291.24 ) | 0.01 ( 0.01 to 0.01 ) | 20.49 ( 18.85 to 22.06 ) | 12.29 ( 12.03 to 12.56 ) | 31376.02 ( 30703.73 to 32182.02 ) | 0.45 ( 0.42 to 0.46 ) | 1163.86 ( 1122.22 to 1179.88 ) |
| Germany | 2540.72 ( 2350.12 to 2758.07 ) | 326675.23 ( 295376.67 to 362719.77 ) | 0.32 ( 0.28 to 0.35 ) | 14.37 ( 13.11 to 15.49 ) | 206.5 ( 195.18 to 218.34 ) | 20652.89 ( 19684.34 to 21644.45 ) | 6.17 ( 5.74 to 6.74 ) | 472.15 ( 430.25 to 521.46 ) |
| Ghana | 1149.13 ( 1061.16 to 1242.43 ) | 370370.07 ( 345552.84 to 395404.79 ) | 0.79 ( 0.69 to 0.89 ) | 360.31 ( 318.1 to 413.03 ) | 162.41 ( 160.01 to 163.65 ) | 53261.46 ( 52650.79 to 53834.06 ) | 44.82 ( 39.14 to 51.09 ) | 15163.03 ( 13439.13 to 17001.88 ) |
| Greece | 294.34 ( 269.9 to 322.13 ) | 322066.02 ( 291227.57 to 358155.27 ) | 0.06 ( 0.05 to 0.07 ) | 20.58 ( 18.23 to 22.1 ) | 22.08 ( 21.27 to 22.8 ) | 18613.15 ( 18003.46 to 19039.25 ) | 0.97 ( 0.91 to 1.05 ) | 558.58 ( 515.56 to 605.18 ) |
| Greenland | 2.05 ( 1.88 to 2.25 ) | 382177.82 ( 347486.27 to 422818.36 ) | 0 ( 0 to 0 ) | 38.79 ( 32.68 to 44.43 ) | 0.18 ( 0.18 to 0.19 ) | 29366.97 ( 28530.5 to 30178.11 ) | 0.01 ( 0.01 to 0.01 ) | 1200.02 ( 1069.54 to 1341.99 ) |
| Grenada | 3.81 ( 3.53 to 4.11 ) | 380524.64 ( 351168.91 to 412482.72 ) | 0 ( 0 to 0 ) | 63.24 ( 57.64 to 67.91 ) | 0.45 ( 0.44 to 0.45 ) | 40821.92 ( 39979.65 to 41461.03 ) | 0.02 ( 0.02 to 0.02 ) | 2038.63 ( 1927.66 to 2072.46 ) |
| Guam | 6.61 ( 6.13 to 7.1 ) | 386071.96 ( 357696.53 to 414237.37 ) | 0 ( 0 to 0 ) | 34.6 ( 29.98 to 40.64 ) | 0.74 ( 0.73 to 0.75 ) | 43053.65 ( 42288.98 to 43642.09 ) | 0.03 ( 0.03 to 0.04 ) | 1914.59 ( 1745.38 to 2104.43 ) |
| Guatemala | 603.54 ( 552.73 to 658.77 ) | 337328.11 ( 311817.69 to 364314.33 ) | 0.15 ( 0.12 to 0.18 ) | 124.2 ( 102.22 to 149.94 ) | 93.64 ( 90.66 to 96.43 ) | 54193.92 ( 52778.2 to 55575.04 ) | 7.13 ( 5.77 to 8.73 ) | 4358.07 ( 3555.82 to 5306.24 ) |
| Guinea | 482.35 ( 443.49 to 522.08 ) | 383745.98 ( 357861.18 to 409944.2 ) | 0.51 ( 0.42 to 0.61 ) | 501.69 ( 413.5 to 599.85 ) | 67.45 ( 66.89 to 68.3 ) | 57000.4 ( 56633.99 to 57626.62 ) | 34.63 ( 28.62 to 41.01 ) | 23109.4 ( 19163.27 to 27266.16 ) |
| Guinea-Bissau | 70.59 ( 64.65 to 76.87 ) | 379086.21 ( 352698.58 to 405493.02 ) | 0.06 ( 0.05 to 0.07 ) | 498.58 ( 418.59 to 604.97 ) | 9.43 ( 9.36 to 9.53 ) | 52921.05 ( 52465.04 to 53718.78 ) | 3.79 ( 3.22 to 4.5 ) | 20622.92 ( 17452.41 to 24676.39 ) |
| Guyana | 27.56 ( 25.31 to 30.01 ) | 364629.77 ( 336052.66 to 394289.53 ) | 0.01 ( 0.01 to 0.01 ) | 101.05 ( 85.93 to 118.03 ) | 3.65 ( 3.46 to 4.03 ) | 47157.24 ( 44738.68 to 51962.03 ) | 0.32 ( 0.29 to 0.36 ) | 4446.8 ( 3969.26 to 4986.95 ) |
| Haiti | 516.81 ( 474.93 to 564.2 ) | 414619.86 ( 383525.6 to 449557.63 ) | 0.21 ( 0.19 to 0.24 ) | 208.45 ( 179.59 to 239.52 ) | 58.52 ( 57.87 to 59.06 ) | 48547.72 ( 48135.2 to 48960.93 ) | 14.09 ( 12.54 to 15.64 ) | 10819.88 ( 9693.34 to 11978.14 ) |
| Honduras | 361.39 ( 330.15 to 395.91 ) | 363401.8 ( 334340.29 to 394696.14 ) | 0.04 ( 0.03 to 0.05 ) | 57.51 ( 49.22 to 69.53 ) | 55.19 ( 53.53 to 56.77 ) | 57742.93 ( 56073.13 to 59187.93 ) | 2.23 ( 1.88 to 2.56 ) | 2442.82 ( 2124.39 to 2835.96 ) |
| Hungary | 296.69 ( 275.31 to 320.34 ) | 323563.62 ( 298458.4 to 351001.51 ) | 0.02 ( 0.01 to 0.02 ) | 9.55 ( 8.29 to 11.25 ) | 26.38 ( 25.66 to 27.01 ) | 24156.02 ( 23674.86 to 24526.77 ) | 0.68 ( 0.61 to 0.76 ) | 614.54 ( 559.07 to 677.58 ) |
| Iceland | 10.5 ( 9.61 to 11.58 ) | 324943.45 ( 293859.16 to 360933.31 ) | 0 ( 0 to 0 ) | 16.73 ( 14.22 to 18.86 ) | 0.73 ( 0.7 to 0.76 ) | 19344.3 ( 18489.64 to 20131.8 ) | 0.02 ( 0.02 to 0.02 ) | 486.86 ( 448.57 to 535.02 ) |
| India | 48376.28 ( 45098.67 to 51981.01 ) | 353056.12 ( 329684.9 to 378252.34 ) | 18.41 ( 15.85 to 21.92 ) | 179.26 ( 151.56 to 216.61 ) | 4798.18 ( 4667.19 to 4955.47 ) | 33975.09 ( 33149.86 to 35061.03 ) | 807.7 ( 754.33 to 868.3 ) | 6617.76 ( 6187.89 to 7154.87 ) |
| Indonesia | 9476.79 ( 8721.64 to 10408.94 ) | 377526.18 ( 348781.66 to 412564.45 ) | 2.29 ( 2 to 2.57 ) | 128.57 ( 111.12 to 145.58 ) | 1274.16 ( 1235.51 to 1322.53 ) | 47810.39 ( 46411.61 to 49704.57 ) | 102.18 ( 94.3 to 108.15 ) | 4703.25 ( 4335.77 to 5031.49 ) |
| Iran (Islamic Republic of) | 3179.07 ( 2942.01 to 3450.88 ) | 387172.55 ( 357770.01 to 418210.08 ) | 0.16 ( 0.15 to 0.16 ) | 23.79 ( 22.59 to 24.78 ) | 354.46 ( 341.79 to 368.03 ) | 39928 ( 38549.89 to 41268.79 ) | 7.83 ( 7.38 to 8.16 ) | 1020.04 ( 968.39 to 1058.45 ) |
| Iraq | 1542.74 ( 1403.47 to 1699.33 ) | 362443.41 ( 333558.32 to 394567.34 ) | 0.07 ( 0.06 to 0.08 ) | 25.18 ( 22.43 to 28.81 ) | 149.68 ( 144.72 to 154.78 ) | 36755.62 ( 35624.33 to 37774.3 ) | 5.9 ( 5.28 to 6.54 ) | 1464.47 ( 1332.99 to 1595.27 ) |
| Ireland | 145.51 ( 132.33 to 160.5 ) | 315984.04 ( 284699.73 to 353145.78 ) | 0.02 ( 0.02 to 0.02 ) | 23.84 ( 20.55 to 26.2 ) | 10.1 ( 9.64 to 10.61 ) | 18761.95 ( 17979.1 to 19551.51 ) | 0.31 ( 0.29 to 0.33 ) | 507.68 ( 471.75 to 540.54 ) |
| Israel | 301.96 ( 273.96 to 334.54 ) | 323938.86 ( 292684.71 to 359956.78 ) | 0.02 ( 0.02 to 0.03 ) | 18.05 ( 16.14 to 19.5 ) | 18.28 ( 17.72 to 18.79 ) | 19346.08 ( 18775 to 19874.96 ) | 0.57 ( 0.53 to 0.62 ) | 536.95 ( 495.75 to 589.97 ) |
| Italy | 1697.72 ( 1563.96 to 1857.62 ) | 325879.17 ( 294082.98 to 362457.73 ) | 0.16 ( 0.13 to 0.17 ) | 9.11 ( 8.37 to 9.48 ) | 137.34 ( 132.53 to 141.88 ) | 19531.31 ( 19037.52 to 19923.15 ) | 3.16 ( 2.87 to 3.46 ) | 385.99 ( 348.24 to 438.97 ) |
| Jamaica | 96.1 ( 88.5 to 104.77 ) | 350784.76 ( 322661.27 to 382350.84 ) | 0.01 ( 0.01 to 0.01 ) | 33.46 ( 30.23 to 37.9 ) | 11.89 ( 11.72 to 12.08 ) | 40417.58 ( 39844.38 to 41002.14 ) | 0.46 ( 0.45 to 0.48 ) | 1656.55 ( 1593.98 to 1698.98 ) |
| Japan | 3662.64 ( 3358.95 to 4010.25 ) | 328555.67 ( 295870.09 to 367569.39 ) | 1.29 ( 1.02 to 1.45 ) | 24.88 ( 20.59 to 27.28 ) | 364.8 ( 353.14 to 380.59 ) | 24860.51 ( 24156.97 to 25567.92 ) | 16.16 ( 14.3 to 17.3 ) | 561.07 ( 517 to 622.32 ) |
| Jordan | 337.58 ( 311.07 to 367.76 ) | 292178.82 ( 271600.08 to 316118.19 ) | 0.02 ( 0.01 to 0.02 ) | 23.51 ( 20.41 to 26.9 ) | 24.79 ( 23.71 to 25.84 ) | 22116.06 ( 21150.76 to 23009.36 ) | 1.13 ( 1.01 to 1.27 ) | 1085.15 ( 972.9 to 1210.25 ) |
| Kazakhstan | 537.11 ( 495.54 to 584.97 ) | 292918.67 ( 270167.37 to 319259.78 ) | 0.06 ( 0.05 to 0.06 ) | 33.35 ( 31.17 to 35.7 ) | 55.91 ( 55.09 to 56.75 ) | 29653.81 ( 29267.82 to 30012.58 ) | 3.04 ( 2.9 to 3.1 ) | 1651.47 ( 1588.22 to 1677.39 ) |
| Kenya | 2342.05 ( 2135.33 to 2577.95 ) | 465786.47 ( 431210.98 to 504359 ) | 1.32 ( 1.21 to 1.44 ) | 417.77 ( 378.26 to 463.06 ) | 268.72 ( 261.5 to 275.72 ) | 56380.11 ( 55117.47 to 57632.92 ) | 74.56 ( 67.93 to 80.88 ) | 17008.54 ( 15685.34 to 18404.13 ) |
| Kiribati | 4.65 ( 4.27 to 5.05 ) | 399861.86 ( 370596.99 to 431863.5 ) | 0 ( 0 to 0 ) | 270.45 ( 223.89 to 332.92 ) | 0.58 ( 0.57 to 0.59 ) | 50245.71 ( 49528.06 to 50792.87 ) | 0.1 ( 0.09 to 0.11 ) | 9338.78 ( 8289.61 to 10411.43 ) |
| Kuwait | 149.49 ( 136.99 to 164.25 ) | 358156.38 ( 330275.68 to 388357.71 ) | 0.01 ( 0.01 to 0.01 ) | 38.2 ( 31.69 to 44.76 ) | 11.47 ( 11.19 to 11.77 ) | 23401.01 ( 22850.41 to 23877.31 ) | 0.3 ( 0.27 to 0.34 ) | 1002.7 ( 897.47 to 1078.62 ) |
| Kyrgyzstan | 164.38 ( 152.14 to 178.5 ) | 246711.55 ( 228220.65 to 267610.24 ) | 0.02 ( 0.01 to 0.02 ) | 27.24 ( 25.32 to 29.05 ) | 21.18 ( 20.76 to 21.63 ) | 32941.35 ( 32172.56 to 33743.16 ) | 1.2 ( 1.15 to 1.2 ) | 1789.43 ( 1706.48 to 1788.14 ) |
| Lao People's Democratic Republic | 240.66 ( 218.71 to 264.58 ) | 337527.92 ( 309720.9 to 367507.4 ) | 0.08 ( 0.06 to 0.1 ) | 168.65 ( 139.15 to 207.59 ) | 31.18 ( 30.17 to 32.3 ) | 43563.32 ( 42273.21 to 45063.78 ) | 4.54 ( 3.66 to 5.61 ) | 6751.25 ( 5530.36 to 8290.54 ) |
| Latvia | 61.27 ( 56.88 to 65.93 ) | 339011.19 ( 311207.01 to 367971.9 ) | 0.01 ( 0.01 to 0.01 ) | 19.3 ( 17.57 to 21.53 ) | 6.4 ( 6.22 to 6.6 ) | 29803.13 ( 28976.94 to 30491.64 ) | 0.24 ( 0.22 to 0.26 ) | 1110.18 ( 1017.93 to 1205.63 ) |
| Lebanon | 191.18 ( 177 to 207.18 ) | 370966.91 ( 342838.41 to 401704.41 ) | 0.02 ( 0.01 to 0.02 ) | 33.29 ( 28.83 to 39.92 ) | 14.99 ( 14.48 to 15.48 ) | 28343.09 ( 27495.34 to 29207.84 ) | 0.65 ( 0.57 to 0.71 ) | 1255.53 ( 1110.38 to 1374.16 ) |
| Lesotho | 80.17 ( 74.25 to 86.61 ) | 388088.77 ( 361457.8 to 416152.44 ) | 0.17 ( 0.15 to 0.2 ) | 1022.4 ( 894.37 to 1166.84 ) | 10.71 ( 10.55 to 10.79 ) | 52830.3 ( 52123.13 to 53040.48 ) | 8.86 ( 7.81 to 10.18 ) | 45882.2 ( 40728.23 to 51933.48 ) |
| Liberia | 194.11 ( 178.41 to 210.8 ) | 406858.78 ( 378506.29 to 436697.36 ) | 0.14 ( 0.11 to 0.16 ) | 418.28 ( 348.15 to 506.4 ) | 33.34 ( 33.33 to 33.57 ) | 71328.74 ( 71361.53 to 71829.28 ) | 9.17 ( 7.74 to 11.05 ) | 19921.23 ( 17221.82 to 23492.85 ) |
| Libya | 240.71 ( 220.82 to 261.56 ) | 374869.72 ( 345835.16 to 406777.51 ) | 0.01 ( 0.01 to 0.02 ) | 27.26 ( 22.81 to 33.05 ) | 25.84 ( 24.93 to 26.93 ) | 36378.32 ( 35381.17 to 37715.09 ) | 0.74 ( 0.64 to 0.87 ) | 1274.63 ( 1132.28 to 1434.31 ) |
| Lithuania | 89.77 ( 83.05 to 96.54 ) | 337721.82 ( 310454.69 to 365099.93 ) | 0.01 ( 0.01 to 0.01 ) | 17.48 ( 15.46 to 20.22 ) | 9.42 ( 9.17 to 9.69 ) | 29963.08 ( 29214.49 to 30651.94 ) | 0.32 ( 0.28 to 0.36 ) | 977.5 ( 878.23 to 1078.67 ) |
| Luxembourg | 18.52 ( 16.91 to 20.35 ) | 326317.55 ( 294673.82 to 362159.16 ) | 0 ( 0 to 0 ) | 15.4 ( 13.04 to 17.41 ) | 1.33 ( 1.26 to 1.39 ) | 18814.28 ( 18010.16 to 19515.97 ) | 0.04 ( 0.03 to 0.04 ) | 484.46 ( 428.15 to 525.23 ) |
| Madagascar | 1020.12 ( 940.61 to 1110.98 ) | 380357.07 ( 354907.88 to 408906.28 ) | 0.64 ( 0.54 to 0.75 ) | 357.73 ( 299.65 to 429.65 ) | 120.99 ( 120.58 to 121.66 ) | 48373.78 ( 48192.49 to 48505.9 ) | 42.26 ( 36.75 to 48.71 ) | 15296.34 ( 13232.73 to 17702.37 ) |
| Malawi | 796.62 ( 730.33 to 866.24 ) | 428200.55 ( 398939.6 to 458388.56 ) | 0.53 ( 0.47 to 0.6 ) | 455.74 ( 402.89 to 514.26 ) | 80.44 ( 80.52 to 80.61 ) | 48115.19 ( 48085.52 to 48301.21 ) | 32.57 ( 28.7 to 37.36 ) | 19549.3 ( 17545.22 to 21873.12 ) |
| Malaysia | 1044.44 ( 956.41 to 1142.04 ) | 343529.59 ( 316213.38 to 374355.72 ) | 0.29 ( 0.21 to 0.35 ) | 128.41 ( 93.2 to 156.15 ) | 130.45 ( 126.91 to 134.57 ) | 40586.74 ( 39624.45 to 41767.08 ) | 8.93 ( 7.63 to 10.18 ) | 3255.99 ( 2776.66 to 3725.31 ) |
| Maldives | 15.91 ( 14.48 to 17.51 ) | 330308.78 ( 303424.03 to 359643.76 ) | 0 ( 0 to 0 ) | 30.21 ( 27.87 to 32.42 ) | 2.13 ( 2.07 to 2.19 ) | 40675.4 ( 39615.33 to 41769.2 ) | 0.05 ( 0.05 to 0.05 ) | 1286.01 ( 1236.5 to 1325.7 ) |
| Mali | 793.36 ( 734.93 to 861.16 ) | 364047.24 ( 340667.18 to 389986.58 ) | 0.8 ( 0.65 to 0.96 ) | 402.54 ( 331 to 494.36 ) | 93.62 ( 93.55 to 96.88 ) | 48837.14 ( 48767.24 to 50200.49 ) | 58.66 ( 49.08 to 70.71 ) | 19835.28 ( 16523.51 to 23879.29 ) |
| Malta | 12.33 ( 11.3 to 13.51 ) | 313931.77 ( 282618.21 to 350488.09 ) | 0 ( 0 to 0 ) | 20.57 ( 18.06 to 22.98 ) | 0.97 ( 0.93 to 1.01 ) | 19106.36 ( 18494.87 to 19629.24 ) | 0.03 ( 0.03 to 0.04 ) | 546.8 ( 507.71 to 575.9 ) |
| Marshall Islands | 2.08 ( 1.91 to 2.25 ) | 374868.87 ( 347408.36 to 402797.06 ) | 0 ( 0 to 0 ) | 129.25 ( 100.78 to 176.2 ) | 0.27 ( 0.27 to 0.28 ) | 48608.96 ( 48122.24 to 49144.62 ) | 0.03 ( 0.02 to 0.03 ) | 4979.52 ( 4122.03 to 6553.43 ) |
| Mauritania | 159.8 ( 144.41 to 181.44 ) | 403989.96 ( 371569.59 to 446919.21 ) | 0.07 ( 0.05 to 0.09 ) | 250.95 ( 199.63 to 315.41 ) | 16.16 ( 17.26 to 18.98 ) | 43633.41 ( 45715.22 to 49977.55 ) | 3.7 ( 2.9 to 4.87 ) | 9665.42 ( 7578.02 to 12578.9 ) |
| Mauritius | 40.92 ( 37.8 to 44.48 ) | 338124.35 ( 309659.47 to 370190.7 ) | 0 ( 0 to 0.01 ) | 31.18 ( 27.07 to 35.84 ) | 7.49 ( 7.17 to 7.84 ) | 55239.66 ( 52727.26 to 57865.45 ) | 0.22 ( 0.19 to 0.25 ) | 1655.03 ( 1480.75 to 1832.21 ) |
| Mexico | 4073.81 ( 3740.19 to 4463.26 ) | 331309.1 ( 303963.52 to 362660.03 ) | 0.38 ( 0.36 to 0.4 ) | 34.14 ( 32.39 to 35.95 ) | 617.85 ( 589.03 to 649.93 ) | 48673.79 ( 46401.39 to 51244.05 ) | 18.17 ( 17.34 to 18.57 ) | 1536.84 ( 1476.43 to 1576.46 ) |
| Micronesia (Federated States of) | 3.68 ( 3.39 to 3.99 ) | 370791.87 ( 343717.2 to 398508.71 ) | 0 ( 0 to 0 ) | 161.98 ( 107.2 to 351.44 ) | 0.5 ( 0.49 to 0.51 ) | 49227.24 ( 48526.85 to 50041.04 ) | 0.06 ( 0.04 to 0.14 ) | 6341.17 ( 4166.62 to 14766.78 ) |
| Monaco | 1.05 ( 0.96 to 1.15 ) | 316533.49 ( 285305.84 to 352122.84 ) | 0 ( 0 to 0 ) | 22.89 ( 18.54 to 27.58 ) | 0.08 ( 0.08 to 0.09 ) | 19128.6 ( 18348.93 to 19762.1 ) | 0 ( 0 to 0 ) | 603.47 ( 520.09 to 739.61 ) |
| Mongolia | 92.71 ( 85.77 to 100.35 ) | 269765.65 ( 249790.35 to 291666.87 ) | 0.01 ( 0.01 to 0.01 ) | 37.59 ( 31.5 to 46.04 ) | 12.79 ( 12.4 to 13.19 ) | 37254.33 ( 36141.66 to 38432.9 ) | 0.87 ( 0.78 to 1 ) | 2411.46 ( 2155.26 to 2743.72 ) |
| Montenegro | 17.41 ( 16.07 to 18.94 ) | 295657.87 ( 271787.65 to 321349 ) | 0 ( 0 to 0 ) | 10.41 ( 8.99 to 11.99 ) | 1.68 ( 1.64 to 1.73 ) | 24627.02 ( 24109.97 to 25019.18 ) | 0.04 ( 0.03 to 0.04 ) | 589.03 ( 523.89 to 644.45 ) |
| Morocco | 1351.15 ( 1236.17 to 1469.18 ) | 384036.38 ( 351640.28 to 416460.81 ) | 0.14 ( 0.12 to 0.17 ) | 52.26 ( 43.93 to 62.91 ) | 135.41 ( 131.88 to 138.85 ) | 37011.95 ( 35984.69 to 37850.27 ) | 7.24 ( 6.22 to 8.37 ) | 2272.67 ( 1935.13 to 2631.31 ) |
| Mozambique | 1046.52 ( 963.77 to 1125.95 ) | 347491.12 ( 324767.96 to 370950.67 ) | 1.43 ( 1.26 to 1.66 ) | 716.43 ( 628.6 to 833.03 ) | 164.49 ( 162.6 to 165.06 ) | 60414.14 ( 59923.03 to 60595.2 ) | 89.17 ( 78.6 to 100.89 ) | 32462.11 ( 28648.78 to 37185.19 ) |
| Myanmar | 1804.79 ( 1652.94 to 1969.22 ) | 336651.4 ( 309151.22 to 366309.76 ) | 0.59 ( 0.5 to 0.71 ) | 132.65 ( 114.98 to 156.35 ) | 279.36 ( 272.04 to 286.69 ) | 50543.81 ( 49246.65 to 51893.79 ) | 29.79 ( 25.44 to 36.1 ) | 5855.63 ( 5029.23 to 7106.58 ) |
| Namibia | 95 ( 87.33 to 103.91 ) | 396446.44 ( 367417.98 to 430718.24 ) | 0.07 ( 0.06 to 0.08 ) | 403.73 ( 347.88 to 469.14 ) | 14.13 ( 13.96 to 14.61 ) | 61257.46 ( 60664.96 to 63053.93 ) | 3.83 ( 3.31 to 4.41 ) | 17581.67 ( 15294.82 to 20350.73 ) |
| Nauru | 0.39 ( 0.35 to 0.42 ) | 374264.94 ( 344574.39 to 403425.91 ) | 0 ( 0 to 0 ) | 111.95 ( 93 to 143.66 ) | 0.05 ( 0.04 to 0.05 ) | 45695.26 ( 44733.62 to 46337.36 ) | 0 ( 0 to 0.01 ) | 4496.56 ( 3822.84 to 5594.77 ) |
| Nepal | 1179.98 ( 1092.57 to 1273.57 ) | 390667.09 ( 363441.19 to 418961.85 ) | 0.29 ( 0.25 to 0.35 ) | 136 ( 112.65 to 171.98 ) | 124.78 ( 119.66 to 128.56 ) | 40027.64 ( 38534.29 to 41157.33 ) | 15 ( 13.56 to 17.23 ) | 5467.18 ( 4916.12 to 6251.08 ) |
| Netherlands | 492.7 ( 450.33 to 541.76 ) | 315485.87 ( 284987.83 to 353491.92 ) | 0.07 ( 0.06 to 0.08 ) | 19.45 ( 17.26 to 21.08 ) | 39.47 ( 37.2 to 41.66 ) | 20056.9 ( 19060.32 to 21047.81 ) | 1.2 ( 1.12 to 1.28 ) | 480.71 ( 454.21 to 515.9 ) |
| New Zealand | 154.13 ( 141.94 to 168.78 ) | 363656.07 ( 329777.86 to 402040.52 ) | 0.01 ( 0.01 to 0.01 ) | 12.04 ( 10.63 to 12.95 ) | 11.4 ( 11 to 11.83 ) | 23524.84 ( 22802.45 to 24074.16 ) | 0.24 ( 0.22 to 0.26 ) | 448.22 ( 408.87 to 496.68 ) |
| Nicaragua | 207.17 ( 188.89 to 226.9 ) | 317152.22 ( 291045.7 to 344408.51 ) | 0.02 ( 0.02 to 0.02 ) | 42.83 ( 37.29 to 48.81 ) | 33.55 ( 32.13 to 34.82 ) | 52219.7 ( 50152.64 to 54030.49 ) | 1.26 ( 1.12 to 1.39 ) | 2067.82 ( 1841.87 to 2279.49 ) |
| Niger | 927.68 ( 853.09 to 1001.28 ) | 395845.52 ( 369820.53 to 421104.8 ) | 1.13 ( 0.89 to 1.4 ) | 503.28 ( 407.13 to 617.08 ) | 104.82 ( 103.94 to 105.32 ) | 51784.32 ( 51259.67 to 51990.73 ) | 86.89 ( 70.38 to 107.16 ) | 24661.38 ( 19842.82 to 30531.11 ) |
| Nigeria | 8229.49 ( 7577.93 to 8919.2 ) | 377506.05 ( 352254.94 to 403430.89 ) | 8.14 ( 6.88 to 9.58 ) | 455.3 ( 389.55 to 528.61 ) | 1071.15 ( 1075.05 to 1073.46 ) | 54657.92 ( 54592.07 to 54917.8 ) | 584.33 ( 497.48 to 690.61 ) | 23000.28 ( 20115.04 to 26573.69 ) |
| Niue | 0.07 ( 0.06 to 0.07 ) | 386162.9 ( 352986.23 to 418639.73 ) | 0 ( 0 to 0 ) | 69.72 ( 55.9 to 95.01 ) | 0.01 ( 0.01 to 0.01 ) | 43639.27 ( 42878.07 to 44408.79 ) | 0 ( 0 to 0 ) | 3107.67 ( 2577.52 to 4117.94 ) |
| North Macedonia | 64.27 ( 59.47 to 69.84 ) | 314832.68 ( 289906.66 to 341942.95 ) | 0 ( 0 to 0 ) | 12.17 ( 10.25 to 14.18 ) | 6.29 ( 6.14 to 6.51 ) | 26049.75 ( 25487.74 to 26623.7 ) | 0.14 ( 0.13 to 0.16 ) | 751.48 ( 688.09 to 791.88 ) |
| Northern Mariana Islands | 1.66 ( 1.53 to 1.8 ) | 392204.62 ( 360622.93 to 423800.9 ) | 0 ( 0 to 0 ) | 47.72 ( 41.9 to 54.33 ) | 0.2 ( 0.19 to 0.2 ) | 42683.78 ( 41813.88 to 43325.36 ) | 0.01 ( 0.01 to 0.01 ) | 2052.01 ( 1824.37 to 2290.85 ) |
| Norway | 170.68 ( 157.32 to 186.49 ) | 340901.36 ( 309107.33 to 377628.55 ) | 0.03 ( 0.02 to 0.03 ) | 21.45 ( 18.59 to 22.84 ) | 11.55 ( 11.04 to 12.03 ) | 19299.62 ( 18603.5 to 19895.54 ) | 0.39 ( 0.36 to 0.43 ) | 500.84 ( 462.32 to 552.17 ) |
| Oman | 166.33 ( 150.98 to 182.29 ) | 377412.8 ( 347483.77 to 406922.88 ) | 0.01 ( 0.01 to 0.01 ) | 57.76 ( 49.47 to 63.94 ) | 13.65 ( 13.39 to 13.88 ) | 27962.73 ( 27556.48 to 28241.81 ) | 0.48 ( 0.45 to 0.52 ) | 1629.89 ( 1562.25 to 1712.25 ) |
| Pakistan | 8388.21 ( 7738.96 to 9114.42 ) | 372150.48 ( 346615.34 to 400167.53 ) | 3.04 ( 2.69 to 3.37 ) | 191.86 ( 165.52 to 222.39 ) | 689.12 ( 670.76 to 705.23 ) | 30126.34 ( 29380.02 to 30793.38 ) | 200.19 ( 180.51 to 214.24 ) | 8479.84 ( 7714.63 to 8980.48 ) |
| Palau | 0.71 ( 0.66 to 0.77 ) | 393995.26 ( 362487.6 to 424384.14 ) | 0 ( 0 to 0 ) | 152.49 ( 124.01 to 189.54 ) | 0.09 ( 0.08 to 0.09 ) | 43449.59 ( 42615.23 to 44012.72 ) | 0.01 ( 0.01 to 0.01 ) | 5149.06 ( 4270.4 to 6419.43 ) |
| Palestine | 182.6 ( 166.41 to 200.2 ) | 357842.23 ( 330664.43 to 387868.43 ) | 0.01 ( 0.01 to 0.01 ) | 31.68 ( 28.63 to 36.98 ) | 15.44 ( 14.9 to 16 ) | 33413.24 ( 32232.52 to 34748.81 ) | 0.46 ( 0.43 to 0.47 ) | 1090.49 ( 1030.28 to 1138.06 ) |
| Panama | 144.03 ( 132.4 to 156.32 ) | 348456.23 ( 320333.93 to 378404.88 ) | 0.02 ( 0.02 to 0.02 ) | 43.4 ( 36.87 to 50.75 ) | 18.98 ( 18.49 to 19.4 ) | 45358.33 ( 44176.57 to 46374.03 ) | 0.92 ( 0.81 to 1.03 ) | 2246.99 ( 1978.69 to 2534.46 ) |
| Papua New Guinea | 395.39 ( 364.68 to 428.28 ) | 405811.58 ( 378641.8 to 435440.87 ) | 0.19 ( 0.15 to 0.26 ) | 245.48 ( 187.3 to 331.76 ) | 58.64 ( 57.08 to 61.27 ) | 61477.32 ( 59875.02 to 64702.94 ) | 13.67 ( 11.12 to 17.23 ) | 12279.27 ( 9834.47 to 16211.25 ) |
| Paraguay | 290.68 ( 266.43 to 320.06 ) | 420358.76 ( 386759.11 to 460624.46 ) | 0.03 ( 0.02 to 0.04 ) | 50.67 ( 40.91 to 61.54 ) | 31.2 ( 30.47 to 31.88 ) | 45387.15 ( 44347.33 to 46323.14 ) | 1.49 ( 1.28 to 1.73 ) | 2338.2 ( 1984.12 to 2727.54 ) |
| Peru | 1384.65 ( 1273.95 to 1503 ) | 411269.3 ( 379253.39 to 446309.7 ) | 0.25 ( 0.2 to 0.32 ) | 76.09 ( 59.84 to 95.74 ) | 138.78 ( 135.47 to 142.35 ) | 40348 ( 39435.65 to 41391.44 ) | 9.25 ( 7.8 to 11.07 ) | 2811.19 ( 2367.64 to 3374.36 ) |
| Philippines | 4289.39 ( 3935.04 to 4712.61 ) | 383647.54 ( 354496.64 to 418590.97 ) | 1.18 ( 1.06 to 1.28 ) | 157.82 ( 136.8 to 175.3 ) | 603.66 ( 587.27 to 627.42 ) | 54303.7 ( 52970.5 to 56430.44 ) | 55.66 ( 52.37 to 59.12 ) | 5419.24 ( 5090.59 to 5753.42 ) |
| Poland | 1037.21 ( 963.48 to 1125.36 ) | 290607.58 ( 267504.87 to 315639.76 ) | 0.13 ( 0.11 to 0.15 ) | 19.3 ( 16.89 to 21.66 ) | 107.21 ( 104.72 to 109.99 ) | 24738.31 ( 24412.77 to 25162.46 ) | 3.52 ( 3.19 to 3.84 ) | 722.97 ( 662.21 to 753.6 ) |
| Portugal | 296.62 ( 273.79 to 324.66 ) | 315134.69 ( 284415.87 to 350342.58 ) | 0.09 ( 0.08 to 0.1 ) | 33.98 ( 30.18 to 36.34 ) | 25.07 ( 24.21 to 25.95 ) | 20133.01 ( 19582.53 to 20537.3 ) | 1.52 ( 1.42 to 1.6 ) | 875.53 ( 825.35 to 927.08 ) |
| Puerto Rico | 127.92 ( 119.94 to 137.01 ) | 365976.74 ( 337786.27 to 397317.56 ) | 0.02 ( 0.01 to 0.02 ) | 24.77 ( 21.13 to 29.11 ) | 15.63 ( 15.05 to 16.2 ) | 39132.76 ( 37820.47 to 40485.44 ) | 0.51 ( 0.45 to 0.57 ) | 1163.45 ( 1050.78 to 1262.76 ) |
| Qatar | 91.98 ( 82.78 to 102.2 ) | 346826.99 ( 320213.86 to 376645.92 ) | 0 ( 0 to 0 ) | 29.77 ( 24.94 to 36.01 ) | 8.27 ( 7.98 to 8.53 ) | 25711.56 ( 25228.63 to 26241.93 ) | 0.12 ( 0.11 to 0.14 ) | 812.82 ( 731.95 to 896.08 ) |
| Republic of Korea | 1583.12 ( 1446.4 to 1746.03 ) | 319716.63 ( 288289.84 to 357589.93 ) | 0.21 ( 0.14 to 0.23 ) | 26.27 ( 18.53 to 29.48 ) | 191.97 ( 184.87 to 198.7 ) | 30798.68 ( 29671.4 to 31686.49 ) | 4.4 ( 3.88 to 4.8 ) | 650.57 ( 587.44 to 707.5 ) |
| Republic of Moldova | 116.56 ( 107.66 to 125.62 ) | 327534.03 ( 300922.31 to 354295.01 ) | 0.01 ( 0.01 to 0.01 ) | 28.63 ( 26.53 to 30.73 ) | 12.72 ( 12.49 to 13.04 ) | 30508.6 ( 29950.63 to 31013.15 ) | 0.62 ( 0.59 to 0.65 ) | 1685.58 ( 1575.02 to 1746.67 ) |
| Romania | 557.45 ( 517.66 to 602 ) | 311352.82 ( 287349.76 to 336617.24 ) | 0.08 ( 0.07 to 0.09 ) | 27.18 ( 23.82 to 31.01 ) | 55.57 ( 54.14 to 57.33 ) | 25731.72 ( 25142.12 to 26371.13 ) | 2.85 ( 2.58 to 3.13 ) | 1457.74 ( 1330.44 to 1564.18 ) |
| Russian Federation | 5002.49 ( 4651.43 to 5378.14 ) | 355065.38 ( 327473.81 to 383186.09 ) | 0.63 ( 0.58 to 0.69 ) | 34.5 ( 32.08 to 36.74 ) | 505.19 ( 490.77 to 519.99 ) | 30930.63 ( 29986.15 to 31845.6 ) | 31.33 ( 29.24 to 33.42 ) | 1961.66 ( 1840.7 to 2077.93 ) |
| Rwanda | 475.09 ( 434.95 to 518.35 ) | 376998.25 ( 350459.44 to 405080 ) | 0.22 ( 0.19 to 0.26 ) | 275.78 ( 242.01 to 317.27 ) | 71.38 ( 69.55 to 73.75 ) | 57604.19 ( 56426.18 to 59149.17 ) | 13.39 ( 11.42 to 15.9 ) | 11312.21 ( 9899.52 to 13092.39 ) |
| Saint Kitts and Nevis | 2.15 ( 2 to 2.33 ) | 373620.18 ( 344649.35 to 405954.41 ) | 0 ( 0 to 0 ) | 96.38 ( 62.89 to 157.93 ) | 0.24 ( 0.23 to 0.24 ) | 35822.34 ( 35229.33 to 36412.71 ) | 0.02 ( 0.01 to 0.04 ) | 3802.62 ( 2425.04 to 6327.27 ) |
| Saint Lucia | 6.42 ( 5.98 to 6.92 ) | 381544.69 ( 353577.35 to 413676.08 ) | 0 ( 0 to 0 ) | 41.15 ( 36.03 to 47.38 ) | 0.75 ( 0.74 to 0.76 ) | 39637.12 ( 39035.06 to 39910.72 ) | 0.03 ( 0.03 to 0.03 ) | 1610.48 ( 1509.16 to 1724.91 ) |
| Saint Vincent and the Grenadines | 4.06 ( 3.76 to 4.39 ) | 367594.85 ( 339361.32 to 398589.76 ) | 0 ( 0 to 0 ) | 61.09 ( 56.32 to 67.1 ) | 0.48 ( 0.47 to 0.48 ) | 40188.42 ( 39610.62 to 40485.14 ) | 0.03 ( 0.03 to 0.03 ) | 2410.89 ( 2271.94 to 2534.16 ) |
| Samoa | 7.91 ( 7.27 to 8.64 ) | 382485.56 ( 355630.52 to 411523.8 ) | 0 ( 0 to 0 ) | 83.45 ( 64.68 to 125.65 ) | 1.03 ( 1 to 1.05 ) | 50563.9 ( 49343.05 to 51439.91 ) | 0.06 ( 0.05 to 0.09 ) | 3396.8 ( 2654.15 to 5108.5 ) |
| San Marino | 0.95 ( 0.87 to 1.04 ) | 317605.52 ( 286702.22 to 353472.66 ) | 0 ( 0 to 0 ) | 14.8 ( 10.66 to 19.69 ) | 0.07 ( 0.07 to 0.07 ) | 19337.65 ( 18573.07 to 20008.71 ) | 0 ( 0 to 0 ) | 554.55 ( 462.23 to 684.86 ) |
| Sao Tome and Principe | 6.87 ( 6.33 to 7.48 ) | 344426.09 ( 321877.92 to 369403.61 ) | 0 ( 0 to 0 ) | 168.68 ( 140.41 to 197.62 ) | 0.78 ( 0.76 to 0.81 ) | 40857.65 ( 39738.55 to 43161.36 ) | 0.1 ( 0.08 to 0.12 ) | 5680.94 ( 4827.82 to 6654.52 ) |
| Saudi Arabia | 1236.46 ( 1128.17 to 1350.38 ) | 367422.2 ( 336233.08 to 397778.54 ) | 0.08 ( 0.06 to 0.09 ) | 46.8 ( 40.71 to 54.44 ) | 129.3 ( 123.66 to 134.88 ) | 33442.72 ( 32413.76 to 34503.06 ) | 4 ( 3.52 to 4.71 ) | 1444.16 ( 1315.4 to 1619.49 ) |
| Senegal | 585.85 ( 539.27 to 633.09 ) | 393147.91 ( 366320.45 to 420177.79 ) | 0.3 ( 0.25 to 0.38 ) | 296.65 ( 246.22 to 384.53 ) | 67.05 ( 66.65 to 67.5 ) | 47330.85 ( 46730.77 to 47754.29 ) | 17.51 ( 14.94 to 21.59 ) | 11853.65 ( 10153.34 to 14448.32 ) |
| Serbia | 244.55 ( 226.74 to 264.82 ) | 297635.91 ( 273867.7 to 323570.25 ) | 0.02 ( 0.02 to 0.02 ) | 14.79 ( 12.41 to 17.47 ) | 24.39 ( 23.91 to 24.91 ) | 25192.39 ( 24757.25 to 25494.38 ) | 0.67 ( 0.61 to 0.75 ) | 680.29 ( 621.38 to 724.21 ) |
| Seychelles | 3.36 ( 3.07 to 3.69 ) | 341067.83 ( 309628.45 to 374388.64 ) | 0 ( 0 to 0 ) | 107.54 ( 93.82 to 118.77 ) | 0.46 ( 0.45 to 0.47 ) | 42712.48 ( 41510.82 to 43742.08 ) | 0.03 ( 0.03 to 0.03 ) | 3057.19 ( 2833.01 to 3227.15 ) |
| Sierra Leone | 302.52 ( 278.19 to 327.22 ) | 369677.4 ( 344441.52 to 395734.34 ) | 0.33 ( 0.26 to 0.4 ) | 526.25 ( 418.85 to 641.5 ) | 44.53 ( 44.1 to 45.63 ) | 56880.5 ( 56086.6 to 58473.98 ) | 21.9 ( 17.46 to 26.25 ) | 24469.73 ( 19519.25 to 29363.49 ) |
| Singapore | 169.57 ( 153.42 to 188.73 ) | 317688.16 ( 285457.65 to 356756.57 ) | 0.04 ( 0.03 to 0.04 ) | 52.88 ( 44.27 to 57.79 ) | 17.56 ( 16.92 to 18.18 ) | 25967.31 ( 25126.15 to 26690.94 ) | 0.65 ( 0.61 to 0.69 ) | 975.83 ( 915.32 to 1020.36 ) |
| Slovakia | 158.84 ( 147.03 to 172.05 ) | 310778.05 ( 286225.66 to 337360.5 ) | 0.02 ( 0.02 to 0.02 ) | 23.52 ( 18.81 to 28.35 ) | 15.14 ( 14.73 to 15.55 ) | 24692.61 ( 24111.9 to 25083.53 ) | 0.55 ( 0.47 to 0.63 ) | 874.03 ( 763.3 to 980.34 ) |
| Slovenia | 56.48 ( 52.27 to 61.14 ) | 296066.58 ( 271693.25 to 321584.36 ) | 0.01 ( 0.01 to 0.01 ) | 14.2 ( 11.65 to 17.11 ) | 5.27 ( 5.1 to 5.45 ) | 22464.72 ( 21932.07 to 22855.99 ) | 0.14 ( 0.12 to 0.15 ) | 483.52 ( 436.3 to 541.04 ) |
| Solomon Islands | 27.51 ( 25.35 to 29.85 ) | 430816.13 ( 403254.91 to 460468.88 ) | 0.01 ( 0.01 to 0.01 ) | 334.6 ( 275.65 to 405.85 ) | 3.38 ( 3.32 to 3.42 ) | 53452.89 ( 52699.11 to 54045.22 ) | 0.64 ( 0.56 to 0.76 ) | 11128.68 ( 9542.96 to 13497.63 ) |
| Somalia | 851.22 ( 778.32 to 925.58 ) | 408477.98 ( 379776.47 to 436174.85 ) | 0.89 ( 0.74 to 1.1 ) | 646.85 ( 527.71 to 802.37 ) | 79.96 ( 81.04 to 79.03 ) | 39911.33 ( 41293.2 to 38423.93 ) | 61.8 ( 52 to 74.72 ) | 26952.94 ( 22397.53 to 32658.26 ) |
| South Africa | 1959.46 ( 1816.55 to 2115.14 ) | 356185.38 ( 331120.25 to 383932.34 ) | 2.16 ( 1.99 to 2.44 ) | 412.49 ( 383.71 to 459.03 ) | 355.1 ( 349.11 to 360.97 ) | 62988.6 ( 61893.87 to 64061.2 ) | 111 ( 101.65 to 124.62 ) | 19653.83 ( 18155.47 to 21676.95 ) |
| South Sudan | 405.2 ( 371.89 to 439.29 ) | 428684.08 ( 400153.82 to 458146.36 ) | 0.35 ( 0.3 to 0.42 ) | 480.66 ( 395.03 to 579.13 ) | 49.38 ( 48.99 to 49.71 ) | 57523.25 ( 57360.52 to 57751.95 ) | 25.06 ( 21.51 to 29.25 ) | 23556.94 ( 20054.82 to 28029.76 ) |
| Spain | 1333.91 ( 1226.54 to 1461.65 ) | 322002.49 ( 291093.41 to 357041.07 ) | 0.17 ( 0.14 to 0.19 ) | 14.38 ( 12.78 to 15.45 ) | 81.39 ( 77.69 to 85.13 ) | 15223 ( 14755.78 to 15597.3 ) | 3.35 ( 3.06 to 3.66 ) | 505.93 ( 465.64 to 561.34 ) |
| Sri Lanka | 704.65 ( 648.76 to 766.68 ) | 331115.44 ( 304188.82 to 361703.53 ) | 0.08 ( 0.06 to 0.11 ) | 40.32 ( 30.79 to 50.97 ) | 93.58 ( 91.4 to 95.74 ) | 41327.84 ( 40294.85 to 42433.16 ) | 3.17 ( 2.7 to 3.6 ) | 1466.86 ( 1265.17 to 1632.75 ) |
| Sudan | 1688.74 ( 1547.32 to 1838.68 ) | 396420.61 ( 366799.02 to 426258.46 ) | 0.29 ( 0.24 to 0.35 ) | 90.78 ( 78.45 to 104.21 ) | 151.05 ( 146.99 to 156.77 ) | 39866.87 ( 38801.52 to 41617.84 ) | 20.64 ( 17.54 to 25.25 ) | 4755.06 ( 4102.38 to 5645.33 ) |
| Suriname | 20.88 ( 19.26 to 22.6 ) | 368661.06 ( 339446.77 to 399305.94 ) | 0 ( 0 to 0 ) | 65.74 ( 57.99 to 75.37 ) | 2.71 ( 2.65 to 2.75 ) | 45898.49 ( 44818.51 to 46499.8 ) | 0.17 ( 0.16 to 0.18 ) | 3113.85 ( 2844.15 to 3383.27 ) |
| Sweden | 331.81 ( 306.91 to 361.84 ) | 342634.51 ( 310917.63 to 381476.79 ) | 0.04 ( 0.03 to 0.04 ) | 14.88 ( 13.08 to 16.17 ) | 31.56 ( 30.11 to 33.15 ) | 27616.18 ( 26486.26 to 28850.71 ) | 0.68 ( 0.62 to 0.75 ) | 441.79 ( 404.47 to 504.42 ) |
| Switzerland | 254.51 ( 234.17 to 278.72 ) | 318277.52 ( 287691.25 to 353189.41 ) | 0.02 ( 0.02 to 0.03 ) | 11.2 ( 9.81 to 12.14 ) | 19.91 ( 18.8 to 20.98 ) | 19523.6 ( 18560.44 to 20381.45 ) | 0.48 ( 0.43 to 0.52 ) | 396.1 ( 362.74 to 444.7 ) |
| Syrian Arab Republic | 509.29 ( 465.33 to 559.68 ) | 360120.46 ( 332063.03 to 392881.4 ) | 0.03 ( 0.03 to 0.04 ) | 31.21 ( 25.81 to 38.21 ) | 46.52 ( 45.45 to 47.67 ) | 32417.29 ( 31718.74 to 33077.19 ) | 1.99 ( 1.75 to 2.25 ) | 1539.7 ( 1350.87 to 1730.24 ) |
| Taiwan (Province of China) | 621.22 ( 577.97 to 669.81 ) | 271696.31 ( 250219.25 to 295267.71 ) | 0.15 ( 0.12 to 0.18 ) | 37.01 ( 30.06 to 45.62 ) | 116.14 ( 111.52 to 120.33 ) | 43464.13 ( 41610.69 to 45120.98 ) | 2.92 ( 2.49 to 3.36 ) | 941.55 ( 821.15 to 1063.24 ) |
| Tajikistan | 230.28 ( 213.51 to 248.47 ) | 235348.79 ( 219300.65 to 253163.11 ) | 0.05 ( 0.05 to 0.07 ) | 69.15 ( 59.32 to 86.86 ) | 34.81 ( 33.72 to 35.93 ) | 37762.49 ( 36569.26 to 38804.92 ) | 4.05 ( 3.45 to 4.94 ) | 3891.69 ( 3368.06 to 4704.63 ) |
| Thailand | 2928.44 ( 2703.48 to 3200.88 ) | 437727.16 ( 400223.83 to 481604.19 ) | 0.67 ( 0.53 to 0.82 ) | 74.13 ( 60.09 to 89.13 ) | 342.91 ( 333.42 to 354.05 ) | 45023.79 ( 43696.95 to 46687.47 ) | 21.61 ( 18.71 to 25.06 ) | 2755.91 ( 2465.05 to 3111.47 ) |
| Timor-Leste | 44.81 ( 40.71 to 49.3 ) | 329861.52 ( 303777.11 to 358237.06 ) | 0.02 ( 0.01 to 0.02 ) | 175.35 ( 143.33 to 245.36 ) | 6.82 ( 6.49 to 7.79 ) | 53903.44 ( 51469.08 to 62410.86 ) | 0.85 ( 0.71 to 1.17 ) | 6554.74 ( 5542.31 to 9494.28 ) |
| Togo | 329.27 ( 303.68 to 356.24 ) | 418389.39 ( 390578.69 to 448683.06 ) | 0.24 ( 0.2 to 0.28 ) | 429.06 ( 367.22 to 505.88 ) | 40.7 ( 40.62 to 40.74 ) | 53857.43 ( 53469.32 to 54106.03 ) | 14.61 ( 12.72 to 17.14 ) | 18498.46 ( 16201.31 to 21248.33 ) |
| Tokelau | 0.05 ( 0.05 to 0.06 ) | 370060.95 ( 342209.39 to 399395.56 ) | 0 ( 0 to 0 ) | 71.35 ( 54.84 to 103.71 ) | 0.01 ( 0.01 to 0.01 ) | 45096.63 ( 44423.11 to 45751.51 ) | 0 ( 0 to 0 ) | 2795.7 ( 2203.13 to 4049.06 ) |
| Tonga | 3.78 ( 3.48 to 4.09 ) | 370047.99 ( 343257.8 to 397942.09 ) | 0 ( 0 to 0 ) | 77.19 ( 63.83 to 93.41 ) | 0.45 ( 0.44 to 0.45 ) | 45536.34 ( 44902.47 to 46079.04 ) | 0.03 ( 0.03 to 0.04 ) | 3113.36 ( 2648.25 to 3616.67 ) |
| Trinidad and Tobago | 46.24 ( 42.76 to 49.97 ) | 344156.57 ( 316530.68 to 373257.24 ) | 0.01 ( 0 to 0.01 ) | 35.75 ( 30.66 to 41.16 ) | 5.91 ( 5.8 to 6.01 ) | 38938.26 ( 38423.15 to 39314.82 ) | 0.25 ( 0.24 to 0.27 ) | 1753.46 ( 1682.56 to 1801.81 ) |
| Tunisia | 405.05 ( 374.22 to 436.28 ) | 361823.28 ( 333226.12 to 391256.09 ) | 0.03 ( 0.02 to 0.03 ) | 24.61 ( 19.28 to 30.8 ) | 38.63 ( 37.33 to 39.9 ) | 31698.96 ( 30634.97 to 32757.91 ) | 1.14 ( 0.99 to 1.32 ) | 1044.39 ( 908.55 to 1185.87 ) |
| Turkey | 3329.85 ( 3055.3 to 3609.13 ) | 428160.01 ( 392270.75 to 465910.33 ) | 0.18 ( 0.16 to 0.21 ) | 23.95 ( 21.61 to 26.11 ) | 266.06 ( 259.54 to 274.59 ) | 30614.26 ( 29940.68 to 31255.96 ) | 7.53 ( 7.14 to 7.7 ) | 1030.21 ( 982.49 to 1054.54 ) |
| Turkmenistan | 128.62 ( 119.18 to 139.94 ) | 249988.82 ( 231907.47 to 271323.38 ) | 0.02 ( 0.02 to 0.02 ) | 42.06 ( 36.14 to 50.09 ) | 16.45 ( 15.98 to 16.85 ) | 32318.71 ( 31435 to 33174.92 ) | 1.56 ( 1.39 to 1.79 ) | 2937.27 ( 2622.1 to 3364.84 ) |
| Tuvalu | 0.43 ( 0.4 to 0.47 ) | 374468.75 ( 345761.19 to 402689.17 ) | 0 ( 0 to 0 ) | 99.55 ( 76.26 to 137 ) | 0.05 ( 0.05 to 0.06 ) | 46506.94 ( 46100.41 to 46785.56 ) | 0 ( 0 to 0.01 ) | 3818.53 ( 3009.25 to 5064.88 ) |
| Uganda | 1879.7 ( 1716.62 to 2059.69 ) | 440600.11 ( 408238.7 to 473934.97 ) | 1.04 ( 0.93 to 1.2 ) | 372.06 ( 329.39 to 423.47 ) | 264.63 ( 264.27 to 265 ) | 67947.91 ( 67884.71 to 68044.85 ) | 72.07 ( 63.36 to 83.24 ) | 16644.45 ( 15016.89 to 18802.37 ) |
| Ukraine | 1399.86 ( 1294.88 to 1511.75 ) | 334199.73 ( 307592.12 to 363116.03 ) | 0.21 ( 0.19 to 0.23 ) | 37.32 ( 34.56 to 40.69 ) | 162.83 ( 157.12 to 168.76 ) | 32346.59 ( 31322.61 to 33369.07 ) | 10.2 ( 9.4 to 11.09 ) | 2077.82 ( 1953.28 to 2218.59 ) |
| United Arab Emirates | 302.05 ( 269.45 to 339.17 ) | 365388.66 ( 336538.56 to 396172.68 ) | 0.01 ( 0.01 to 0.02 ) | 69.25 ( 38.99 to 95.03 ) | 38.17 ( 36.09 to 39.97 ) | 34936.55 ( 33926.19 to 35905.94 ) | 0.73 ( 0.61 to 0.95 ) | 1539.95 ( 1305.95 to 1912.97 ) |
| United Kingdom | 2053.08 ( 1891.28 to 2249.56 ) | 330837.16 ( 299963.25 to 367693.83 ) | 0.45 ( 0.39 to 0.48 ) | 30.97 ( 27.86 to 32.31 ) | 140.85 ( 135.36 to 146.16 ) | 18823.59 ( 18225.46 to 19360.91 ) | 6.91 ( 6.56 to 7.28 ) | 696.7 ( 664.63 to 731.96 ) |
| United Republic of Tanzania | 2259.42 ( 2062.56 to 2455.83 ) | 393686.7 ( 364664 to 423055.53 ) | 1.33 ( 1.18 to 1.52 ) | 333.62 ( 300.36 to 372.55 ) | 260.64 ( 260.76 to 260.8 ) | 49807.81 ( 49543.51 to 50084.56 ) | 84.25 ( 74.34 to 97.24 ) | 14341.87 ( 12839.41 to 16127.09 ) |
| United States of America | 12113.78 ( 11144.13 to 13243.28 ) | 396770.2 ( 361392.3 to 438311.24 ) | 1.09 ( 0.98 to 1.14 ) | 18.82 ( 17.63 to 19.38 ) | 1017.7 ( 967.01 to 1067.05 ) | 27642.78 ( 26363.91 to 28810.9 ) | 28.95 ( 26.72 to 31.94 ) | 718.3 ( 656.97 to 791.8 ) |
| United States Virgin Islands | 3.79 ( 3.54 to 4.08 ) | 369461.35 ( 340630.43 to 401121.86 ) | 0 ( 0 to 0 ) | 28.46 ( 25.89 to 31.09 ) | 0.42 ( 0.41 to 0.43 ) | 36189.39 ( 35413.78 to 36818.98 ) | 0.01 ( 0.01 to 0.02 ) | 1286.59 ( 1188.96 to 1372.43 ) |
| Uruguay | 112.78 ( 103.16 to 123.89 ) | 345300.39 ( 314132.22 to 381131.1 ) | 0.02 ( 0.02 to 0.02 ) | 35.14 ( 32.41 to 37.37 ) | 11.97 ( 11.58 to 12.33 ) | 31924.38 ( 30920.14 to 32710.11 ) | 0.47 ( 0.45 to 0.49 ) | 1131.85 ( 1086.16 to 1169.66 ) |
| Uzbekistan | 793.19 ( 733.2 to 857.73 ) | 230475.76 ( 214140.5 to 248579.27 ) | 0.15 ( 0.13 to 0.18 ) | 54.96 ( 48.04 to 63.13 ) | 100.64 ( 99.43 to 101.66 ) | 30112.57 ( 29792.39 to 30423.93 ) | 11.3 ( 10.15 to 12.74 ) | 3366.85 ( 3025.07 to 3787.29 ) |
| Vanuatu | 11.37 ( 10.48 to 12.34 ) | 392876.09 ( 366247.28 to 422059.42 ) | 0 ( 0 to 0 ) | 158.87 ( 122.82 to 212.59 ) | 1.58 ( 1.55 to 1.62 ) | 54397.5 ( 53597.04 to 55660.43 ) | 0.17 ( 0.14 to 0.2 ) | 6037.69 ( 5013 to 7555.79 ) |
| Venezuela (Bolivarian Republic of) | 986.74 ( 906.1 to 1072.89 ) | 358559.68 ( 328689.67 to 390512.96 ) | 0.12 ( 0.1 to 0.15 ) | 44.51 ( 35.99 to 54.78 ) | 166.45 ( 161.11 to 171.85 ) | 58264.51 ( 56348.44 to 60267.22 ) | 5.76 ( 4.82 to 6.7 ) | 2107.14 ( 1766.13 to 2456.24 ) |
| Viet Nam | 2593.59 ( 2383.24 to 2826.42 ) | 279885.09 ( 257330.63 to 305070.15 ) | 0.55 ( 0.49 to 0.61 ) | 67.18 ( 59.97 to 74.66 ) | 558.66 ( 543.27 to 573.8 ) | 55990.63 ( 54234.94 to 57648.31 ) | 22.44 ( 20.43 to 24.81 ) | 2513.71 ( 2315.85 to 2749.35 ) |
| Yemen | 1463.34 ( 1334.42 to 1600.91 ) | 441229.63 ( 407956.58 to 476067.59 ) | 0.23 ( 0.19 to 0.28 ) | 98.57 ( 77.53 to 130.85 ) | 110.78 ( 109 to 113.5 ) | 38498.93 ( 37859.16 to 39450.3 ) | 17.75 ( 16.36 to 19.71 ) | 5246.66 ( 4697.74 to 5995.39 ) |
| Zambia | 811.32 ( 747.33 to 883.19 ) | 440775.51 ( 410953.39 to 471577.05 ) | 0.55 ( 0.49 to 0.64 ) | 463.47 ( 415.36 to 526.54 ) | 83.4 ( 83.23 to 83.76 ) | 49874.96 ( 49600.13 to 50140.26 ) | 35.15 ( 31.23 to 40.59 ) | 20675.69 ( 18626.16 to 23083.59 ) |
| Zimbabwe | 571.12 ( 523.59 to 620.36 ) | 380795.33 ( 354327.11 to 409590.23 ) | 0.55 ( 0.5 to 0.61 ) | 551.64 ( 489.95 to 621.92 ) | 64.48 ( 63.5 to 65.12 ) | 47516.14 ( 46629.81 to 48016.36 ) | 30.45 ( 27.66 to 33.65 ) | 22905.13 ( 20753.36 to 25127.27 ) |

Table 8 Burden of non-communicable diseases in 204 countries and regions in 2019.

| **location** | **Incidence** | | **Deaths** | | **Prevalence** | | **DALYs** | |
| --- | --- | --- | --- | --- | --- | --- | --- | --- |
|  | **Count**  **(*10^5^)** | **ASIR**  **(per100,000)** | **Count**  **(*10^5^)** | **ASDR**  **(per100,000)** | **Count**  **(*10^5^)** | **ASPR**  **(per100,000)** | **Count**  **(*10^5^)** | **Age-standardized DALY rate**  **(per100,000)** |
| Afghanistan | 577.66 ( 538.36 to 617.74 ) | 159365.9 ( 150665.76 to 167445.7 ) | 1.32 ( 1.06 to 1.58 ) | 1027.22 ( 853.74 to 1195.48 ) | 335.2 ( 328.41 to 341.38 ) | 91662.66 ( 90534.85 to 92689.87 ) | 76.02 ( 62.29 to 90.11 ) | 33303.39 ( 28003.98 to 38804.9 ) |
| Albania | 45.86 ( 44.01 to 47.68 ) | 161110.63 ( 153491.92 to 168904.37 ) | 0.21 ( 0.17 to 0.26 ) | 519.94 ( 412.87 to 648.81 ) | 25.36 ( 25.13 to 25.57 ) | 90504.32 ( 89269.81 to 91599.11 ) | 6.32 ( 5.19 to 7.57 ) | 17621.17 ( 14627.75 to 21166.79 ) |
| Algeria | 668.73 ( 633.89 to 705.85 ) | 160054.07 ( 151838.15 to 168516.57 ) | 1.64 ( 1.41 to 1.92 ) | 631.29 ( 549.24 to 724.83 ) | 381.51 ( 376.59 to 386.17 ) | 91177.01 ( 89989.48 to 92303.74 ) | 74.47 ( 63.26 to 87.15 ) | 20551.25 ( 17641.5 to 23826.85 ) |
| American Samoa | 0.87 ( 0.82 to 0.92 ) | 158096.6 ( 149168.64 to 166611.17 ) | 0 ( 0 to 0 ) | 769.63 ( 678.36 to 876.27 ) | 0.5 ( 0.5 to 0.51 ) | 90891.34 ( 89724.73 to 91951.79 ) | 0.13 ( 0.11 to 0.15 ) | 26266.74 ( 22751.41 to 30252.93 ) |
| Andorra | 1.6 ( 1.54 to 1.66 ) | 179682.75 ( 171166.16 to 188196 ) | 0.01 ( 0 to 0.01 ) | 366.26 ( 292.12 to 451.47 ) | 0.78 ( 0.78 to 0.79 ) | 88683.28 ( 87597.01 to 89714.92 ) | 0.19 ( 0.16 to 0.23 ) | 16039.71 ( 13073.09 to 19368.69 ) |
| Angola | 521.38 ( 483.99 to 561.5 ) | 178902.14 ( 169482.3 to 188679.64 ) | 0.67 ( 0.55 to 0.82 ) | 668.95 ( 584.11 to 788.54 ) | 273.22 ( 269.14 to 277.44 ) | 93721.24 ( 92829.43 to 94613.69 ) | 41.77 ( 34.36 to 49.55 ) | 23277.68 ( 19840.62 to 27211 ) |
| Antigua and Barbuda | 1.64 ( 1.58 to 1.7 ) | 182255.89 ( 174239.32 to 190833.26 ) | 0.01 ( 0 to 0.01 ) | 583.14 ( 522.7 to 647.42 ) | 0.83 ( 0.82 to 0.83 ) | 91383.88 ( 90333.38 to 92406.34 ) | 0.2 ( 0.17 to 0.23 ) | 20217.28 ( 17368.21 to 23475.94 ) |
| Argentina | 724.63 ( 689.21 to 758.44 ) | 157214.44 ( 148956.91 to 165319.1 ) | 2.8 ( 2.74 to 2.86 ) | 513.56 ( 501.84 to 526.29 ) | 407.05 ( 401.88 to 411.93 ) | 88730.79 ( 87400.99 to 90006.95 ) | 96.99 ( 85.92 to 109.36 ) | 19303.81 ( 16993.7 to 21847.72 ) |
| Armenia | 50.07 ( 47.89 to 52.32 ) | 159561.61 ( 151745.64 to 167441.94 ) | 0.26 ( 0.23 to 0.3 ) | 662.41 ( 577.37 to 754.42 ) | 27.86 ( 27.57 to 28.11 ) | 89794.1 ( 88514.46 to 90959.53 ) | 7.8 ( 6.78 to 8.88 ) | 20789.97 ( 18064.59 to 23668.24 ) |
| Australia | 463.14 ( 445.02 to 481.83 ) | 178557.8 ( 170383.43 to 186946.11 ) | 1.55 ( 1.52 to 1.59 ) | 345.74 ( 338.82 to 353.27 ) | 224.53 ( 222.39 to 226.7 ) | 87956.98 ( 86670.96 to 89249.48 ) | 53.24 ( 45.91 to 61.42 ) | 16056.02 ( 13537.48 to 18933.29 ) |
| Austria | 187.18 ( 180.71 to 194.64 ) | 194306.71 ( 185839.12 to 203522.33 ) | 0.77 ( 0.75 to 0.78 ) | 380.67 ( 373.83 to 387.96 ) | 83.63 ( 83.11 to 84.15 ) | 89241.21 ( 88203.49 to 90285.08 ) | 22.31 ( 19.48 to 25.59 ) | 16238.88 ( 13738.48 to 19151.55 ) |
| Azerbaijan | 162.45 ( 154.35 to 170.65 ) | 158073.16 ( 149985.52 to 166217.57 ) | 0.66 ( 0.6 to 0.75 ) | 994.95 ( 905.64 to 1103.34 ) | 94.03 ( 92.96 to 94.97 ) | 89988.61 ( 88741.9 to 91093.35 ) | 24.26 ( 21.21 to 27.67 ) | 26391.42 ( 23407.93 to 29779.29 ) |
| Bahamas | 6.92 ( 6.63 to 7.23 ) | 182528.88 ( 173907.13 to 191336.14 ) | 0.02 ( 0.02 to 0.03 ) | 592.78 ( 501.28 to 706.51 ) | 3.52 ( 3.49 to 3.55 ) | 91658.81 ( 90598.88 to 92652.7 ) | 0.87 ( 0.73 to 1.04 ) | 22044.61 ( 18546.78 to 26066.96 ) |
| Bahrain | 22.96 ( 21.89 to 24.14 ) | 158862.16 ( 150785.26 to 167211.05 ) | 0.04 ( 0.03 to 0.04 ) | 666.01 ( 572.81 to 768.5 ) | 13.82 ( 13.74 to 13.9 ) | 93710.2 ( 92850.22 to 94579.28 ) | 2.4 ( 1.99 to 2.86 ) | 20772.19 ( 17717.57 to 24226.88 ) |
| Bangladesh | 2826.91 ( 2693.95 to 2958.42 ) | 178717.49 ( 170456.32 to 186761.02 ) | 6.33 ( 5.26 to 7.54 ) | 543.53 ( 453.08 to 641.78 ) | 1450.22 ( 1431.97 to 1468.01 ) | 90985 ( 89838.61 to 92105.38 ) | 282.57 ( 238.31 to 330.67 ) | 20089.94 ( 16976.26 to 23392.49 ) |
| Barbados | 5.69 ( 5.48 to 5.91 ) | 182377.57 ( 174232.55 to 190973.03 ) | 0.03 ( 0.02 to 0.03 ) | 567.66 ( 487.02 to 648.61 ) | 2.81 ( 2.79 to 2.83 ) | 91409.26 ( 90346.29 to 92453.17 ) | 0.83 ( 0.71 to 0.96 ) | 20106.96 ( 17074.2 to 23581.41 ) |
| Belarus | 175.88 ( 168.62 to 182.69 ) | 171831.27 ( 163486.98 to 180162.65 ) | 1.11 ( 0.91 to 1.37 ) | 699.88 ( 572.83 to 862.27 ) | 88.68 ( 87.91 to 89.38 ) | 89856.66 ( 88550.86 to 91021.94 ) | 31.19 ( 25.96 to 37.15 ) | 22491.14 ( 18776.62 to 26874.52 ) |
| Belgium | 217.05 ( 208.49 to 225.65 ) | 177455.39 ( 168624.66 to 185947.77 ) | 0.98 ( 0.96 to 1.01 ) | 378.4 ( 369.62 to 388.72 ) | 106.08 ( 105.33 to 106.82 ) | 88783.74 ( 87751.74 to 89836.77 ) | 28.69 ( 24.92 to 32.87 ) | 16512.63 ( 13920.44 to 19413.16 ) |
| Belize | 7.23 ( 6.88 to 7.6 ) | 180861.64 ( 172773 to 189547.44 ) | 0.01 ( 0.01 to 0.02 ) | 517.31 ( 461.78 to 578.18 ) | 3.74 ( 3.69 to 3.78 ) | 91678.43 ( 90571.85 to 92630.45 ) | 0.68 ( 0.58 to 0.78 ) | 20164.35 ( 17533.3 to 23132.94 ) |
| Benin | 215.38 ( 200.18 to 230.62 ) | 178565.96 ( 169174.17 to 188179.87 ) | 0.32 ( 0.25 to 0.4 ) | 627 ( 519.69 to 761.55 ) | 112.83 ( 110.93 to 114.77 ) | 92758.91 ( 91802.39 to 93721.86 ) | 19.15 ( 15.23 to 23.92 ) | 22656.17 ( 18788.69 to 27293.6 ) |
| Bermuda | 1.26 ( 1.22 to 1.31 ) | 183765.41 ( 175758.88 to 192100.9 ) | 0.01 ( 0 to 0.01 ) | 392.9 ( 343.85 to 461.03 ) | 0.61 ( 0.61 to 0.61 ) | 91147.5 ( 90102.74 to 92194.86 ) | 0.16 ( 0.14 to 0.19 ) | 15777.08 ( 13288.98 to 18769.75 ) |
| Bhutan | 13.19 ( 12.63 to 13.75 ) | 176750.83 ( 168951.25 to 184490.41 ) | 0.03 ( 0.03 to 0.04 ) | 620.3 ( 525.16 to 703.99 ) | 6.77 ( 6.68 to 6.86 ) | 89524.47 ( 88254.16 to 90711.02 ) | 1.33 ( 1.12 to 1.56 ) | 21044.62 ( 17849.51 to 24392.01 ) |
| Bolivia (Plurinational State of) | 223.63 ( 212.83 to 234.92 ) | 191016.02 ( 182400.27 to 200346.67 ) | 0.53 ( 0.44 to 0.63 ) | 668.13 ( 563.59 to 778.44 ) | 108.14 ( 106.47 to 109.61 ) | 91316.15 ( 90125.27 to 92410.69 ) | 22.48 ( 18.82 to 26.59 ) | 22441.18 ( 18859.94 to 26389.63 ) |
| Bosnia and Herzegovina | 57.72 ( 55.31 to 60.28 ) | 163537.11 ( 155805.98 to 171353.54 ) | 0.36 ( 0.29 to 0.43 ) | 637.45 ( 527.14 to 766.29 ) | 31.12 ( 30.89 to 31.34 ) | 90459.01 ( 89315.16 to 91554.49 ) | 9.98 ( 8.37 to 11.79 ) | 19619.53 ( 16507.26 to 23178.77 ) |
| Botswana | 43.5 ( 41.29 to 45.73 ) | 190195.05 ( 181428.81 to 199285.21 ) | 0.09 ( 0.07 to 0.12 ) | 793.76 ( 641.04 to 991 ) | 20.99 ( 20.7 to 21.27 ) | 90368.05 ( 89217.81 to 91527.21 ) | 4.34 ( 3.5 to 5.29 ) | 25671.73 ( 20993.37 to 31174.74 ) |
| Brazil | 4298.79 ( 4145.88 to 4456.81 ) | 195541.98 ( 187808.69 to 204095.61 ) | 10.71 ( 10.43 to 11.01 ) | 474.55 ( 462.01 to 487.86 ) | 2010.23 ( 1993.21 to 2025.6 ) | 91278.3 ( 90317.5 to 92158.99 ) | 466.36 ( 409.15 to 532.49 ) | 20309.17 ( 17782.45 to 23166.19 ) |
| Brunei Darussalam | 6.93 ( 6.61 to 7.26 ) | 162669.7 ( 154659.3 to 170550.25 ) | 0.02 ( 0.01 to 0.02 ) | 746.06 ( 685.25 to 815.9 ) | 3.93 ( 3.88 to 3.97 ) | 88544.72 ( 87312.56 to 89725.65 ) | 0.78 ( 0.68 to 0.9 ) | 22663.02 ( 20004.15 to 25528.27 ) |
| Bulgaria | 124.35 ( 119.18 to 129.76 ) | 163196.74 ( 155346 to 171417.39 ) | 1.18 ( 0.99 to 1.41 ) | 831.04 ( 692.28 to 992.11 ) | 65.46 ( 64.99 to 65.9 ) | 90302.92 ( 89036.37 to 91448.53 ) | 28.86 ( 24.37 to 33.93 ) | 24342.41 ( 20405.94 to 28775.13 ) |
| Burkina Faso | 388.25 ( 363.27 to 415.62 ) | 179491.32 ( 170695.05 to 189184.9 ) | 0.67 ( 0.53 to 0.84 ) | 657.51 ( 564.93 to 763.08 ) | 207.3 ( 204.23 to 210.42 ) | 94310.85 ( 93434.04 to 95167.42 ) | 42.03 ( 32.56 to 53.3 ) | 24698.78 ( 20574.89 to 29489.31 ) |
| Burundi | 197.25 ( 183.39 to 211.63 ) | 171472.04 ( 162281.32 to 181008.95 ) | 0.29 ( 0.23 to 0.36 ) | 658.11 ( 543.72 to 804.28 ) | 104.1 ( 101.98 to 106.03 ) | 91134.03 ( 89979.69 to 92256.92 ) | 16.58 ( 13.49 to 20.08 ) | 22518.77 ( 18744.32 to 26737.64 ) |
| Cabo Verde | 9.92 ( 9.42 to 10.46 ) | 178254.42 ( 169383.52 to 187815.53 ) | 0.03 ( 0.02 to 0.03 ) | 601.14 ( 550.26 to 653.06 ) | 5.09 ( 5.02 to 5.15 ) | 90339.77 ( 89214.17 to 91524.77 ) | 0.97 ( 0.83 to 1.12 ) | 20231.67 ( 17561.1 to 23080.23 ) |
| Cambodia | 260.37 ( 245.9 to 274.6 ) | 159000.05 ( 150915.01 to 167437.81 ) | 0.73 ( 0.61 to 0.83 ) | 706.88 ( 599.2 to 782.27 ) | 152.84 ( 151.13 to 154.41 ) | 92594.54 ( 91640.38 to 93474.93 ) | 32.43 ( 27.65 to 37.64 ) | 23979.08 ( 20577.03 to 27477.1 ) |
| Cameroon | 493.07 ( 461.02 to 525.01 ) | 177464.7 ( 168966.61 to 186645.95 ) | 0.73 ( 0.57 to 0.95 ) | 651.28 ( 536.12 to 809.2 ) | 258.95 ( 254.94 to 262.93 ) | 91996.11 ( 91030.87 to 92983.93 ) | 42.76 ( 34.34 to 52.62 ) | 22920.36 ( 18688.62 to 27660.92 ) |
| Canada | 550.89 ( 529.96 to 573.44 ) | 142397.43 ( 135035.41 to 149975.56 ) | 2.58 ( 2.54 to 2.62 ) | 356.64 ( 351.5 to 362.3 ) | 337.46 ( 334.53 to 340.31 ) | 88303.34 ( 87084.41 to 89586.13 ) | 86.34 ( 75.22 to 98.94 ) | 16352.54 ( 13875.99 to 19155.34 ) |
| Central African Republic | 91.69 ( 85.5 to 98.19 ) | 178586.91 ( 169305.08 to 188560.3 ) | 0.18 ( 0.14 to 0.23 ) | 873.67 ( 690.69 to 1083.37 ) | 48.36 ( 47.67 to 49.05 ) | 93760.38 ( 92876.35 to 94646.95 ) | 10 ( 8.03 to 12.59 ) | 29741.38 ( 24354.52 to 36287.71 ) |
| Chad | 277.86 ( 256.47 to 299.29 ) | 179887.14 ( 170647.88 to 189249.74 ) | 0.4 ( 0.33 to 0.48 ) | 657.42 ( 562.3 to 773.6 ) | 143.61 ( 140.72 to 146.37 ) | 92509.39 ( 91485.04 to 93476.55 ) | 24.6 ( 19.5 to 30.18 ) | 23640.22 ( 20062.54 to 27713.27 ) |
| Chile | 296.5 ( 284.43 to 308.17 ) | 158518.57 ( 150805.44 to 165864.78 ) | 0.97 ( 0.94 to 1 ) | 412.45 ( 400.93 to 425.38 ) | 167.69 ( 166.13 to 169.14 ) | 89578.95 ( 88381.18 to 90695.55 ) | 37.1 ( 32.32 to 42.71 ) | 17179.46 ( 14867.35 to 19879.12 ) |
| China | 20822.44 ( 20078.03 to 21723.2 ) | 145546.32 ( 138972.85 to 152643.48 ) | 95.97 ( 84.02 to 108.79 ) | 562.25 ( 496.27 to 630.65 ) | 13197.62 ( 13108.92 to 13282.13 ) | 89329.89 ( 88335.09 to 90241.4 ) | 3246.38 ( 2814.43 to 3697.57 ) | 18058.96 ( 15688.21 to 20661.96 ) |
| Colombia | 765.37 ( 734.15 to 801.39 ) | 159318.99 ( 151923.05 to 167297.1 ) | 1.94 ( 1.56 to 2.39 ) | 356.91 ( 284.68 to 442.19 ) | 435.51 ( 431.68 to 439.04 ) | 89723.41 ( 88812 to 90591.44 ) | 81.8 ( 67.47 to 99.4 ) | 16020.65 ( 13188.42 to 19478.03 ) |
| Comoros | 12.02 ( 11.31 to 12.7 ) | 171474.45 ( 162379.69 to 180272.54 ) | 0.03 ( 0.02 to 0.03 ) | 601.67 ( 521.19 to 715.18 ) | 6.41 ( 6.33 to 6.5 ) | 90916.8 ( 89827.91 to 92003.82 ) | 1.18 ( 0.98 to 1.4 ) | 20846.33 ( 17561.32 to 24473.57 ) |
| Congo | 91.64 ( 85.9 to 97.77 ) | 178165.52 ( 168952.65 to 188109.73 ) | 0.17 ( 0.14 to 0.2 ) | 753.46 ( 649.23 to 893.04 ) | 48.1 ( 47.43 to 48.79 ) | 93074.66 ( 92097.32 to 94091.99 ) | 8.51 ( 6.95 to 10.22 ) | 24508.74 ( 20566.79 to 28872.45 ) |
| Cook Islands | 0.29 ( 0.27 to 0.3 ) | 159078.81 ( 150764.3 to 167147.46 ) | 0 ( 0 to 0 ) | 610.93 ( 530.5 to 712.56 ) | 0.17 ( 0.16 to 0.17 ) | 90389.13 ( 89193.8 to 91485.7 ) | 0.05 ( 0.04 to 0.06 ) | 21803.12 ( 18652.68 to 25522.04 ) |
| Costa Rica | 80.11 ( 76.74 to 83.5 ) | 168759.16 ( 160832.43 to 176933.47 ) | 0.2 ( 0.16 to 0.25 ) | 398.15 ( 319.98 to 491.08 ) | 42.99 ( 42.49 to 43.45 ) | 89444.96 ( 88180.76 to 90627.29 ) | 8.58 ( 7.1 to 10.37 ) | 17073.79 ( 14139.22 to 20641.45 ) |
| Croatia | 78.91 ( 76.04 to 81.83 ) | 168962.25 ( 161489.97 to 176758.08 ) | 0.49 ( 0.4 to 0.59 ) | 540.02 ( 444.96 to 653.25 ) | 40.16 ( 39.86 to 40.45 ) | 90442.1 ( 89207.17 to 91647.31 ) | 12.78 ( 10.71 to 15.05 ) | 17974.42 ( 14981.03 to 21350.72 ) |
| Cuba | 218.32 ( 210.52 to 227.41 ) | 181985.72 ( 173835.61 to 191172.78 ) | 0.89 ( 0.75 to 1.05 ) | 457.98 ( 384.9 to 540.42 ) | 107.13 ( 106.33 to 107.9 ) | 90943.52 ( 89804.9 to 92061.94 ) | 29 ( 24.61 to 33.9 ) | 17953.75 ( 15147.77 to 21149.27 ) |
| Cyprus | 24.25 ( 23.35 to 25.18 ) | 175116.62 ( 166948.79 to 183485.05 ) | 0.08 ( 0.07 to 0.09 ) | 465.97 ( 431.26 to 505.54 ) | 12.3 ( 12.22 to 12.38 ) | 90451.26 ( 89445.46 to 91436.2 ) | 2.77 ( 2.37 to 3.22 ) | 16598.88 ( 14056.53 to 19447.94 ) |
| Czechia | 194.43 ( 187.31 to 202.12 ) | 167973.14 ( 160265.51 to 176052.29 ) | 1.04 ( 0.88 to 1.22 ) | 484.62 ( 409.83 to 571.37 ) | 99.84 ( 99.04 to 100.58 ) | 89958.77 ( 88692.88 to 91126.16 ) | 29.44 ( 25.15 to 34.38 ) | 17124.76 ( 14440.86 to 20203.46 ) |
| Democratic People's Republic of Korea | 377.45 ( 361.02 to 395.27 ) | 143026.78 ( 135619.28 to 150829.23 ) | 2.03 ( 1.8 to 2.33 ) | 694.18 ( 619.24 to 794.47 ) | 239.01 ( 236.74 to 241.13 ) | 88658.11 ( 87479.44 to 89743.63 ) | 69.21 ( 59.39 to 80.35 ) | 22644.73 ( 19402.82 to 26270.29 ) |
| Democratic Republic of the Congo | 1508.45 ( 1408.12 to 1615.8 ) | 177330.34 ( 168481.47 to 186666.88 ) | 2.19 ( 1.8 to 2.63 ) | 681.47 ( 568.69 to 809.23 ) | 799.86 ( 788.15 to 811.25 ) | 93724.1 ( 92815.37 to 94612 ) | 124.89 ( 103.13 to 148.18 ) | 23259.95 ( 19577.09 to 27298.69 ) |
| Denmark | 113.46 ( 109.38 to 118.1 ) | 182563.65 ( 174115.64 to 191919.06 ) | 0.5 ( 0.48 to 0.51 ) | 410.71 ( 398.68 to 423.25 ) | 53.76 ( 53.37 to 54.13 ) | 88238.95 ( 87167.65 to 89262.23 ) | 15.08 ( 13.27 to 17.06 ) | 17165.59 ( 14671.52 to 19954.93 ) |
| Djibouti | 19.98 ( 18.84 to 21.18 ) | 171120.25 ( 162378.69 to 180052.52 ) | 0.03 ( 0.02 to 0.04 ) | 633.69 ( 530.75 to 786.41 ) | 10.68 ( 10.52 to 10.84 ) | 90644.54 ( 89525.22 to 91738.04 ) | 1.82 ( 1.46 to 2.25 ) | 21673.83 ( 17966.34 to 26200.12 ) |
| Dominica | 1.28 ( 1.23 to 1.33 ) | 181711 ( 173445.55 to 190568.31 ) | 0.01 ( 0.01 to 0.01 ) | 713.43 ( 616.43 to 834.74 ) | 0.64 ( 0.64 to 0.65 ) | 91600.14 ( 90565.36 to 92575.05 ) | 0.2 ( 0.17 to 0.23 ) | 24217.72 ( 20524.98 to 28471.92 ) |
| Dominican Republic | 194.72 ( 185.99 to 203.68 ) | 181059.44 ( 172828.54 to 189404.31 ) | 0.53 ( 0.44 to 0.66 ) | 601.61 ( 497.42 to 734 ) | 99.24 ( 97.99 to 100.38 ) | 91322.89 ( 90183.01 to 92363.99 ) | 21.45 ( 17.7 to 25.78 ) | 21486.58 ( 17806.05 to 25796.25 ) |
| Ecuador | 341.67 ( 326.66 to 357.83 ) | 196585.21 ( 188308.41 to 205870.85 ) | 0.69 ( 0.56 to 0.85 ) | 501.76 ( 412.97 to 615.24 ) | 160.45 ( 158.29 to 162.36 ) | 91392.22 ( 90191.26 to 92445.21 ) | 29.99 ( 25.13 to 35.79 ) | 18685.83 ( 15713.19 to 22245.97 ) |
| Egypt | 1581.39 ( 1498.46 to 1663.63 ) | 161841.35 ( 154298.99 to 169312.32 ) | 4.76 ( 3.82 to 5.89 ) | 913.17 ( 751.55 to 1098.67 ) | 899.45 ( 886.98 to 910.19 ) | 91819.19 ( 90729.88 to 92765.77 ) | 203.92 ( 168.46 to 244.4 ) | 27807.15 ( 23129.98 to 33041.68 ) |
| El Salvador | 105.14 ( 100.25 to 110.03 ) | 168642.18 ( 160750.85 to 176518.35 ) | 0.3 ( 0.24 to 0.37 ) | 476.6 ( 375.39 to 595.06 ) | 56.16 ( 55.4 to 56.91 ) | 89642.26 ( 88396.41 to 90843.07 ) | 11.95 ( 9.79 to 14.39 ) | 19698.81 ( 16114.16 to 23760.2 ) |
| Equatorial Guinea | 24.42 ( 22.8 to 25.99 ) | 179022.56 ( 170065.49 to 187817.91 ) | 0.03 ( 0.02 to 0.04 ) | 626.03 ( 504.25 to 804.5 ) | 12.68 ( 12.5 to 12.87 ) | 92068.93 ( 91136.16 to 93061.5 ) | 1.67 ( 1.31 to 2.11 ) | 21242.3 ( 17021.61 to 26369.91 ) |
| Eritrea | 111 ( 103.73 to 118.43 ) | 171505.71 ( 162705.94 to 180794.91 ) | 0.17 ( 0.14 to 0.22 ) | 729.17 ( 587.33 to 906.49 ) | 59.45 ( 58.4 to 60.41 ) | 91312.68 ( 90138.5 to 92396.89 ) | 9.98 ( 8.06 to 12.22 ) | 24453.39 ( 19900.75 to 29628.15 ) |
| Estonia | 24.78 ( 23.81 to 25.82 ) | 172018.26 ( 163898.31 to 180221.51 ) | 0.15 ( 0.12 to 0.18 ) | 528.39 ( 432.11 to 640.13 ) | 12.29 ( 12.19 to 12.38 ) | 89835.73 ( 88574 to 90987.77 ) | 3.93 ( 3.3 to 4.63 ) | 18873.54 ( 15777.06 to 22305.49 ) |
| Eswatini | 20.89 ( 19.74 to 21.99 ) | 189504.75 ( 180812.54 to 198315.05 ) | 0.04 ( 0.03 to 0.06 ) | 885.41 ( 684.6 to 1140.03 ) | 10.21 ( 10.04 to 10.36 ) | 91432.22 ( 90293.97 to 92488.37 ) | 2.08 ( 1.67 to 2.56 ) | 28159.05 ( 22774.01 to 34635.54 ) |
| Ethiopia | 2016.65 ( 1889.87 to 2149.74 ) | 190254.76 ( 181151.98 to 199599.73 ) | 2.19 ( 1.97 to 2.41 ) | 553.4 ( 501.88 to 604.94 ) | 949.72 ( 933.1 to 964.19 ) | 91480.82 ( 90438.2 to 92423.2 ) | 131.14 ( 111.62 to 152.1 ) | 19689.46 ( 17222.12 to 22357.74 ) |
| Fiji | 14.38 ( 13.61 to 15.14 ) | 159092.38 ( 151223.06 to 167071.03 ) | 0.06 ( 0.05 to 0.08 ) | 1011.44 ( 841.81 to 1210.79 ) | 8.22 ( 8.12 to 8.32 ) | 90370.88 ( 89235.9 to 91461.33 ) | 2.53 ( 2.11 to 3.03 ) | 31693.75 ( 26586.67 to 37599.13 ) |
| Finland | 113 ( 108.78 to 117.29 ) | 190825.24 ( 182378.05 to 199490.84 ) | 0.52 ( 0.5 to 0.54 ) | 385.23 ( 372.93 to 398.32 ) | 51.69 ( 51.34 to 52.04 ) | 88980.76 ( 87952.45 to 90068.45 ) | 14.58 ( 12.83 to 16.54 ) | 16427.51 ( 13963.37 to 19205.2 ) |
| France | 1257.63 ( 1213.55 to 1307.02 ) | 177394.33 ( 169650.66 to 185463.8 ) | 5.24 ( 5.13 to 5.37 ) | 328.15 ( 321.22 to 336.03 ) | 612.81 ( 607.84 to 617.59 ) | 88711.06 ( 87628.47 to 89813.72 ) | 153.35 ( 133.29 to 175.56 ) | 15461.17 ( 13059.12 to 18229.56 ) |
| Gabon | 30.72 ( 28.96 to 32.56 ) | 179374.63 ( 170320.89 to 188863.63 ) | 0.06 ( 0.05 to 0.08 ) | 713.47 ( 601.2 to 819.66 ) | 16.16 ( 15.99 to 16.34 ) | 93385.93 ( 92542.28 to 94259.76 ) | 3.03 ( 2.48 to 3.57 ) | 23685.98 ( 19688.07 to 27537.98 ) |
| Gambia | 38.6 ( 36.09 to 41.26 ) | 179974.8 ( 170742.37 to 189888.55 ) | 0.06 ( 0.05 to 0.07 ) | 674.07 ( 559.4 to 788.09 ) | 19.87 ( 19.52 to 20.19 ) | 91666.14 ( 90570.82 to 92692.46 ) | 3.3 ( 2.7 to 3.92 ) | 23228.58 ( 19395.26 to 27199.02 ) |
| Georgia | 60.77 ( 58.2 to 63.53 ) | 157342.48 ( 149701.6 to 165510.43 ) | 0.46 ( 0.4 to 0.52 ) | 731.02 ( 636.92 to 833.14 ) | 33.99 ( 33.67 to 34.29 ) | 90050.92 ( 88805.29 to 91165.87 ) | 12.22 ( 10.67 to 13.86 ) | 23376.56 ( 20363.69 to 26527.3 ) |
| Germany | 1738.92 ( 1677.78 to 1801.75 ) | 189253.87 ( 180942.06 to 197761.72 ) | 8.86 ( 8.72 to 9.03 ) | 418.62 ( 411.73 to 426.55 ) | 799.19 ( 794.28 to 803.75 ) | 89233.58 ( 88175.9 to 90178.49 ) | 243.52 ( 214.81 to 275.35 ) | 17277.05 ( 14709.89 to 20233.14 ) |
| Ghana | 512.63 ( 480.98 to 544.75 ) | 167467.16 ( 158247.61 to 176468.91 ) | 0.96 ( 0.82 to 1.13 ) | 661.86 ( 584.48 to 760.67 ) | 284.98 ( 281.37 to 288.77 ) | 92185.87 ( 91261.82 to 93162.89 ) | 49.93 ( 41.92 to 58.53 ) | 22702.9 ( 19467.27 to 26374.62 ) |
| Greece | 203.38 ( 195.67 to 211.47 ) | 180675.09 ( 172451.53 to 189050.81 ) | 1.18 ( 1.16 to 1.21 ) | 423.98 ( 414.28 to 434.99 ) | 97.44 ( 96.8 to 98.05 ) | 89545.43 ( 88391.61 to 90668.88 ) | 30.19 ( 26.8 to 33.97 ) | 17222.36 ( 14700.04 to 20067.59 ) |
| Greenland | 0.83 ( 0.79 to 0.86 ) | 145149.04 ( 137860.76 to 152435.71 ) | 0 ( 0 to 0 ) | 646.17 ( 546.68 to 746.64 ) | 0.51 ( 0.51 to 0.52 ) | 88670.26 ( 87415.16 to 89901.09 ) | 0.16 ( 0.13 to 0.18 ) | 23733.83 ( 20048.76 to 27938.03 ) |
| Grenada | 1.88 ( 1.81 to 1.96 ) | 181467.07 ( 173235.05 to 189933.73 ) | 0.01 ( 0.01 to 0.01 ) | 695.11 ( 652.3 to 739.4 ) | 0.96 ( 0.95 to 0.97 ) | 91621.23 ( 90550.06 to 92641.74 ) | 0.26 ( 0.23 to 0.29 ) | 23589.56 ( 20939.4 to 26408.65 ) |
| Guam | 2.71 ( 2.57 to 2.85 ) | 158562.13 ( 149946.43 to 167038.79 ) | 0.01 ( 0.01 to 0.01 ) | 519.52 ( 448.79 to 600.5 ) | 1.55 ( 1.53 to 1.57 ) | 90387.31 ( 89224.75 to 91451.84 ) | 0.38 ( 0.32 to 0.44 ) | 20657.79 ( 17718.39 to 24064.82 ) |
| Guatemala | 291.4 ( 275.52 to 307.87 ) | 168568.81 ( 160506.67 to 177168.97 ) | 0.6 ( 0.48 to 0.73 ) | 573.11 ( 468.29 to 692.06 ) | 156.31 ( 153.68 to 158.88 ) | 89570.12 ( 88300.92 to 90805.02 ) | 30.15 ( 24.94 to 36.22 ) | 21808.7 ( 18122.39 to 26023.51 ) |
| Guinea | 217.34 ( 201.97 to 233.3 ) | 179536.67 ( 170691.1 to 189099.06 ) | 0.41 ( 0.33 to 0.5 ) | 706.52 ( 587.97 to 847.05 ) | 113.41 ( 111.6 to 115.18 ) | 93092.59 ( 92209.54 to 93987.67 ) | 21.69 ( 17.34 to 26.94 ) | 25112.67 ( 20952.58 to 30174.3 ) |
| Guinea-Bissau | 32.45 ( 30.33 to 34.73 ) | 178692.42 ( 169360.91 to 188131.09 ) | 0.06 ( 0.05 to 0.07 ) | 817.45 ( 674.78 to 974.75 ) | 16.9 ( 16.6 to 17.18 ) | 92099.06 ( 91013.04 to 93124.4 ) | 3.2 ( 2.61 to 3.89 ) | 27689.55 ( 23217.44 to 32664.93 ) |
| Guyana | 13.91 ( 13.27 to 14.59 ) | 183422.58 ( 175022.31 to 192396.52 ) | 0.05 ( 0.04 to 0.06 ) | 870.52 ( 706.28 to 1055.5 ) | 7.08 ( 7 to 7.15 ) | 91814.11 ( 90725.79 to 92778.94 ) | 2 ( 1.65 to 2.4 ) | 29197.85 ( 24143.56 to 34924.27 ) |
| Haiti | 216.41 ( 204.61 to 228.13 ) | 180701.63 ( 172244.67 to 189247.63 ) | 0.59 ( 0.47 to 0.76 ) | 902.5 ( 734.13 to 1137.38 ) | 113.43 ( 111.97 to 114.79 ) | 92817.13 ( 91843 to 93737.26 ) | 28.74 ( 23.86 to 34.72 ) | 30689.56 ( 25479.23 to 37067 ) |
| Honduras | 159.3 ( 150.83 to 168.42 ) | 167106.98 ( 159712.37 to 175453.14 ) | 0.4 ( 0.36 to 0.47 ) | 740.88 ( 690.21 to 839.5 ) | 86.21 ( 84.74 to 87.62 ) | 89639.23 ( 88367.48 to 90855.69 ) | 17.27 ( 14.7 to 20.53 ) | 23995.82 ( 20840.38 to 27928.74 ) |
| Hungary | 173.69 ( 167.28 to 180.31 ) | 164542.76 ( 157280.95 to 172540.15 ) | 1.21 ( 1.03 to 1.42 ) | 615.22 ( 522.51 to 723.19 ) | 91.04 ( 90.37 to 91.68 ) | 89998.57 ( 88747.73 to 91131.94 ) | 32.47 ( 27.78 to 37.63 ) | 20458.06 ( 17349.6 to 23915.42 ) |
| Iceland | 6.15 ( 5.9 to 6.42 ) | 170148.59 ( 161755.58 to 178630.24 ) | 0.02 ( 0.02 to 0.02 ) | 306.07 ( 283.58 to 331.7 ) | 3.18 ( 3.15 to 3.21 ) | 89147.98 ( 88074.6 to 90209.54 ) | 0.67 ( 0.57 to 0.78 ) | 14844.95 ( 12379.22 to 17681.91 ) |
| India | 24781.42 ( 23722.77 to 25834.42 ) | 179932.37 ( 172191.19 to 187751.55 ) | 60.99 ( 53.41 to 68.87 ) | 604.42 ( 530.69 to 680.5 ) | 12876.65 ( 12759.17 to 12988.97 ) | 92463.41 ( 91588.92 to 93279.15 ) | 2710.97 ( 2353.2 to 3138.07 ) | 22071.57 ( 19365.08 to 25376.52 ) |
| Indonesia | 4128.95 ( 3932.8 to 4339.74 ) | 160179.56 ( 152328.44 to 168406.3 ) | 13.28 ( 11.4 to 14.75 ) | 745.6 ( 642.86 to 806.65 ) | 2378.42 ( 2354.95 to 2400.85 ) | 90839.97 ( 89819.66 to 91796.45 ) | 560.15 ( 485.94 to 634.68 ) | 24284.06 ( 21341.24 to 27271.14 ) |
| Iran (Islamic Republic of) | 1412.35 ( 1349.69 to 1476.42 ) | 165910.59 ( 158062.61 to 173639.27 ) | 3.27 ( 3.18 to 3.36 ) | 508.93 ( 496.46 to 522.71 ) | 788.55 ( 781.36 to 794.53 ) | 92646.45 ( 91678.34 to 93466.63 ) | 154.95 ( 132.29 to 180.79 ) | 19632.9 ( 17000.07 to 22614.3 ) |
| Iraq | 650.68 ( 612.85 to 687.26 ) | 158147.19 ( 150221.23 to 165968 ) | 1.38 ( 1.14 to 1.63 ) | 709.21 ( 599.08 to 813.96 ) | 381.2 ( 375.98 to 386.11 ) | 91738 ( 90643.99 to 92745.61 ) | 67.8 ( 56.49 to 80.69 ) | 23229.03 ( 19515.38 to 27274.56 ) |
| Ireland | 92.03 ( 88.22 to 96.03 ) | 179953.01 ( 171022.28 to 188790.66 ) | 0.29 ( 0.28 to 0.3 ) | 383.7 ( 370.9 to 397.89 ) | 44.93 ( 44.57 to 45.31 ) | 88662.69 ( 87671.05 to 89668.14 ) | 10.47 ( 8.99 to 12.13 ) | 16792.22 ( 14165.72 to 19734.61 ) |
| Israel | 174.01 ( 166.74 to 181.37 ) | 185293.54 ( 177323.56 to 193279.7 ) | 0.43 ( 0.42 to 0.44 ) | 346.12 ( 337.35 to 355.61 ) | 84.33 ( 83.48 to 85.23 ) | 90366.36 ( 89428.43 to 91365.93 ) | 15.7 ( 13.43 to 18.31 ) | 15090.44 ( 12748.72 to 17823.95 ) |
| Italy | 1139.94 ( 1099.63 to 1187.07 ) | 173247.62 ( 165375.41 to 181145.75 ) | 6 ( 5.94 to 6.07 ) | 352.55 ( 348.89 to 356.29 ) | 576.3 ( 573.64 to 579.07 ) | 91315.43 ( 90446.75 to 92322.75 ) | 163.14 ( 142.17 to 187.02 ) | 15752.5 ( 13159.38 to 18645.54 ) |
| Jamaica | 51.15 ( 49.07 to 53.33 ) | 180696.08 ( 172439.61 to 189303.23 ) | 0.17 ( 0.14 to 0.21 ) | 543.78 ( 446.31 to 657.07 ) | 26.24 ( 25.99 to 26.45 ) | 92143.37 ( 91141.67 to 93091.74 ) | 5.95 ( 4.97 to 7.04 ) | 20140.32 ( 16787.41 to 23872.28 ) |
| Japan | 2328.79 ( 2246.23 to 2420.95 ) | 170577.15 ( 163440.55 to 178159.68 ) | 11.97 ( 11.73 to 12.35 ) | 268.39 ( 263.99 to 275.26 ) | 1204.46 ( 1197.7 to 1210.93 ) | 88866.71 ( 87800.85 to 89927.69 ) | 312.5 ( 273.6 to 355.49 ) | 13027.95 ( 10894.25 to 15491.95 ) |
| Jordan | 179.16 ( 168.76 to 189.55 ) | 157108.71 ( 149223.72 to 165318 ) | 0.26 ( 0.23 to 0.3 ) | 504.77 ( 443.62 to 577.73 ) | 105.75 ( 104.34 to 107.14 ) | 91755.01 ( 90678.12 to 92828.26 ) | 16.05 ( 13.25 to 19.18 ) | 18842.24 ( 15971.63 to 22057.5 ) |
| Kazakhstan | 293.6 ( 278.72 to 308.46 ) | 159726.44 ( 151465.89 to 167675.4 ) | 1.18 ( 1.06 to 1.32 ) | 805.99 ( 727.37 to 892.01 ) | 165.81 ( 163.83 to 167.69 ) | 89831.01 ( 88716.56 to 90859.41 ) | 43.73 ( 38.72 to 49.48 ) | 24916.93 ( 22179.86 to 28049.12 ) |
| Kenya | 839.66 ( 792.53 to 884.8 ) | 172883.54 ( 165097.3 to 180804.32 ) | 1.15 ( 1 to 1.3 ) | 595.97 ( 531.91 to 663.61 ) | 452.73 ( 447.3 to 457.94 ) | 92289.18 ( 91453.07 to 93086.69 ) | 65.14 ( 55.15 to 75.98 ) | 20749.39 ( 18048.17 to 23680.52 ) |
| Kiribati | 1.84 ( 1.72 to 1.95 ) | 157186.91 ( 149041.83 to 165843.04 ) | 0.01 ( 0.01 to 0.01 ) | 1312.36 ( 1103.26 to 1525.95 ) | 1.06 ( 1.05 to 1.08 ) | 91344.2 ( 90209.49 to 92373.09 ) | 0.36 ( 0.3 to 0.43 ) | 42018.83 ( 35365.11 to 49147.69 ) |
| Kuwait | 72.26 ( 68.61 to 75.95 ) | 160702.55 ( 152589.52 to 168777.33 ) | 0.08 ( 0.07 to 0.09 ) | 362.47 ( 316.76 to 416.72 ) | 41.12 ( 40.73 to 41.5 ) | 90737.49 ( 89505.66 to 91891.88 ) | 6.09 ( 4.96 to 7.33 ) | 16280.53 ( 13665.95 to 19242.77 ) |
| Kyrgyzstan | 101.09 ( 95.78 to 106.96 ) | 158168.53 ( 150559.11 to 167099.41 ) | 0.29 ( 0.26 to 0.32 ) | 715.95 ( 648.75 to 789.33 ) | 58.36 ( 57.48 to 59.17 ) | 90283.11 ( 89092.55 to 91386.58 ) | 11.72 ( 10.34 to 13.36 ) | 22541.78 ( 19991.15 to 25461.4 ) |
| Lao People's Democratic Republic | 111.17 ( 105.2 to 117 ) | 158098.37 ( 150074.85 to 166101.18 ) | 0.29 ( 0.24 to 0.35 ) | 764.88 ( 652.05 to 875.89 ) | 64.47 ( 63.66 to 65.21 ) | 91080.05 ( 90069.43 to 92009.01 ) | 13.57 ( 11.32 to 16.08 ) | 25287.21 ( 21441.96 to 29431.35 ) |
| Latvia | 36.33 ( 34.84 to 37.84 ) | 171585.38 ( 163307.86 to 179870.36 ) | 0.25 ( 0.22 to 0.29 ) | 605.04 ( 527.82 to 706.14 ) | 17.95 ( 17.79 to 18.09 ) | 89602.26 ( 88220.58 to 90833.39 ) | 6.52 ( 5.66 to 7.52 ) | 20565.5 ( 17662.26 to 23817.02 ) |
| Lebanon | 84.94 ( 80.52 to 89.55 ) | 162708.47 ( 154210.66 to 171601.05 ) | 0.3 ( 0.28 to 0.33 ) | 596.48 ( 542.71 to 651.39 ) | 47.47 ( 46.93 to 47.98 ) | 91328.09 ( 90202.68 to 92352.37 ) | 11.2 ( 9.65 to 12.97 ) | 21417.97 ( 18457.78 to 24779.84 ) |
| Lesotho | 38.8 ( 36.83 to 40.82 ) | 190604.55 ( 181919.75 to 199372.26 ) | 0.11 ( 0.08 to 0.14 ) | 978.94 ( 767.19 to 1209.8 ) | 18.71 ( 18.44 to 18.98 ) | 90571.61 ( 89424.26 to 91726.66 ) | 4.54 ( 3.68 to 5.49 ) | 30424.25 ( 24861.7 to 36750.69 ) |
| Liberia | 82.32 ( 77.14 to 87.44 ) | 178676.72 ( 169752.35 to 187668.34 ) | 0.11 ( 0.09 to 0.14 ) | 585.11 ( 482.58 to 722.63 ) | 43.48 ( 42.89 to 44.06 ) | 92925.92 ( 91994.47 to 93847.97 ) | 6.57 ( 5.24 to 7.92 ) | 20875.69 ( 17142.88 to 25166.16 ) |
| Libya | 108.57 ( 102.78 to 114.09 ) | 160452.24 ( 151855.92 to 168868.4 ) | 0.25 ( 0.21 to 0.3 ) | 541.83 ( 451.92 to 646.76 ) | 63.25 ( 62.7 to 63.76 ) | 92689.98 ( 91696.38 to 93614.7 ) | 12.61 ( 10.54 to 14.99 ) | 21167.49 ( 17800.39 to 24995.68 ) |
| Lithuania | 53.55 ( 51.51 to 55.59 ) | 173543 ( 165343.1 to 182073.6 ) | 0.35 ( 0.3 to 0.41 ) | 581.02 ( 493.81 to 680.19 ) | 26.31 ( 26.1 to 26.5 ) | 90120.81 ( 88831.15 to 91281.46 ) | 9.14 ( 7.85 to 10.56 ) | 20070.27 ( 17138.42 to 23240.76 ) |
| Luxembourg | 11.94 ( 11.52 to 12.39 ) | 182068.72 ( 173430.45 to 190458.14 ) | 0.04 ( 0.03 to 0.04 ) | 341.91 ( 310.3 to 379.48 ) | 5.74 ( 5.7 to 5.78 ) | 88745.1 ( 87716.12 to 89800.98 ) | 1.32 ( 1.12 to 1.54 ) | 15739.53 ( 13160.96 to 18606.44 ) |
| Madagascar | 437.28 ( 409.05 to 466.23 ) | 170248.78 ( 161544.97 to 179286.04 ) | 0.71 ( 0.57 to 0.88 ) | 722.63 ( 589.54 to 880.29 ) | 239.01 ( 235.42 to 242.55 ) | 92283.98 ( 91302.19 to 93229.26 ) | 40.45 ( 33.46 to 47.77 ) | 24476.95 ( 20382.96 to 28977.62 ) |
| Malawi | 305.6 ( 284.34 to 327.48 ) | 171308.54 ( 162156.52 to 180034.96 ) | 0.44 ( 0.37 to 0.52 ) | 621.05 ( 535.63 to 718.03 ) | 162.71 ( 159.92 to 165.42 ) | 91522.69 ( 90481.74 to 92570.92 ) | 25.16 ( 21.09 to 29.57 ) | 22082.07 ( 18949.64 to 25567.95 ) |
| Malaysia | 490.77 ( 466.07 to 516.15 ) | 157743.63 ( 149529.81 to 166367.32 ) | 1.3 ( 1.06 to 1.58 ) | 555.25 ( 459.19 to 666.87 ) | 280.63 ( 277.31 to 283.83 ) | 88956.94 ( 87811.33 to 90082.64 ) | 56.94 ( 47.73 to 67.42 ) | 19936.16 ( 16766.83 to 23479.74 ) |
| Maldives | 7.72 ( 7.35 to 8.12 ) | 157464.07 ( 149728.38 to 165787.71 ) | 0.01 ( 0.01 to 0.01 ) | 455.66 ( 395.17 to 520.1 ) | 4.56 ( 4.51 to 4.6 ) | 90641 ( 89592.8 to 91641.91 ) | 0.67 ( 0.56 to 0.78 ) | 16863.35 ( 14380.52 to 19579.74 ) |
| Mali | 386.67 ( 359.07 to 414.57 ) | 183819.47 ( 174178.3 to 193794.93 ) | 0.58 ( 0.46 to 0.73 ) | 639.63 ( 539.71 to 766.35 ) | 194.03 ( 190.32 to 197.57 ) | 92635.66 ( 91592.71 to 93614.29 ) | 34.84 ( 26.79 to 44.09 ) | 23054.99 ( 19163.95 to 27563.21 ) |
| Malta | 8.34 ( 8.03 to 8.65 ) | 175790.42 ( 167476.38 to 183795.9 ) | 0.03 ( 0.03 to 0.04 ) | 354.06 ( 323.91 to 386.34 ) | 4.1 ( 4.07 to 4.13 ) | 88692.36 ( 87596.19 to 89760.81 ) | 1.08 ( 0.93 to 1.25 ) | 15953.23 ( 13411.48 to 18838.5 ) |
| Marshall Islands | 0.88 ( 0.83 to 0.93 ) | 156834.08 ( 148486.28 to 164937.24 ) | 0 ( 0 to 0 ) | 1096.44 ( 896.12 to 1323.62 ) | 0.51 ( 0.5 to 0.52 ) | 90727.43 ( 89541.68 to 91805.15 ) | 0.15 ( 0.12 to 0.18 ) | 34967.81 ( 29009.91 to 41736.63 ) |
| Mauritania | 68.91 ( 64.36 to 73.39 ) | 177805.45 ( 168397.26 to 186835.42 ) | 0.1 ( 0.08 to 0.12 ) | 522.73 ( 434.81 to 617.85 ) | 35.92 ( 35.36 to 36.48 ) | 92090.71 ( 91087.59 to 93124.37 ) | 5.12 ( 4.11 to 6.27 ) | 18648.31 ( 15252.12 to 22419.96 ) |
| Mauritius | 20.74 ( 19.87 to 21.7 ) | 160002.84 ( 151747.26 to 168235.83 ) | 0.1 ( 0.08 to 0.12 ) | 596.03 ( 498.07 to 709.37 ) | 11.87 ( 11.77 to 11.96 ) | 89856.23 ( 88744.43 to 90949.1 ) | 3.56 ( 3 to 4.17 ) | 22359.16 ( 18861.18 to 26247.4 ) |
| Mexico | 2229.87 ( 2143.93 to 2326.14 ) | 179042.5 ( 171813.91 to 187057.4 ) | 5.98 ( 5.26 to 6.74 ) | 540.74 ( 478.19 to 607.85 ) | 1115.11 ( 1101.55 to 1127.72 ) | 88538.54 ( 87353.12 to 89633.65 ) | 254.69 ( 221.37 to 294.22 ) | 21022.82 ( 18320.9 to 24173.91 ) |
| Micronesia (Federated States of) | 1.58 ( 1.49 to 1.66 ) | 156734.18 ( 148926.37 to 165141.41 ) | 0.01 ( 0.01 to 0.01 ) | 1203.99 ( 963.49 to 1422.8 ) | 0.92 ( 0.91 to 0.93 ) | 90730.9 ( 89507.45 to 91830.49 ) | 0.3 ( 0.23 to 0.36 ) | 36712.78 ( 28609.55 to 43786.11 ) |
| Monaco | 0.74 ( 0.71 to 0.77 ) | 179693.3 ( 171164.89 to 188240.87 ) | 0 ( 0 to 0.01 ) | 445.87 ( 371.46 to 507.09 ) | 0.35 ( 0.35 to 0.36 ) | 88760.89 ( 87681.96 to 89737.36 ) | 0.12 ( 0.1 to 0.14 ) | 18085.99 ( 15031.77 to 21454.73 ) |
| Mongolia | 53.13 ( 50.35 to 56.12 ) | 159985.01 ( 151988.58 to 168429.36 ) | 0.2 ( 0.16 to 0.25 ) | 1066.05 ( 885.47 to 1287 ) | 30.45 ( 30.03 to 30.83 ) | 90430.83 ( 89213.4 to 91479.37 ) | 8.35 ( 6.89 to 9.99 ) | 30697.14 ( 25631.87 to 36355.22 ) |
| Montenegro | 10.63 ( 10.16 to 11.09 ) | 163077.43 ( 155110.55 to 171610.58 ) | 0.06 ( 0.06 to 0.07 ) | 696.91 ( 614.29 to 793.06 ) | 5.78 ( 5.73 to 5.83 ) | 90078.01 ( 88805.32 to 91216.87 ) | 1.81 ( 1.57 to 2.07 ) | 20739.64 ( 17939.79 to 23961.86 ) |
| Morocco | 579.56 ( 547.82 to 609.92 ) | 161312.19 ( 152699.76 to 169656.69 ) | 1.89 ( 1.55 to 2.14 ) | 722.26 ( 603.46 to 795.72 ) | 331.23 ( 327.32 to 334.56 ) | 91619.17 ( 90441.31 to 92634.19 ) | 75.97 ( 63.59 to 89.18 ) | 23380.86 ( 19760.03 to 27081.81 ) |
| Mozambique | 489.42 ( 454.43 to 524.44 ) | 172596.67 ( 163471.02 to 181766.62 ) | 0.76 ( 0.61 to 0.93 ) | 705.76 ( 586.95 to 858.05 ) | 258.85 ( 253.79 to 263.84 ) | 91801.29 ( 90701.67 to 92865.36 ) | 43.87 ( 36.22 to 52.85 ) | 24334.45 ( 20482.48 to 29040.55 ) |
| Myanmar | 841.04 ( 794.84 to 887.47 ) | 155095.59 ( 146570.29 to 164027.07 ) | 3.17 ( 2.8 to 3.68 ) | 774.22 ( 700.56 to 877.93 ) | 496.19 ( 490.71 to 501.68 ) | 90613.03 ( 89601.04 to 91660.32 ) | 126.99 ( 108.22 to 148.33 ) | 25830.84 ( 22271.55 to 29913.05 ) |
| Namibia | 44.17 ( 41.75 to 46.54 ) | 189309.49 ( 180501.31 to 198679.12 ) | 0.09 ( 0.07 to 0.11 ) | 691.5 ( 581.39 to 835.64 ) | 21.22 ( 20.9 to 21.55 ) | 90309.11 ( 89202.89 to 91455.28 ) | 3.82 ( 3.09 to 4.64 ) | 22518.33 ( 18576.54 to 26992.37 ) |
| Nauru | 0.16 ( 0.15 to 0.17 ) | 158038.17 ( 149575.61 to 166271.99 ) | 0 ( 0 to 0 ) | 1273.71 ( 1119.14 to 1449.08 ) | 0.09 ( 0.09 to 0.09 ) | 90634.13 ( 89463.79 to 91755.84 ) | 0.02 ( 0.02 to 0.03 ) | 39308.13 ( 34468.53 to 45147.15 ) |
| Nepal | 522.21 ( 498.3 to 546.58 ) | 175215.32 ( 167970.85 to 183153.98 ) | 1.38 ( 1.16 to 1.55 ) | 721.21 ( 611.1 to 799.66 ) | 269.48 ( 265.77 to 273.03 ) | 89430.8 ( 88340.59 to 90479.82 ) | 56.86 ( 48.02 to 65.44 ) | 23282.38 ( 19807.84 to 26562.33 ) |
| Netherlands | 326.02 ( 313.38 to 339.52 ) | 178149.12 ( 170470.93 to 186501.38 ) | 1.41 ( 1.38 to 1.45 ) | 392.71 ( 383.08 to 403.54 ) | 159.4 ( 158.3 to 160.43 ) | 88563.28 ( 87580.11 to 89512.73 ) | 42.44 ( 37.27 to 48.24 ) | 16214.98 ( 13796.71 to 19002.46 ) |
| New Zealand | 89.38 ( 86.12 to 92.95 ) | 189013.14 ( 180605.84 to 197650.99 ) | 0.32 ( 0.31 to 0.32 ) | 381.88 ( 375.31 to 388.49 ) | 40.7 ( 40.39 to 41.03 ) | 86973.82 ( 86016.44 to 87972.44 ) | 10.15 ( 8.88 to 11.58 ) | 16536.73 ( 14139.86 to 19281.1 ) |
| Nicaragua | 106.77 ( 101.14 to 112.5 ) | 167225.94 ( 158948.92 to 175523 ) | 0.24 ( 0.2 to 0.27 ) | 624.58 ( 538.75 to 701.41 ) | 57.63 ( 56.74 to 58.46 ) | 89362.61 ( 88093.95 to 90557.65 ) | 10.75 ( 9.05 to 12.62 ) | 20984.36 ( 17880.19 to 24403.78 ) |
| Niger | 398.59 ( 372.56 to 427.62 ) | 181450.58 ( 172560.09 to 190825.18 ) | 0.5 ( 0.39 to 0.64 ) | 612.79 ( 513.44 to 730.63 ) | 206.85 ( 203.54 to 210.05 ) | 93309.31 ( 92475.47 to 94121.92 ) | 32.86 ( 25.21 to 42.19 ) | 22014.67 ( 18540 to 26453.72 ) |
| Nigeria | 4006.59 ( 3758.61 to 4275.22 ) | 193636.47 ( 184626.7 to 203397.69 ) | 4.84 ( 3.94 to 5.78 ) | 567.09 ( 474.97 to 664.63 ) | 1975.99 ( 1953.74 to 1998.2 ) | 94398.5 ( 93690.58 to 95104.49 ) | 310.5 ( 252.2 to 369.82 ) | 21141.61 ( 17769.49 to 24745.69 ) |
| Niue | 0.03 ( 0.03 to 0.03 ) | 158018.86 ( 149921.38 to 166831.82 ) | 0 ( 0 to 0 ) | 827.21 ( 707.84 to 965.24 ) | 0.02 ( 0.02 to 0.02 ) | 90464.29 ( 89298.95 to 91554.65 ) | 0.01 ( 0 to 0.01 ) | 27636.19 ( 23589.01 to 32186.08 ) |
| North Macedonia | 36.37 ( 34.77 to 38.03 ) | 162234.75 ( 154330.24 to 170480.08 ) | 0.23 ( 0.19 to 0.28 ) | 876.61 ( 739.23 to 1035.48 ) | 20.11 ( 19.93 to 20.27 ) | 90087.54 ( 88827.25 to 91275.71 ) | 6.56 ( 5.49 to 7.73 ) | 23171.13 ( 19562.31 to 27168.34 ) |
| Northern Mariana Islands | 0.66 ( 0.63 to 0.69 ) | 157570.87 ( 149557.53 to 165783 ) | 0 ( 0 to 0 ) | 722.21 ( 639.5 to 809.99 ) | 0.4 ( 0.39 to 0.4 ) | 90562.91 ( 89392.18 to 91618.33 ) | 0.12 ( 0.11 to 0.14 ) | 24166.6 ( 20888.71 to 27655.24 ) |
| Norway | 107.63 ( 103.71 to 111.68 ) | 190333.6 ( 181888.21 to 198569.89 ) | 0.36 ( 0.36 to 0.37 ) | 341.68 ( 335.5 to 349.17 ) | 50.11 ( 49.78 to 50.48 ) | 90380.7 ( 89403.81 to 91460.96 ) | 11.82 ( 10.21 to 13.72 ) | 15642.44 ( 13123.18 to 18526.96 ) |
| Oman | 69.89 ( 66.22 to 73.64 ) | 155116.66 ( 147450.99 to 163104.96 ) | 0.09 ( 0.08 to 0.09 ) | 763.37 ( 712.29 to 815.17 ) | 42.92 ( 42.51 to 43.31 ) | 93211.96 ( 92255.74 to 94122.48 ) | 5.83 ( 4.88 to 6.92 ) | 22001.96 ( 19605.09 to 24836.5 ) |
| Pakistan | 3876.99 ( 3657.63 to 4087.97 ) | 179214.6 ( 171467.43 to 187019.71 ) | 8.3 ( 7.19 to 9.68 ) | 814.48 ( 712.88 to 942.91 ) | 1934.03 ( 1898.35 to 1964.75 ) | 89525.68 ( 88341.9 to 90567.85 ) | 411.96 ( 355.83 to 473.68 ) | 27623.54 ( 24126.11 to 31700.97 ) |
| Palau | 0.28 ( 0.27 to 0.3 ) | 157263.72 ( 148767.05 to 165564.42 ) | 0 ( 0 to 0 ) | 917.08 ( 753.18 to 1097.07 ) | 0.17 ( 0.17 to 0.17 ) | 90325.89 ( 89121.97 to 91448.52 ) | 0.06 ( 0.05 to 0.08 ) | 29982.28 ( 24915.55 to 35513.26 ) |
| Palestine | 78.55 ( 74.02 to 82.88 ) | 163558.93 ( 156090.2 to 171548.92 ) | 0.14 ( 0.12 to 0.15 ) | 716.43 ( 642.68 to 795.53 ) | 44.23 ( 43.63 to 44.83 ) | 91400.96 ( 90454.43 to 92359.53 ) | 7.21 ( 6.13 to 8.45 ) | 22924 ( 19948.92 to 26232.9 ) |
| Panama | 69.76 ( 66.41 to 73.22 ) | 167542.73 ( 159458.91 to 175973.83 ) | 0.16 ( 0.12 to 0.19 ) | 367.79 ( 293.19 to 458 ) | 37.36 ( 36.84 to 37.85 ) | 89497.07 ( 88226.43 to 90691.57 ) | 6.89 ( 5.68 to 8.39 ) | 16552.33 ( 13657.28 to 20161.33 ) |
| Papua New Guinea | 152 ( 142.41 to 161.75 ) | 157287.53 ( 148878.3 to 166101.16 ) | 0.39 ( 0.31 to 0.49 ) | 901.28 ( 749.31 to 1085.05 ) | 87.88 ( 86.49 to 89.16 ) | 91394.11 ( 90299.9 to 92406.89 ) | 20.17 ( 16.35 to 24.59 ) | 30048.11 ( 24881.05 to 36072.49 ) |
| Paraguay | 129.2 ( 123.45 to 135.21 ) | 188810.87 ( 180734.83 to 197555.75 ) | 0.26 ( 0.21 to 0.33 ) | 491.01 ( 389.93 to 609.24 ) | 62.67 ( 61.84 to 63.41 ) | 90519.49 ( 89337.21 to 91573.21 ) | 12.14 ( 9.98 to 14.74 ) | 19730.56 ( 16235.51 to 23707.88 ) |
| Peru | 650.35 ( 621.51 to 682.69 ) | 191597.67 ( 183033.26 to 201141.58 ) | 1.08 ( 0.83 to 1.37 ) | 331.45 ( 255.84 to 421.34 ) | 310.7 ( 306.72 to 314.31 ) | 91137.05 ( 89922.54 to 92238.39 ) | 50.95 ( 41.36 to 62.08 ) | 15303.04 ( 12445.86 to 18602.91 ) |
| Philippines | 1869.32 ( 1773.97 to 1966.09 ) | 169584.5 ( 161744.77 to 177868.67 ) | 4.44 ( 3.76 to 5.17 ) | 635.11 ( 542.88 to 733.73 ) | 1000.04 ( 986.34 to 1012.1 ) | 90224.18 ( 89114.49 to 91194.32 ) | 211.02 ( 180.61 to 244.22 ) | 23163.47 ( 19932.29 to 26614.2 ) |
| Poland | 614.26 ( 592.16 to 640.7 ) | 149538.57 ( 142253.66 to 157400.95 ) | 3.71 ( 3.2 to 4.24 ) | 520.64 ( 448.01 to 596.51 ) | 357.59 ( 354.82 to 360.15 ) | 88698.36 ( 87465.99 to 89827.2 ) | 106.92 ( 92.11 to 123.06 ) | 18312.98 ( 15640.21 to 21392.71 ) |
| Portugal | 190.92 ( 183.63 to 198.77 ) | 164445.23 ( 156346.77 to 172280.55 ) | 1.02 ( 0.99 to 1.05 ) | 375.8 ( 366.47 to 386.12 ) | 100.41 ( 99.81 to 100.97 ) | 89097.57 ( 88081.28 to 90081.46 ) | 29.26 ( 25.59 to 33.33 ) | 16664.49 ( 14085.09 to 19599.02 ) |
| Puerto Rico | 69.09 ( 66.5 to 71.8 ) | 183063.79 ( 174835.99 to 191709.59 ) | 0.29 ( 0.23 to 0.35 ) | 375.8 ( 300.76 to 466.34 ) | 33.47 ( 33.26 to 33.68 ) | 91536.53 ( 90448.68 to 92513.78 ) | 8.96 ( 7.47 to 10.71 ) | 16660.8 ( 13747.49 to 20138.56 ) |
| Qatar | 44.12 ( 41.96 to 46.28 ) | 155672.71 ( 148138.6 to 163609.57 ) | 0.03 ( 0.02 to 0.04 ) | 781.99 ( 657.96 to 924.67 ) | 26.79 ( 26.55 to 27.02 ) | 91166.28 ( 90082.56 to 92211.85 ) | 3.32 ( 2.65 to 4.07 ) | 20983.28 ( 17740.24 to 24618.54 ) |
| Republic of Korea | 884.79 ( 851.6 to 921.72 ) | 163255.43 ( 156094.27 to 171271.92 ) | 2.65 ( 2.55 to 2.78 ) | 319.02 ( 306.54 to 334.36 ) | 499.04 ( 495.67 to 502.26 ) | 88892.63 ( 87707.78 to 89941.67 ) | 101.01 ( 86.78 to 116.76 ) | 13534.56 ( 11432.62 to 15827.79 ) |
| Republic of Moldova | 66.54 ( 63.73 to 69.4 ) | 169236.33 ( 161255.66 to 177429.16 ) | 0.37 ( 0.34 to 0.41 ) | 654.76 ( 590.79 to 725.12 ) | 34.62 ( 34.34 to 34.89 ) | 90191.11 ( 88966.43 to 91403.98 ) | 11.2 ( 9.95 to 12.64 ) | 21961.41 ( 19474.44 to 24885.96 ) |
| Romania | 353.28 ( 339.56 to 367.14 ) | 169361.12 ( 161491.99 to 177509.06 ) | 2.45 ( 2.09 to 2.84 ) | 644.92 ( 550.97 to 750.52 ) | 181.04 ( 179.61 to 182.41 ) | 90362.44 ( 89128.27 to 91516.2 ) | 63.66 ( 54.68 to 73.34 ) | 20643.15 ( 17686.16 to 23857.13 ) |
| Russian Federation | 2776.2 ( 2667.2 to 2889.82 ) | 177409.19 ( 169607.01 to 185424.88 ) | 15.8 ( 14.16 to 17.65 ) | 691.34 ( 619.84 to 771.73 ) | 1366.47 ( 1355.52 to 1376.67 ) | 89925.96 ( 88795.16 to 90941.71 ) | 473.68 ( 419 to 539.29 ) | 23206.69 ( 20403.52 to 26611.89 ) |
| Rwanda | 213.47 ( 200.18 to 227.54 ) | 173615.85 ( 164551.46 to 182993.15 ) | 0.33 ( 0.28 to 0.39 ) | 616.97 ( 551.61 to 714.16 ) | 111.36 ( 109.5 to 113.13 ) | 90359.55 ( 89231.79 to 91449.59 ) | 18.22 ( 15.46 to 21.41 ) | 21414.45 ( 18543.88 to 24764.94 ) |
| Saint Kitts and Nevis | 1.1 ( 1.05 to 1.14 ) | 182668.34 ( 174258.85 to 191751.39 ) | 0 ( 0 to 0 ) | 717.38 ( 635.53 to 819.86 ) | 0.56 ( 0.55 to 0.56 ) | 91414.42 ( 90342.78 to 92412.86 ) | 0.15 ( 0.13 to 0.18 ) | 23475.04 ( 19940.13 to 27293.88 ) |
| Saint Lucia | 3.24 ( 3.11 to 3.38 ) | 181956.23 ( 173192.39 to 190771.92 ) | 0.01 ( 0.01 to 0.01 ) | 588.97 ( 515.63 to 673.39 ) | 1.64 ( 1.63 to 1.65 ) | 91655.65 ( 90562.01 to 92669.88 ) | 0.43 ( 0.37 to 0.5 ) | 21338.41 ( 18378.07 to 24820.89 ) |
| Saint Vincent and the Grenadines | 2.08 ( 2 to 2.17 ) | 181503.3 ( 173644.59 to 189343.08 ) | 0.01 ( 0.01 to 0.01 ) | 695.22 ( 627.17 to 772.21 ) | 1.06 ( 1.05 to 1.07 ) | 92116.08 ( 91101.6 to 93086.13 ) | 0.3 ( 0.26 to 0.34 ) | 23487.29 ( 20539.36 to 26923.86 ) |
| Samoa | 3.29 ( 3.07 to 3.48 ) | 156328.07 ( 147792.02 to 164392.36 ) | 0.01 ( 0.01 to 0.01 ) | 825.12 ( 700.71 to 981.48 ) | 1.89 ( 1.86 to 1.92 ) | 90627 ( 89461.76 to 91691.58 ) | 0.44 ( 0.37 to 0.52 ) | 26856.19 ( 22670.3 to 31690.31 ) |
| San Marino | 0.64 ( 0.61 to 0.67 ) | 180909.74 ( 172247.15 to 190062.5 ) | 0 ( 0 to 0 ) | 371.9 ( 260.15 to 509.92 ) | 0.31 ( 0.31 to 0.31 ) | 88873.37 ( 87814.09 to 89944.96 ) | 0.08 ( 0.06 to 0.1 ) | 16186.85 ( 12710.08 to 20398.03 ) |
| Sao Tome and Principe | 3.53 ( 3.31 to 3.75 ) | 177192.67 ( 168294.88 to 186560.35 ) | 0.01 ( 0.01 to 0.01 ) | 736.95 ( 640.69 to 800.34 ) | 1.86 ( 1.84 to 1.89 ) | 92268.25 ( 91265.63 to 93268.96 ) | 0.33 ( 0.28 to 0.39 ) | 23949.95 ( 20597.35 to 27518.41 ) |
| Saudi Arabia | 552.37 ( 526.43 to 579.32 ) | 154260.38 ( 146980.63 to 161006.85 ) | 0.85 ( 0.7 to 1.01 ) | 607.94 ( 524.38 to 687.69 ) | 336.98 ( 334.7 to 339.13 ) | 93130.33 ( 92378 to 93869.48 ) | 56.14 ( 46.64 to 66.74 ) | 21219.2 ( 18169.55 to 24494.42 ) |
| Senegal | 263.26 ( 246.83 to 279.66 ) | 179659.28 ( 170445.01 to 189177.97 ) | 0.42 ( 0.35 to 0.51 ) | 604.51 ( 516.81 to 717.95 ) | 137.3 ( 135.1 to 139.41 ) | 93157.24 ( 92080.11 to 94156.74 ) | 22.19 ( 18.15 to 26.04 ) | 21401.14 ( 17910.56 to 25176.2 ) |
| Serbia | 150.86 ( 144.79 to 157.45 ) | 160469.07 ( 153047.7 to 168535.05 ) | 1.12 ( 0.93 to 1.33 ) | 765.56 ( 645.63 to 906.55 ) | 82.02 ( 81.31 to 82.67 ) | 90416.3 ( 89159.88 to 91553.22 ) | 28.9 ( 24.32 to 33.83 ) | 21378.14 ( 18027.55 to 25091.91 ) |
| Seychelles | 1.62 ( 1.54 to 1.69 ) | 157963.23 ( 149857.02 to 166542.23 ) | 0.01 ( 0.01 to 0.01 ) | 633.79 ( 582.83 to 689 ) | 0.94 ( 0.93 to 0.95 ) | 89930.52 ( 88774.01 to 91056.13 ) | 0.24 ( 0.21 to 0.28 ) | 22285.68 ( 19598.28 to 25220.86 ) |
| Sierra Leone | 140.9 ( 131.84 to 149.89 ) | 177300.08 ( 168580.38 to 186265.74 ) | 0.24 ( 0.18 to 0.31 ) | 633.68 ( 520.74 to 783.95 ) | 75.25 ( 74.24 to 76.29 ) | 93253.48 ( 92369.98 to 94151.16 ) | 14 ( 10.57 to 18.05 ) | 23375.72 ( 18973.17 to 28808.26 ) |
| Singapore | 92.78 ( 88.86 to 96.75 ) | 163244.98 ( 155116.56 to 171291.11 ) | 0.19 ( 0.18 to 0.19 ) | 256 ( 247.24 to 266.59 ) | 52.4 ( 51.98 to 52.81 ) | 88168.92 ( 86939.62 to 89344.54 ) | 8.72 ( 7.36 to 10.26 ) | 12309.91 ( 10316.91 to 14559.36 ) |
| Slovakia | 99.73 ( 95.75 to 103.74 ) | 172458.25 ( 164855.29 to 180715.83 ) | 0.5 ( 0.41 to 0.6 ) | 557.72 ( 458.03 to 675.86 ) | 51 ( 50.59 to 51.38 ) | 90214.89 ( 88965.58 to 91344.42 ) | 14.8 ( 12.37 to 17.52 ) | 18755.25 ( 15623.64 to 22337.66 ) |
| Slovenia | 38.41 ( 36.92 to 39.92 ) | 168586.57 ( 160844.91 to 176664.3 ) | 0.19 ( 0.15 to 0.23 ) | 392.28 ( 316.77 to 489.44 ) | 19.52 ( 19.37 to 19.66 ) | 89956.54 ( 88648.56 to 91114.98 ) | 5.21 ( 4.33 to 6.24 ) | 15164.38 ( 12510.76 to 18271.8 ) |
| Solomon Islands | 10.05 ( 9.42 to 10.69 ) | 156404.85 ( 148230.03 to 164433.15 ) | 0.04 ( 0.03 to 0.05 ) | 1429.08 ( 1220.83 to 1643.16 ) | 5.84 ( 5.74 to 5.93 ) | 91668.92 ( 90505.65 to 92672.51 ) | 1.86 ( 1.58 to 2.18 ) | 44700.53 ( 38219.47 to 51870.96 ) |
| Somalia | 331.88 ( 308.3 to 355.15 ) | 170515.63 ( 161199.22 to 179291.06 ) | 0.49 ( 0.38 to 0.63 ) | 738.4 ( 587.21 to 924.46 ) | 178.22 ( 174.75 to 181.41 ) | 91762.11 ( 90688.14 to 92766.02 ) | 30.27 ( 24.5 to 37.48 ) | 25354.95 ( 20965.72 to 31036.19 ) |
| South Africa | 1077.77 ( 1031.43 to 1124.27 ) | 195332.38 ( 186999.27 to 203922.68 ) | 2.29 ( 2.17 to 2.41 ) | 582.35 ( 554.41 to 609.66 ) | 506.16 ( 500.68 to 511.29 ) | 90779.43 ( 89750.18 to 91735.91 ) | 102.14 ( 89.59 to 115.99 ) | 20844.5 ( 18495.98 to 23443.16 ) |
| South Sudan | 155.06 ( 144.51 to 166.15 ) | 173895.1 ( 165257.88 to 183358.25 ) | 0.21 ( 0.17 to 0.27 ) | 563.62 ( 452.02 to 678.38 ) | 81.16 ( 79.68 to 82.63 ) | 91294.47 ( 90263.52 to 92358.63 ) | 13.05 ( 10.57 to 15.89 ) | 20799.17 ( 17192.24 to 24829.72 ) |
| Spain | 862.77 ( 830.46 to 895.53 ) | 174113.08 ( 166256.04 to 182151.6 ) | 3.94 ( 3.87 to 4.02 ) | 347.63 ( 341.02 to 354.74 ) | 428.74 ( 425.91 to 431.75 ) | 88213.17 ( 87067.52 to 89398.56 ) | 112.34 ( 97.96 to 128.96 ) | 15453.71 ( 13046.47 to 18340.69 ) |
| Sri Lanka | 349.58 ( 333.32 to 366.22 ) | 158817.84 ( 150553.49 to 166905.91 ) | 1.12 ( 0.87 to 1.42 ) | 508.88 ( 399.38 to 636.81 ) | 200.61 ( 198.47 to 202.62 ) | 90296.27 ( 89117.22 to 91396.55 ) | 44.13 ( 35.98 to 53.35 ) | 18456.53 ( 15109.98 to 22300.26 ) |
| Sudan | 627.87 ( 590.45 to 666.45 ) | 159186.29 ( 151431.08 to 167097.67 ) | 1.3 ( 1.11 to 1.55 ) | 740.38 ( 646.69 to 873.32 ) | 363.24 ( 357.46 to 368.89 ) | 91519.11 ( 90437.65 to 92553.27 ) | 69.87 ( 57.85 to 82.11 ) | 25231.58 ( 21432.66 to 29346.06 ) |
| Suriname | 10.53 ( 10.09 to 11 ) | 181885.31 ( 173922.5 to 190476.78 ) | 0.03 ( 0.03 to 0.04 ) | 600.81 ( 520.53 to 691.9 ) | 5.3 ( 5.25 to 5.36 ) | 91283.09 ( 90187.46 to 92282.23 ) | 1.37 ( 1.17 to 1.6 ) | 23039.81 ( 19770.53 to 26854.77 ) |
| Sweden | 198.39 ( 191.24 to 206.11 ) | 181937.67 ( 173874.61 to 190088.53 ) | 0.85 ( 0.84 to 0.86 ) | 353.31 ( 348.34 to 358.63 ) | 95.21 ( 94.46 to 95.94 ) | 89740.41 ( 88631.74 to 90861.76 ) | 24.03 ( 20.99 to 27.43 ) | 15350.61 ( 12977.86 to 18096.02 ) |
| Switzerland | 168.62 ( 162.48 to 174.97 ) | 178804.92 ( 170099.42 to 187442.63 ) | 0.63 ( 0.61 to 0.65 ) | 311.08 ( 303.38 to 319.67 ) | 81.76 ( 81.2 to 82.35 ) | 88605.42 ( 87515.1 to 89693.08 ) | 19.38 ( 16.7 to 22.39 ) | 14760.75 ( 12347.18 to 17562.86 ) |
| Syrian Arab Republic | 228.39 ( 216.59 to 240.38 ) | 160896.47 ( 152469.18 to 168983.64 ) | 0.69 ( 0.54 to 0.88 ) | 726.6 ( 584.96 to 897.51 ) | 132.66 ( 131.21 to 134.14 ) | 91257.31 ( 90307.85 to 92258.72 ) | 29.25 ( 23.76 to 35.68 ) | 22993.16 ( 18873.46 to 27730.31 ) |
| Taiwan (Province of China) | 358 ( 344.33 to 372.35 ) | 147526.4 ( 139881.34 to 155034.73 ) | 1.57 ( 1.28 to 1.96 ) | 399.9 ( 324.72 to 497.39 ) | 219 ( 217.45 to 220.43 ) | 87903.39 ( 86686.87 to 89047.86 ) | 54.77 ( 45.07 to 65.75 ) | 16170.56 ( 13281.36 to 19368.89 ) |
| Tajikistan | 142.04 ( 134.25 to 150.23 ) | 155939.19 ( 148333.83 to 163866.89 ) | 0.38 ( 0.31 to 0.45 ) | 1011.92 ( 867.82 to 1185 ) | 83.92 ( 82.56 to 85.16 ) | 90197.9 ( 89011.85 to 91304.08 ) | 17.2 ( 14.49 to 20.23 ) | 27901.25 ( 23942.35 to 32436 ) |
| Thailand | 1137.72 ( 1088.8 to 1189.51 ) | 159014.32 ( 150846 to 167366.15 ) | 3.8 ( 2.92 to 4.82 ) | 389.6 ( 301.02 to 492.65 ) | 661.34 ( 656.75 to 665.72 ) | 91039.08 ( 89957.24 to 92036.01 ) | 154.12 ( 125.63 to 186.09 ) | 17021.68 ( 14011.12 to 20580.12 ) |
| Timor-Leste | 20.82 ( 19.53 to 22.03 ) | 159471.69 ( 151287.6 to 167848.3 ) | 0.05 ( 0.04 to 0.06 ) | 713.97 ( 601.72 to 792.73 ) | 11.88 ( 11.69 to 12.05 ) | 91150.41 ( 90033.19 to 92101.26 ) | 2.21 ( 1.79 to 2.59 ) | 23076.33 ( 18918.39 to 26642.53 ) |
| Togo | 137.14 ( 128.38 to 146.36 ) | 179954.74 ( 171121.72 to 189479.25 ) | 0.21 ( 0.17 to 0.26 ) | 631.57 ( 535.33 to 758.85 ) | 71.65 ( 70.63 to 72.66 ) | 92776.54 ( 91818.45 to 93715.55 ) | 11.9 ( 9.69 to 14.23 ) | 22394.62 ( 18721.26 to 26673.87 ) |
| Tokelau | 0.02 ( 0.02 to 0.02 ) | 157321.58 ( 148785.28 to 165647.77 ) | 0 ( 0 to 0 ) | 762.52 ( 638.36 to 927.66 ) | 0.01 ( 0.01 to 0.01 ) | 90690.58 ( 89541.16 to 91767.39 ) | 0 ( 0 to 0 ) | 25437.27 ( 21255.59 to 30495.33 ) |
| Tonga | 1.6 ( 1.5 to 1.69 ) | 156915.49 ( 148996.86 to 165098.86 ) | 0.01 ( 0 to 0.01 ) | 692.94 ( 586.75 to 814.41 ) | 0.91 ( 0.9 to 0.93 ) | 90581.6 ( 89409.96 to 91672.31 ) | 0.2 ( 0.17 to 0.24 ) | 23714.12 ( 20038.29 to 27870.17 ) |
| Trinidad and Tobago | 26.05 ( 25.01 to 27.14 ) | 181599.59 ( 172917.71 to 190487.34 ) | 0.1 ( 0.08 to 0.13 ) | 577 ( 448.67 to 732.78 ) | 12.95 ( 12.84 to 13.05 ) | 90706.55 ( 89725.46 to 91672.28 ) | 3.65 ( 2.95 to 4.42 ) | 21717.17 ( 17636.43 to 26387.75 ) |
| Tunisia | 190.03 ( 181.32 to 199.28 ) | 161793.96 ( 153864.97 to 169992.15 ) | 0.59 ( 0.46 to 0.74 ) | 529.75 ( 421.38 to 661.48 ) | 107.74 ( 106.69 to 108.67 ) | 91718.6 ( 90589.31 to 92726.7 ) | 23.41 ( 19.25 to 28.37 ) | 19258.35 ( 15962.8 to 23287.07 ) |
| Turkey | 1327.26 ( 1266.86 to 1388.56 ) | 160760.77 ( 152354 to 168769.72 ) | 4.06 ( 3.41 to 4.81 ) | 496.86 ( 419.38 to 586.12 ) | 763.42 ( 757 to 769.45 ) | 91898.76 ( 90834.31 to 92932.46 ) | 164.02 ( 138.4 to 192.97 ) | 19194.55 ( 16313.8 to 22540.65 ) |
| Turkmenistan | 78.81 ( 75 to 82.9 ) | 157809.42 ( 150394.24 to 165350.99 ) | 0.29 ( 0.24 to 0.35 ) | 808.34 ( 673.53 to 978.56 ) | 45.5 ( 44.84 to 46.08 ) | 90002.31 ( 88740.05 to 91088.36 ) | 11.72 ( 9.87 to 13.88 ) | 26726.68 ( 22568.78 to 31453.26 ) |
| Tuvalu | 0.18 ( 0.17 to 0.19 ) | 156404.33 ( 148068.81 to 164819.55 ) | 0 ( 0 to 0 ) | 967.43 ( 783.55 to 1199.99 ) | 0.11 ( 0.11 to 0.11 ) | 90648.02 ( 89488.51 to 91688.72 ) | 0.03 ( 0.03 to 0.04 ) | 30835.83 ( 25442.53 to 37292.73 ) |
| Uganda | 690.02 ( 640.9 to 737.95 ) | 175460.25 ( 166467.1 to 184340.24 ) | 0.85 ( 0.72 to 1 ) | 619.66 ( 537.26 to 700.25 ) | 363.9 ( 357.95 to 370.07 ) | 92235.2 ( 91288.27 to 93187.58 ) | 52.88 ( 43.87 to 61.89 ) | 21890.06 ( 18796.14 to 25185.41 ) |
| Ukraine | 847.32 ( 815.47 to 882.55 ) | 177881.16 ( 169865.27 to 186209.42 ) | 6.32 ( 5.58 to 7.19 ) | 857.92 ( 757.34 to 977.75 ) | 412.98 ( 409.89 to 415.74 ) | 89744.43 ( 88540.45 to 90817.22 ) | 176.1 ( 154.18 to 201.09 ) | 27243.17 ( 23756.53 to 31211.46 ) |
| United Arab Emirates | 140.23 ( 133.28 to 147.98 ) | 153637.53 ( 146452.23 to 160860.89 ) | 0.21 ( 0.16 to 0.28 ) | 693.71 ( 590.87 to 820.47 ) | 87.97 ( 87.4 to 88.53 ) | 92006.31 ( 91154.2 to 92813.41 ) | 16.38 ( 13.4 to 19.9 ) | 23217.15 ( 19694.8 to 27258.14 ) |
| United Kingdom | 1332.24 ( 1288.64 to 1377.42 ) | 183854.31 ( 177429.57 to 190760.92 ) | 5.54 ( 5.48 to 5.62 ) | 409.88 ( 405.57 to 415.29 ) | 622.32 ( 618.88 to 625.6 ) | 88722.51 ( 87974.33 to 89468.8 ) | 173.91 ( 151.78 to 198.98 ) | 18000.62 ( 15317.11 to 21079.16 ) |
| United Republic of Tanzania | 934.27 ( 865.83 to 1003.14 ) | 171929.38 ( 162516.84 to 181043.08 ) | 1.47 ( 1.27 to 1.67 ) | 603.51 ( 532.59 to 673.71 ) | 500.05 ( 493.36 to 506.98 ) | 91747.56 ( 90910.41 to 92595.87 ) | 84.69 ( 71.92 to 98.83 ) | 21835.44 ( 18964.73 to 25004.33 ) |
| United States of America | 5214.91 ( 5037.22 to 5402.46 ) | 151272.86 ( 144370.8 to 158276.74 ) | 26.39 ( 26.15 to 26.65 ) | 458.92 ( 454.73 to 463.17 ) | 3059.75 ( 3038.41 to 3080.72 ) | 90320.56 ( 89349.26 to 91285.51 ) | 959.77 ( 839.21 to 1090.37 ) | 21717.37 ( 18649.42 to 25075.39 ) |
| United States Virgin Islands | 2.03 ( 1.95 to 2.11 ) | 185000.15 ( 176801.68 to 193633.28 ) | 0.01 ( 0.01 to 0.01 ) | 677.24 ( 605.49 to 740.99 ) | 0.98 ( 0.97 to 0.98 ) | 91309.25 ( 90262.01 to 92305.95 ) | 0.35 ( 0.3 to 0.39 ) | 22423.51 ( 19450.17 to 25752.55 ) |
| Uruguay | 57.28 ( 54.76 to 59.96 ) | 159634.07 ( 151511.1 to 168257.06 ) | 0.29 ( 0.28 to 0.3 ) | 496.24 ( 482.13 to 511.05 ) | 31.42 ( 31.08 to 31.74 ) | 88881.47 ( 87543.83 to 90135.46 ) | 8.57 ( 7.65 to 9.62 ) | 18881.72 ( 16586.44 to 21506.83 ) |
| Uzbekistan | 514.68 ( 486.52 to 544.34 ) | 157736.71 ( 150015.64 to 166097.8 ) | 1.68 ( 1.44 to 1.93 ) | 1253.49 ( 1121.45 to 1392.94 ) | 300.66 ( 296.24 to 304.56 ) | 90019.72 ( 88819.64 to 91118.91 ) | 70.6 ( 61.21 to 81.22 ) | 31275.16 ( 27580.51 to 35390.38 ) |
| Vanuatu | 4.53 ( 4.24 to 4.82 ) | 155369.8 ( 147414.51 to 163592.15 ) | 0.02 ( 0.01 to 0.02 ) | 1060.14 ( 885.14 to 1303.41 ) | 2.61 ( 2.56 to 2.65 ) | 90734.7 ( 89469.37 to 91822.39 ) | 0.69 ( 0.57 to 0.84 ) | 33457.86 ( 27724.24 to 40205.37 ) |
| Venezuela (Bolivarian Republic of) | 469.55 ( 449.52 to 489.96 ) | 166867.75 ( 159129 to 174717.55 ) | 1.46 ( 1.15 to 1.85 ) | 524.09 ( 414.37 to 661.61 ) | 254.22 ( 251.02 to 257.23 ) | 89505.54 ( 88224.43 to 90705.64 ) | 57.69 ( 47.19 to 69.75 ) | 19910.39 ( 16290.86 to 24036.86 ) |
| Viet Nam | 1585.05 ( 1508.13 to 1660.55 ) | 164548.9 ( 155815.05 to 173032.14 ) | 5.08 ( 4.34 to 5.79 ) | 622.9 ( 540.3 to 694.97 ) | 879.76 ( 870.84 to 888.14 ) | 89812.77 ( 88682.26 to 90869.2 ) | 194.3 ( 164.92 to 226.63 ) | 20466.24 ( 17490.64 to 23618.01 ) |
| Yemen | 484.63 ( 453.18 to 516.94 ) | 159342.66 ( 151479.24 to 167508.05 ) | 0.96 ( 0.8 to 1.19 ) | 793.45 ( 690.93 to 964.23 ) | 280.9 ( 276.25 to 285.42 ) | 91897 ( 90817.98 to 92916.71 ) | 52.79 ( 43.35 to 63.03 ) | 26255.88 ( 22128.86 to 31328.27 ) |
| Zambia | 303.27 ( 282.72 to 323.72 ) | 172156.94 ( 163274.01 to 180519.6 ) | 0.47 ( 0.39 to 0.57 ) | 740.99 ( 632.94 to 874.12 ) | 164.26 ( 161.79 to 166.69 ) | 92886.89 ( 91931.11 to 93833.49 ) | 26.5 ( 22.12 to 31.37 ) | 24596.16 ( 20859.75 to 28846.62 ) |
| Zimbabwe | 269.61 ( 253.79 to 285.32 ) | 187495.56 ( 178739.68 to 196121.46 ) | 0.48 ( 0.39 to 0.57 ) | 769.07 ( 634.84 to 912.84 ) | 130.48 ( 128.34 to 132.77 ) | 90261.26 ( 89222.89 to 91401.02 ) | 23.64 ( 19.86 to 27.91 ) | 25297.14 ( 21337.78 to 29749.17 ) |
